# Protein identification by 3D OrbiSIMS to facilitate in situ imaging and depth profiling

**DOI:** 10.1038/s41467-020-19445-x

**Published:** 2020-11-17

**Authors:** Anna M. Kotowska, Gustavo F. Trindade, Paula M. Mendes, Philip M. Williams, Jonathan W. Aylott, Alexander G. Shard, Morgan R. Alexander, David J. Scurr

**Affiliations:** 1 School of Pharmacy, University of Nottingham, Nottingham, NG7 2RD UK; 2 School of Chemical Engineering, University of Birmingham, Edgbaston Birmingham, B15 2TT UK; 3 National Physical Laboratory, Hampton Road, Teddington, Middlesex TW11 0LW UK

**Keywords:** Proteins, Molecular imaging, Mass spectrometry

## Abstract

Label-free protein characterization at surfaces is commonly achieved using digestion and/or matrix application prior to mass spectrometry. We report the assignment of undigested proteins at surfaces in situ using secondary ion mass spectrometry (SIMS). Ballistic fragmentation of proteins induced by a gas cluster ion beam (GCIB) leads to peptide cleavage producing fragments for subsequent Orbitrap^TM^ analysis. In this work we annotate 16 example proteins (up to 272 kDa) by de novo peptide sequencing and illustrate the advantages of this approach by characterizing a protein monolayer biochip and the depth distribution of proteins in human skin.

## Abstract (web summary)

Label-free protein characterization at surfaces requires digestion or matrix application prior to mass spectrometry. Here, the authors report the assignment of undigested proteins at surfaces by de novo sequencing and apply the methodology to a protein monolayer biochip and for in situ depth profiling of proteins through human skin.

## Introduction

Direct analysis of proteins such as in tissue^1^, at the surface of materials for use in the body^2^ and biochips^3,4^, offers insight into the molecular mechanism of disease and aids the development of medicines and medical devices. Label-free methods of imaging proteins are based on mass spectrometry (MS), with the most widely employed being matrix-assisted laser desorption/ionization (MALDI)^5^. MALDI allows lipids and small peptides to be imaged with high lateral resolution (1.4 μm)^6^. However, routine analysis of proteins with this technique uses enzymatic digestion and the application of a matrix, which may modify the structure of the sample surface and consequently the spatial distribution of analytes in two and three dimensions^7^. Other methods used for protein characterization at surfaces are desorption electrospray ionization^8^ and liquid extraction surface analysis MS^9^; however, these techniques have limited lateral resolution (>100 μm)^10^.

Time-of-flight secondary ion MS (ToF-SIMS) is a surface analysis technique able to provide in situ label- and matrix-free chemical information from sample surfaces and in three dimensions (3D) (from a few nanometers to several hundred micrometers into the sample)^11^. Analysis of proteins in ToF-SIMS has been limited, because they are heavily fragmented by the energetic primary ion beams traditionally applied for imaging, resulting in only single amino acid residue secondary ions which are devoid of any primary structural information. In addition, analysis of macromolecules in ToF-SIMS is limited by the mass-resolving power of the ToF analyser. Information about protein identity, conformation or orientation can be obtained using individual amino acid fragment intensities by statistical analysis as shown by Wagner and Castner^12,13^. This fingerprinting approach is however limited to comparison of known protein samples and their mixtures^14,15^. Multi-amino acid fragments and molecular ions of peptides up to 1.6 kDa can also be detected by small polyatomic metal primary ion beams Bi_3_^+^ and Au_3_^+^^16^. The emergence of large cluster primary ion source analysis beams, such as C_60_^+^ and gas cluster ion beams (GCIBs) such as Ar_*n*_^+^, has enabled more information to be derived from larger peptides up to 3 kDa in ToF-SIMS^17^ and, recently, the detection of whole proteins up to 12 kDa has been reported^18^. Nonetheless, large proteins have yet to be identified without pre-analysis digestion to constituent peptides.

Recently, the 3D OrbiSIMS instrument which combines a GCIB and an Orbitrap^TM^ analyser has been developed to aid analysis of biological samples^19^. Here we describe findings illustrating how this combination can be used to achieve in situ label- and matrix-free three-dimensional profiling and mapping of undigested proteins at surfaces.

## Results

### Peptidic fragments are generated by Argon GCIB

**Fig. 1:**
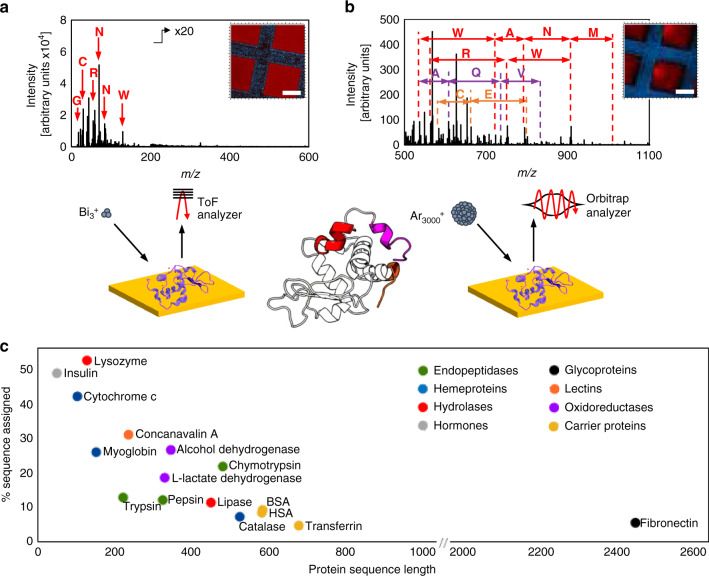
Comparison of Bi_3_^+^ primary ion beam ToF-SIMS and Ar_3000_^+^ primary ion beam 3D OrbiSIMS analysis of lysozyme. **a** In the positive ion secondary mass spectrum obtained using ToF-SIMS, only single amino acid fragments can be identified. **b** In the GCIB 3D OrbiSIMS data, protein fragments larger than single amino acids are generated due to the GCIB bombardment. Amino acid neutral losses, W-tryptophan, A-alanine, N-asparagine, M-methionine, R-arginine, Q-glutamine, T-tyrosine and V-valine can be assigned due to the high mass-resolving power and mass accuracy of the Orbitrap^TM^ analyser. The amino acid sequences are highlighted in the lysozyme structure, exported from PDB entry1AZF^38^. In the associated LMIG ToF-SIMS and GCIB 3D OrbiSIMS images of lysozyme film under a gold grid, blue represents the gold signal and red represents a sum of (**a**) amino acid fragments listed in Supplementary Table 1 and (**b**) assigned peptide fragments listed in Supplementary Fig. 6. The scale bars represent 50 μm. **c** Sixteen proteins of different biological functions were analysed. From 5% (transferrin) to 53% (lysozyme) sequence coverage was achieved for a range of sizes from 51 to 2446 amino acids.

Only single amino acids are observed in the ToF-SIMS Bi_3_^+^ spectrum (Fig. 1a and Supplementary Fig. 1) of lysozyme (14 kDa) and peptidic secondary ions resulting from ballistic fragmentation are observed in the 3D OrbiSIMS spectra acquired using an Ar_3000_^+^ primary ion beam (Fig. 1b). The mass-resolving power of 240,000 and mass accuracy (<2 p.p.m.) of the Orbitrap^TM^ analyser allowed the assignment of characteristic amino acid neutral losses between peptide fragments of the protein with high certainty enabling protein identification by de novo sequencing illustrated for lysozyme in Supplementary Fig. 2.

We assessed the method using 16 model protein films covering a range of functions and sizes from insulin to fibronectin at 6 and 272 kDa respectively (Fig. 1c). Sequence coverages from 5% (33 of 679 amino acids for transferrin) to 53% (68 of 129 amino acids for lysozyme) can be readily assigned (Fig. 1c and Supplementary Table 2) in the positive polarity spectra. The decrease in the proportion of the protein sequence that is assigned as protein size increases reflects a limit in the number of peptides that can be extracted from the data using this approach. The MS/MS capability of the 3D OrbiSIMS instrument further confirms the sequencing for selected ions, as shown in the example of insulin in Supplementary Fig. 3.

The peptidic secondary ions are derived from ballistic fragmentation of the intact proteins, in contrast to enzymatically digested proteins. Therefore, they cannot be assigned using existing databases employed for peptide fingerprint identification. However, the application of de novo sequencing identification of proteins directly from primary ion-induced fragments is explored herein. Unlike MS/MS of peptides, the peptidic ions observed in the positive SIMS spectra are found to be mostly sodium adducts of neutral protein fragments (Supplementary Tables 10–147). Moreover, in addition to b and y ions, normally seen in collision-induced ionization (CID)^20^, the spectra obtained with the 20 keV Ar_3000_^+^ GCIB also contain a, c and z ions characteristic to other methods of ionization (Supplementary Fig. 4) such as electron capture dissociation (ECD)^21^. Lastly, in addition to N- and C-terminal ions, a large proportion of the peaks observed in the spectrum are internal fragments of the sequence, not just those derived from the chain ends. Similar to CID fragmentation, cysteine residues participating in disulphide bonds have been found to form R-S and R-SH_2_ ions resulting from partial side chain cleavage (Supplementary Table 3)^22,23^. The presence of internal ions, formed when a y ion undergoes further fragmentation into ya, yb, yc and ya-NH_3_ ions is similar to high-energy collision dissociation^24^. The proposed structure of an Ar_3000_^+^ induced internal fragment ya is presented on an example NAWVA sequence shown in Supplementary Fig. 5a. At the C termini, we detected y ions (typical of CID) and *z*, *z* + 1 and *z* + 2 ions, characteristic of MALDI in-source decay^25–27^. The fragment structure of a *z* + 1 ion is proposed on an example RGCRL sequence in Supplementary Fig. 5b. The identified sequences include a number of biologically important binding and active sites listed in Table 1. Full assignments of the identified sequences are presented in Supplementary Tables 10–147.

**Table 1:** Examples of functional sites detected in the 16 analysed proteins.

Functional site	Location in the amino acid sequence	Protein
Haem-binding site	H18 and M80	Cytochrome *c*
NAD-binding site	R99	l-lactate dehydrogenase
	R341	Alcohol dehydrogenase
Substrate-binding site	T248	l-lactate dehydrogenase
Carbonate-binding site	T139, T471	Transferrin
Metal-binding site	H66	Alcohol dehydrogenase
	H604	Transferrin
	H3, E6, D13, H67, D248	Bovine serum albumin
	E8, D10	Concanavalin A
Active site	H74	Catalase
Fibrin- and heparin-binding site	C53-R273, T1813-T2083, C2298-G2429	Fibronectin
Collagen-binding site	C309-T609	Fibronectin
Cell attachment site	V1359-T1632	Fibronectin
Charge relay system	C194	Trypsin
	H57, D102, S195	Chymotrypsin

The location of the functional sites is based on UniProt database^28^.

Analysis of the spectra of similar proteins revealed that both common and distinct fragments can be identified. Bovine serum albumin (BSA) and human serum albumin (HSA) have 76% identical sequences (441 of 584 amino acids) calculated by protein BLAST^28^. Their spectra contain 32% (1178 of 3709 peaks) common peaks. Common sequences and species-specific sequences were detected in the 3D OrbiSIMS spectra and are presented in Supplementary Fig. 26.

The negative polarity spectra in proteomics can provide additional information to complement the positive polarity^29,30^. This work focuses on the analysis of positive polarity data in order to place this emerging technology in the context of the widely used CID and MALDI methods. However, analogous fragmentation patterns were observed in the negative polarity spectra acquired with Ar_3000_^+^ GCIB and Orbitrap^TM^, as shown in the example of bovine α-chymotrypsin (Supplementary Tables 148–152). Ions observed in negative polarity are observed as the deprotonated N terminus fragments *a*, *b*, *c* and deprotonated C terminus fragments *y*, *z*-H and *x* ions. These types of ions are consistent with negative polarity ions reported in previous studies for methods such as CID^31^ or ECD^21^.

### Protein fragments can be used for imaging and mixture deconvolution

The ability to image the proteins with high chemical specificity and high lateral resolution was demonstrated by masking a thin protein film (300 nm) using a transmission electron microscopy grid (Fig. 1b and Supplementary Fig. 6). The 3D OrbiSIMS imaging achieved lateral resolution of 10 μm using the sum of 27 of the characteristic peptidic fragments (Supplementary Fig. 6).

**Fig. 2:**
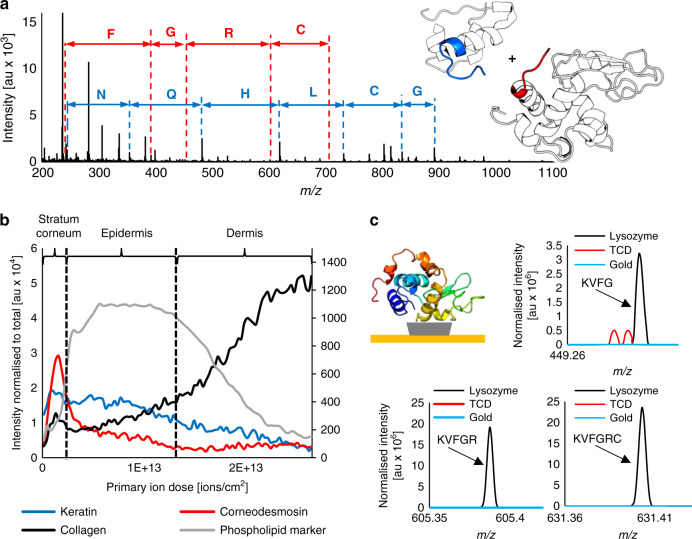
The applications of the ballistic sequencing in surface analysis. **a** Sequences of lysozyme (red) and insulin (blue) were simultaneously detected, allowing imaging of a protein mixture. The identified sequences are highlighted in insulin and lysozyme structures, exported from PDB entries 3I40^37^ and 1AZF^38^, respectively. **b** Three proteins were profiled in situ in a human skin sample. The dashed lines indicate borders between the skin layers, assigned based on the profile of phospholipid marker (PO_3_^−^). PO_3_^−^ is presented on a secondary scale for clarity due to high intensity throughout the profile. Example peaks of keratin (*stratum corneum* and epidermis, blue), corneodesmosin (*stratum corneum*, red), and collagen (dermis, black). (**c**) Characteristic lysozyme fragments representing the KVFGRC sequence were detected in a protein biochip, consisting of a protein monolayer immobilized on heptakis-(6-deoxy-6-thio)-β-cyclodextrin (TCD).

To further test the limits of 3D OrbiSIMS analysis of proteins, we analysed an equimolar mixture of lysozyme and insulin (Fig. 2a). It was possible to assign unique sequences from both lysozyme and insulin, allowing the unambiguous identification of both proteins in the mixture (Supplementary Figs. 7 and 8).

### Protein distribution in human skin

The capability of the method to identify and locate proteins within complex 3D biological samples was assessed by depth profiling through human skin (Fig. 2b and Supplementary Fig. 9). We detected three proteins, corneodesmosin, keratin and collagen, known to be abundant in the *stratum corneum*, epidermal and dermal layers, respectively. Sequences characteristic to these proteins were manually assigned in situ in the skin layers where they are known to be present exclusively or predominantly. The layers of the skin in the depth profile, specifically the *stratum corneum*, underlying epidermis and dermis, were assigned based on the abundance of a phospholipid marker (PO_3_^−^), as described in previous analysis of skin in SIMS by Starr et al.^32^ (Fig. 2c). Protein locations in the skin layers were based on the immunochemically stained tissue sections gathered in the Protein Atlas^33^.

Hydroxyproline is an indicator of collagen, as it forms 13.5% of the collagen sequence and is rarely found in other proteins^34^. The intensity of hydroxyproline, metoxyproline and fragments of hydroxyproline, as well as the PGE sequence all increased with the depth of analysis, reaching a maximum in the dermis region (Supplementary Fig. 10 and Supplementary Table 4). Similarly, the characteristic sequence QHGSG was assigned to corneodesmosin, present in the *stratum corneum* (Supplementary Fig. 11 and Supplementary Table 5) and SFGGGG sequence assigned to keratin, abundant throughout the *stratum corneum* and the epidermis (Supplementary Fig. 12 and Supplementary Table 6). The method, together with the knowledge of skin composition, allowed targeted assignment of corneodesmosin, keratin and collagen in situ, which indicates the capability to profile proteins in complex biological systems.

### Analysis of a protein monolayer biochip

The method developed here allows the analysis of proteins at surfaces without any pre-treatment, which is particularly advantageous for investigating protein distribution on materials when the amount of the protein on the surface is in pico- or femtomolar range. We assessed the efficacy of the 3D OrbiSIMS in these challenging biological applications by analysing a biochip produced using a method developed by Di Palma et al.^35^ consisting of a lysozyme monolayer immobilized on a self-assembled monolayer (SAM) of heptakis-(6-deoxy-6-thio)-β-cyclodextrin (TCD) (Fig. 2c). The samples were confirmed to be a monolayer of proteins by ellipsometry and X-ray photoelectron spectroscopy (XPS), as illustrated in Supplementary Fig. 13. Seven diagnostic fragment ions from the N and C terminus of lysozyme were detected in the samples confirming lysozyme immobilization on the biochip. This illustrates that the sensitivity of the analytical technique is sufficient to detect and assign monolayer protein coverage (Supplementary Note 1).

### Automated sequence search and protein identification

**Fig. 3:**
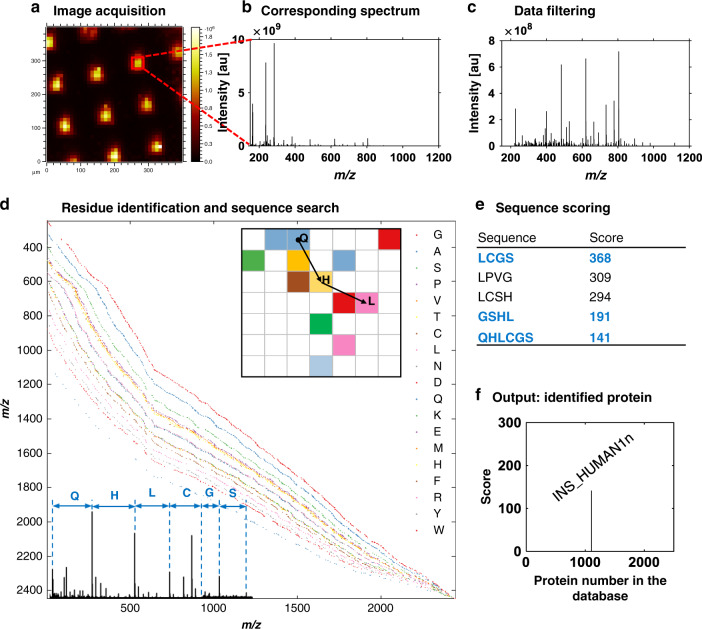
Workflow for automated protein identification directly from a surface. **a** Secondary ion image of an example protein. **b** Acquired spectrum from protein region. **c** The data requires processing consisting of chemical and intensity filtering. **d** The script detects amino acid neutral losses and continuous sequences in the processed spectra. **e** Detected sequences are scored against the UniProt database. **f** Possible proteins are presented in the graphical output to assess ambiguities.

Computational analysis of the human protein sequences listed in the UniProt database^28^ showed that 95% of the proteins in the database contain an eight-residue sequence that is unique to each particular protein (Supplementary Fig. 15b). We manually assigned 8- to 13-membered sequences in the spectra of model proteins; therefore, we propose that the GCIB ballistic sequencing method produces sufficient information to be used for protein identification (Supplementary Note 2). Existing protein identification software^36^ is, however, unsuitable for the data produced by the combination of GCIB and Orbitrap^TM^; therefore, a set of functions for de novo sequencing was developed in MATLAB. Protein identification is achieved in five steps illustrated in Fig. 3c–f as follows: chemical filtering of the OrbiSIMS data to identify peptides, intensity filtering to remove the background signals, residue identification, sequence search against a protein database, sequence scoring and protein ranking. The script is described in detail in Supplementary Note 3 and the code is available online (https://github.com/guferraz/simsdenovo/). The functions were used to automate sequence assignment of 16 known proteins and allowed unambiguous confirmation of the identity of 6 proteins (Supplementary Table 9). Future development of the script will include expanding a database of protein fingerprints, optimizing data filtering and sequence scoring to aid identification of unknown proteins and indicate the presence of proteins in complex samples.

## Discussion

In summary, this work uses the 3D OrbiSIMS to achieve the first matrix- and label-free in situ assignment of undigested proteins at surfaces. A bottom-up de novo sequencing approach demonstrated sufficient sequence coverage for the correct identification of 16 example proteins up to 272 kDa. Beneficial aspects of the method include the capability to image proteins with high chemical specificity, a lateral resolution of 10 μm, sufficient sensitivity to achieve the analysis of a protein monolayer and the accurate assignment of proteins in a mixture. A protein monolayer biochip has been analysed to demonstrate the suitability of the method in the development of biosensors and materials reliant upon adsorbed protein layers. The capability to map proteins in a challenging complex biological environment is demonstrated by targeted assignment of collagen, keratin and corneodesmosin within specific layers of the human skin. Further applications of this approach include obtaining information unavailable by classical methods due to limited solubility of the protein and analysis of protein fouling of medical device materials. The method can be utilized on any instrument with a combined GCIB primary ion beam and analyser with mass-resolving power better than 240,000. An automated spectral search is proposed for assignment of protein sequences in known model protein samples. This work is a step towards adapting conventional bottom-up approach to analysing proteins at surfaces; however, similar to routine proteomic methods, confident untargeted high-throughput identification of proteins remains a challenge for extended studies.

## Methods

### Sample preparation

*Proteins*: lysozyme from chicken egg white, α-chymotrypsin from bovine pancreas, insulin solution human, recombinant BSA, horse skeletal muscle myoglobin, l-lactate dehydrogenase from rabbit muscle, human holo-transferrin, concanavalin A from Jack Bean, bovine plasma fibronectin, alcohol dehydrogenase from *Saccharomyces cerevisiae*, porcine lipase, bovine liver catalase, HSA and cytochrome *c* from equine heart were purchased from Sigma Aldrich. Porcine pepsin and porcine trypsin were purchased from Promega. Heptakis-(6-deoxy-6-thio)-β-cyclodextrin was purchased from Cyclodextrin-Shop (Tilburg, The Netherlands) to >97% purity. Hydrogen peroxide (H_2_O_2_) 30% w/w solution, sulfuric acid (H_2_SO_4_) reagent grade 95–95% and *N*,*N*-dimethylformamide (DMF) for molecular biology ≥ 99% were purchased from Sigma Aldrich. Amicon Ultra 0.5 mL Centrifugal Filters were purchased from Merck Milipore. Human skin was purchased from Tissue Solutions (https://www.tissue-solutions.com/). Female >40 years of age, ex vivo skin from cosmetic surgery. Insulin, lysozyme and chymotrypsin were purified for the 3D OrbiSIMS analysis. Protein purification was done using Milipore Amicon Ultracentrifuge filter units. The purification was done by following the protocol provided by Milipore: 0.5 mL of 100 μM protein solution in MiliQ water (18 Ω) was placed in the Amicon filter units. The units were centrifuged for 30 min at 14,000 × *g*. The protein concentrate was recovered by inverting the filter unit in a clean tube and centrifugation for 2 min at 1000 × *g*. The protein concentration was recovered to 100 μM by refilling the solution with MiliQ water to 0.5 mL. Gold slides were cleaned by ultraviolet and sonication in isopropanol for 10 min, subsequently rinsed with MiliQ water and dried under Argon flow. Purified proteins were spotted and dried onto a gold slide three times to obtain a thick protein film. Protein : peptide mixture was prepared by 1:1 volumetric mixing of 100 μM insulin and 100 μM lysozyme concentrations. The mixture of purified lysozyme and insulin was spotted and dried onto a gold slide three times to obtain a thick protein film. Gold grids for electron transmission microscopy of 200 mesh × 125 μm pitch were purchased from Sigma Aldrich. For the imaging experiment, the bare gold grid was placed on top of the protein film sample. A bare gold slide and a bare gold grid have been analysed as control samples.

For the preparation of protein monolayer biochips, the gold substrates were cleaned by immersion in piranha solution (70% H_2_SO_4_, 30% H_2_O_2_) at room temperature for 8 min, rinsed with MiliQ water and dried with an argon flow (caution: Piranha solution reacts violently with organic solvents and should be handled with care). The clean gold substrates were immersed for 24 h in 50 μM DMF solutions of heptakis-(6-deoxy-6-thio)-β-cyclodextrin (TCD) for preparation of a self assembled monolayer (SAM). Subsequently, the gold substrates were rinsed with DMF and MiliQ water, and dried under an argon flow. The TCD SAMs were immersed in a 0.05 mM lysozyme in PBS solution for 2 h. Following protein immobilization, the samples were washed with PBS buffer followed by submersion in MiliQ water for 1 min. The samples were then dried under argon and placed in the instrument for the analysis immediately.

### Instrument calibration

Calibration of positive mode ToF-SIMS spectra was made using CH_3_^+^, C_2_H_3_^+^, C_3_H_5_^+^, C_7_H_7_^+^, and Au^+^ ions.

Calibration of the Orbitrap analyser was performed on the silver sample, using silver clusters following the method described by Passarelli et al.^19^. The Bi_3_^+^ liquid metal ion gun and the ThermoFisher Tune software were employed for calibration.

### Data acquisition

For the acquisition of the 3D OrbiSIMS spectra, a 20 keV Ar_3000_^+^ analysis beam of 20 µm diameter, was used as primary ion beam. Ar_3000_^+^ with duty cycle set to 4.4% and GCIB current was 218 pA. The Q Exactive depth profile was run on the area of 200 × 200 µm using random raster mode with crater size 280 × 280 µm. The cycle time was set to 400 μs. Optimal target potential varied for different samples, oscillating at approximately +68 V. Argon gas flooding was in operation in order to aid charge compensation, pressure in the main chamber was maintained at 9.0 × 10^−7^ bar. The spectra were collected in positive polarity, in mass range 150–2250 *m*/*z*. The injection time was set to 500 ms. Three separate areas were analysed on each sample and each measurement lasted 30 scans, the total ion dose per measurement was 1.63 × 10^11^. Mass-resolving power was set to 240,000 at 200 *m*/*z*.

For the acquisition of the 3D OrbiSIMS MS/MS, a 20 keV Ar_3000_^+^ analysis beam of 20 µm diameter, was used as primary ion beam. Ar_3000_^+^ with duty cycle set to 4.4% and GCIB current was 218 pA. The Q Exactive depth profile was run on the area of 200 × 200 µm using random raster mode with crater size 280 × 280 µm. The cycle time was set to 400 μs. The target potential was set to +68 V. Argon gas flooding was in operation in order to aid charge compensation, pressure in the main chamber was maintained at 9.0 × 10^−7^ bar. The spectra were collected in positive polarity, in mass range 75–1125 *m*/*z*. The injection time was set to 500 ms. Three separate areas were analysed on the sample and each measurement lasted 20 scans, the total ion dose per measurement was 1.63 × 10^11^. Mass-resolving power was set to 240,000 at 200 *m*/*z*. Normalized collision energy was set to 35, isolation width was 10 u.

For the acquisition of the 3D OrbiSIMS image, a 20 keV Ar_3000_^+^ analysis beam of 20 µm diameter (imaging of the protein mixture) or a 20 keV Ar_3000_^+^ imaging beam of 5 µm diameter (imaging of lysozyme film masked with a gold grid) was used as primary ion beam. The 20 µm analysis beam was configured as described in the spectra acquisition section. The 5 µm imaging beam duty cycle set to 37.7% and GCIB current was 18 pA. The Q Exactive images were run on the area of 200 × 200 µm using random raster mode. The cycle time was set to 400 μs. Optimal target potential was set to +58 V. Argon gas flooding was in operation; to aid charge compensation, pressure in the main chamber was maintained at 9.0 × 10^−7^ bar. The images were collected in positive polarity, in mass range 150–2250 *m*/*z*. The injection time was set to 500 ms. Three separate areas were analysed on each sample and each measurement lasted one scan, the total ion dose per measurement was 1.11 × 10^12^. Mass-resolving power was set to 240,000 at 200 *m*/*z*.

For the acquisition of the 3D OrbiSIMS depth profile of human skin, a 20 keV Ar_3000_^+^ analysis beam of 20 µm diameter was used as the primary ion beam. Ar_3000_^+^ with duty cycle set to 15% and GCIB current was 230 pA. The Q Exactive depth profile was run on the area of 200 × 200 µm using random raster mode with crater size 280 × 280 µm. The cycle time was set to 200 μs. Optimal target potential varied for different samples, oscillating at approximately −384.4 V. The acquisition was run in temperature −170 °C. The depth profile was collected in negative polarity, in mass range 75–1125 *m*/*z*. The injection time was set to 500 ms. The measurement lasted 18,307 scans and the total ion dose per measurement was 2.65 × 10^13^. Mass-resolving power was set to 240,000 at 200 *m*/*z*.

For the acquisition of liquid metal ion gun (LMIG) ToF-SIMS image, a 30 keV Bi_3_^+^ primary beam was used. LMIG current was 0.05 pA. The ToF image was run on the area of 200 × 200 µm using random raster mode. The cycle time was set to 250 ms. Optimal target potential was set to +58 V. Three separate areas were analysed on each sample and each measurement lasted 15 scans; the total ion dose per measurement was 9.44 × 10^10^.

The depth of the sputtered material was estimated by the SurfaceLab software as 10 nm per scan and confirmed by profilometry as 300 nm after acquisition of 30 scans. Optical profilometry scans were obtained using a Zeta-20 optical microscope (Zeta Instruments, CA, USA). The scans were acquired in a *Z* range of 4.6 µm. The number of steps was set to 328, allowing for step size (*Z* resolution) of 0.014 µm.

The SAM and the SAM with immobilized lysozyme were measured using an Axis-Ultra XPS instrument (Kratos Analytical, UK) with monochromated Al Kα X-ray source, based at the National Physical Laboratory, Teddington, UK. Wide scans were acquired for analysis of organic layer thickness on the gold slide with step size 1000 meV, pass energy 160 eV, dwell time 300 ms, in a range of 1300 to −10 eV.

The thickness of the SAM and the SAM with immobilized lysozyme was measured using J.A. Woollam Co. Inc. alpha-SE^TM^ spectroscopic ellipsometer. The CompleteEASE software was employed to determine the thickness values and the calculations were based on a two-phase organic/Au model, in which the organic layer was assumed to be isotropic and assigned a refractive index of 1.50. The thickness reported is the average of three different measurements on SAM or SAM and protein samples, with the errors reported as SD.

### Data analysis

Thermo Xcalibur 3.1.66.10 and IonToF SurfaceLab 7.1.116182 were used to process the results and assign the peaks. Xcalibur was used to identify amino acid neutral losses between peaks and SurfaceLab was used to create the peak lists. SurfaceLab was used to measure lateral resolution of ToF-SIMS and 3D OrbiSIMS images. Ellipsometry results were analysed using CompleteEASE 4.06. XPS results were analysed using CasaXPS 2.3.19PR1.0. Protein illustrations with highlighted sequences were generated using PyMol^TM^ 2.3.2. on the structures exported from PDB, referenced for each protein in the Supplementary Information. Proposed chemical structures of observed protein fragments were produced using ChemDraw Professional 16.0. A custom code was written in MATLAB for automated sequence assignment and protein identification. The code is available on Github (https://github.com/guferraz/simsdenovo/) and described in Supplementary Note 3.

### Reporting summary

Further information on research design is available in the Nature Research Reporting Summary linked to this article.

## Supplementary information

Supplementary Information

Reporting Summary

## Data Availability

All data have been placed in Nottingham Research Data Management Repository and is available at https://rdmc.nottingham.ac.uk/handle/internal/8604 (10.17639/nott.7070). PDB entries 1HRC, 1AZF, 1WLA, 1EPT, 4PEP, 1AB9, 4W6Z, 1JBC, 4F5S, 1AO6, 5NQB, 3RE8 and 3I40 were used for structural visualization and can be accessed at https://www.rcsb.org/.

## Code availability

The code for automated sequence assignment and protein identification is available on Github (https://github.com/guferraz/simsdenovo/) and described in Supplementary Note 3.
