# Supplementary material for: Protein identification by 3D OrbiSIMS to facilitate in situ imaging and depth profiling
Source: Nat Commun. 2020 Nov 17;11:5832. doi: 10.1038/s41467-020-19445-x (PMC7672064; doi:10.1038/s41467-020-19445-x)
Supplement: Supplementary file 1 — Supplementary Information [file 41467_2020_19445_MOESM1_ESM.pdf]

Supplementary Information - Protein identification by 3D OrbiSIMS to facilitate *in situ* imaging and depth profiling

Anna M. Kotowska[1], Gustavo F. Trindade[1], Paula M. Mendes[2], Philip M. Williams[1], Jonathan W. Aylott[1], Alexander G. Shard[3], Morgan R. Alexander[1] and David J. Scurr[1]*

[1] School of Pharmacy, University of Nottingham, Nottingham NG7 2RD, United Kingdom

[2] School of Chemical Engineering, University of Birmingham, Edgbaston, Birmingham B15 2TT

[3] National Physical Laboratory, Hampton Road, Teddington, Middlesex TW11 0LW, United Kingdom

*email: david.scurr@nottingham.ac.uk

Supplementary Table 1 Amino acid fragments used in ToF-SIMS of proteins, first assigned by Wagner and Castner [1]. The listed ions were used in the Bi_3^+ ToF-SIMS image (Figure 1a).

number	*m/z*	Assignment	Possible fragment origin
1	30.0351	CH_4N^+	G
2	44.0119	CH_2NO^+	N
3	44.0500	$C_2H_6N^+$	A
4	44.9775	CHS^+	C
5	56.0520	$C_3H_6N^+$	K
6	59.0483	$CH_5N_3^+$	R
8	61.0099	$C_2H_5S^+$	M
9	68.0506	$C_4H_6N^+$	P
11	70.0269	$C_3H_4NO^+$	N
12	70.0673	$C_4H_8N^+$	R
13	71.0089	$C_3H_3O_2^+$	S
14	72.0431	$C_3H_6NO^+$	A
15	72.0804	$C_4H_{10}N^+$	V
16	74.0583	$C_3H_8NO^+$	T
17	76.0199	$C_2H_6SN^+$	C
18	81.0345	$C_4H_5N_2^+$	H
19	83.0474	$C_5H_7O^+$	V
20	84.0397	$C_4H_6NO^+$	E/Q
21	84.0842	$C_5H_{10}N^+$	I, L, K
22	86.0988	$C_5H_{12}N^+$	I, L
23	87.0504	$C_3H_7N_2O^+$	N
24	88.0379	$C_3H_6NO_2^+$	D
25	98.0192	$C_4H_4NO_2^+$	N
26	100.0820	$C_4H_{10}N_3^+$	R
27	102.0533	$C_4H_8NO_2^+$	E
28	107.0443	$C_7H_7O^+$	T
29	110.0746	$C_5H_8N_3^+$	H
30	120.0783	$C_8H_{10}N^+$	F
31	130.0562	$C_9H_8N^+$	W
32	136.0749	$C_8H_{10}NO^+$	T
33	145.0937	$C_{10}H_{11}N^+$	W
34	159.0808	$C_{10}H_{11}N_2^+$	W
35	170.0616	$C_{11}H_8NO^+$	W

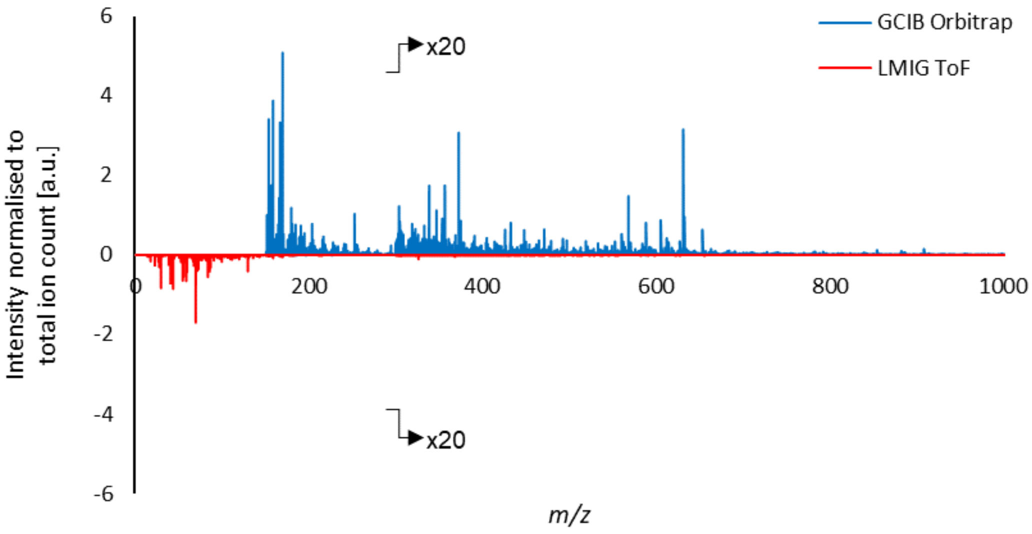

Supplementary Figure 1 Positive mode 3D OrbiSIMS spectrum of lysozyme (blue) and positive mode LMIG ToF-SIMS spectrum of lysozyme (red). Argon gas cluster ion beam (GCIB) results in large multi amino acid fragments, which can be detected and assigned with high accuracy by the Orbitrap™ analyser. Spectra intensities have been normalised to total ion count.

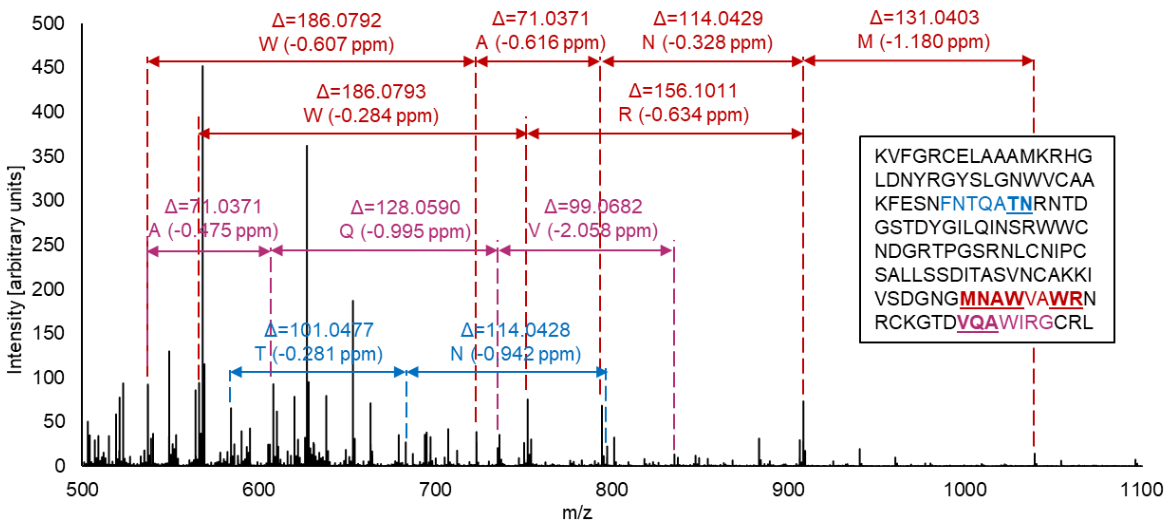

Supplementary Figure 2 Positive mode 3D OrbiSIMS spectrum of lysozyme highlighting example sections of amino acid sequence (red, blue, purple), explained by the peptide fragments assigned in the spectrum. In the example section of the protein sequence, colour coded bold amino acid residues are a result of the direct observations and coloured non-bold adjacent residues are inferred from the database protein sequence. Values in the brackets show deviation of each residue assignment. Amino acid neutral losses can be assigned with confidence due to high mass accuracy of the Orbitrap™ analyser. The total ion dose per measurement was 1.63×10^{11}.

Supplementary Table 2 List of analysed proteins from the smallest (insulin, 51 amino acid sequence) to the largest (fibronectin, 2446 amino acid sequence). Sequence coverage achieved with the 3D OrbiSIMS is presented as number of assigned amino acids and fraction of the whole protein sequence.

Name	UniProt database code	Taxonomy	Length of the protein sequence (amino acids)	Number of assigned amino acids	% of the sequence assigned
Insulin	INS_HUMAN	9606	51	25	49.02
Cytochrome c	CYC_HORSE	9796	104	44	42.31
Lysozyme	LYSC_CHICK	9031	129	68	52.71
Myoglobin	MYG_HORSE	9796	153	40	26.14
Trypsin	TRYP_PIG	9823	223	29	13.00
Concanavalin A	CONA_CANEN	3823	237	74	31.22
Pepsin	PEPA_PIG	9823	326	40	12.27
L-lactate dehydrogenase	LDHA_RABIT	9986	331	62	18.73
Alcohol dehydrogenase	ADH1_YEAST	559292	347	93	26.80
Lipase	LIPL_PIG	9823	451	52	11.53
Chymotrypsin	CTRA_BOVIN	9913	482	47	9.75
Catalase	CATA_BOVIN	9913	526	39	7.41
BSA	ALBU_BOVIN	9913	583	55	9.43
HSA	ALBU_HUMAN	9606	585	50	8.58
Transferrin	TRFE_HUMAN	9606	679	33	4.86
Fibronectin	FINC_BOVIN	9913	2446	139	5.68

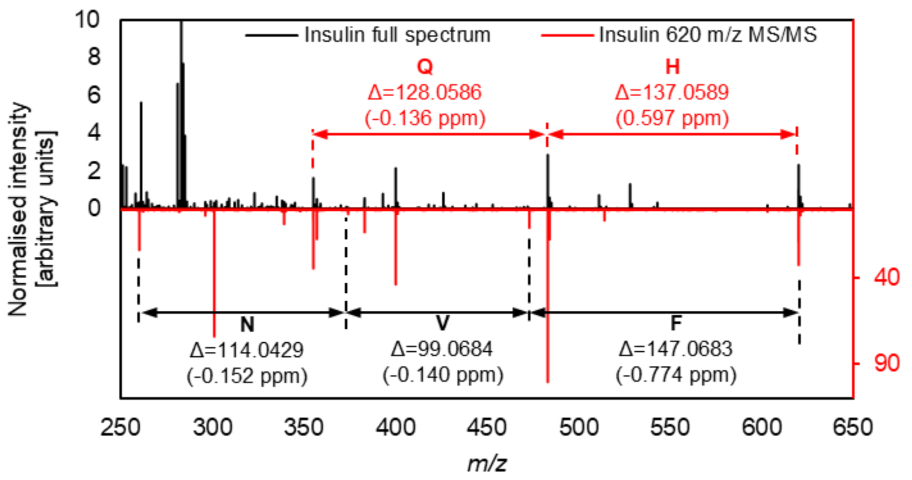

Supplementary Figure 3 Inverted overlay comparison of full 3D OrbiSIMS spectrum of insulin (black) and 3D OrbiSIMS MS/MS of the 620.29 *m/z* (red) ion, assigned as the first 5 amino acids in the insulin chain b sequence: FVNQH. All labelled amino acid neutral losses are present in both the full spectrum and the MS/MS spectrum, which confirms the suggested fragmentation. The peak at 300.05 *m/z* in MS/MS spectrum (red) is an instrument artefact and should be ignored[2].

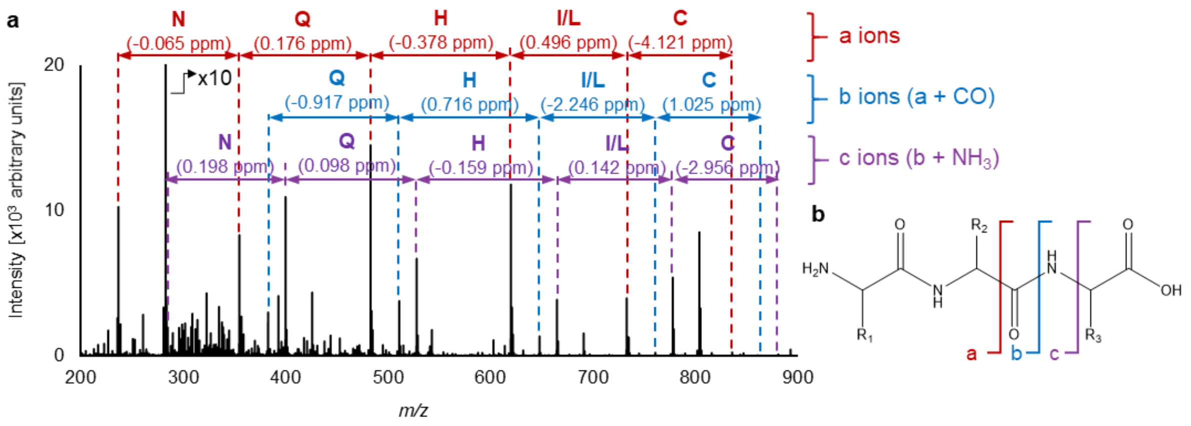

Supplementary Figure 4 (a) 3D OrbiSIMS of thin (300 nm) insulin film. The first 8 amino acids of the insulin B chain (FVNQHLC) dominate the spectrum. Each peptide fragment (FVN, FVNQ, FVNQH etc.) is observed as a-, b- and c-type ion. (b) N-terminus ions a, b and c are formed when different bonds related to the peptide bond are broken. The total ion dose per measurement was 1.63×10^{11}.

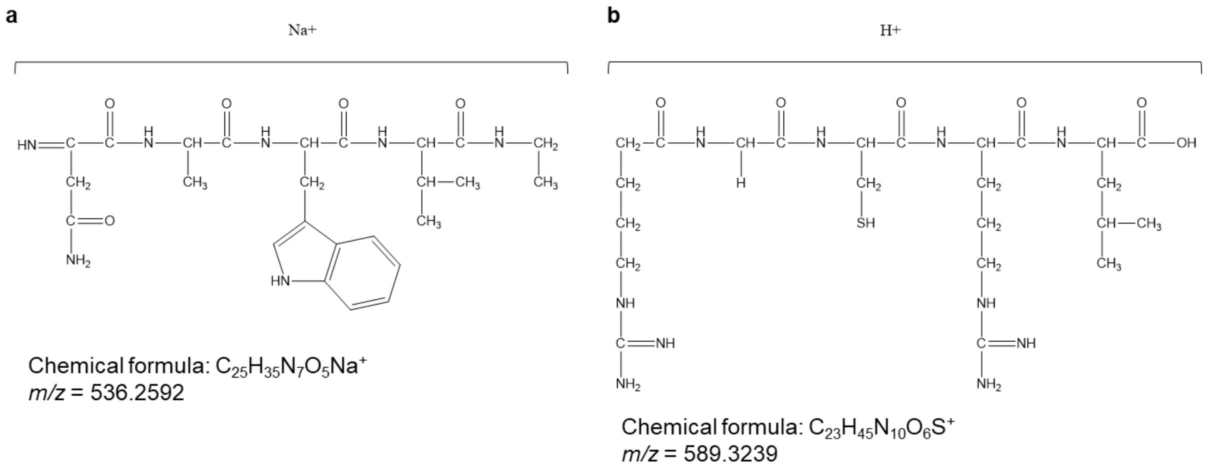

Supplementary Figure 5 Proposed structures of observed ions dissimilar to CID MS of tryptic peptides, similar to HCD or MALDI-ISD fragmentation. (a) Internal ion ya is presented on an example NAWVA sequence observed in the lysozyme spectrum. (b) C-terminal ion z+1 is presented on an example RGCRL sequence observed in the lysozyme spectrum. The elemental composition and theoretical *m/z* of the structure was generated in ChemDraw.

Supplementary Table 3 Examples of detected disulphide bonds in the 16 analysed proteins. The referenced Supplementary Tables provide full assignments of the relevant sequences.

Protein	Examples
insulin	Supplementary Tables 11 and 13
lipase	Supplementary Table 17
lysozyme	Supplementary Table 36
trypsin	Supplementary Tables 44 and 47
bovine serum albumin	Supplementary Tables 98 and 99
human serum albumin	Supplementary Table 111
fibronectin	Supplementary Table 146

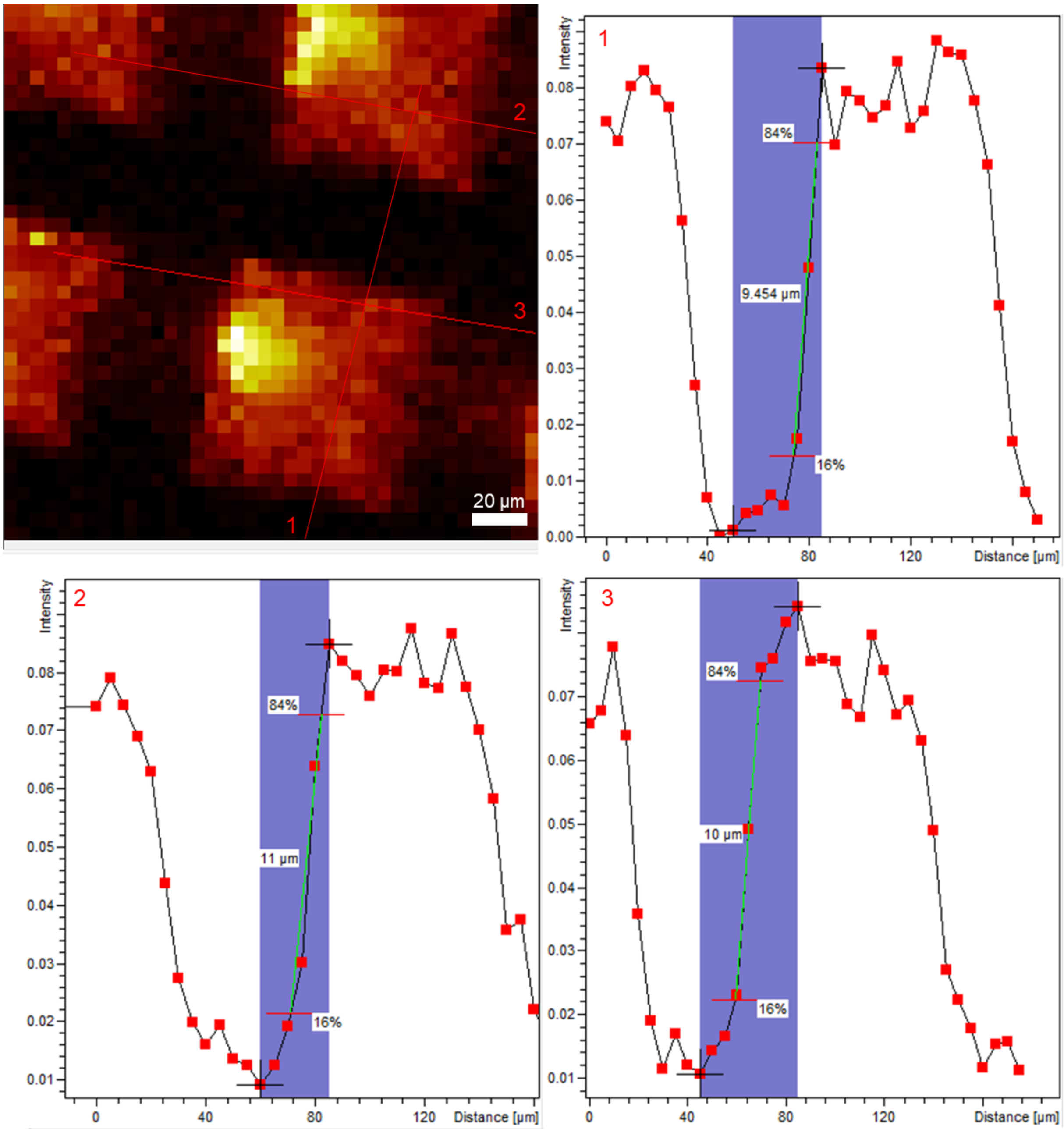

Supplementary Figure 6 3D OrbiSIMS image of a thin protein film (300 nm) under a transmission electron microscopy grid, acquired with total ion dose 1.11 × 10^{12} Lateral resolution of the GCIB Orbitrap image obtained with a focussed 5 μm diameter $Argon_{3000}^{+}$ primary beam. The image shows a sum of lysozyme peaks KVFG c, KVFG+Na c, KVFGR a5, KVFGR a5-NH3, KVFGR b5, KVFGR b5-NH3, KVFGR b-CN3H4, KVFGR c, KVFGRC a, KVFGRC b, KVFGRC-S a, KVFGRC-SH2 a, KVFGRCE a, KVFGRCE b, RL y, RL z+1, RL z-1, RG yb, IRG yb, WIRG yb, WIRG ya-NH3, AWIRG yb, AWIRGCRL z-1, VQAWIRG yc, DVQAWIRG yb, NAWV+Na yc, FNTQ+Na a, normalised to total ion count. Line scans 1, 2 and 3 demonstrate the ability to obtain a high chemical specificity image with lateral resolution of approximately 10 μm. Mean of three (n=3) measurements is 10.15 μm, standard deviation SD = 0.78 μm.

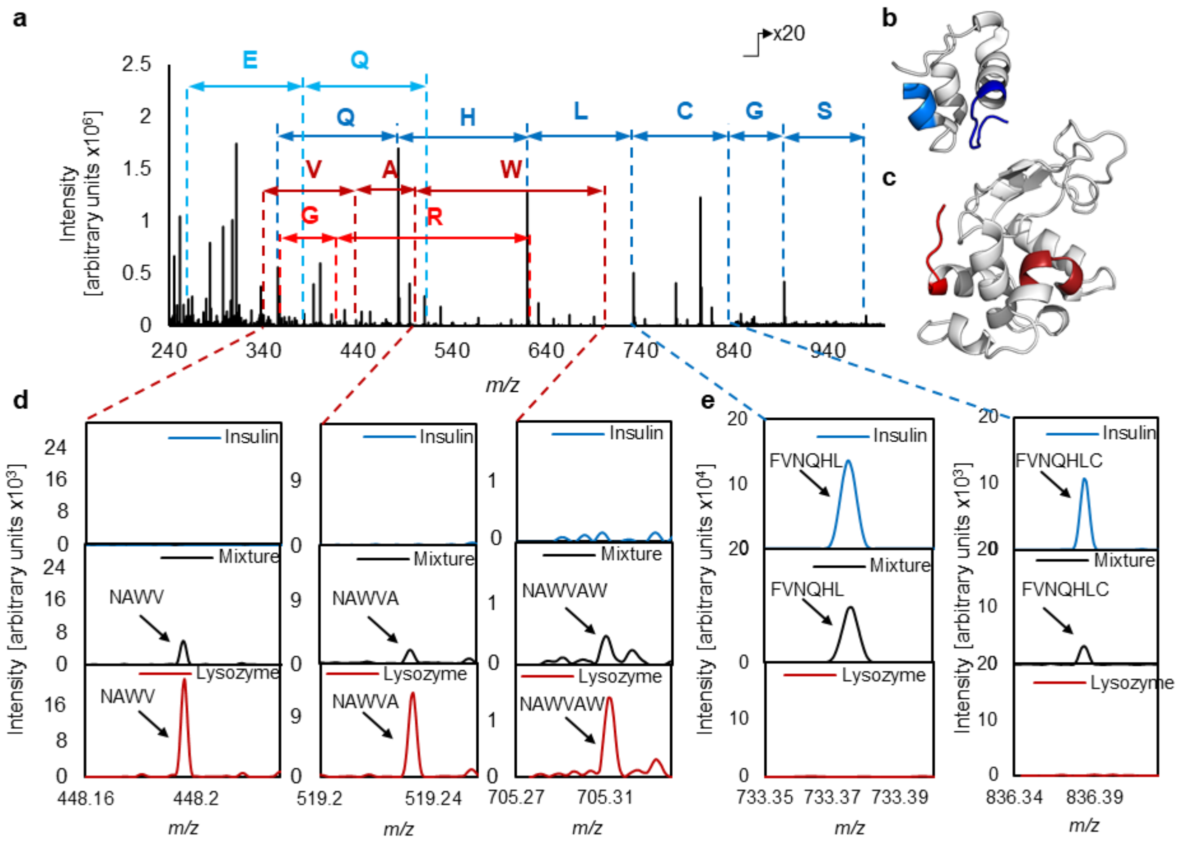

Supplementary Figure 7 Spectrum of a thin film (300 nm) of 1:1 lysozyme: insulin mixture. Segments of amino acids originating from insulin (light and dark blue) and lysozyme (light and dark red) are presented in the spectrum (a). Segments of insulin sequence observed in the spectrum are highlighted blue in the insulin cartoon (b). Segments of lysozyme sequence observed in the spectrum are highlighted red in the lysozyme cartoon (c). An additional overlay of insulin, mixture and lysozyme samples was magnified to demonstrate lysozyme (d) and insulin (e) related fragments. Peaks assigned as lysozyme sequence NAWVAW (dark red) are detected in the mixture spectrum (black) and are absent in the insulin spectrum (blue) (d). Peaks assigned as insulin sequence FVNQHLC (blue) are detected in the mixture spectrum (black) and are absent in the lysozyme spectrum (red) (e).

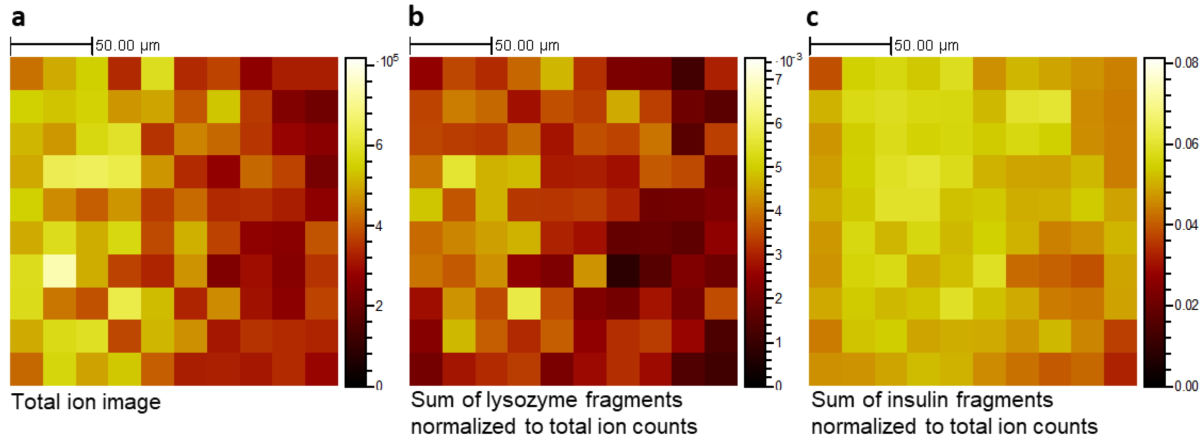

Supplementary Figure 8 GCIB Orbitrap images of the model mixture The maps represent the total ion image (a), sum of lysozyme fragments, normalized to total (b) and sum of insulin fragments, normalized to total (c). Fragment ions originating from both lysozyme (FN, FNT, FNTQ, FNTQA, NTQA, GI, GIL, GILQ, KVF, KVFG, KVFGR, KVFGRC, KVFGRCE,NA, NAW, NAWV, NAWVA, NAWVAWR, RG) (b) and insulin (FVN, FVNQ, FVNQH, FVNQHL, FVNQHLC, FVNQHLCG, FVNQHLCGS, FVNQHLCGSH, GI, GIV, GIVEQ, GIVEQC) (c) are distributed across the whole analysis area and are detected simultaneously from a mixture.

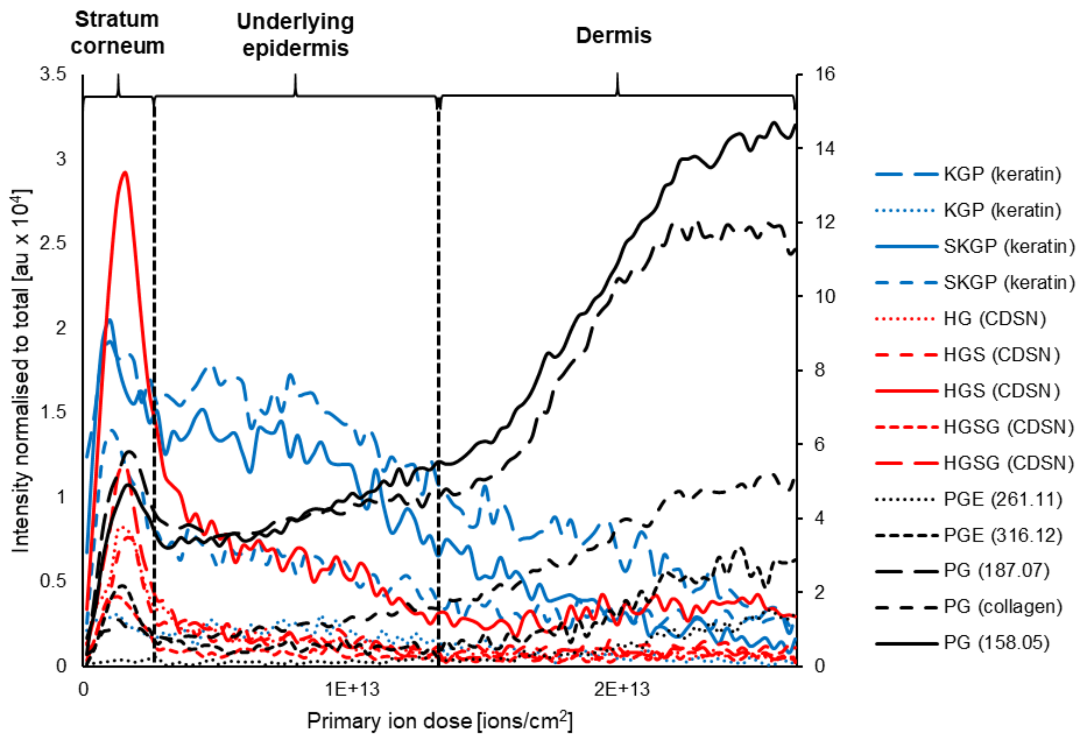

Supplementary Figure 9 3D OrbiSIMS depth profile overlay of example ions representing three major proteins in the skin. Corneodesmosin (red) is found mostly in stratum corneum, keratin (blue) is abundant throughout the epidermis and collagen (black) is most prevalent in the dermis. The dashed lines indicate borders between the skin layers, assigned based on the profile of phospholipid marker (PO_3^-).

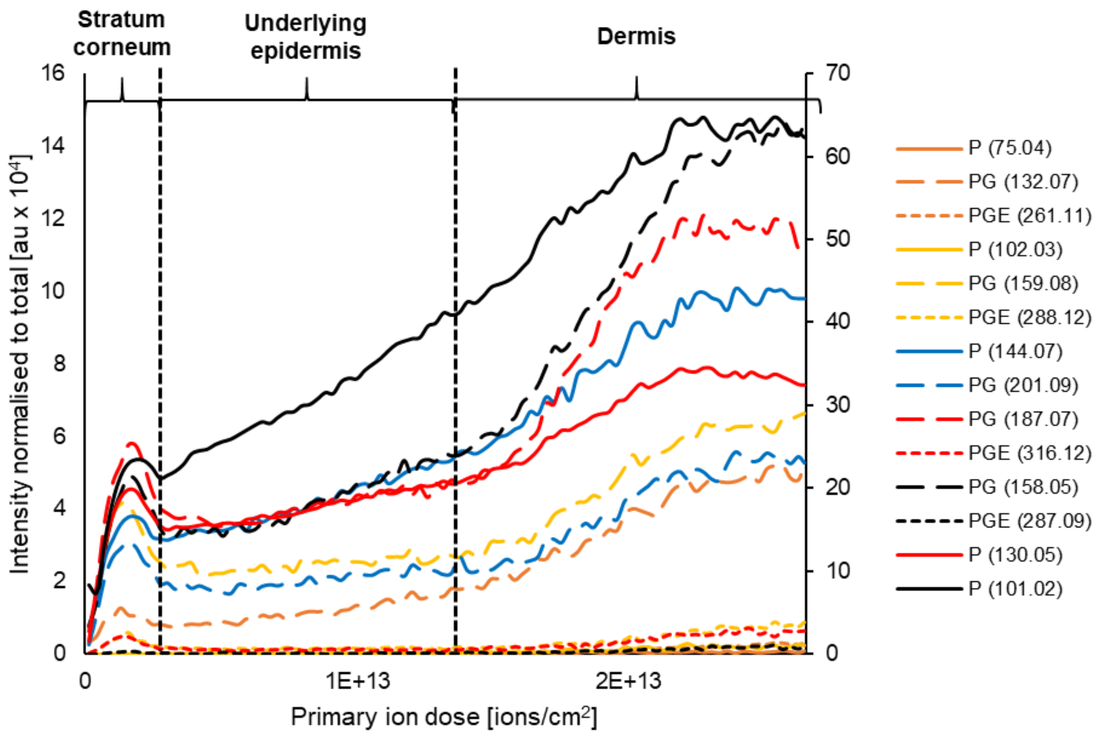

Supplementary Figure 10 3D OrbiSIMS depth profile overlay of example ions representing collagen. The sequence PGE, frequently occurring in the collagen sequence, is profiled through the skin with hydorxyproline (130.05 *m/z*), metoxyproline (144.07 *m/z*) or hydroxyproline fragments (75.04, 101.02, 102.03 *m/z*) as starting points. Each colour represents one sequence and all ions are assigned in Supplementary Table 4.

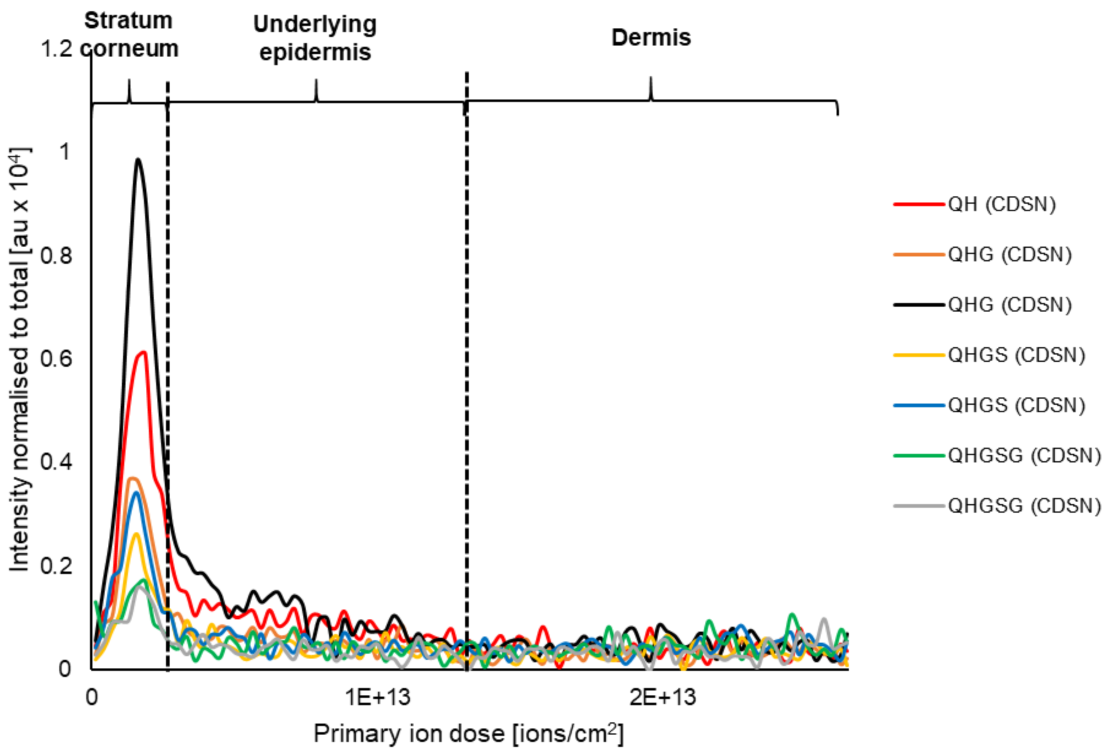

Supplementary Figure 11 3D OrbiSIMS depth profile overlay of example ions representing QHGSG sequence of cormenodesmosin (CDSN), abundant predominantly in the *stratum corneum*. All presented ions are assigned in Supplementary Table 5.

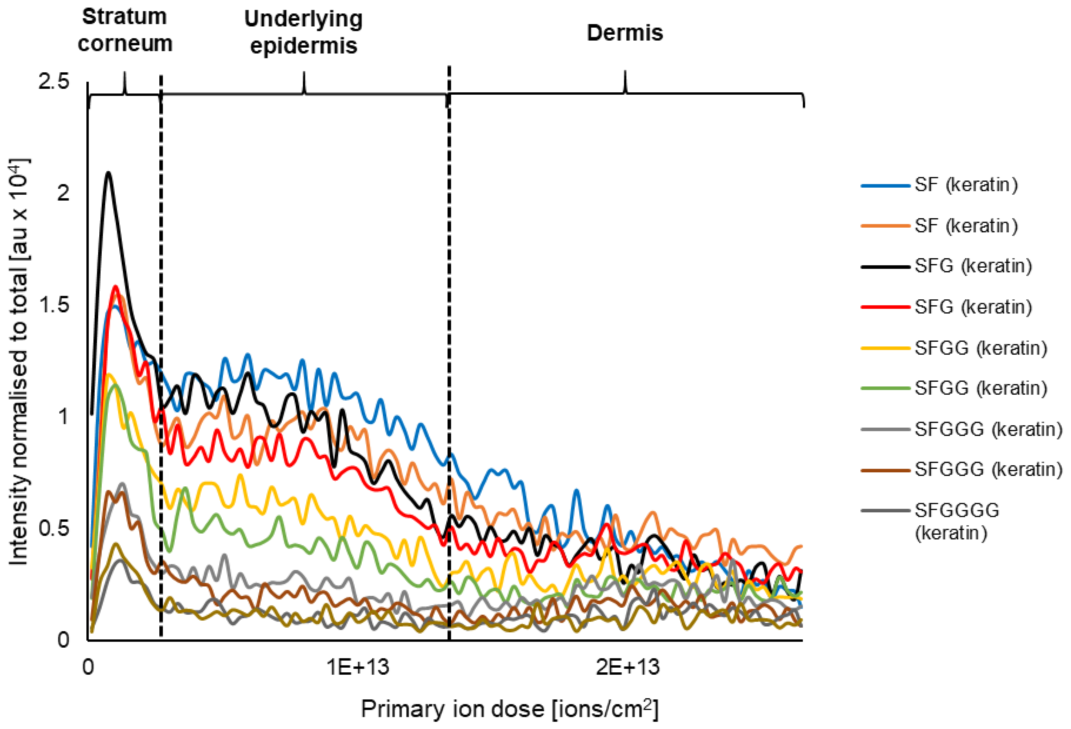

Supplementary Figure 12 3D OrbiSIMS depth profile overlay of example ions representing keratin sequence SFGGGG, detected throughout the stratum corneum and the underlying epidermis. All ions are assigned in Supplementary Table 6.

Supplementary Table 4 Peak list exported from SurfaceLab negative mode depth profile through human skin, consisting of ions detected in the spectrum and assigned as sequence PGE of collagen. The *m/z* values represent the experimentally observed center mass of each peak. The deviation (dev.) represents the parts per million (ppm) accuracy of the assignment. The colours represent the labelling in the depth distribution of the assigned peaks in Supplementary Figure 10.

	P			PG			PGE		
**Description**	***m/z***	**Assignment**	**Dev. (ppm)**	***m/z***	**Assignment**	**Dev. (ppm)**	***m/z***	**Assignment**	**Dev. (ppm)**
Metoxyproline	144.0665	$C_6H_{10}NO_3^-$	-1.0039	201.0880	$C_8H_{13}N_2O_4^-$	-0.3301	330.1306	$C_{13}H_{20}N_3O_7^-$	-0.1054
Hydroxyproline fragment	75.0448	$C_3H_7O_2^-$	-5.2346	132.0665	$C_5H_{10}NO_3^-$	-0.8348	261.1094	$C_{10}H_{17}N_2O_6^-$	0.8199
Hydroxyproline fragment	101.0243	$C_4H_5O_3^-$	-0.8169	158.0457	$C_6H_8NO_4^-$	-0.9927	287.0886	$C_{11}H_{15}N_2O_7^-$	0.4788
Hydroxyproline fragment	102.0322	$C_4H_6O_3^-$	-0.5861	159.0774	$C_6H_{11}N_2O_3^-$	-0.9402	288.1202	$C_{11}H_{18}N_3O_6^-$	0.4506
Hydroxyproline	130.0508	$C_5H_8NO_3^-$	-1.0131	187.0723	$C_7H_{11}N_2O_4^-$	-0.6061	316.1148	$C_{12}H_{18}N_3O_7^-$	-0.5758

Supplementary Table 5 Peak list exported from SurfaceLab negative mode depth profile through human skin, consisting of ions detected in the spectrum and assigned as sequence QHGSG of corneodesmosin. The *m/z* values represent the experimentally observed center mass of each peak. The deviation (dev.) represents the parts per million (ppm) accuracy of the assignment. The depth distribution of the assigned peaks is presented in Supplementary Figure 11.

	a			b			c			a-NH3		
**Description**	***m/z***	**Assignment**	**Dev. (ppm)**	***m/z***	**Assignment**	**Dev. (ppm)**	***m/z***	**Assignment**	**Dev. (ppm)**	***m/z***	**Assignment**	**Dev. (ppm)**
QH	236.1153	$C_{10}H_{14}N_5O_2^-$	0.0475	264.1104	$C_{11}H_{14}N_5O_3^-$	0.5526	281.1369	$C_{11}H_{17}N_6O_3^-$	0.4540	219.0886	$C_{10}H_{11}N_4O_2^-$	-0.5614
QHG	293.1370	$C_{12}H_{17}N_6O_3^-$	0.9411	321.1315	$C_{13}H_{17}N_6O_4^-$	-0.5284	338.1585	$C_{13}H_{20}N_7O_4^-$	0.8865	276.1102	$C_{12}H_{14}N_5O_3^-$	0.0404
QHGS	380.1689	$C_{15}H_{22}N_7O_5^-$	0.3898	408.1636	$C_{16}H_{22}N_7O_6^-$	-0.2528				363.1422	$C_{15}H_{19}N_6O_5^-$	-0.0194
QHGSG	437.1901	$C_{17}H_{25}N_8O_6^-$	-0.4396	465.1849	$C_{18}H_{25}N_8O_7^-$	-0.6714				420.1639	$C_{17}H_{22}N_7O_6^-$	0.3438

Supplementary Table 6 Peak list exported from SurfaceLab negative mode depth profile through human skin, consisting of ions detected in the spectrum and assigned as sequence SFGGGG of keratin. The *m/z* values represent the experimentally observed center mass of each peak. The deviation (dev.) represents the parts per million (ppm) accuracy of the assignment. The depth distribution of the assigned peaks is presented in Supplementary Figure 12.

	a			b			c			a-NH3		
**Description**	***m/z***	**Assignment**	**Dev. (ppm)**	***m/z***	**Assignment**	**Dev. (ppm)**	***m/z***	**Assignment**	**Dev. (ppm)**	***m/z***	**Assignment**	**Dev. (ppm)**
SF	207.1138	$C_{11}H_{15}N_2O_2^-$	-0.5626	235.1088	$C_{12}H_{15}N_2O_3^-$	-0.0630	252.1354	$C_{12}H_{18}N_3O_3^-$	0.3139	190.0873	$C_{11}H_{12}NO_2^-$	-0.4510
SFG	264.1355	$C_{13}H_{18}N_3O_3^-$	0.6372	292.1304	$C_{14}H_{18}N_3O_4^-$	0.5157	309.1568	$C_{14}H_{21}N_4O_4^-$	-0.0093	247.1088	$C_{13}H_{15}N_2O_3^-$	0.1317
SFGG	321.1566	$C_{15}H_{21}N_4O_4^-$	-0.6429	349.1515	$C_{16}H_{21}N_4O_5^-$	-0.6948	366.1780	$C_{16}H_{24}N_5O_5^-$	-0.7797	304.1302	$C_{15}H_{18}N_3O_4^-$	-0.1081
SFGGG	378.1781	$C_{17}H_{24}N_5O_5^-$	-0.5307	406.1728	$C_{18}H_{24}N_5O_6^-$	-0.8831	423.1997	$C_{18}H_{27}N_6O_6^-$	-0.0624	361.1517	$C_{17}H_{21}N_4O_5^-$	-0.2364
SFGGGG	435.1995	$C_{19}H_{27}N_6O_6^-$	-0.5562	463.1944	$C_{20}H_{27}N_6O_7^-$	-0.4827	480.2210	$C_{20}H_{30}N_7O_7^-$	-0.4193	418.1728	$C_{19}H_{24}N_5O_6^-$	-0.9422

Supplementary Note 1: Analysis of a protein biochip

A practical application of the developed method is demonstrated by detection of information-rich protein fragments on a protein biochip. The spectra of the biochip contain information from the first two scans of the surface, after which the signal rapidly declines, as the monolayer of the protein is removed from the surface. The sensitivity limits the detection of the complete sequences and the peaks that are most abundant in the reference sample are visible in the biochip sample. The thickness of the layer was analysed by ellipsometry and calculated from the X-ray photoelectron spectroscopy (Supplementary Figure 13).[3]

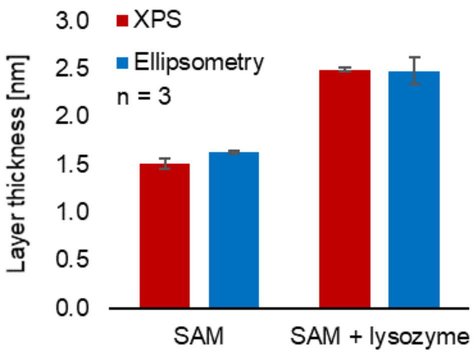

Supplementary Figure 13 Layer thickness measurements results of the self assembled monolayer (SAM) and SAM with immobilised lysozyme, derived from XPS results (red) and measured by ellipsometry (blue). The bars represent mean of three measurements on one sample. The error bars represent the standard deviation between three (n=3) different areas at the surface of one sample.

The amount of protein molecules in the analysed volume is calculated by Equation 1. A is the analysed area, 200 × 200 μm (40000 μm^2). A_{TCD} is the area of one molecule of thiol β-cyclodextrin (TCD) forming the self assembled monolayer. A_{TCD} is 1.83 nm^2, as the diameter of one TCD molecule is 1.53 nm.

$$n = \frac{A}{A_{TCD}} \qquad \text{Equation 1}$$

The actual amount of protein molecules is smaller than the amount of cyclodextrin molecules and is determined by the size of the protein. Therefore, the maximum amount of lysozyme molecules in the analysed area is 2.18 × 10^{10} (40 femtomoles). This amount of lysozyme allows detection of seven distinct lysozyme fragments (Supplementary Table 7).

Supplementary Table 7 Lysozyme fragments visible in the spectra obtained from a protein monolayer sample. Protein monolayer was obtained by immobilisation of the protein on a self assembled monolayer (SAM) on a gold slide. Three areas on a sample were analysed and a SAM sample without the protein was analysed as a control. The Supplementary Table presents average intensity (arbitrary units) of selected ions across the three measurements per sample.

No	*m/z*	Assignment	Description	SAM + lysozyme		SAM only		Bare gold	
				Sample 1	Sample 2	Sample 1	Sample 2	Sample 1	Sample 2
1	214.1295	$C_8H_{16}N_5O_2^+$	RG	46277.9	59307.63	217.33	0	0	0
2	271.1765	$C_{12}H_{23}N_4O_3^+$	RL	3421.62	1859.43	0	0	0	0
3	449.2864	$C_{22}H_{37}N_6O_4^+$	KVFG	2058.25	3253.01	0	24.75	0	0
4	560.3665	$C_{27}H_{46}N_9O_4^+$	KVFGR	652.65	1236.11	0	0	0	0
5	588.3612	$C_{28}H_{46}N_9O_5^+$	KVFGR	9752.09	6227.74	0	0	0	0
6	605.3875	$C_{28}H_{49}N_{10}O_5^+$	KVFGR	19300.05	14136.18	0	33.58	0	0
7	631.4030	$C_{30}H_{51}N_{10}O_5^+$	KVFGRC-S	6798.67	3267.04	0	0	0	0

Sensitivity and limit of detection of the peptidic fragments were also calculated for the lysozyme film. In reference lysozyme film samples, the amount of material removed during sputtering is assumed to be pure protein. The amount of protein molecules can be calculated by Equation 2, where ρ is the density of the protein (1.37 g/mL)[4], A is the analysed area, 200 × 200 μm (40000 μm^2), d is the depth of material consumed during the analysis, estimated by the SurfaceLab software and confirmed by profilometry (300 nm, Supplementary Table 8).

$$n = \frac{\rho A d N_A}{M_W} \qquad \text{Equation 2}$$

The profilometry results allow for calculation of the depth resolution of the instrument with the chosen settings. In reference protein samples, with 300 nm total depth after 30 scans, depth per one scan was 10 nm. This is consistent with SurfaceLab estimation based on the primary ion current. In cryogenic skin depth profile, depth was estimated based on the knowledge of the skin layers with stratum corneum making up approximately outermost 20 μm of the skin.[5] Based on the profile of phosphate marker PO_3^-, underlying epidermis was reached after 1500 scans (Supplementary Figure 9), therefore the depth resolution calculated is 13 nm per scan.

The amount of lysozyme molecules that enabled the protein fragment assignment in the analysed sample was 6.8742 × 10^{11} (1 picomole). The amount of lysozyme analysed from the biochip

monolayer sample (40 femtomoles) is sufficient for the detection of seven diagnostic peaks, however does not enable direct primary structure analysis of this protein.

Supplementary Table 8 Crater depth measured by optical profilometry. The average of three measurements on one sample (one measurement per crater) is 304 nm. The accuracy (step size) of the profilometer is 14 nm. The standard deviation (SD) of three measueremnts is within the instrument accuracy limit.

	**Cursor Left Avg Ht (µm)**	**Cursor Right Avg Ht (µm)**	**Cursor L-R Step (µm)**
1	0.3025	0.0025	-0.3000
2	0.2976	0.0026	-0.2951
3	0.3398	0.0236	-0.3162
Average	0.3133	0.0096	-0.3038
SD	0.0231	0.0122	0.0110

Supplementary Note 2: Analysis of protein assignment from protein structural database

Analysis of the UniProt [https://www.UniProt.org] protein sequences (28th July 2019) and function database was undertaken to determine the degree to which proteins can be identified from sequences deduced from their mass spectrum. For each protein sequence of length n in the database of 21417, all n-l sequences of length l, from 2 to 20 residues, were searched for, with isoleucine substituted for leucine, as they have identical mass. In this analysis first 3 residues were considered by us to be of unknown in composition, because experimentally they could not be assigned through sequencing. The composition of the initial tripeptide can be assigned using the MS/MS capability of the 3D OrbiSIMS instrument. The number of proteins that contained a match for each of its n-l fragment sequences was counted. Supplementary Figure 14 shows the number of proteins that can be uniquely identified from its sequence from residue 4 to residue 3+l. The total number of proteins which shared at least one sequence with that tested is shown in Supplementary Figure 15a. The fewest number of proteins that shared a sequence with the test protein is shown in Supplementary Figure 15b. 89% of the proteins can be identified only by a known N-terminal sequence of 8 amino acids (Supplementary Figure 14). The capability to readily identify an unknown protein from a N-terminal sequence is limited due to the presence of mid-sequence ions in the spectrum, however the described method provides information about amino acid sequences of sufficient length to enable assignment of 89% of human proteins provided the protein spectra databases are suitably adapted or devised. The method as it is, allows for identification of between 10% (Supplementary Figure 15a) and 89% (Supplementary Figure 15b) proteins from a 8-residue sequence found, depending on the composition of the observed sequence.

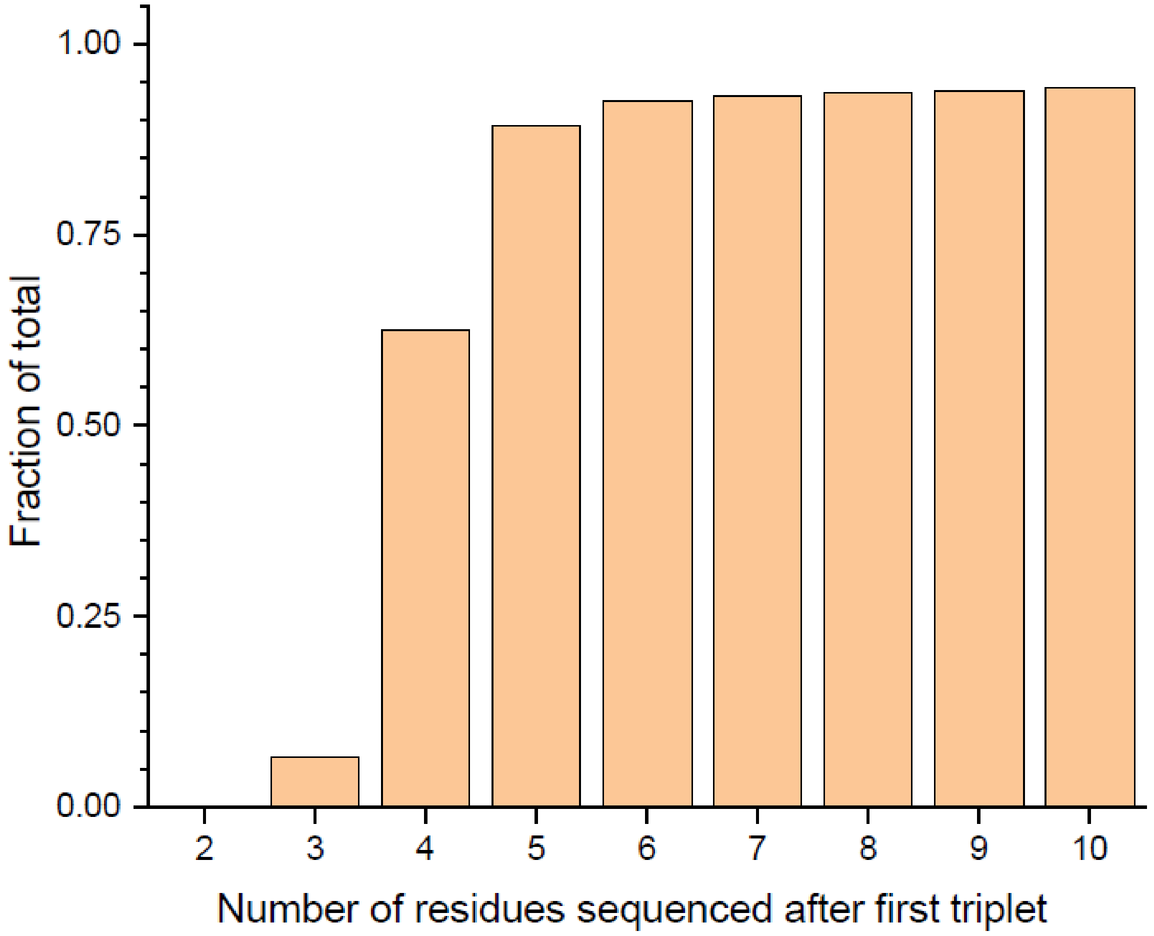

Supplementary Figure 14 Statistical analysis of the fraction of the proteome that can be identified by N-terminal sequences. 89% of the human proteins in the UniProt sequence database (28th July 2019) can be confidently identified if the smallest fragment is a tripeptide and is followed by five amino acid residues.

a

b

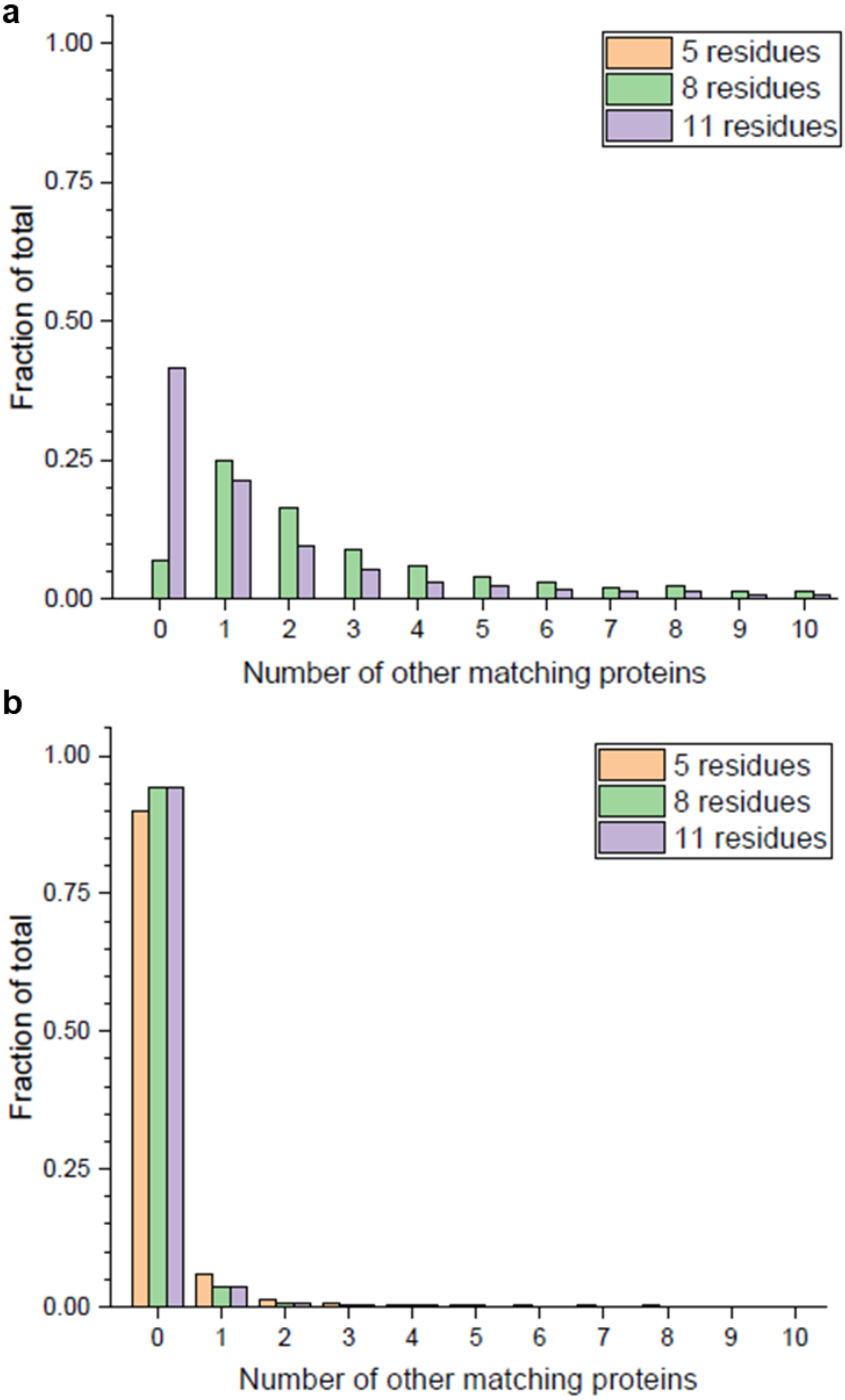

Supplementary Figure 15 (a) Analysis of human proteins in the UniProt sequence database (28th July 2019) that share 5, 8 or 11-residue long sequences with any other. Depending on the composition of the sequence found, the sequence may be diagnostic or common to many proteins. (a) 93% of the proteins share an 8-residue sequence with at least one other protein. (b) 95% of the proteins, however, contain an 8-residue sequence that is unique to the particular protein. 89% of the proteins contain a 5-residue unique sequence.

Supplementary Note 3: Automated sequence search

Conventional software for protein identification, including programmes focusing on *de novo* sequencing such as PEAKS, Novor, PepNovo, MSNovo, UniNovo and others[6], are not appropriate for the data produced by the combination of GCIB and Orbitrap™ due to different mode of fragmentation. These tools also do not allow for identification of a protein from an image. In order to enable automatic high-throughput identification of intact proteins directly from a surface, a *de novo* sequencing script was developed in MATLAB (Figure 3) and the code is available online (https://github.com/guferraz/simsdenovo/). The input to the script is a peak list exported from data analysis software, here IONTOF SurfaceLab 7.1 as a text file containing peak *m/z* and intensity values (Figure 3b). **Chemical filtering** is based on matching the masses of the input peak list to the exact masses of possible peptidic fragments of each ion type (all combinations of up to 10-membered a, b, c and a-NH_3). **Intensity filtering** is done on transformed values of Intensity × mass (I × m) to retain the high mass information-rich. Filtering allowed to shorten 2450 peak-long lists to 250 peak-long lists. **Residues identification** is done by calculating a matrix of differences between all pairs of masses in the filtered peak list and checking for matches to amino acid residues within a given tolerance in ppm. All differences without a match are discarded from the matrix. As a result, each residue has a "beginning" (matrix rows) and "end" ion (matrix columns), the distance between which is unique to the given residue. In Figure 3d, each residue is given a different colour. **Sequences are searched** by sequentially connecting the "end" ion of a given residue to the "beginning" ion of the next residue (Figure 3d inset). Sequences are elongated until no further residue can be connected and the search is done recursively using all found residues as seed points. 3 to 8 membered sequences are retained and ordered from longest to shortest. **Protein identification** is done by comparing found and rated sequences to the UniProt database for a given taxonomy. Ranking is done iteratively starting from the longest sequence: i. find all the database protein sequences where the sequence matches a terminal fragment of a protein (first or last 15 members). ii. for each protein found, count the number of times any of them appear. iii. the next longest sequence is then searched for (back to i) and continued until all the sequences have been used. The full list of proteins is ranked from highest to lowest number of counts.

The script was tested against the 16 analysed proteins. The pre-processing filtering parameters have to be set individually per each sample due to different spectra qualities and a diagnostic tool was developed for this purpose (Supplementary Figure 16). For each protein, peak lists generated by applying 13 chemical filters to the original peak list, were subsequently filtered by I × m varying with equal increments in a logarithmic space between 10^5 and 10^8 (Supplementary Figure 16).

The script unambiguously identified 6 proteins: insulin (6 kDa), chymotrypsin (25 kDa), concanavalin (31 kDa), alcohol dehydrogenase (36 kDa), and fibronectin (272 kDa), with 3 others being listed in the top 15 possible matches: lipase (53 kDa), cytochrome c (12 kDa) and lysozyme (14 kDa) (Supplementary Figure 16, Supplementary Table 9).

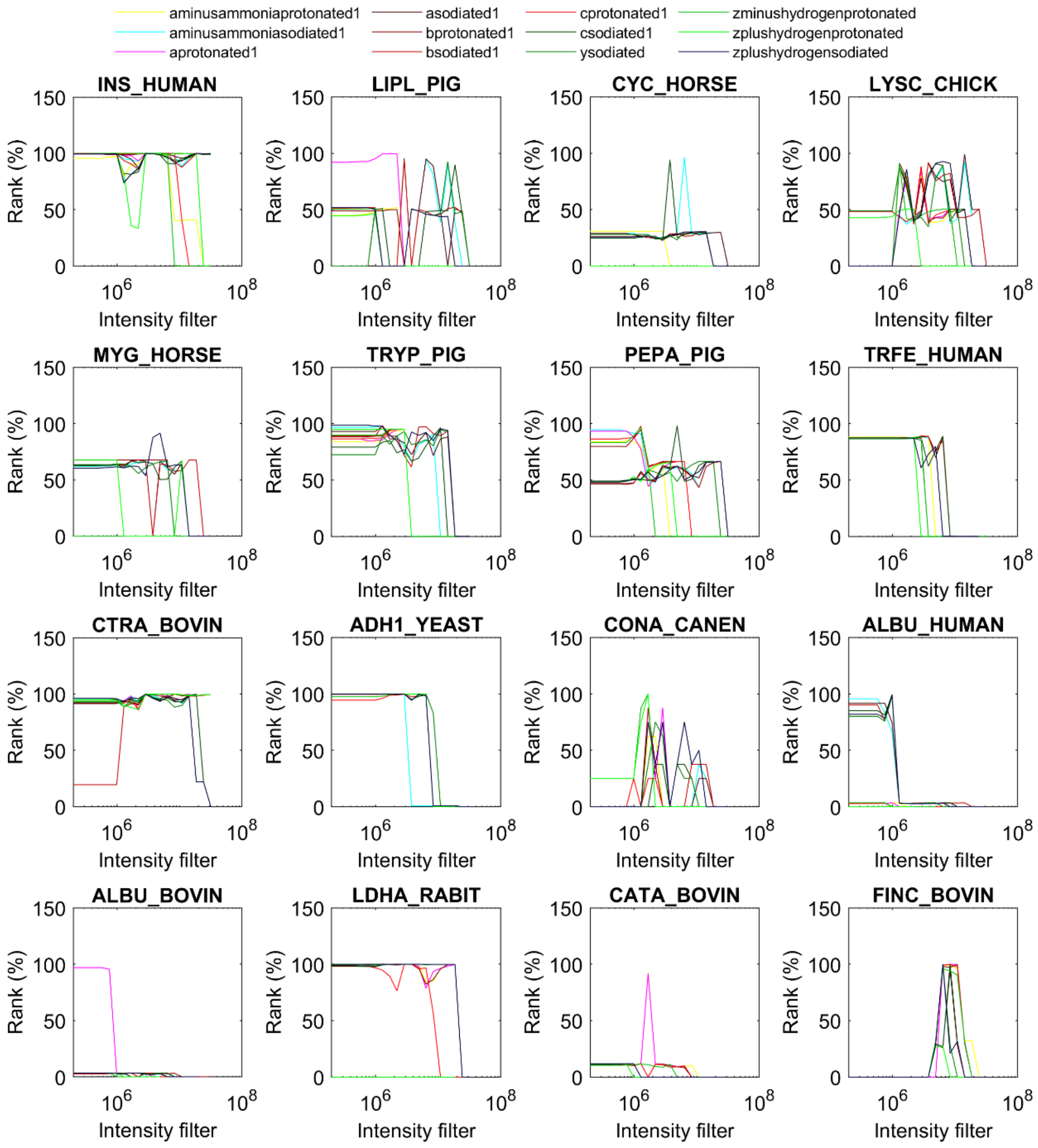

Supplementary Figure 16: Diagnostics of the optimal chemical and intensity filters for identification of proteins from unknown spectra. Chemically filtered peak lists for each protein were subsequently filtered by intensity. The rank shows the relative position of the detected protein against all proteins in the database. Detailed rank for each protein is listed in Supplementary Table 9.

Supplementary Table 9: Best diagnostics results for each protein. Each protein is given a rank number among all proteins in a given taxonomy. The rank is also given as a percent confidence in confirmation of the identity. Most suiSupplementary Table chemical filters demonstrate the type of ion (a, b, c, a-NH3, z+1 or z-1) with the longest sequences detected for each protein. The edge of the intensity filter describes the value of the intensity threshold, which results with the best rank for a given protein.

Protein	Best rank	Rank %	Best chemical filter	Intensity filter edge
INS_HUMAN	1 out of 21451	100.00	bprotonated1	4.89E+06
LIPL_PIG	4 out of 1671	99.82	aprotonated1	1.68E+06
CYC_HORSE	13 out of 318	96.23	aminusammoniasodiated1	6.39E+06
LYSC_CHICK	18 out of 2392	99.29	asodiated1	1.42E+07
MYG_HORSE	28 out of 318	91.51	zplushydrogensodiated	4.89E+06
TRYP_PIG	22 out of 1671	98.74	zplushydrogensodiated	5.80E+05
PEPA_PIG	32 out of 1671	98.14	csodiated1	4.89E+06
TRFE_HUMAN	2326 out of 21451	89.16	aminusammoniaprotonated1	3.75E+06
CTRA_BOVIN	1 out of 6414	100.00	cprotonated1	8.34E+06
ADH1_YEAST	1 out of 7043	100.00	aprotonated1	1.68E+06
CONA_CANEN	1 out of 8	100.00	zminushydrogenprotonated	1.68E+06
ALBU_HUMAN	126 out of 21451	99.42	csodiated1	9.88E+05
ALBU_BOVIN	196 out of 6414	96.96	aprotonated1	5.80E+05
LDHA_RABIT	1 out of 959	100.00	aminusammoniaprotonated1	3.75E+06
CATA_BOVIN	483 out of 6414	92.49	aminusammoniaprotonated1	1.68E+06
FINC_BOVIN	1 out of 6414	100.00	bsodiated1	8.34E+06

Insulin

a)

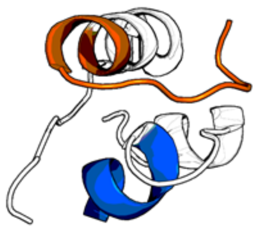

b)

Uniprot database code	Amino acid sequence
INS_HUMAN1n	FVNQHLCGSHLVEALYLVCGERGFFYTPKT
INS_HUMAN2n	GIVEQCCTSICSLYQLENYCN

Supplementary Figure 17 Human insulin (a) cartoon exported from PBD structure 3I40[7] and (b) amino acid sequence exported from the UniProt database. The highlighted colours correspond to assigned segments of the amino acid sequence, presented in Supplementary Tables 10-13.

Supplementary Table 10 Peak list exported from SurfaceLab spectrum of human insulin, consisting of ions detected in the positive polarity spectrum and assigned as sodium adducts of N-terminal sequence FVNQHLCGSHLV of human insulin. The sequence is observed as a, b, c and a-NH_3 ions. The *m/z* values represent the experimentally observed center mass of each peak. The deviation (dev.) represents the parts per million (ppm) accuracy of the assignment. The colour corresponds to the presence of the observed sequence in human insulin presented in Supplementary Figure 17.

	**a**			**b**			**c**			**a-NH_3**		
**Description**	***m/z***	**Assignment**	**Dev. (ppm)**	***m/z***	**Assignment**	**Dev. (ppm)**	***m/z***	**Assignment**	**Dev. (ppm)**	***m/z***	**Assignment**	**Dev. (ppm)**
FVN	355.1738	$C_{17}H_{24}N_4O_3Na^+$	-0.6305	383.1688	$C_{18}H_{24}N_4O_4Na^+$	-0.5894	400.1953	$C_{18}H_{27}N_5O_4Na^+$	-0.5872	338.1472	$C_{17}H_{21}N_3O_3Na^+$	-0.7458
FVNQ	483.2324	$C_{22}H_{32}N_6O_5Na^+$	-0.4139	511.2273	$C_{23}H_{32}N_6O_6Na^+$	-0.5153	528.2539	$C_{23}H_{35}N_7O_6Na^+$	-0.4489	466.2058	$C_{22}H_{29}N_5O_5Na^+$	-0.6667
FVNQH	620.2912	$C_{28}H_{39}N_9O_6Na^+$	-0.4746	648.2861	$C_{29}H_{39}N_9O_7Na^+$	-0.5111	665.3127	$C_{29}H_{42}N_{10}O_7Na^+$	-0.4668	603.2648	$C_{28}H_{36}N_8O_6Na^+$	-0.4038
FVNQHL	733.3753	$C_{34}H_{50}N_{10}O_7Na^+$	-0.3553	761.3701	$C_{35}H_{50}N_{10}O_8Na^+$	-0.5003	778.3967	$C_{35}H_{53}N_{11}O_8Na^+$	-0.4391	716.3489	$C_{34}H_{47}N_9O_7Na^+$	-0.1565
FVNQHLC	836.3844	$C_{37}H_{55}N_{11}O_8SNa^+$	-0.4028	864.3798	$C_{38}H_{55}N_{11}O_9SNa^+$	0.0746	881.4056	$C_{38}H_{58}N_{12}O_9SNa^+$	-0.7268	x	x	x
FVNQHLCG	893.4058	$C_{39}H_{58}N_{12}O_9SNa^+$	-0.4869	921.4013	$C_{40}H_{58}N_{12}O_{10}SNa^+$	0.1357	938.4272	$C_{40}H_{61}N_{13}O_{10}SNa^+$	-0.5241	x	x	x
FVNQHLCGS	980.4378	$C_{42}H_{63}N_{13}O_{11}SNa^+$	-0.4731	x	x	x	1,025.4595	$C_{43}H_{66}N_{14}O_{12}SNa^+$	-0.2207	x	x	x
FVNQHLCGSH	1,117.4965	$C_{48}H_{70}N_{16}O_{12}SNa^+$	-0.6497	x	x	x	1,162.5181	$C_{49}H_{73}N_{17}O_{13}SNa^+$	-0.4593	x	x	x
FVNQHLCGSHL	1,230.5793	$C_{54}H_{81}N_{17}O_{13}SNa^+$	-1.6029	x	x	x	1,275.5999	$C_{55}H_{84}N_{18}O_{14}SNa^+$	-2.1904	x	x	x
FVNQHLCGSHLV	1,329.6479	$C_{59}H_{90}N_{18}O_{14}SNa^+$	-1.3351	x	x	x	1,374.6705	$C_{60}H_{93}N_{19}O_{15}SNa^+$	-0.4986	x	x	x

Supplementary Table 11 Peak list exported from SurfaceLab spectrum of human insulin, consisting of ions detected in the spectrum and assigned as N-terminal sequence FVNQHLCGSH of human insulin, with sulphur (black letter S) and SH_2 removed from the structure. The sequence is observed as a, b and c ions. The *m/z* values represent the experimentally observed center mass. The deviation (dev.) represents the parts per million (ppm) accuracy of the assignment. The colour corresponds to the presence of the observed sequence in human insulin presented in Supplementary Figure 17.

	a			b			c		
**Description**	***m/z***	**Assignment**	**Dev. (ppm)**	***m/z***	**Assignment**	**Dev. (ppm)**	***m/z***	**Assignment**	**Dev. (ppm)**
FVNQHLC-S	804.4124	$C_{37}H_{55}N_{11}O_8Na^+$	-0.3555	832.4074	$C_{38}H_{55}N_{11}O_9Na^+$	-0.24362	849.4339	$C_{38}H_{58}N_{12}O_9Na^+$	-0.3822
FVNQHLC-SH_2	802.3967	$C_{37}H_{53}N_{11}O_8Na^+$	-0.4309	830.3914	$C_{38}H_{53}N_{11}O_9Na^+$	-0.66818	847.4182	$C_{38}H_{56}N_{12}O_9Na^+$	-0.4367
FVNQHLCG-S	861.4338	$C_{39}H_{58}N_{12}O_9Na^+$	-0.4498	x	x	x	906.4555	$C_{40}H_{61}N_{13}O_{10}Na^+$	-0.1694
FVNQHLCG-SH_2	859.4181	$C_{39}H_{56}N_{12}O_9Na^+$	-0.5272	x	x	x	904.4393	$C_{40}H_{59}N_{13}O_{10}Na^+$	-0.7691
FVNQHLCGS-S	948.4657	$C_{42}H_{63}N_{13}O_{11}Na^+$	-0.5271	x	x	x	993.4865	$C_{43}H_{66}N_{14}O_{12}Na^+$	-1.1499
FVNQHLCGS-SH_2	946.4503	$C_{42}H_{61}N_{13}O_{11}Na^+$	-0.3125	x	x	x	991.4713	$C_{43}H_{64}N_{14}O_{12}Na^+$	-0.7005
FVNQHLCGSH-S	1,085.5246	$C_{48}H_{70}N_{16}O_{12}Na^+$	-0.4644	x	x	x	x	x	x
FVNQHLCGSH-SH_2	1,083.5094	$C_{48}H_{68}N_{16}O_{12}Na^+$	-0.0919	x	x	x	x	x	x

Supplementary Table 12 Peak list exported from SurfaceLab spectrum of human insulin, consisting of ions detected in the spectrum and assigned as sodium adducts of N-terminal sequence GIVEQC of the B chain of human insulin. The sequence is observed as a, b and c ions. The *m/z* values represent the experimentally observed center mass of each peak. The deviation (dev.) represents the parts per million (ppm) accuracy of the assignment. The colour corresponds to the presence of the observed sequence in human insulin presented in Supplementary Figure 17.

	a			b			c		
**Description**	***m/z***	**Assignment**	**Dev. (ppm)**	***m/z***	**Assignment**	**Dev. (ppm)**	***m/z***	**Assignment**	**Dev. (ppm)**
GI	165.0994	$C_7H_{14}N_2ONa^+$	-2.3491	193.0945	$C_8H_{14}N_2O_2Na^+$	-1.2589	210.1211	$C_8H_{17}N_3O_2Na^+$	-0.7347
GIV	264.1681	$C_{12}H_{23}N_3O_2Na^+$	-0.5409	292.1630	$C_{13}H_{23}N_3O_3Na^+$	-0.4912	309.1895	$C_{13}H_{26}N_4O_3Na^+$	-0.6666
GIVE	393.2106	$C_{17}H_{30}N_4O_5Na^+$	-0.7227	421.2055	$C_{18}H_{30}N_4O_6Na^+$	-0.5668	438.2321	$C_{18}H_{33}N_5O_6Na^+$	-0.5479
GIVEQ	521.2692	$C_{22}H_{38}N_6O_7Na^+$	-0.5115	549.2642	$C_{23}H_{38}N_6O_8Na^+$	-0.2508	x	x	x
GIVEQC	624.2783	$C_{25}H_{43}N_7O_8SNa^+$	-0.5621	x	x	x	x	x	x

Supplementary Table 13 Peak list exported from SurfaceLab spectrum of human insulin, consisting of ions detected in the spectrum and assigned as N-terminal sequence GIVEQC of the B chain of human insulin, with sulphur (black letter S) and SH_2 removed from the structure. The *m/z* values represent the experimentally observed center mass of each peak. The deviation (dev.) represents the parts per million (ppm) accuracy of the assignment. The colour corresponds to the presence of the observed sequence in human insulin presented in Supplementary Figure 17.

	a			b			c		
**Description**	***m/z***	**Assignment**	**Dev. (ppm)**	***m/z***	**Assignment**	**Dev. (ppm)**	***m/z***	**Assignment**	**Dev. (ppm)**
GIVEQC-S	592.3063	$C_{25}H_{43}N_7O_8Na^+$	-0.4222	x	x	x	x	x	x
GIVEQC-SH_2	590.2913	$C_{25}H_{41}N_7O_8Na^+$	0.6227	x	x	x	x	x	x

Lipase

The highlighted colours correspond to assigned segments of the amino acid sequence of pig lipase (LIPL_PIG1n) presented in Supplementary Tables 14-22.

ADRISGGRDFTDIESKFALRTPEDTVEDTCHLIPGVTESVANCHFNHSSKTFVVIHGWTVTGMYESWVPKLVAALYKREPDSNVIVVDWLSRAQQH
YPISAGYTKLVGQDVATFIDWMAVEFSYPPNNVHLLGYSLGAHAAGIAGSLTKKKVNRITGLDPAGPNFEYAEAPSRLSPDDADFVDVLHTFTRGS
PGRSIGIQKPVGHVDIYPNGGTFQPGCNIGEAIRVIAERGLGDVDQLVKCSHERSIHLFIDSLLNEENPSKAYRCNSKEAFEKGLCLSCRKNRCNNLG
YEINKVRAKRSSKMYLKTRAQMPYKVFHYQVKMRFSGTESDTHTNQAFEISLYGTVAESENIPFTLPEVSTNKTYSFLIYTEVDIGELLMLKLKWVS
DSYFSWSNWWSSPGFAIEKIRVKAGETQKKVIFCSREKKSHLQKGKSSVVFVKCHDKSLNRKSG

Supplementary Table 14 Peak list exported from SurfaceLab spectrum of pig lipase, consisting of ions detected in the spectrum and assigned as fragments of N-terminal sequence ADRI. The *m/z* values represent the experimentally observed center mass of each peak. The deviation (dev.) represents the parts per million (ppm) accuracy of the assignment.

	a			b			c			a-NH3		
**Description**	***m/z***	**Assignment**	**Dev. (ppm)**	***m/z***	**Assignment**	**Dev. (ppm)**	***m/z***	**Assignment**	**Dev. (ppm)**	***m/z***	**Assignment**	**Dev. (ppm)**
AD	181.0583	$C_6H_{10}N_2O_3Na^+$	-0.5734	209.0535	$C_7H_{10}N_2O_4Na^+$	1.2097	226.0800	$C_7H_{13}N_3O_4Na^+$	0.7329			
ADRI	450.2438	$C_{18}H_{33}N_7O_5Na^+$	0.4802							433.2173	$C_{18}H_{30}N_6O_5Na^+$	0.8294

Supplementary Table 15 Peak list exported from SurfaceLab spectrum of pig lipase, consisting of ions detected in the spectrum and assigned as internal fragment of the amino acid sequence GHVDIY. The *m/z* values represent the experimentally observed center mass of each peak. The deviation (dev.) represents the parts per million (ppm) accuracy of the assignment.

	a			b			b-NH3			a-NH3		
**Description**	***m/z***	**Assignment**	**Dev. (ppm)**	***m/z***	**Assignment**	**Dev. (ppm)**	***m/z***	**Assignment**	**Dev. (ppm)**	***m/z***	**Assignment**	**Dev. (ppm)**
GH				219.0854	$C_8H_{12}N_4O_2Na^+$	0.6074	202.0585	$C_8H_9N_3O_2Na^+$	-0.7734	174.0638	$C_7H_9N_3ONa^+$	0.0643
GHV				316.1385	$C_{13}H_{19}N_5O_3Na^+$	1.4603						
GHVD	405.1860	$C_{16}H_{26}N_6O_5Na^+$	0.8848									
GHVDI	518.2702	$C_{22}H_{37}N_7O_6Na^+$	0.8497									
GHVDIY	681.3333	$C_{31}H_{46}N_8O_8Na^+$	0.2643									

Supplementary Table 16 Peak list exported from SurfaceLab spectrum of pig lipase, consisting of ions detected in the spectrum and assigned as internal fragment of the amino acid sequence PKLVAA. The *m/z* values represent the experimentally observed center mass of each peak. The deviation (dev.) represents the parts per million (ppm) accuracy of the assignment.

	a			b			b-NH3			a-NH3		
**Description**	***m/z***	**Assignment**	**Dev. (ppm)**	***m/z***	**Assignment**	**Dev. (ppm)**	***m/z***	**Assignment**	**Dev. (ppm)**	***m/z***	**Assignment**	**Dev. (ppm)**
PK	222.1579	$C_{10}H_{21}N_3ONa^+$	0.7615	250.1528	$C_{11}H_{21}N_3O_2Na^+$	0.7821	233.1262	$C_{11}H_{18}N_2O_2Na^+$	0.7978	205.1312	$C_{10}H_{18}N_2ONa^+$	0.2630
PKL	335.2421	$C_{16}H_{32}N_4O_2Na^+$	1.0119	363.2370	$C_{17}H_{32}N_4O_3Na^+$	0.8974	346.2104	$C_{17}H_{29}N_3O_3Na^+$	0.8869	318.2155	$C_{16}H_{29}N_3O_2Na^+$	1.0273
PKLV	434.3109	$C_{21}H_{41}N_5O_3Na^+$	1.7186	462.3055	$C_{22}H_{41}N_5O_4Na^+$	0.8189	445.2789	$C_{22}H_{38}N_4O_4Na^+$	0.7356	417.2838	$C_{21}H_{38}N_4O_3Na^+$	0.4543
PKLVA				533.3425	$C_{25}H_{46}N_6O_5Na^+$	0.6494	516.3162	$C_{25}H_{43}N_5O_5Na^+$	0.9905	488.3213	$C_{24}H_{43}N_5O_4Na^+$	1.1856
PKLVAA										559.3574	$C_{27}H_{48}N_6O_5Na^+$	-0.6998

Supplementary Table 17 Peak list exported from SurfaceLab spectrum of pig lipase, consisting of ions detected in the spectrum and assigned as internal fragment of the amino acid sequence TFQPGC. The sequence is observed as a ions. The *m/z* values represent the experimentally observed center mass of each peak. The deviation (dev.) represents the parts per million (ppm) accuracy of the assignment.

	a		
**Description**	***m/z***	**Assignment**	**Dev. (ppm)**
TF	245.1262	$C_{12}H_{18}N_2O_2Na^+$	0.7168
TFQ	373.1849	$C_{17}H_{26}N_4O_4Na^+$	0.8085
TFQP	470.2377	$C_{22}H_{33}N_5O_5Na^+$	0.5704
TFQPG	527.2592	$C_{24}H_{36}N_6O_6Na^+$	0.7229
TFQPGC -S	598.2969	$C_{27}H_{41}N_7O_7Na^+$	1.5801
TFQPGC -SH_2	596.2810	$C_{27}H_{39}N_7O_7Na^+$	1.2166

Supplementary Table 18 Peak list exported from SurfaceLab spectrum of pig lipase, consisting of ions detected in the spectrum and assigned as internal fragment of the amino acid sequence GYSLGAH. The *m/z* values represent the experimentally observed center mass of each peak. The deviation (dev.) represents the parts per million (ppm) accuracy of the assignment.

	a			b			c			a-NH3		
**Description**	***m/z***	**Assignment**	**Dev. (ppm)**	***m/z***	**Assignment**	**Dev. (ppm)**	***m/z***	**Assignment**	**Dev. (ppm)**	***m/z***	**Assignment**	**Dev. (ppm)**
GYS	304.1272	$C_{13}H_{19}N_3O_4Na^+$	1.2963									
GYSL	417.2112	$C_{19}H_{30}N_4O_5Na^+$	0.8114	445.2051	$C_{20}H_{30}N_4O_6Na^+$	-1.4727	462.2325	$C_{20}H_{33}N_5O_6Na^+$	0.5033	400.1835	$C_{19}H_{27}N_3O_5Na^+$	-1.8650
GYSLG	474.2324	$C_{21}H_{33}N_5O_6Na^+$	0.2477	502.2276	$C_{22}H_{33}N_5O_7Na^+$	0.7212	519.2543	$C_{22}H_{36}N_6O_7Na^+$	0.9395			
GYSLGA	545.2699	$C_{24}H_{38}N_6O_7Na^+$	0.9279	573.2649	$C_{25}H_{38}N_6O_8Na^+$	0.9901				528.2430	$C_{24}H_{35}N_5O_7Na^+$	0.2220
GYSLGAH										665.3020	$C_{30}H_{42}N_8O_8Na^+$	0.3554

Supplementary Table 19 Peak list exported from SurfaceLab spectrum of pig lipase, consisting of ions detected in the spectrum and assigned as internal fragment of the amino acid sequence SLGAHA. The *m/z* values represent the experimentally observed center mass of each peak. The deviation (dev.) represents the parts per million (ppm) accuracy of the assignment.

	a			b			b-NH3			a-NH3		
**Description**	***m/z***	**Assignment**	**Dev. (ppm)**	***m/z***	**Assignment**	**Dev. (ppm)**	***m/z***	**Assignment**	**Dev. (ppm)**	***m/z***	**Assignment**	**Dev. (ppm)**
SL				225.1211	$C_9H_{18}N_2O_3Na^+$	0.7658						
SLG	254.1476	$C_{10}H_{21}N_3O_3Na^+$	0.5171	282.1426	$C_{11}H_{21}N_3O_4Na^+$	0.7445	265.1160	$C_{11}H_{18}N_2O_4Na^+$	0.5055	237.1212	$C_{10}H_{18}N_2O_3Na^+$	0.9459
SLGA	325.1850	$C_{13}H_{26}N_4O_4Na^+$	1.0277	353.1798	$C_{14}H_{26}N_4O_5Na^+$	0.8529	336.1534	$C_{14}H_{23}N_3O_5Na^+$	1.2479	308.1583	$C_{13}H_{23}N_3O_4Na^+$	0.8333
SLGAH				490.2384	$C_{20}H_{33}N_7O_6Na^+$	-0.0446				445.2172	$C_{19}H_{30}N_6O_5Na^+$	0.4019
SLGAHA										516.2539	$C_{22}H_{35}N_7O_6Na^+$	-0.3258

Supplementary Table 20 Peak list exported from SurfaceLab spectrum of pig lipase, consisting of ions detected in the spectrum and assigned as internal fragment of the amino acid sequence IGIQKP. The *m/z* values represent the experimentally observed center mass of each peak. The deviation (dev.) represents the parts per million (ppm) accuracy of the assignment.

	a			b			b-NH3			a-NH3		
**Description**	***m/z***	**Assignment**	**Dev. (ppm)**	***m/z***	**Assignment**	**Dev. (ppm)**	***m/z***	**Assignment**	**Dev. (ppm)**	***m/z***	**Assignment**	**Dev. (ppm)**
IG	167.1154	$C_7H_{16}N_2ONa^+$	-0.5585	195.1104	$C_8H_{16}N_2O_2Na^+$	0.0006	178.0838	$C_8H_{13}NO_2Na^+$	-0.4331			
IGI	280.1999	$C_{13}H_{27}N_3O_2Na^+$	1.0821	308.1947	$C_{14}H_{27}N_3O_3Na^+$	0.6890	291.1681	$C_{14}H_{24}N_2O_3Na^+$	0.5136	263.1733	$C_{13}H_{24}N_2O_2Na^+$	0.9733
IGIQ	408.2584	$C_{18}H_{35}N_5O_4Na^+$	0.7911	436.2534	$C_{19}H_{35}N_5O_5Na^+$	0.8991	419.2268	$C_{19}H_{32}N_4O_5Na^+$	0.7349	391.2319	$C_{18}H_{32}N_4O_4Na^+$	0.8177
IGIQK				564.3488	$C_{25}H_{47}N_7O_6Na^+$	1.4069	547.3217	$C_{25}H_{44}N_6O_6Na^+$	0.4328	519.3270	$C_{24}H_{44}N_6O_5Na^+$	0.8671
IGIQKP										616.3797	$C_{29}H_{51}N_7O_6Na^+$	0.6457

Supplementary Table 21 Peak list exported from SurfaceLab spectrum of pig lipase, consisting of ions detected in the spectrum and assigned as internal fragment of the amino acid sequence LDPAGP. The *m/z* values represent the experimentally observed center mass of each peak. The deviation (dev.) represents the parts per million (ppm) accuracy of the assignment.

	a			b			c			a-NH3		
**Description**	***m/z***	**Assignment**	**Dev. (ppm)**	***m/z***	**Assignment**	**Dev. (ppm)**	***m/z***	**Assignment**	**Dev. (ppm)**	***m/z***	**Assignment**	**Dev. (ppm)**
LD	225.1211	$C_9H_{18}N_2O_3Na^+$	0.7658	253.1161	$C_{10}H_{18}N_2O_4Na^+$	0.7160				208.0948	$C_9H_{15}NO_3Na^+$	2.0533
LDP	322.1740	$C_{14}H_{25}N_3O_4Na^+$	0.8592	350.1690	$C_{15}H_{25}N_3O_5Na^+$	0.9922	367.1955	$C_{15}H_{28}N_4O_5Na^+$	0.7475			
LDPA	393.2109	$C_{17}H_{30}N_4O_5Na^+$	0.1168	421.2060	$C_{18}H_{30}N_4O_6Na^+$	0.5264	438.2327	$C_{18}H_{33}N_5O_6Na^+$	0.8888			
LDPAG	450.2327	$C_{19}H_{33}N_5O_6Na^+$	0.8807	478.2274	$C_{20}H_{33}N_5O_7Na^+$	0.3133	495.2541	$C_{20}H_{36}N_6O_7Na^+$	0.6078	433.2065	$C_{19}H_{30}N_4O_6Na^+$	1.6249
LDPAGP	547.2852	$C_{24}H_{40}N_6O_7Na^+$	0.3037				592.3076	$C_{25}H_{43}N_7O_8Na^+$	1.8701	530.2572	$C_{24}H_{37}N_5O_7Na^+$	-2.4918

Supplementary Table 22 Peak list exported from SurfaceLab spectrum of pig lipase, consisting of ions detected in the spectrum and assigned as internal fragment of the amino acid sequence SPGFAI. The *m/z* values represent the experimentally observed center mass of each peak. The deviation (dev.) represents the parts per million (ppm) accuracy of the assignment .

	**a**			**b**			**c**			**a-NH3**		
**Description**	***m/z***	**Assignment**	**Dev. (ppm)**	***m/z***	**Assignment**	**Dev. (ppm)**	***m/z***	**Assignment**	**Dev. (ppm)**	***m/z***	**Assignment**	**Dev. (ppm)**
SP	181.0946	$C_7H_{14}N_2O_2Na^+$	-0.5921	209.0898	$C_8H_{14}N_2O_3Na^+$	0.4668	226.1164	$C_8H_{17}N_3O_3Na^+$	0.7155			
SPG	238.1164	$C_9H_{17}N_3O_3Na^+$	0.6732	266.1113	$C_{10}H_{17}N_3O_4Na^+$	0.6278				221.0898	$C_9H_{14}N_2O_3Na^+$	0.6557
SPGF	385.1849	$C_{18}H_{26}N_4O_4Na^+$	0.7905	413.1800	$C_{19}H_{26}N_4O_5Na^+$	1.0528	430.2071	$C_{19}H_{29}N_5O_5Na^+$	2.2363	368.1585	$C_{18}H_{23}N_3O_4Na^+$	1.2226
SPGFA	456.2220	$C_{21}H_{31}N_5O_5Na^+$	0.6232	484.2170	$C_{22}H_{31}N_5O_6Na^+$	0.7201	501.2436	$C_{22}H_{34}N_6O_6Na^+$	0.6989	439.1952	$C_{21}H_{28}N_4O_5Na^+$	0.0313
SPGFAI	569.3063	$C_{27}H_{42}N_6O_6Na^+$	0.8595	597.3005	$C_{28}H_{42}N_6O_7Na^+$	-0.4383	614.3282	$C_{28}H_{45}N_7O_7Na^+$	1.4916			

Cytochrome C

a)

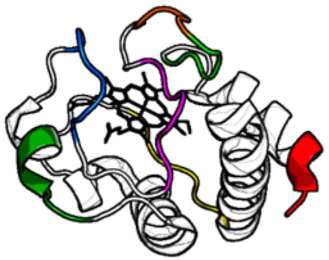

b)

Uniprot database code	Amino acid sequence
CYC_HORSE1n	GDVEKGKKIFVQKCAQCHTVEKGGKHKTGPNLHGLFGRKTGQAPGFTYTDAN<br>KNKGITWKEETLMEYLENPKKYIPGTKMIFAGIKKKTEREDLIAYLKKATNE

Supplementary Figure 18 Horse cytochrome C (a) cartoon exported from PDB entry 1HRC[8] and (b) amino acid sequence exported from the UniProt database. The highlighted colours correspond to assigned segments of the amino acid sequence, presented in Supplementary Tables 23-30.

Supplementary Table 23 Peak list exported from SurfaceLab spectrum of horse cytochrome C, consisting of ions detected in the spectrum and assigned as fragments of N-terminal sequence GDVE. The *m/z* values represent the experimentally observed center mass of each peak. The deviation (dev.) represents the parts per million (ppm) accuracy of the assignment. The colour corresponds to the presence of the observed sequence in horse cytochrome C presented in Supplementary Figure 18.

	**a**			**b**			**b-NH3**			**a-NH3**		
**Description**	***m/z***	**Assignment**	**Dev. (ppm)**	***m/z***	**Assignment**	**Dev. (ppm)**	***m/z***	**Assignment**	**Dev. (ppm)**	***m/z***	**Assignment**	**Dev. (ppm)**
GDV	266.1111	$C_{10}H_{17}N_3O_4Na^+$	-0.1672	294.1059	$C_{11}H_{17}N_3O_5Na^+$	-0.3812	277.0796	$C_{11}H_{14}N_2O_5Na^+$	0.2842			
GDVE	395.1541	$C_{15}H_{24}N_4O_7Na^+$	1.0855				406.1210	$C_{16}H_{21}N_3O_8Na^+$	-2.6012	378.1275	$C_{15}H_{21}N_3O_7Na^+$	0.7418

Supplementary Table 24 Peak list exported from SurfaceLab spectrum of horse cytochrome C, consisting of ions detected in the spectrum and assigned as internal fragment of the amino acid sequence FAGIKK. The *m/z* values represent the experimentally observed center mass of each peak. The deviation (dev.) represents the parts per million (ppm) accuracy of the assignment. The colour corresponds to the presence of the observed sequence in horse cytochrome C presented in Supplementary Figure 18.

	**a**			**b**			**c**			**a-NH3**		
**Description**	***m/z***	**Assignment**	**Dev. (ppm)**	***m/z***	**Assignment**	**Dev. (ppm)**	***m/z***	**Assignment**	**Dev. (ppm)**	***m/z***	**Assignment**	**Dev. (ppm)**
FA				243.1103	$C_{12}H_{16}N_2O_2Na^+$	-0.2601	260.1370	$C_{12}H_{19}N_3O_2Na^+$	0.1744			
FAG	272.1370	$C_{13}H_{19}N_3O_2Na^+$	0.0518	328.1267	$C_{15}H_{19}N_3O_4Na^+$	-0.2086	345.1533	$C_{15}H_{22}N_4O_4Na^+$	-0.1971	255.1103	$C_{13}H_{16}N_2O_2Na^+$	-0.3864
FAGI	385.2210	$C_{19}H_{30}N_4O_3Na^+$	-0.0970	413.2158	$C_{20}H_{30}N_4O_4Na^+$	-0.2262	430.2424	$C_{20}H_{33}N_5O_4Na^+$	-0.0697	368.1944	$C_{19}H_{27}N_3O_3Na^+$	-0.2789
FAGIK	513.3154	$C_{25}H_{42}N_6O_4Na^+$	-1.0533	541.3112	$C_{26}H_{42}N_6O_5Na^+$	0.5132	558.3374	$C_{26}H_{45}N_7O_5Na^+$	-0.1064	496.2893	$C_{25}H_{39}N_5O_4Na^+$	-0.1659
FAGIKK				669.4062	$C_{32}H_{54}N_8O_6Na^+$	0.4462				624.3847	$C_{31}H_{51}N_7O_5Na^+$	0.4207

Supplementary Table 25 Peak list exported from SurfaceLab spectrum of horse cytochrome C, consisting of ions detected in the spectrum and assigned as internal fragment of the amino acid sequence GGKHK. The *m/z* values represent the experimentally observed center mass of each peak. The deviation (dev.) represents the parts per million (ppm) accuracy of the assignment. The colour corresponds to the presence of the observed sequence in horse cytochrome C presented in Supplementary Figure 18.

	**a**			**b**			**c**			**a-NH3**		
**Description**	***m/z***	**Assignment**	**Dev. (ppm)**	***m/z***	**Assignment**	**Dev. (ppm)**	***m/z***	**Assignment**	**Dev. (ppm)**	***m/z***	**Assignment**	**Dev. (ppm)**
GGK	239.1478	$C_9H_{20}N_4O_2Na^+$	-0.0413	267.1427	$C_{10}H_{20}N_4O_3Na^+$	-0.2282				222.1212	$C_9H_{17}N_3O_2Na^+$	-0.2298
GGKH	376.2067	$C_{15}H_{27}N_7O_3Na^+$	-0.1873	404.2019	$C_{16}H_{27}N_7O_4Na^+$	0.5863	421.2283	$C_{16}H_{30}N_8O_4Na^+$	0.2544	359.1801	$C_{15}H_{24}N_6O_3Na^+$	-0.1918
GGKHK										487.2747	$C_{21}H_{36}N_8O_4Na^+$	-0.9404

Supplementary Table 26 Peak list exported from SurfaceLab spectrum of horse cytochrome C, consisting of ions detected in the spectrum and assigned as internal fragment of the amino acid sequence GQAPGF. The *m/z* values represent the experimentally observed center mass of each peak. The deviation (dev.) represents the parts per million (ppm) accuracy of the assignment. The colour corresponds to the presence of the observed sequence in horse cytochrome C presented in Supplementary Figure 18.

	a			b			b-NH3			a-NH3		
**Description**	***m/z***	**Assignment**	**Dev. (ppm)**	***m/z***	**Assignment**	**Dev. (ppm)**	***m/z***	**Assignment**	**Dev. (ppm)**	***m/z***	**Assignment**	**Dev. (ppm)**
GQ							193.0582	$C_7H_{10}N_2O_3Na^+$	-0.6373	165.0632	$C_6H_{10}N_2O_2Na^+$	-1.3415
GQA	253.1269	$C_9H_{18}N_4O_3Na^+$	-0.9330				264.0956	$C_{10}H_{15}N_3O_4Na^+$	0.2834	236.1006	$C_9H_{15}N_3O_3Na^+$	-0.0515
GQAP	350.1798	$C_{14}H_{25}N_5O_4Na^+$	-0.3331	378.1748	$C_{15}H_{25}N_5O_5Na^+$	0.0244	361.1486	$C_{15}H_{22}N_4O_5Na^+$	0.8643	333.1532	$C_{14}H_{22}N_4O_4Na^+$	-0.2783
GQAPG	407.2013	$C_{16}H_{28}N_6O_5Na^+$	-0.0641	435.1965	$C_{17}H_{28}N_6O_6Na^+$	0.5403	418.1702	$C_{17}H_{25}N_5O_6Na^+$	1.1538	390.1747	$C_{16}H_{25}N_5O_5Na^+$	-0.1053
GQAPGF	554.2702	$C_{25}H_{37}N_7O_6Na^+$	0.8474	582.2652	$C_{26}H_{37}N_7O_7Na^+$	0.9784	565.2375	$C_{26}H_{34}N_6O_7Na^+$	-1.1229	537.2434	$C_{25}H_{34}N_6O_6Na^+$	0.3487

Supplementary Table 27 Peak list exported from SurfaceLab spectrum of horse cytochrome C, consisting of ions detected in the spectrum and assigned as internal fragment of the amino acid sequence HTVEK. The *m/z* values represent the experimentally observed center mass of each peak. The deviation (dev.) represents the parts per million (ppm) accuracy of the assignment. The colour corresponds to the presence of the observed sequence in horse cytochrome C presented in Supplementary Figure 18.

	a			b			c			a-NH3		
**Description**	***m/z***	**Assignment**	**Dev. (ppm)**	***m/z***	**Assignment**	**Dev. (ppm)**	***m/z***	**Assignment**	**Dev. (ppm)**	***m/z***	**Assignment**	**Dev. (ppm)**
HTV	334.1849	$C_{14}H_{25}N_5O_3Na^+$	-0.3184	362.1798	$C_{15}H_{25}N_5O_4Na^+$	-0.2032	379.2064	$C_{15}H_{28}N_6O_4Na^+$	-0.1009	317.1584	$C_{14}H_{22}N_4O_3Na^+$	-0.1106
HTVE	463.2278	$C_{19}H_{32}N_6O_6Na^+$	0.4486	491.2228	$C_{20}H_{32}N_6O_7Na^+$	0.7376				446.2010	$C_{19}H_{29}N_5O_6Na^+$	0.0005
HTVEK	591.3249	$C_{25}H_{44}N_8O_7Na^+$	3.9919									

Supplementary Table 28 Peak list exported from SurfaceLab spectrum of horse cytochrome C, consisting of ions detected in the spectrum and assigned as internal fragment of the amino acid sequence KNKGI. The *m/z* values represent the experimentally observed center mass of each peak. The deviation (dev.) represents the parts per million (ppm) accuracy of the assignment. The colour corresponds to the presence of the observed sequence in horse cytochrome C presented in Supplementary Figure 18.

	a			b			b-NH3			a-NH3		
**Description**	***m/z***	**Assignment**	**Dev. (ppm)**	***m/z***	**Assignment**	**Dev. (ppm)**	***m/z***	**Assignment**	**Dev. (ppm)**	***m/z***	**Assignment**	**Dev. (ppm)**
KN	239.1478	$C_9H_{20}N_4O_2Na^+$	-0.0413	267.1427	$C_{10}H_{20}N_4O_3Na^+$	-0.2282	250.1162	$C_{10}H_{17}N_3O_3Na^+$	-0.1971	222.1212	$C_9H_{17}N_3O_2Na^+$	-0.2298
KNK	367.2427	$C_{15}H_{32}N_6O_3Na^+$	-0.2686	395.2378	$C_{16}H_{32}N_6O_4Na^+$	0.2264	378.2112	$C_{16}H_{29}N_5O_4Na^+$	0.0838	350.2162	$C_{15}H_{29}N_5O_3Na^+$	-0.2268
KNKG	424.2644	$C_{17}H_{35}N_7O_4Na^+$	0.2052				435.2327	$C_{18}H_{32}N_6O_5Na^+$	0.1435	407.2377	$C_{17}H_{32}N_6O_4Na^+$	-0.1373
KNKGI	537.3493	$C_{23}H_{46}N_8O_5Na^+$	1.8029							520.3221	$C_{23}H_{43}N_7O_5Na^+$	0.5788

Supplementary Table 29 Peak list exported from SurfaceLab spectrum of horse cytochrome C, consisting of ions detected in the spectrum and assigned as internal fragment of the amino acid sequence NKGI. The *m/z* values represent the experimentally observed center mass of each peak. The deviation (dev.) represents the parts per million (ppm) accuracy of the assignment. The colour corresponds to the presence of the observed sequence in horse cytochrome C presented in Supplementary Figure 18.

	a			b			b-NH3			a-NH3		
**Description**	***m/z***	**Assignment**	**Dev. (ppm)**	***m/z***	**Assignment**	**Dev. (ppm)**	***m/z***	**Assignment**	**Dev. (ppm)**	***m/z***	**Assignment**	**Dev. (ppm)**
NK	239.1478	$C_9H_{20}N_4O_2Na^+$	-0.0413	267.1427	$C_{10}H_{20}N_4O_3Na^+$	-0.2282	250.1162	$C_{10}H_{17}N_3O_3Na^+$	-0.1971	222.1212	$C_9H_{17}N_3O_2Na^+$	-0.2298
NKG	296.1693	$C_{11}H_{23}N_5O_3Na^+$	-0.0599	324.1642	$C_{12}H_{23}N_5O_4Na^+$	0.0370	307.1377	$C_{12}H_{20}N_4O_4Na^+$	0.0012	279.1427	$C_{11}H_{20}N_4O_3Na^+$	-0.1187
NKGI	409.2534	$C_{17}H_{34}N_6O_4Na^+$	-0.0032				420.2220	$C_{18}H_{31}N_5O_5Na^+$	0.5382	392.2268	$C_{17}H_{31}N_5O_4Na^+$	-0.0818

Supplementary Table 30 Peak list exported from SurfaceLab spectrum of horse cytochrome C, consisting of ions detected in the spectrum and assigned as internal fragment of the amino acid sequence NLHGLF. The *m/z* values represent the experimentally observed center mass of each peak. The deviation (dev.) represents the parts per million (ppm) accuracy of the assignment. The colour corresponds to the presence of the observed sequence in horse cytochrome C presented in Supplementary Figure 18.

	**a**			**b**			**b-NH3**			**a-NH3**		
**Description**	***m/z***	**Assignment**	**Dev. (ppm)**	***m/z***	**Assignment**	**Dev. (ppm)**	***m/z***	**Assignment**	**Dev. (ppm)**	***m/z***	**Assignment**	**Dev. (ppm)**
NL	224.1369	$C_9H_{19}N_3O_2Na^+$	-0.1352	252.1318	$C_{10}H_{19}N_3O_3Na^+$	-0.3651	235.1057	$C_{10}H_{16}N_2O_3Na^+$	1.5857	207.1103	$C_9H_{16}N_2O_2Na^+$	-0.4490
NLH	361.1959	$C_{15}H_{26}N_6O_3Na^+$	0.2314	389.1909	$C_{16}H_{26}N_6O_4Na^+$	0.2361	372.1643	$C_{16}H_{23}N_5O_4Na^+$	0.0675	344.1692	$C_{15}H_{23}N_5O_3Na^+$	-0.2171
NLHG				446.2122	$C_{18}H_{29}N_7O_5Na^+$	-0.0181	429.1859	$C_{18}H_{26}N_6O_5Na^+$	0.4262	401.1908	$C_{17}H_{26}N_6O_4Na^+$	0.1013
NLHGL	531.3027	$C_{23}H_{40}N_8O_5Na^+$	2.3953	559.2975	$C_{24}H_{40}N_8O_6Na^+$	2.0653	542.2694	$C_{24}H_{37}N_7O_6Na^+$	-0.6688	514.2749	$C_{23}H_{37}N_7O_5Na^+$	0.1239
NLHGLF										661.3426	$C_{32}H_{46}N_8O_6Na^+$	-1.0393

Lysozyme

a)

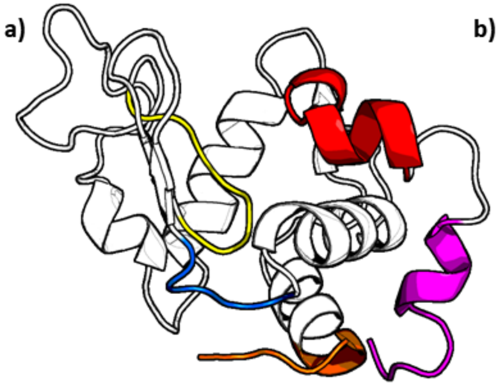

b)

Uniprot database code	Amino acid sequence
LYSC_CHICK1n	KVFGRCELAAAMKRHGLDNYRGYSLGNWVCAAKFESNFNTQATNRNTDGS<br>TDYGILQINSRWWCNDGRTPGSRNLCNIPCSALLSSDITASVNCAKKIVSDGN<br>GMNAWVAWRNRCKGTDVQAWIRGCRL

Supplementary Figure 19 Horse lysozyme (a) cartoon exported from PDB entry 1AZF[9] and (b) amino acid sequence exported from the UniProt database. The highlighted colours correspond to assigned segments of the amino acid sequence, presented in Supplementary Tables 31-36.

Supplementary Table 31 Peak list exported from SurfaceLab spectrum of chicken egg lysozyme, consisting of ions detected in the spectrum and assigned as sodium adducts of N-terminal sequence KVFGRCE. The sequence is observed as a, b, c and a-NH$_3$ ions. The *m/z* values represent the experimentally observed center mass of each peak. The deviation (dev.) represents the parts per million (ppm) accuracy of the assignment. The colour corresponds to the presence of the observed sequence in chicken egg lysozyme presented in Supplementary Figure 19.

	a			b			c			a-NH$_3$		
**Description**	***m/z***	**Assignment**	**Dev. (ppm)**	***m/z***	**Assignment**	**Dev. (ppm)**	***m/z***	**Assignment**	**Dev. (ppm)**	***m/z***	**Assignment**	**Dev. (ppm)**
KVF	369.2260	$C_{19}H_{30}N_4O_2Na^+$	-0.1771	397.2210	$C_{20}H_{30}N_4O_3Na^+$	-0.0668	414.2473	$C_{20}H_{33}N_5O_3Na^+$	-0.5331	x	x	x
KVFG	426.2475	$C_{21}H_{33}N_5O_3Na^+$	-0.1383	454.2422	$C_{22}H_{33}N_5O_4Na^+$	-0.5043	471.2689	$C_{22}H_{36}N_6O_4Na^+$	-0.2544	409.2207	$C_{21}H_{30}N_4O_3Na^+$	-0.7236
KVFGR	582.3493	$C_{27}H_{45}N_9O_4Na^+$	0.9943	610.3447	$C_{28}H_{45}N_9O_5Na^+$	1.7820	627.3709	$C_{28}H_{48}N_{10}O_5Na^+$	1.1688	565.3217	$C_{27}H_{42}N_8O_4Na^+$	-0.7593
KVFGRC	685.3588	$C_{30}H_{50}N_{10}O_5SNa^+$	1.3660	713.3531	$C_{31}H_{50}N_{10}O_6SNa^+$	0.5080	730.3795	$C_{31}H_{53}N_{11}O_6SNa^+$	0.2715	x	x	x
KVFGRCE	814.4001	$C_{35}H_{57}N_{11}O_8SNa^+$	-0.3742	x	x	x	859.4222	$C_{36}H_{60}N_{12}O_9SNa^+$	0.3087	x	x	x

Supplementary Table 32 Peak list exported from SurfaceLab spectrum of chicken egg lysozyme, consisting of ions detected in the spectrum and assigned as N-terminal sequence KVFGRCE. The sequence is observed as a, b, c and a-NH_3 ions. The *m/z* values represent the experimentally observed center mass of each peak. The deviation (dev.) represents the parts per million (ppm) accuracy of the assignment. The colour corresponds to the presence of the observed sequence in chicken egg lysozyme presented in Supplementary Figure 19.

	a			b			c			a-NH_3		
**Description**	***m/z***	**Assignment**	**Dev. (ppm)**	***m/z***	**Assignment**	**Dev. (ppm)**	***m/z***	**Assignment**	**Dev. (ppm)**	***m/z***	**Assignment**	**Dev. (ppm)**
KVFG	x	x	x	x	x	x	449.2871	$C_{22}H_{37}N_6O_4^+$	-0.0082	x	x	x
KVFGR	560.3669	$C_{27}H_{46}N_9O_4^+$	0.2947	588.3618	$C_{28}H_{46}N_9O_5^+$	0.2719	605.3884	$C_{28}H_{49}N_{10}O_5^+$	0.3188	543.3404	$C_{27}H_{43}N_8O_4^+$	0.38712
KVFGRC	663.3763	$C_{30}H_{51}N_{10}O_5S^+$	0.5377	691.3713	$C_{31}H_{51}N_{10}O_6S^+$	0.7062	708.3970	$C_{31}H_{54}N_{11}O_6S^+$	-0.4672	x	x	x
KVFGRCE	792.4187	$C_{35}H_{58}N_{11}O_8S^+$	0.2363	820.4125	$C_{36}H_{58}N_{11}O_9S^+$	-1.1700	837.4403	$C_{36}H_{61}N_{12}O_9S^+$	0.3673	x	x	x

Supplementary Table 33 Peak list exported from SurfaceLab spectrum of chicken egg lysozyme, consisting of ions detected in the spectrum and assigned as sodium adducts of an internal fragment of the amino acid sequence, GILQINS. The sequence is observed as ya, yb, yc and ya-NH_3 ions. The *m/z* values represent the experimentally observed center mass of each peak. The deviation (dev.) represents the parts per million (ppm) accuracy of the assignment. The colour corresponds to the presence of the observed sequence in chicken egg lysozyme presented in Supplementary Figure 19.

	ya			yb			yc			ya-NH_3		
**Description**	***m/z***	**Assignment**	**Dev. (ppm)**	***m/z***	**Assignment**	**Dev. (ppm)**	***m/z***	**Assignment**	**Dev. (ppm)**	***m/z***	**Assignment**	**Dev. (ppm)**
GI	x	x	x	193.0946	$C_8H_{14}N_2O_2Na^+$	-0.7927	210.1213	$C_8H_{17}N_3O_2Na^+$	-0.2090	x	x	x
GIL	278.1840	$C_{13}H_{25}N_3O_2Na^+$	0.2682	306.1788	$C_{14}H_{25}N_3O_3Na^+$	-0.1843	323.2053	$C_{14}H_{28}N_4O_3Na^+$	-0.1234	261.1574	$C_{13}H_{22}N_2O_2Na^+$	0.0814
GILQ	406.2425	$C_{18}H_{33}N_5O_4Na^+$	0.0092	434.2374	$C_{19}H_{33}N_5O_5Na^+$	-0.0188	451.2644	$C_{19}H_{36}N_6O_5Na^+$	1.0462	389.2159	$C_{18}H_{30}N_4O_4Na^+$	-0.0761
GILQI	519.3266	$C_{24}H_{44}N_6O_5Na^+$	0.2001	547.3217	$C_{25}H_{44}N_6O_6Na^+$	0.4593	564.3480	$C_{25}H_{47}N_7O_6Na^+$	0.0331	x	x	x
GILQIN	633.3693	$C_{28}H_{50}N_8O_7Na^+$	-0.2149	661.3646	$C_{29}H_{50}N_8O_8Na^+$	0.3362	x	x	x	616.3427	$C_{28}H_{47}N_7O_7Na^+$	-0.3422
GILQINS	720.4005	$C_{31}H_{55}N_9O_9Na^+$	-1.3281	x	x	x	x	x	x	x	x	x

Supplementary Table 34 Peak list exported from SurfaceLab spectrum of chicken egg lysozyme, consisting of ions detected in the spectrum and assigned as sodium adducts of an internal fragment of the amino acid sequence, FNTQA. The sequence is observed as ya, yb, yc and ya-NH_3 ions. The *m/z* values represent the experimentally observed center mass of each peak. The deviation (dev.) represents the parts per million (ppm) accuracy of the assignment. The colour corresponds to the presence of the observed sequence in chicken egg lysozyme presented in Supplementary Figure 19.

	ya			yb			yc			ya-NH_3		
**Description**	***m/z***	**Assignment**	**Dev. (ppm)**	***m/z***	**Assignment**	**Dev. (ppm)**	***m/z***	**Assignment**	**Dev. (ppm)**	***m/z***	**Assignment**	**Dev. (ppm)**
FN	258.1214	$C_{12}H_{17}N_3O_2Na^+$	0.5245	286.1163	$C_{13}H_{17}N_3O_3Na^+$	0.3113	303.1428	$C_{13}H_{20}N_4O_3Na^+$	0.0698	x	x	x
FNT	359.1689	$C_{16}H_{24}N_4O_4Na^+$	-0.1399	387.1639	$C_{17}H_{24}N_4O_5Na^+$	-0.0304	404.1904	$C_{17}H_{27}N_5O_5Na^+$	-0.0471	342.1424	$C_{16}H_{21}N_3O_4Na^+$	0.0096
FNTQ	487.2275	$C_{21}H_{32}N_6O_6Na^+$	-0.0114	515.2225	$C_{22}H_{32}N_6O_7Na^+$	0.1231	532.2500	$C_{22}H_{35}N_7O_7Na^+$	1.8018	470.2005	$C_{21}H_{29}N_5O_6Na^+$	-1.0115
FNTQA	558.2651	$C_{24}H_{37}N_7O_7Na^+$	0.8337	586.2593	$C_{25}H_{37}N_7O_8Na^+$	-0.4676	603.2865	$C_{25}H_{40}N_8O_8Na^+$	0.6512	541.2381	$C_{24}H_{34}N_6O_7Na^+$	-0.0159
NTQA	411.1963	$C_{15}H_{28}N_6O_6Na^+$	0.2309	439.1914	$C_{16}H_{28}N_6O_7Na^+$	0.6327	x	x	x	394.1696	$C_{15}H_{25}N_5O_6Na^+$	-0.1482

Supplementary Table 35 Peak list exported from SurfaceLab spectrum of chicken egg lysozyme, consisting of ions detected in the spectrum and assigned as sodium adducts of an internal fragment of the amino acid sequence, NAWVAWRNR. The sequence is observed as ya, yb, yc and ya-NH_3 ions. The *m/z* values represent the experimentally observed center mass of each peak. The deviation (dev.) represents the parts per million (ppm) accuracy of the assignment. The colour corresponds to the presence of the observed sequence in chicken egg lysozyme presented in Supplementary Figure 19.

	ya			yb			yc			ya-NH_3		
**Description**	***m/z***	**Assignment**	**Dev. (ppm)**	***m/z***	**Assignment**	**Dev. (ppm)**	***m/z***	**Assignment**	**Dev. (ppm)**	***m/z***	**Assignment**	**Dev. (ppm)**
NA	180.0743	$C_6H_{11}N_3O_2Na^+$	-0.3451	208.0692	$C_7H_{11}N_3O_3Na^+$	-0.1725	225.0958	$C_7H_{14}N_4O_3Na^+$	0.1671	163.048	$C_6H_8N_2O_2Na^+$	-1.1551
NAW	366.1534	$C_{17}H_{21}N_5O_3Na^+$	-0.7604	394.1484	$C_{18}H_{21}N_5O_4Na^+$	-0.5582	411.1749	$C_{18}H_{24}N_6O_4Na^+$	-0.4455	349.127	$C_{17}H_{18}N_4O_3Na^+$	-0.0065
NAWV	465.2220	$C_{22}H_{30}N_6O_4Na^+$	-0.1686	493.2168	$C_{23}H_{30}N_6O_5Na^+$	-0.2974	510.2430	$C_{23}H_{33}N_7O_5Na^+$	-1.1528	448.195	$C_{22}H_{27}N_5O_4Na^+$	-0.0918
NAWVA	536.2590	$C_{25}H_{35}N_7O_5Na^+$	-0.4148	564.2542	$C_{26}H_{35}N_7O_6Na^+$	0.1925	581.2808	$C_{26}H_{38}N_8O_6Na^+$	0.2173	519.233	$C_{25}H_{32}N_6O_5Na^+$	0.1979
NAWVAW	722.3385	$C_{36}H_{45}N_9O_6Na^+$	-0.0096	750.3339	$C_{37}H_{45}N_9O_7Na^+$	0.6554	767.3604	$C_{37}H_{48}N_{10}O_7Na^+$	0.6248	705.312	$C_{36}H_{42}N_8O_6Na^+$	0.2354
NAWVAWR	878.4399	$C_{42}H_{57}N_{13}O_7Na^+$	0.3470	906.4350	$C_{43}H_{57}N_{13}O_8Na^+$	0.4979	923.4617	$C_{43}H_{60}N_{14}O_8Na^+$	0.6619	x	x	x
NAWVAWRN	x	x	x	1020.4761	$C_{47}H_{63}N_{15}O_{10}Na^+$	-1.3684	x	x	x	x	x	x
NAWVAWRNR	x	x	x	1176.5789	$C_{53}H_{75}N_{19}O_{11}Na^+$	0.2907	x	x	x	1,131.5503	$C_{52}H_{72}N_{18}O_{10}Na^+$	-6.0393

Supplementary Table 36 Peak list exported from SurfaceLab spectrum of chicken egg lysozyme, consisting of ions detected in the spectrum and assigned as C-terminal sequence DVQAWIRGCRL. The sequence is observed as y, z-1, z+1 and z+1-SH_2 ions. The *m/z* values represent the experimentally observed center mass of each peak. The deviation (dev.) represents the parts per million (ppm) accuracy of the assignment. The colour corresponds to the presence of the observed sequence in chicken egg lysozyme presented in Supplementary Figure 19.

	**y**			**z-1**			**z+1**			**z+1-SH_2**		
**Description**	***m/z***	**Assignment**	**Dev. (ppm)**	***m/z***	**Assignment**	**Dev. (ppm)**	***m/z***	**Assignment**	**Dev. (ppm)**	***m/z***	**Assignment**	**Dev. (ppm)**
RL	288.2030	$C_{12}H_{26}N_5O_3^+$	-0.0232	271.1765	$C_{12}H_{23}N_4O_3^+$	-0.0394	273.1920	$C_{12}H_{25}N_4O_3^+$	-0.2931	x	x	x
CRL	x	x	x	x	x	x	x	x	x	342.2137	$C_{15}H_{28}N_5O_4^+$	0.4080
GCRL	x	x	x	x	x	x	433.2231	$C_{17}H_{33}N_6O_5S^+$	0.7444	x	x	x
RGCRL	x	x	x	587.3074	$C_{23}H_{43}N_{10}O_6S^+$	-1.3632	589.3241	$C_{23}H_{45}N_{10}O_6S^+$	0.3742	555.3364	$C_{23}H_{43}N_{10}O_6^+$	0.4417
IRGCRL	x	x	x	700.3918	$C_{29}H_{54}N_{11}O_7S^+$	-0.7718	702.4079	$C_{29}H_{56}N_{11}O_7S^+$	-0.0981	668.4207	$C_{29}H_{54}N_{11}O_7^+$	0.6856
WIRGCRL	x	x	x	886.4697	$C_{40}H_{64}N_{13}O_8S^+$	-2.1753	888.4875	$C_{40}H_{66}N_{13}O_8S^+$	0.2773	854.4993	$C_{40}H_{64}N_{13}O_8^+$	-0.2662
AWIRGCRL	x	x	x	957.5085	$C_{43}H_{69}N_{14}O_9S^+$	-0.2061	959.5243	$C_{43}H_{71}N_{14}O_9S^+$	-0.0749	925.5357	$C_{43}H_{69}N_{14}O_9^+$	-1.0216
QAWIRGCRL	x	x	x	1085.5692	$C_{48}H_{77}N_{16}O_{11}S^+$	1.7543	1087.5831	$C_{48}H_{79}N_{16}O_{11}S^+$	0.1508	x	x	x
VQAWIRGCRL	x	x	x	1184.6359	$C_{53}H_{86}N_{17}O_{12}S^+$	0.1884	1186.6513	$C_{53}H_{88}N_{17}O_{12}S^+$	-0.0846	x	x	x
DVQAWIRGCRL	x	x	x	x	x	x	1301.6790	$C_{57}H_{93}N_{18}O_{15}S^+$	0.5609	x	x	x

Myoglobin

a)

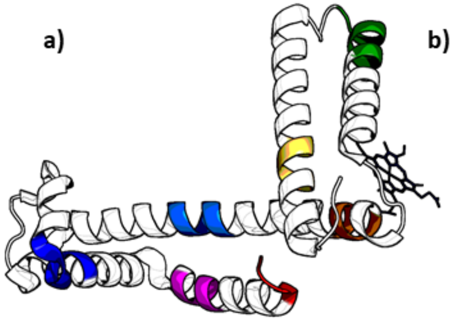

b)

Uniprot database code	Amino acid sequence
MYG_HORSE1n	GLSDGEWQQVLNVWGKVEADIAGHGQEVLIRLFTGHPETLEKFDKFKHLKTEA<br>EMKASEDLKKHGTVVLTALGGILKKKGHHEAELKPLAQSHATKHKIPIKYLEFISD<br>AIIHVLHSKHPGDFGADAQGAMTKALELFRNDIAAKYKELGFQG

Supplementary Figure 20 Horse myoglobin (a) cartoon exported from PDB entry 1WLA[10] and (b) amino acid sequence exported from the UniProt database. The highlighted colours correspond to assigned segments of the amino acid sequence, presented in Supplementary Tables 37-43.

Supplementary Table 37 Peak list exported from SurfaceLab spectrum of horse myoglobin, consisting of ions detected in the spectrum and assigned as fragments of N-terminal sequence GLSD. The *m/z* values represent the experimentally observed center mass of each peak. The deviation (dev.) represents the parts per million (ppm) accuracy of the assignment. The colour corresponds to the presence of the observed sequence in horse myoglobin presented in Supplementary Figure 20.

	a			b			c			a-NH3		
**Description**	***m/z***	**Assignment**	**Dev. (ppm)**	***m/z***	**Assignment**	**Dev. (ppm)**	***m/z***	**Assignment**	**Dev. (ppm)**	***m/z***	**Assignment**	**Dev. (ppm)**
GLS	252.1319	$C_{10}H_{19}N_3O_3Na^+$	0.3369	280.1269	$C_{11}H_{19}N_3O_4Na^+$	0.4303	297.1534	$C_{11}H_{22}N_4O_4Na^+$	0.2136	235.1057	$C_{10}H_{16}N_2O_3Na^+$	1.5510
GLSD				395.154	$C_{15}H_{24}N_4O_7Na^+$	0.8123	412.1814	$C_{15}H_{27}N_5O_7Na^+$	2.7667	350.1327	$C_{14}H_{21}N_3O_6Na^+$	1.1871

Supplementary Table 38 Peak list exported from SurfaceLab spectrum of horse myoglobin, consisting of ions detected in the spectrum and assigned as sodium adducts of an internal fragment of the sequence ALGGIL. The *m/z* values represent the experimentally observed center mass of each peak. The deviation (dev.) represents the parts per million (ppm) accuracy of the assignment. The colour corresponds to the presence of the observed sequence in horse myoglobin presented in Supplementary Figure 20.

	a			b			c			a-NH3		
**Description**	***m/z***	**Assignment**	**Dev. (ppm)**	***m/z***	**Assignment**	**Dev. (ppm)**	***m/z***	**Assignment**	**Dev. (ppm)**	***m/z***	**Assignment**	**Dev. (ppm)**
ALG	238.1522	$C_{10}H_{21}N_3O_2Na^+$	-1.7720	266.1477	$C_{11}H_{21}N_3O_3Na^+$	0.6087				221.1261	$C_{10}H_{18}N_2O_2Na^+$	0.1973
ALGG	295.1742	$C_{12}H_{24}N_4O_3Na^+$	0.4756	323.1691	$C_{13}H_{24}N_4O_4Na^+$	0.2423				278.1476	$C_{12}H_{21}N_3O_3Na^+$	0.1945
ALGGI	408.2582	$C_{18}H_{35}N_5O_4Na^+$	0.1620	436.2530	$C_{19}H_{35}N_5O_5Na^+$	-0.0869				391.2316	$C_{18}H_{32}N_4O_4Na^+$	0.0462
ALGGIL	0.0000			549.3370	$C_{25}H_{46}N_6O_6Na^+$	-0.1500						

Supplementary Table 39 Peak list exported from SurfaceLab spectrum of horse myoglobin, consisting of ions detected in the spectrum and assigned as sodium adducts of an internal fragment of the sequence FRNDI. The *m/z* values represent the experimentally observed center mass of each peak. The deviation (dev.) represents the parts per million (ppm) accuracy of the assignment. The colour corresponds to the presence of the observed sequence in horse myoglobin presented in Supplementary Figure 20.

	a			b			c			a-NH3		
**Description**	***m/z***	**Assignment**	**Dev. (ppm)**	***m/z***	**Assignment**	**Dev. (ppm)**	***m/z***	**Assignment**	**Dev. (ppm)**	***m/z***	**Assignment**	**Dev. (ppm)**
FR				328.1744	$C_{15}H_{23}N_5O_2Na^+$	-0.0727						
FRN	414.2226	$C_{18}H_{29}N_7O_3Na^+$	0.5420	442.2175	$C_{19}H_{29}N_7O_4Na^+$	0.3747	459.2440	$C_{19}H_{32}N_8O_4Na^+$	0.3071	397.1960	$C_{18}H_{26}N_6O_3Na^+$	0.3503
FRND	529.2497	$C_{22}H_{34}N_8O_6Na^+$	0.7258	557.2445	$C_{23}H_{34}N_8O_7Na^+$	0.4853	574.2712	$C_{23}H_{37}N_9O_7Na^+$	0.7430	512.2231	$C_{22}H_{31}N_7O_6Na^+$	0.5690
FRNDI	642.3335	$C_{28}H_{45}N_9O_7Na^+$	0.1567	670.3294	$C_{29}H_{45}N_9O_8Na^+$	1.6481	687.3555	$C_{29}H_{48}N_{10}O_8Na^+$	0.9497			

Supplementary Table 40 Peak list exported from SurfaceLab spectrum of horse myoglobin, consisting of ions detected in the spectrum and assigned as sodium adducts of an internal fragment of the sequence HVLHSKHP. The *m/z* values represent the experimentally observed center mass of each peak. The deviation (dev.) represents the parts per million (ppm) accuracy of the assignment. The colour corresponds to the presence of the observed sequence in horse myoglobin presented in Supplementary Figure 20.

	a			b			c			a-NH3		
**Description**	***m/z***	**Assignment**	**Dev. (ppm)**	***m/z***	**Assignment**	**Dev. (ppm)**	***m/z***	**Assignment**	**Dev. (ppm)**	***m/z***	**Assignment**	**Dev. (ppm)**
HV				261.1323	$C_{11}H_{18}N_4O_2Na^+$	0.3211				216.1108	$C_{10}H_{15}N_3ONa^+$	0.2988
HVL	346.2216	$C_{16}H_{29}N_5O_2Na^+$	0.6610	374.2163	$C_{17}H_{29}N_5O_3Na^+$	0.1477				329.1949	$C_{16}H_{26}N_4O_2Na^+$	0.2332
HVLH	483.2809	$C_{22}H_{36}N_8O_3Na^+$	1.3691	511.2758	$C_{23}H_{36}N_8O_4Na^+$	1.2599				466.2540	$C_{22}H_{33}N_7O_3Na^+$	0.6384
HVLHS	570.3125	$C_{25}H_{41}N_9O_5Na^+$	0.3241	598.3076	$C_{26}H_{41}N_9O_6Na^+$	0.6286				553.2859	$C_{25}H_{38}N_8O_5Na^+$	0.3075
HVLHSK	698.4065	$C_{31}H_{53}N_{11}O_6Na^+$	-1.0156	726.4016	$C_{32}H_{53}N_{11}O_7Na^+$	-0.8272				681.3802	$C_{31}H_{50}N_{10}O_6Na^+$	-0.7400
HVLHSKH				863.4581	$C_{38}H_{60}N_{14}O_8Na^+$	-3.4400				818.4402	$C_{37}H_{57}N_{13}O_7Na^+$	0.7635
HVLHSKHP										915.4949	$C_{42}H_{64}N_{14}O_8Na^+$	2.7602

Supplementary Table 41 Peak list exported from SurfaceLab spectrum of horse myoglobin, consisting of ions detected in the spectrum and assigned as sodium adducts of an internal fragment of the sequence PLAQS. The *m/z* values represent the experimentally observed center mass of each peak. The deviation (dev.) represents the parts per million (ppm) accuracy of the assignment. The colour corresponds to the presence of the observed sequence in horse myoglobin presented in Supplementary Figure 20.

	a			b			c			a-NH3		
**Description**	***m/z***	**Assignment**	**Dev. (ppm)**	***m/z***	**Assignment**	**Dev. (ppm)**	***m/z***	**Assignment**	**Dev. (ppm)**	***m/z***	**Assignment**	**Dev. (ppm)**
PL				235.1418	$C_{11}H_{20}N_2O_2Na^+$	0.2555	252.1683	$C_{11}H_{23}N_3O_2Na^+$	0.1584			
PLA	278.1841	$C_{13}H_{25}N_3O_2Na^+$	0.5933	306.1789	$C_{14}H_{25}N_3O_3Na^+$	0.2692	323.2054	$C_{14}H_{28}N_4O_3Na^+$	0.0289	261.1575	$C_{13}H_{22}N_2O_2Na^+$	0.3931
PLAQ	406.2426	$C_{18}H_{33}N_5O_4Na^+$	0.2284	434.2376	$C_{19}H_{33}N_5O_5Na^+$	0.4319	451.2643	$C_{19}H_{36}N_6O_5Na^+$	0.7535	389.2160	$C_{18}H_{30}N_4O_4Na^+$	0.2063
PLAQS	493.2748	$C_{21}H_{38}N_6O_6Na^+$	0.6402	521.2699	$C_{22}H_{38}N_6O_7Na^+$	0.8378				476.2482	$C_{21}H_{35}N_5O_6Na^+$	0.5893

Supplementary Table 42 Peak list exported from SurfaceLab spectrum of horse myoglobin, consisting of ions detected in the spectrum and assigned as sodium adducts of an internal fragment of the sequence VWGKV. The *m/z* values represent the experimentally observed center mass of each peak. The deviation (dev.) represents the parts per million (ppm) accuracy of the assignment. The colour corresponds to the presence of the observed sequence in horse myoglobin presented in Supplementary Figure 20.

	a			b			c			a-NH3		
**Description**	***m/z***	**Assignment**	**Dev. (ppm)**	***m/z***	**Assignment**	**Dev. (ppm)**	***m/z***	**Assignment**	**Dev. (ppm)**	***m/z***	**Assignment**	**Dev. (ppm)**
VW				310.1528	$C_{16}H_{21}N_3O_2Na^+$	0.6364	327.1792	$C_{16}H_{24}N_4O_2Na^+$	0.1928			
VWG	339.1791	$C_{17}H_{24}N_4O_2Na^+$	-0.1578	367.1742	$C_{18}H_{24}N_4O_3Na^+$	0.4089	384.2007	$C_{18}H_{27}N_5O_3Na^+$	0.1997			
VWGK	467.2746	$C_{23}H_{36}N_6O_3Na^+$	0.9783	495.2699	$C_{24}H_{36}N_6O_4Na^+$	1.7887	512.2959	$C_{24}H_{39}N_7O_4Na^+$	0.7300	450.2482	$C_{23}H_{33}N_5O_3Na^+$	1.3820
VWGKV	566.3428	$C_{28}H_{45}N_7O_4Na^+$	0.5699	723.3809	$C_{34}H_{52}N_8O_8Na^+$	1.1554						

Supplementary Table 43 Peak list exported from SurfaceLab spectrum of horse myoglobin, consisting of ions detected in the spectrum and assigned as sodium adducts of an internal fragment of the sequence LFTGHP. The *m/z* values represent the experimentally observed center mass of each peak. The deviation (dev.) represents the parts per million (ppm) accuracy of the assignment. The colour corresponds to the presence of the observed sequence in horse myoglobin presented in Supplementary Figure 20.

	a			b			c			a-NH3		
**Description**	***m/z***	**Assignment**	**Dev. (ppm)**	***m/z***	**Assignment**	**Dev. (ppm)**	***m/z***	**Assignment**	**Dev. (ppm)**	***m/z***	**Assignment**	**Dev. (ppm)**
LF										302.1840	$C_{15}H_{25}N_3O_2Na^+$	0.2121
LFT	358.2101	$C_{18}H_{29}N_3O_3Na^+$	0.0517				403.2316	$C_{19}H_{32}N_4O_4Na^+$	0.1294			
LFTG	415.2316	$C_{20}H_{32}N_4O_4Na^+$	-0.0437				460.2533	$C_{21}H_{35}N_5O_5Na^+$	0.5306	398.2047	$C_{20}H_{29}N_3O_4Na^+$	-0.8082
LFTGH							597.3125	$C_{27}H_{42}N_8O_6Na^+$	0.9228	535.2643	$C_{26}H_{36}N_6O_5Na^+$	0.6839
LFTGHP	649.3434	$C_{31}H_{46}N_8O_6Na^+$	0.2070	677.3388	$C_{32}H_{46}N_8O_7Na^+$	1.0070	694.3651	$C_{32}H_{49}N_9O_7Na^+$	0.5145	632.3158	$C_{31}H_{43}N_7O_6Na^+$	-1.3640

Trypsin

a) 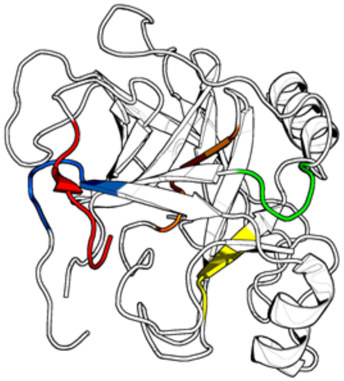

b) **Uniprot database code** TRYP_PIG1n

Amino acid sequence

IVGGYTCAANSIPYQVSLNSGSHFCGGSLINSQWVVSAAHCYKSRIQVRLGEHN
IDVLEGNEQFINAAKIITHPNFNGNTLDNDIMLIKLSSPATLNSRVATVSLPRSCAA
AGTECLISGWGNTKSSGSSYPSLLQCLKAPVLSDSSCKSSYPGQITGNMICVGF
LEGGKDSCQGDSGGPVVCNGQLQGIVSWGYGCAQKNKPGVYTKVCNYVNWI
QQTIAAN

Supplementary Figure 21 Pig trypsin (a) cartoon exported from PDB entry 1EPT[11] and (b) amino acid sequence exported from the UniProt database. The highlighted colours correspond to assigned segments of the amino acid sequence, presented in Supplementary Tables 44-48.

Supplementary Table 44 Peak list exported from SurfaceLab spectrum of pig trypsin, consisting of ions detected in the spectrum and assigned as fragments of N-terminal sequence IVGGYTC. The *m/z* values represent the experimentally observed center mass of each peak. The deviation (dev.) represents the parts per million (ppm) accuracy of the assignment. The colour corresponds to the presence of the observed sequence in pig trypsin presented in Supplementary Figure 21.

	a			b			c			a-NH3		
**Description**	***m/z***	**Assignment**	**Dev. (ppm)**	***m/z***	**Assignment**	**Dev. (ppm)**	***m/z***	**Assignment**	**Dev. (ppm)**	***m/z***	**Assignment**	**Dev. (ppm)**
IVG	264.1683	$C_{12}H_{23}N_3O_2Na^+$	0.3265	292.1633	$C_{13}H_{23}N_3O_3Na^+$	0.3966	309.1896	$C_{13}H_{26}N_4O_3Na^+$	-0.2805	247.1418	$C_{12}H_{20}N_2O_2Na^+$	0.5755
IVGG	321.1898	$C_{14}H_{26}N_4O_3Na^+$	0.1216							304.1634	$C_{14}H_{23}N_3O_3Na^+$	0.7677
IVGGY	484.2529	$C_{23}H_{35}N_5O_5Na^+$	-0.2232	512.2478	$C_{24}H_{35}N_5O_6Na^+$	-0.3810	529.2746	$C_{24}H_{38}N_6O_6Na^+$	0.2378			
IVGGYT	585.3009	$C_{27}H_{42}N_6O_7Na^+$	0.3093				630.3220	$C_{28}H_{45}N_7O_8Na^+$	-0.2964			
IVGGYTC-S	656.3379	$C_{30}H_{47}N_7O_8Na^+$	0.1048									

Supplementary Table 45 Peak list exported from SurfaceLab spectrum of pig trypsin, consisting of ions detected in the spectrum and assigned as sodium adducts of an internal fragment of the sequence AAAGT. The *m/z* values represent the experimentally observed center mass of each peak. The deviation (dev.) represents the parts per million (ppm) accuracy of the assignment. The colour corresponds to the presence of the observed sequence in pig trypsin presented in Supplementary Figure 21.

	a			b			c			a-NH3		
**Description**	***m/z***	**Assignment**	**Dev. (ppm)**	***m/z***	**Assignment**	**Dev. (ppm)**	***m/z***	**Assignment**	**Dev. (ppm)**	***m/z***	**Assignment**	**Dev. (ppm)**
AA				167.0789	$C_6H_{12}N_2O_2Na^+$	-1.1700						
AAA	210.1213	$C_8H_{17}N_3O_2Na^+$	-0.0218	238.1162	$C_9H_{17}N_3O_3Na^+$	0.0514				193.0947	$C_8H_{14}N_2O_2Na^+$	-0.4572
AAAG	267.1427	$C_{10}H_{20}N_4O_3Na^+$	-0.2171							250.1162	$C_{10}H_{17}N_3O_3Na^+$	0.1260
AAAGT				396.1856	$C_{15}H_{27}N_5O_6Na^+$	0.7355				351.1636	$C_{14}H_{24}N_4O_5Na^+$	-0.7484

Supplementary Table 46 Peak list exported from SurfaceLab spectrum of pig trypsin, consisting of ions detected in the spectrum and assigned as sodium adducts of an internal fragment of the sequence PSLLQC. The *m/z* values represent the experimentally observed center mass of each peak. The deviation (dev.) represents the parts per million (ppm) accuracy of the assignment. The colour corresponds to the presence of the observed sequence in pig trypsin presented in Supplementary Figure 21.

	a			b			c			a-NH3		
**Description**	***m/z***	**Assignment**	**Dev. (ppm)**	***m/z***	**Assignment**	**Dev. (ppm)**	***m/z***	**Assignment**	**Dev. (ppm)**	***m/z***	**Assignment**	**Dev. (ppm)**
PS	181.0945	$C_7H_{14}N_2O_2Na^+$	-1.1144	209.0895	$C_8H_{14}N_2O_3Na^+$	-0.5592	226.1162	$C_8H_{17}N_3O_3Na^+$	-0.0078	164.0681	$C_7H_{11}NO_2Na^+$	-0.8012
PSL	294.1791	$C_{13}H_{25}N_3O_3Na^+$	0.8881	322.1739	$C_{14}H_{25}N_3O_4Na^+$	0.5920	339.2006	$C_{14}H_{28}N_4O_4Na^+$	1.0410	277.1525	$C_{13}H_{22}N_2O_3Na^+$	0.9425
PSLL	407.2630	$C_{19}H_{36}N_4O_4Na^+$	0.3552	435.2581	$C_{20}H_{36}N_4O_5Na^+$	0.6145	452.2847	$C_{20}H_{39}N_5O_5Na^+$	0.8586	390.2364	$C_{19}H_{33}N_3O_4Na^+$	0.0900
PSLLQ	535.3219	$C_{24}H_{44}N_6O_6Na^+$	0.9165							518.2953	$C_{24}H_{41}N_5O_6Na^+$	0.8536
PSLLQC	638.3310	$C_{27}H_{49}N_7O_7SNa^+$	0.5620									

Supplementary Table 47 Peak list exported from SurfaceLab spectrum of pig trypsin, consisting of ions detected in the spectrum and assigned as sodium adducts of an internal fragment of the sequence SGGPVVC. The *m/z* values represent the experimentally observed center mass of each peak. The deviation (dev.) represents the parts per million (ppm) accuracy of the assignment. The colour corresponds to the presence of the observed sequence in pig trypsin presented in Supplementary Figure 21.

	a			b			c			a-NH3		
**Description**	***m/z***	**Assignment**	**Dev. (ppm)**	***m/z***	**Assignment**	**Dev. (ppm)**	***m/z***	**Assignment**	**Dev. (ppm)**	***m/z***	**Assignment**	**Dev. (ppm)**
SGG				226.0798	$C_7H_{13}N_3O_4Na^+$	-0.2264				181.0580	$C_6H_{10}N_2O_3Na^+$	-1.7463
SGGP	295.1377	$C_{11}H_{20}N_4O_4Na^+$	-0.0394	323.1326	$C_{12}H_{20}N_4O_5Na^+$	-0.0744				278.1113	$C_{11}H_{17}N_3O_4Na^+$	0.6162
SGGPV	394.2064	$C_{16}H_{29}N_5O_5Na^+$	0.7242	422.2012	$C_{17}H_{29}N_5O_6Na^+$	0.4870						
SGGPVV	493.2748	$C_{21}H_{38}N_6O_6Na^+$	0.6960	521.2701	$C_{22}H_{38}N_6O_7Na^+$	1.2405				476.2484	$C_{21}H_{35}N_5O_6Na^+$	0.8996
SGGPVVC-S	564.3129	$C_{24}H_{43}N_7O_7Na^+$	2.3484	592.3071	$C_{25}H_{43}N_7O_8Na^+$	0.9875				547.2861	$C_{24}H_{40}N_6O_7Na^+$	1.8497
SGGPVVC-SH_2	562.2964	$C_{24}H_{41}N_7O_7Na^+$	0.7667	590.2922	$C_{25}H_{41}N_7O_8Na^+$	2.2846						

Supplementary Table 48 Peak list exported from SurfaceLab spectrum of pig trypsin, consisting of ions detected in the spectrum and assigned as sodium adducts of an internal fragment of the sequence PGVY. The *m/z* values represent the experimentally observed center mass of each peak. The deviation (dev.) represents the parts per million (ppm) accuracy of the assignment. The colour corresponds to the presence of the observed sequence in pig trypsin presented in Supplementary Figure 21.

	a			b			c			a-NH3		
**Description**	***m/z***	**Assignment**	**Dev. (ppm)**	***m/z***	**Assignment**	**Dev. (ppm)**	***m/z***	**Assignment**	**Dev. (ppm)**	***m/z***	**Assignment**	**Dev. (ppm)**
VY				287.1370	$C_{14}H_{20}N_2O_3Na^+$	1.2197						
GVY	316.1636	$C_{15}H_{23}N_3O_3Na^+$	1.4706	344.1583	$C_{16}H_{23}N_3O_4Na^+$	0.6103						
PGVY	413.2160	$C_{20}H_{30}N_4O_4Na^+$	0.1276	441.2107	$C_{21}H_{30}N_4O_5Na^+$	-0.2638	458.2376	$C_{21}H_{33}N_5O_5Na^+$	0.3540			

Pepsin

a) 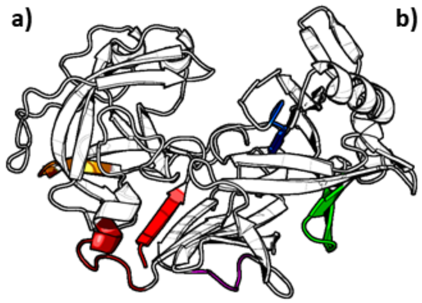

b)

Uniprot database code	Amino acid sequence
PEPA_PIG1n	IGDEPLENYLDTEYFGTIGIGTPAQDFTVIFDTGSSNLWVPSVYCSSLACSDHNQ<br>FNPDDSSTFEATSQELSITYGTGSMTGILGYDTVQVGGISDTNQIFGLSETEPGS<br>FLYYAPFDGILGLAYPSISASGATPVFDNLWDQGLVSQDLFSVYLSSNDDSGSV<br>VLLGGIDSSYYTGSLNWVPVSVEGYWQITLDSITMDGETIACSGGCQAIVDTGT<br>SLLTGPTSAIANIQSDIGASENSDGEMVISCSSIDSLPDIVFTINGVQYPLSPSAYIL<br>QDDDSCTSGFEGMDVPTSSGELWILGDVFIRQYYTVFDRANNKVGLAPVA

Supplementary Figure 22 Pig pepsin (a) cartoon exported from PDB entry 4PEP[12] and (b) amino acid sequence exported from the UniProt database. The highlighted colours correspond to assigned segments of the amino acid sequence, presented in Supplementary Tables 49-57.

Supplementary Table 49 Peak list exported from SurfaceLab spectrum of pig pepsin, consisting of ions detected in the spectrum and assigned fragments of N-terminal sequence IGDEP. The *m/z* values represent the experimentally observed center mass of each peak. The deviation (dev.) represents the parts per million (ppm) accuracy of the assignment. The colour corresponds to the presence of the observed sequence in pig pepsin presented in Supplementary Figure 22.

	a			b			c			a-NH3		
**Description**	***m/z***	**Assignment**	**Dev. (ppm)**	***m/z***	**Assignment**	**Dev. (ppm)**	***m/z***	**Assignment**	**Dev. (ppm)**	***m/z***	**Assignment**	**Dev. (ppm)**
IG	165.0994	$C_7H_{14}N_2ONa^+$	-2.3885	193.0946	$C_8H_{14}N_2O_2Na^+$	-1.0057	210.1212	$C_8H_{17}N_3O_2Na^+$	-0.5850			
IGD	280.1267	$C_{11}H_{19}N_3O_4Na^+$	-0.3044	308.1215	$C_{12}H_{19}N_3O_5Na^+$	-0.4683	325.1481	$C_{12}H_{22}N_4O_5Na^+$	-0.3805			
IGDE	409.1692	$C_{16}H_{26}N_4O_7Na^+$	-0.3207									
IGDEP	506.2220	$C_{21}H_{33}N_5O_8Na^+$	-0.2380									

Supplementary Table 50 Peak list exported from SurfaceLab spectrum of pig pepsin, consisting of ions detected in the spectrum and assigned as sodium adducts of an internal fragment of the sequence FTINGVQ. The *m/z* values represent the experimentally observed center mass of each peak. The deviation (dev.) represents the parts per million (ppm) accuracy of the assignment. The colour corresponds to the presence of the observed sequence in pig pepsin presented in Supplementary Figure 22.

	a			b			c			a-NH3		
**Description**	***m/z***	**Assignment**	**Dev. (ppm)**	***m/z***	**Assignment**	**Dev. (ppm)**	***m/z***	**Assignment**	**Dev. (ppm)**	***m/z***	**Assignment**	**Dev. (ppm)**
FTI	358.2101	$C_{18}H_{29}N_3O_3Na^+$	-0.0796				403.2314	$C_{19}H_{32}N_4O_4Na^+$	-0.4723			
FTIN	472.2530	$C_{22}H_{35}N_5O_5Na^+$	-0.1712				517.2744	$C_{23}H_{38}N_6O_6Na^+$	-0.2388	455.2265	$C_{22}H_{32}N_4O_5Na^+$	-0.0522
FTING	529.2744	$C_{24}H_{38}N_6O_6Na^+$	-0.1150	557.2681	$C_{25}H_{38}N_6O_7Na^+$	-2.3935	574.2955	$C_{25}H_{41}N_7O_7Na^+$	-0.8153	512.2479	$C_{24}H_{35}N_5O_6Na^+$	-0.0871
FTINGV	628.3427	$C_{29}H_{47}N_7O_7Na^+$	-0.2762							611.3163	$C_{29}H_{44}N_6O_7Na^+$	-0.1727
FTINGVQ										739.3746	$C_{34}H_{52}N_8O_9Na^+$	-0.4855

Supplementary Table 51 Peak list exported from SurfaceLab spectrum of pig pepsin, consisting of ions detected in the spectrum and assigned as sodium adducts of an internal fragment of the sequence GVQYPLS. The *m/z* values represent the experimentally observed center mass of each peak. The deviation (dev.) represents the parts per million (ppm) accuracy of the assignment. The colour corresponds to the presence of the observed sequence in pig pepsin presented in Supplementary Figure 22.

	a			b			c			a-NH3		
**Description**	***m/z***	**Assignment**	**Dev. (ppm)**	***m/z***	**Assignment**	**Dev. (ppm)**	***m/z***	**Assignment**	**Dev. (ppm)**	***m/z***	**Assignment**	**Dev. (ppm)**
GVQY	444.2215	$C_{20}H_{31}N_5O_5Na^+$	-0.4745	472.2166	$C_{21}H_{31}N_5O_6Na^+$	-0.1410	489.2429	$C_{21}H_{34}N_6O_6Na^+$	-0.6302	427.1946	$C_{20}H_{28}N_4O_5Na^+$	-1.4555
GVQYP	541.2744	$C_{25}H_{38}N_6O_6Na^+$	-0.1867	569.2695	$C_{26}H_{38}N_6O_7Na^+$	0.0889				524.2477	$C_{25}H_{35}N_5O_6Na^+$	-0.4801
GVQYPL				682.3533	$C_{32}H_{49}N_7O_8Na^+$	-0.2060				637.3318	$C_{31}H_{46}N_6O_7Na^+$	-0.3576
GVQYPLS										724.3633	$C_{34}H_{51}N_7O_9Na^+$	-1.0942

Supplementary Table 52 Peak list exported from SurfaceLab spectrum of pig pepsin, consisting of ions detected in the spectrum and assigned as sodium adducts of an internal fragment of the sequence GELWIL. The *m/z* values represent the experimentally observed center mass of each peak. The deviation (dev.) represents the parts per million (ppm) accuracy of the assignment. The colour corresponds to the presence of the observed sequence in pig pepsin presented in Supplementary Figure 22.

	a			b			c			a-NH3		
**Description**	***m/z***	**Assignment**	**Dev. (ppm)**	***m/z***	**Assignment**	**Dev. (ppm)**	***m/z***	**Assignment**	**Dev. (ppm)**	***m/z***	**Assignment**	**Dev. (ppm)**
GEL	296.1579	$C_{12}H_{23}N_3O_4Na^+$	-0.6758	324.1529	$C_{13}H_{23}N_3O_5Na^+$	-0.3319						
GELW	482.2370	$C_{23}H_{33}N_5O_5Na^+$	-0.7269	510.2322	$C_{24}H_{33}N_5O_6Na^+$	-0.2057						
GELWI	595.3212	$C_{29}H_{44}N_6O_6Na^+$	-0.4500	623.3160	$C_{30}H_{44}N_6O_7Na^+$	-0.5862						
GELWIL				736.4001	$C_{36}H_{55}N_7O_8Na^+$	-0.4737						

Supplementary Table 53 Peak list exported from SurfaceLab spectrum of pig pepsin, consisting of ions detected in the spectrum and assigned as sodium adducts of an internal fragment of the sequence SGELWILG. The *m/z* values represent the experimentally observed center mass of each peak. The deviation (dev.) represents the parts per million (ppm) accuracy of the assignment. The colour corresponds to the presence of the observed sequence in pig pepsin presented in Supplementary Figure 22.

	a			b			c			a-NH3		
**Description**	***m/z***	**Assignment**	**Dev. (ppm)**	***m/z***	**Assignment**	**Dev. (ppm)**	***m/z***	**Assignment**	**Dev. (ppm)**	***m/z***	**Assignment**	**Dev. (ppm)**
SGEL										366.1631	$C_{15}H_{25}N_3O_6Na^+$	-1.2603
SGELW	569.2695	$C_{26}H_{38}N_6O_7Na^+$	0.0889									
SGELWI	682.3533	$C_{32}H_{49}N_7O_8Na^+$	-0.2060									
SGELWI				710.3477	$C_{33}H_{49}N_7O_9Na^+$	-1.0141						
SGELWIL				823.4328	$C_{39}H_{60}N_8O_{10}Na^+$	0.4637						
SGELWILG	852.4577	$C_{40}H_{63}N_9O_{10}Na^+$	-1.5466									

Supplementary Table 54 Peak list exported from SurfaceLab spectrum of pig pepsin, consisting of ions detected in the spectrum and assigned as sodium adducts of an internal fragment of the sequence GIGTP. The *m/z* values represent the experimentally observed center mass of each peak. The deviation (dev.) represents the parts per million (ppm) accuracy of the assignment. The colour corresponds to the presence of the observed sequence in pig pepsin presented in Supplementary Figure 22.

	a			b			c			a-NH3		
**Description**	***m/z***	**Assignment**	**Dev. (ppm)**	***m/z***	**Assignment**	**Dev. (ppm)**	***m/z***	**Assignment**	**Dev. (ppm)**	***m/z***	**Assignment**	**Dev. (ppm)**
GIG	224.1369	$C_9H_{19}N_3O_2Na^+$	-0.2379	252.1317	$C_{10}H_{19}N_3O_3Na^+$	-0.8401				207.1103	$C_9H_{16}N_2O_2Na^+$	-0.5094
GIGT	325.1845	$C_{13}H_{26}N_4O_4Na^+$	-0.3684	353.1794	$C_{14}H_{26}N_4O_5Na^+$	-0.4454				308.1579	$C_{13}H_{23}N_3O_4Na^+$	-0.4783
GIGTP				521.2694	$C_{22}H_{38}N_6O_7Na^+$	-0.0581				476.2479	$C_{21}H_{35}N_5O_6Na^+$	-0.1287

Supplementary Table 55 Peak list exported from SurfaceLab spectrum of pig pepsin, consisting of ions detected in the spectrum and assigned as sodium adducts of an internal fragment of the sequence IGIGTPA. The *m/z* values represent the experimentally observed center mass of each peak. The deviation (dev.) represents the parts per million (ppm) accuracy of the assignment. The colour corresponds to the presence of the observed sequence in pig pepsin presented in Supplementary Figure 22.

	a			b			c			a-NH3		
**Description**	***m/z***	**Assignment**	**Dev. (ppm)**	***m/z***	**Assignment**	**Dev. (ppm)**	***m/z***	**Assignment**	**Dev. (ppm)**	***m/z***	**Assignment**	**Dev. (ppm)**
IGI				308.1943	$C_{14}H_{27}N_3O_3Na^+$	-0.4573						
IGIG	337.2209	$C_{15}H_{30}N_4O_3Na^+$	-0.2966									
IGIGT	438.2685	$C_{19}H_{37}N_5O_5Na^+$	-0.4471									
IGIGTP	563.3166	$C_{25}H_{44}N_6O_7Na^+$	0.3476									
IGIGTPA	606.3581	$C_{27}H_{49}N_7O_7Na^+$	-0.7115									

Supplementary Table 56 Peak list exported from SurfaceLab spectrum of pig pepsin, consisting of ions detected in the spectrum and assigned as sodium adducts of an internal fragment of the sequence LWDQGLVS. The *m/z* values represent the experimentally observed center mass of each peak. The deviation (dev.) represents the parts per million (ppm) accuracy of the assignment. The colour corresponds to the presence of the observed sequence in pig pepsin presented in Supplementary Figure 22.

	a			b			c			a-NH3		
**Description**	***m/z***	**Assignment**	**Dev. (ppm)**	***m/z***	**Assignment**	**Dev. (ppm)**	***m/z***	**Assignment**	**Dev. (ppm)**	***m/z***	**Assignment**	**Dev. (ppm)**
LWD	411.2004	$C_{20}H_{28}N_4O_4Na^+$	0.2941	439.1945	$C_{21}H_{28}N_4O_5Na^+$	-1.4922	456.2213	$C_{21}H_{31}N_5O_5Na^+$	-1.0124			
LWDQ	539.2588	$C_{25}H_{36}N_6O_6Na^+$	-0.1019	567.2534	$C_{26}H_{36}N_6O_7Na^+$	-0.6632	584.2797	$C_{26}H_{39}N_7O_7Na^+$	-1.0883	522.2323	$C_{25}H_{33}N_5O_6Na^+$	0.0288
LWDQG	596.2801	$C_{27}H_{39}N_7O_7Na^+$	-0.3297	624.2750	$C_{28}H_{39}N_7O_8Na^+$	-0.3304				579.2535	$C_{27}H_{36}N_6O_7Na^+$	-0.4212
LWDQGL	709.3642	$C_{33}H_{50}N_8O_8Na^+$	-0.2253	737.3591	$C_{34}H_{50}N_8O_9Na^+$	-0.2818				692.3352	$C_{33}H_{47}N_7O_8Na^+$	-3.7912
LWDQGLV	808.4330	$C_{38}H_{59}N_9O_9Na^+$	0.2801	836.4280	$C_{39}H_{59}N_9O_{10}Na^+$	0.3535				791.4039	$C_{38}H_{56}N_8O_9Na^+$	-3.0284
LWDQGLVS	895.4621	$C_{41}H_{64}N_{10}O_{11}Na^+$	-3.0376							878.4365	$C_{41}H_{61}N_9O_{11}Na^+$	-2.0323

Supplementary Table 57 Peak list exported from SurfaceLab spectrum of pig pepsin, consisting of ions detected in the spectrum and assigned as sodium adducts of an internal fragment of the sequence IDSSY. The *m/z* values represent the experimentally observed center mass of each peak. The deviation (dev.) represents the parts per million (ppm) accuracy of the assignment. The colour corresponds to the presence of the observed sequence in pig pepsin presented in Supplementary Figure 22.

	a			b			c			a-NH3		
**Description**	***m/z***	**Assignment**	**Dev. (ppm)**	***m/z***	**Assignment**	**Dev. (ppm)**	***m/z***	**Assignment**	**Dev. (ppm)**	***m/z***	**Assignment**	**Dev. (ppm)**
SY	247.1053	$C_{11}H_{16}N_2O_3Na^+$	-0.2308	275.1003	$C_{12}H_{16}N_2O_4Na^+$	0.2358						
SSY	334.1372	$C_{14}H_{21}N_3O_5Na^+$	-0.4438	362.1321	$C_{15}H_{21}N_3O_6Na^+$	-0.3984						
DSSY	449.1641	$C_{18}H_{26}N_4O_8Na^+$	-0.4061									
IDSSY	562.2481	$C_{24}H_{37}N_5O_9Na^+$	-0.4906									

Transferrin

Human transferrin (UniProt ID TRFE_HUMAN1n) amino acid sequence exported from the UniProt database. The highlighted colours correspond to assigned segments of the amino acid sequence, presented in Supplementary Tables 58-62.

VPDKTVRWCAVSEHEATKCQSFRDHMKSVIPSDGPSVACVKKASYLDCIRAIAANEADAVTLDAGLVYDAYLAPNNLKPVVAEFYGSKEDPQTFY
YAVAVVKKDSGFQMNQLRGKKSCHTGLGRSAGWNIPIGLLYCDLPEPRKPLEKAVANFFSGSCAPCADGTDFPQLCQLCPGCGCSTLNQYFGYSG
AFKCLKDGAGDVAFVKHSTIFENLANKADRDQYELLCLDNTRKPVDEYKDCHLAQVPSHTVVARSMGGKEDLIWELLNQAQEHFGKDKSKEFQL
FSSPHGKDLLFKDSAHGFLKVPPRMDAKMYLGYEYVTAIRNLREGTCPEAPTDECKPVKWCALSHHERLKCDEWSVNSVGKIECVSAETTEDCIAK
IMNGEADAMSLDGGFVYIAGKCGLVPVLAENYNKSDNCEDTPEAGYFAIAVVKKSASDLTWDNLKGKKSCHTAVGRTAGWNIPMGLLYNKINHC
RFDEFFSEGCAPGSKKDSSLCKLCMGSGLNLCEPNNKEGYYGYTGAFRCLVEKGDVAFVKHQTVPQNTGGKNPDPWAKNLNEKDYELLCLDGTR
KPVEEYANCHLARAPNHAVVTRKDKEACVHKILRQQQHLFGSNVTDCSGNFCLFRSETKDLLFRDDTVCLAKLHDRNTYEKYLGEEYVKAVGNL
RKCSTSSLLEACTFRRP

Supplementary Table 58 Peak list exported from SurfaceLab spectrum of human transferrin, consisting of ions detected in the spectrum and assigned as sodium adducts of an internal fragment of the sequence HTGLGR. The *m/z* values represent the experimentally observed center mass of each peak. The deviation (dev.) represents the parts per million (ppm) accuracy of the assignment.

	a			b			b-NH3			a-NH3		
**Description**	***m/z***	**Assignment**	**Dev. (ppm)**	***m/z***	**Assignment**	**Dev. (ppm)**	***m/z***	**Assignment**	**Dev. (ppm)**	***m/z***	**Assignment**	**Dev. (ppm)**
HTG				320.1331	$C_{12}H_{19}N_5O_4Na^+$	0.4050	303.1066	$C_{12}H_{16}N_4O_4Na^+$	0.5778	275.1113	$C_{11}H_{16}N_4O_3Na^+$	-0.5926
HTGL	405.2225	$C_{17}H_{30}N_6O_4Na^+$	1.0826	433.2173	$C_{18}H_{30}N_6O_5Na^+$	0.6607	416.1905	$C_{18}H_{27}N_5O_5Na^+$	0.2089	388.1958	$C_{17}H_{27}N_5O_4Na^+$	0.5834
HTGLG	462.2439	$C_{19}H_{33}N_7O_5Na^+$	0.7765	490.2387	$C_{20}H_{33}N_7O_6Na^+$	0.5547	473.2127	$C_{20}H_{30}N_6O_6Na^+$	1.6851	445.2170	$C_{19}H_{30}N_6O_5Na^+$	0.1151
HTGLGR	618.3442	$C_{25}H_{45}N_{11}O_6Na^+$	-0.6825									

Supplementary Table 59 Peak list exported from SurfaceLab spectrum of human transferrin, consisting of ions detected in the spectrum and assigned as fragments of internal sequence HTAVGR. The *m/z* values represent the experimentally observed center mass of each peak. The deviation (dev.) represents the parts per million (ppm) accuracy of the assignment.

	a			b			b-NH3			a-NH3		
**Description**	**m/z**	**Assignment**	**Dev. (ppm)**	**m/z**	**Assignment**	**Dev. (ppm)**	**m/z**	**Assignment**	**Dev. (ppm)**	**m/z**	**Assignment**	**Dev. (ppm)**
HT	235.1165	$C_9H_{16}N_4O_2Na^+$	-0.1540	263.1116	$C_{10}H_{16}N_4O_3Na^+$	0.5125	246.0850	$C_{10}H_{13}N_3O_3Na^+$	0.3758	218.0901	$C_9H_{13}N_3O_2Na^+$	0.6149
HTA				334.1487	$C_{13}H_{21}N_5O_4Na^+$	0.2831	317.1218	$C_{13}H_{18}N_4O_4Na^+$	-0.8349	289.1271	$C_{12}H_{18}N_4O_3Na^+$	0.0752
HTAV	405.2225	$C_{17}H_{30}N_6O_4Na^+$	1.0826	433.2173	$C_{18}H_{30}N_6O_5Na^+$	0.6607	416.1905	$C_{18}H_{27}N_5O_5Na^+$	0.2089	388.1958	$C_{17}H_{27}N_5O_4Na^+$	0.5834
HTAVG	462.2439	$C_{19}H_{33}N_7O_5Na^+$	0.7765	490.2387	$C_{20}H_{33}N_7O_6Na^+$	0.5547	473.2127	$C_{20}H_{30}N_6O_6Na^+$	1.6851	445.2170	$C_{19}H_{30}N_6O_5Na^+$	0.1151
HTAVGR	618.3442	$C_{25}H_{45}N_{11}O_6Na^+$	-0.6825									

Supplementary Table 60 Peak list exported from SurfaceLab spectrum of human transferrin, consisting of ions detected in the spectrum and assigned as sodium adducts of an internal fragment of the sequence NLKPVVA. The *m/z* values represent the experimentally observed center mass of each peak. The deviation (dev.) represents the parts per million (ppm) accuracy of the assignment.

	a			b			b-NH3			a-NH3		
**Description**	***m/z***	**Assignment**	**Dev. (ppm)**	***m/z***	**Assignment**	**Dev. (ppm)**	***m/z***	**Assignment**	**Dev. (ppm)**	***m/z***	**Assignment**	**Dev. (ppm)**
NL	224.1370	$C_9H_{19}N_3O_2Na^+$	0.2510	252.1320	$C_{10}H_{19}N_3O_3Na^+$	0.4716	235.1055	$C_{10}H_{16}N_2O_3Na^+$	0.6518	207.1104	$C_9H_{16}N_2O_2Na^+$	-0.0853
NLK				380.2269	$C_{16}H_{31}N_5O_4Na^+$	0.2227	363.2004	$C_{16}H_{28}N_4O_4Na^+$	0.2474	335.2055	$C_{15}H_{28}N_4O_3Na^+$	0.2807
NLKP	449.2846	$C_{20}H_{38}N_6O_4Na^+$	-0.2158	477.2799	$C_{21}H_{38}N_6O_5Na^+$	0.5901				432.2583	$C_{20}H_{35}N_5O_4Na^+$	0.4458
NLKPV				576.3486	$C_{26}H_{47}N_7O_6Na^+$	1.0944	559.3214	$C_{26}H_{44}N_6O_6Na^+$	-0.1191	531.3268	$C_{25}H_{44}N_6O_5Na^+$	0.5550
NLKPVV	647.4230	$C_{30}H_{56}N_8O_6Na^+$	2.3348	675.4188	$C_{31}H_{56}N_8O_7Na^+$	3.5560				630.3950	$C_{30}H_{53}N_7O_6Na^+$	0.0128
NLKPVVA										701.4340	$C_{33}H_{58}N_8O_7Na^+$	2.8014

Supplementary Table 61 Peak list exported from SurfaceLab spectrum of human transferrin, consisting of ions detected in the spectrum and assigned as sodium adducts of an internal fragment of the sequence PNHAVV. The *m/z* values represent the experimentally observed center mass of each peak. The deviation (dev.) represents the parts per million (ppm) accuracy of the assignment.

	a			b			b-NH3			a-NH3		
**Description**	***m/z***	**Assignment**	**Dev. (ppm)**	***m/z***	**Assignment**	**Dev. (ppm)**	***m/z***	**Assignment**	**Dev. (ppm)**	***m/z***	**Assignment**	**Dev. (ppm)**
PNH	345.1647	$C_{14}H_{22}N_6O_3Na^+$	0.3725	373.1595	$C_{15}H_{22}N_6O_4Na^+$	0.1089				328.1379	$C_{14}H_{19}N_5O_3Na^+$	-0.3311
PNHA	416.2015	$C_{17}H_{27}N_7O_4Na^+$	-0.4128	444.1972	$C_{18}H_{27}N_7O_5Na^+$	1.4104	427.1704	$C_{18}H_{24}N_6O_5Na^+$	0.7375	399.1753	$C_{17}H_{24}N_6O_4Na^+$	0.4198
PNHAV	515.2704	$C_{22}H_{36}N_8O_5Na^+$	0.5302	543.2652	$C_{23}H_{36}N_8O_6Na^+$	0.3930				498.2437	$C_{22}H_{33}N_7O_5Na^+$	0.2264
PNHAVV							625.3051	$C_{28}H_{42}N_8O_7Na^+$	-2.8477	597.3117	$C_{27}H_{42}N_8O_6Na^+$	-0.4364

Supplementary Table 62 Peak list exported from SurfaceLab spectrum of human transferrin, consisting of ions detected in the spectrum and assigned as sodium adducts of an internal fragment of the sequence VPQN. The *m/z* values represent the experimentally observed center mass of each peak. The deviation (dev.) represents the parts per million (ppm) accuracy of the assignment.

	a			b			c			a-NH3		
**Description**	***m/z***	**Assignment**	**Dev. (ppm)**	***m/z***	**Assignment**	**Dev. (ppm)**	***m/z***	**Assignment**	**Dev. (ppm)**	***m/z***	**Assignment**	**Dev. (ppm)**
VP				221.1262	$C_{10}H_{18}N_2O_2Na^+$	0.5184	238.1527	$C_{10}H_{21}N_3O_2Na^+$	0.3094			
VPQ	321.1898	$C_{14}H_{26}N_4O_3Na^+$	0.3913	349.1847	$C_{15}H_{26}N_4O_4Na^+$	0.3021	366.2112	$C_{15}H_{29}N_5O_4Na^+$	0.1868	304.1632	$C_{14}H_{23}N_3O_3Na^+$	0.2406
VPQN	435.2330	$C_{18}H_{32}N_6O_5Na^+$	0.7826	463.2278	$C_{19}H_{32}N_6O_6Na^+$	0.5347	480.2540	$C_{19}H_{35}N_7O_6Na^+$	-0.2633	418.2061	$C_{18}H_{29}N_5O_5Na^+$	0.0944

Chymotrypsin α

a)

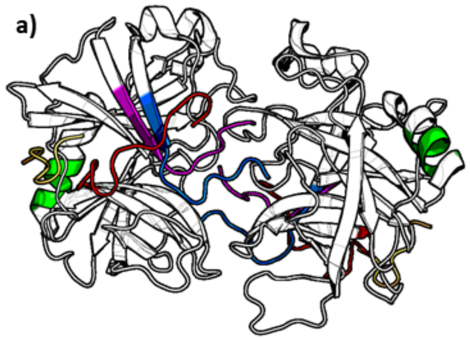

b)

Uniprot database code	Amino acid sequence
CTRA_BOVIN1n	CGVPAIQPVLSGL
CTRA_BOVIN2n	IVNGEEAVPGSWPWQVSLQDKTGFHFCGGSLINENWVVTAAHCGVTTSDVVVAGEFDQGSSSEKIQKLKIAKVFKNSKYNSLTINNDITLLKLSTAASFSQTVSAVCLPSASDDFAAGTTCVTTGWGLTRY
CTRA_BOVIN3n	ANTPDRLQQASLPLLSNTNCKKYWGTKIKDAMICAGASGVSSCMGDSGGPLVCKKNGAWTLVGIVSWGSSTCSTSTPGVYARVTALVNWVQQTLAAN

Supplementary Figure 23 Bovine α-chymotrypsin (a) cartoon exported from PDB entry 1AB9[13] and (b) amino acid sequence exported from the UniProt database. The highlighted colours correspond to assigned segments of the amino acid sequence, presented in Supplementary Tables 63-67.

Supplementary Table 63 Peak list exported from SurfaceLab spectrum of α-chymotrypsin from bovine liver, consisting of ions detected in the spectrum and assigned as sodium adducts of internal fragments of the sequence of the A chain of the protein, IQPVLSG. The *m/z* values represent the experimentally observed center mass of each peak. The deviation (dev.) represents the parts per million (ppm) accuracy of the assignment. The colour corresponds to the presence of the observed sequence in α-chymotrypsin presented in Supplementary Figure 23.

	ya			yb			yc			ya - NH_3		
**Description**	***m/z***	**Assignment**	**Dev. (ppm)**	***m/z***	**Assignment**	**Dev. (ppm)**	***m/z***	**Assignment**	**Dev. (ppm)**	***m/z***	**Assignment**	**Dev. (ppm)**
IQPV	432.2580	$C_{20}H_{35}N_5O_4Na^+$	-0.3296	460.2530	$C_{21}H_{35}N_5O_5Na^+$	-0.1913	477.2796	$C_{21}H_{38}N_6O_5Na^+$	0.0613	415.2314	$C_{20}H_{32}N_4O_4Na^+$	-0.5261
IQPVL	545.3421	$C_{26}H_{46}N_6O_5Na^+$	-0.0825	573.3369	$C_{27}H_{46}N_6O_6Na^+$	-0.2980	590.3640	$C_{27}H_{49}N_7O_6Na^+$	0.5257	x	x	x
IQPVLS	632.3745	$C_{29}H_{51}N_7O_7Na^+$	0.5049	660.3695	$C_{30}H_{51}N_7O_8Na^+$	0.5169	x	x	x	x	x	x
IQPVLSG	689.3963	$C_{31}H_{54}N_8O_8Na^+$	0.8434	717.3910	$C_{32}H_{54}N_8O_9Na^+$	0.5633	x	x	x	x	x	x

Supplementary Table 64 Peak list exported from SurfaceLab spectrum of α-chymotrypsin from bovine liver, consisting of ions detected in the spectrum and assigned as C-terminal sequence of the A chain GVPAIQPVLSGL and a full A chain CGVPAIQPVLSGL. The sequence is observed as y ions. The *m/z* values represent the experimentally observed center mass of each peak. The colour corresponds to the presence of the observed sequence in α-chymotrypsin presented in Supplementary Figure 23.

Description	*m/z*	Assignment	Dev. (ppm)
SGL	298.1374	$C_{11}H_{21}N_3O_5Na^+$	0.1814
LSGL	411.2216	$C_{17}H_{32}N_4O_6Na^+$	0.3605
PVLSGL	607.3429	$C_{27}H_{48}N_6O_8Na^+$	0.4458
IQPVLSGL	848.4858	$C_{38}H_{67}N_9O_{11}Na^+$	0.6353
PAIQPVLSGL	1016.5762	$C_{46}H_{79}N_{11}O_{13}Na^+$	1.1239
GVPAIQPVLSGL	1172.6668	$C_{53}H_{91}N_{13}O_{15}Na^+$	1.5263
CGVPAIQPVLSGL	1275.6733	$C_{56}H_{96}N_{14}O_{16}SNa^+$	-0.6437

Supplementary Table 65 Peak list exported from SurfaceLab spectrum of α-chymotrypsin from bovine liver, consisting of ions detected in the spectrum and assigned as N-terminal sequence of the B chain IVNGEEAVPGSWPW. The sequence is observed as a, b, and c and ions. The *m/z* values represent the experimentally observed center mass of each peak. The deviation (dev.) represents the parts per million (ppm) accuracy of the assignment. The colour corresponds to the presence of the observed sequence in α-chymotrypsin presented in Supplementary Figure 23.

	a			b			c		
**Description**	***m/z***	**Assignment**	**Dev. (ppm)**	***m/z***	**Assignment**	**Dev. (ppm)**	***m/z***	**Assignment**	**Dev. (ppm)**
IVNGE	507.2538	$C_{21}H_{36}N_6O_7Na^+$	0.1435	535.2488	$C_{22}H_{36}N_6O_8Na^+$	0.2665	552.2755	$C_{22}H_{39}N_7O_8Na^+$	0.4851
IVNGEE	636.2966	$C_{26}H_{43}N_7O_{10}Na^+$	0.4378	664.2917	$C_{27}H_{43}N_7O_{11}Na^+$	0.6304	681.3182	$C_{27}H_{46}N_8O_{11}Na^+$	0.6121
IVNGEEA	707.3340	$C_{29}H_{48}N_8O_{11}Na^+$	0.8109	735.3279	$C_{30}H_{48}N_8O_{12}Na^+$	-0.6632	752.3554	$C_{30}H_{51}N_9O_{12}Na^+$	0.6360
IVNGEEAV	806.4025	$C_{34}H_{57}N_9O_{12}Na^+$	0.7118	834.3968	$C_{35}H_{57}N_9O_{13}Na^+$	-0.0203	851.4245	$C_{35}H_{60}N_{10}O_{13}Na^+$	1.3684
IVNGEEAVP	903.4563	$C_{39}H_{64}N_{10}O_{13}Na^+$	1.8086	x	x	x	x	x	x
IVNGEEAVPG	960.4762	$C_{41}H_{67}N_{11}O_{14}Na^+$	0.0345	988.4701	$C_{42}H_{67}N_{11}O_{15}Na^+$	-0.9118	1,005.4982	$C_{42}H_{70}N_{12}O_{15}Na^+$	0.6378
IVNGEEAVPGS	1047.5078	$C_{44}H_{72}N_{12}O_{16}Na^+$	-0.3697	1075.5042	$C_{45}H_{72}N_{12}O_{17}Na^+$	1.1047	1,092.5300	$C_{45}H_{75}N_{13}O_{17}Na^+$	0.3135
IVNGEEAVPGSW	1233.5870	$C_{55}H_{82}N_{14}O_{17}Na^+$	-0.3636	x	x	x	x	x	x
IVNGEEAVPGSWPW	1516.7212	$C_{71}H_{99}N_{17}O_{19}Na^+$	1.0909	1,544.7161	$C_{72}H_{99}N_{17}O_{20}Na^+$	1.0802	x	x	x

Supplementary Table 66 Peak list exported from SurfaceLab spectrum of α-chymotrypsin from bovine liver, consisting of ions detected in the spectrum and assigned as C-terminal sequence of the B chain IVNGEEAVPGSWPW. The sequence is observed as y, y+Na, and z+1 ions. The *m/z* values represent the experimentally observed center mass of each peak. The deviation (dev.) represents the parts per million (ppm) accuracy of the assignment. The colour corresponds to the presence of the observed sequence in α-chymotrypsin presented in Supplementary Figure 23.

	y			y + Na			z+1		
**Description**	***m/z***	**Assignment**	**Dev. (ppm)**	***m/z***	**Assignment**	**Dev. (ppm)**	***m/z***	**Assignment**	**Dev. (ppm)**
RY	340.1978	$C_{15}H_{26}N_5O_4^+$	-0.4456	362.1798	$C_{15}H_{25}N_5O_4Na^+$	-0.3176	323.1712	$C_{15}H_{23}N_4O_4^+$	-0.4901
TRY	441.2455	$C_{19}H_{33}N_6O_6^+$	-0.1419	463.2279	$C_{19}H_{32}N_6O_6Na^+$	0.6577	424.2190	$C_{19}H_{30}N_5O_6^+$	-0.2209
LTRY	x	x	x	576.3118	$C_{25}H_{43}N_7O_7Na^+$	0.3709	537.3033	$C_{25}H_{41}N_6O_7^+$	0.2901
GLTRY	x	x	x	x	x	x	594.3248	$C_{27}H_{44}N_7O_8^+$	0.3256
WGLTRY	x	x	x	x	x	x	780.4042	$C_{38}H_{54}N_9O_9^+$	0.3274
GWGLTRY	x	x	x	876.4342	$C_{40}H_{59}N_{11}O_{10}Na^+$	0.3697	837.4259	$C_{40}H_{57}N_{10}O_{10}^+$	0.6289
TGWGLTRY	x	x	x	x	x	x	938.4736	$C_{44}H_{64}N_{11}O_{12}^+$	0.5432
TTGWGLTRY	x	x	x	x	x	x	1039.5223	$C_{48}H_{71}N_{12}O_{14}^+$	1.5148
VTTGWGLTRY	x	x	x	x	x	x	1138.5914	$C_{53}H_{80}N_{13}O_{15}^+$	1.9637

Supplementary Table 67 Peak list exported from SurfaceLab spectrum of α-chymotrypsin from bovine liver, consisting of ions detected in the spectrum and assigned as N-terminal sequence of the C chain ANTPDRLQQA. The sequence is observed as a, b, c and a-NH_3 ions. The *m/z* values represent the experimentally observed center mass of each peak. The deviation (dev.) represents the parts per million (ppm) accuracy of the assignment. The colour corresponds to the presence of the observed sequence in α-chymotrypsin presented in Supplementary Figure 23.

	a			b			c			a-NH_3		
**Description**	***m/z***	**Assignment**	**Dev. (ppm)**	***m/z***	**Assignment**	**Dev. (ppm)**	***m/z***	**Assignment**	**Dev. (ppm)**	***m/z***	**Assignment**	**Dev. (ppm)**
AN	158.0922	$C_6H_{12}N_3O_2^+$	-1.2982	x	x	x	203.1137	$C_7H_{15}N_4O_3^+$	-0.9171	x	x	x
ANT	x	x	x	287.1347	$C_{11}H_{19}N_4O_5^+$	-0.8970	x	x	x	242.1134	$C_{10}H_{16}N_3O_4^+$	-0.4241
ANTP	356.1927	$C_{15}H_{26}N_5O_5^+$	-0.4034	384.1878	$C_{16}H_{26}N_5O_6^+$	0.0271	x	x	x	x	x	x
ANTPD	471.2198	$C_{19}H_{31}N_6O_8^+$	0.0197	x	x	x	x	x	x	x	x	x
ANTPDR	627.3213	$C_{25}H_{43}N_{10}O_9^+$	0.7068	655.3164	$C_{26}H_{43}N_{10}O_{10}^+$	0.8687	672.3428	$C_{26}H_{46}N_{11}O_{10}^+$	0.7078	x	x	x
ANTPDRL	740.4056	$C_{31}H_{54}N_{11}O_{10}^+$	0.8150	768.4005	$C_{32}H_{54}N_{11}O_{11}^+$	0.8590	785.4269	$C_{32}H_{57}N_{12}O_{11}^+$	0.5838	x	x	x
ANTPDRLQ	868.4644	$C_{36}H_{62}N_{13}O_{12}^+$	0.9576	896.4592	$C_{37}H_{62}N_{13}O_{13}^+$	0.8744	913.4858	$C_{37}H_{65}N_{14}O_{13}^+$	0.8534	851.4392	$C_{36}H_{59}N_{12}O_{12}^+$	2.5788
ANTPDRLQQ	996.5232	$C_{41}H_{70}N_{15}O_{14}^+$	1.1329	1024.5179	$C_{42}H_{70}N_{15}O_{15}^+$	0.8159	1041.5444	$C_{42}H_{73}N_{16}O_{15}^+$	0.7937	979.4974	$C_{41}H_{67}N_{14}O_{14}^+$	1.9010
ANTPDRLQQA	1067.5603	$C_{44}H_{75}N_{16}O_{15}^+$	0.9918	x	x	x	1112.5812	$C_{45}H_{78}N_{17}O_{16}^+$	0.4736	1049.5251	$C_{44}H_{71}N_{15}O_{15}^+$	0.2242

Alcohol dehydrogenase

a)

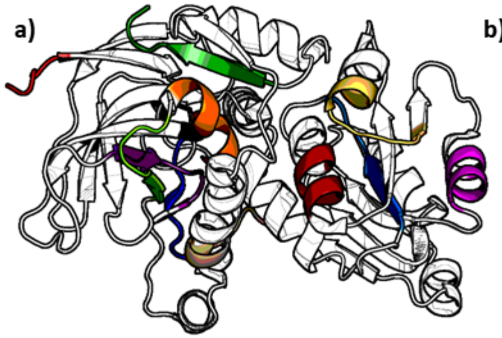

b)

Uniprot database code	Amino acid sequence
ADH1_YEAST1n	SIPETQKGVIFYESHGKLEYKDIPVPKPKANELLINVKYSGVCHTDLHAWHGDWPLP VKLPLVGGHEGAGVVVGMGENVKGWKIGDYAGIKWLNGSCMACEYCELGNESNC PHADLSGYTHDGSFQQYATADAVQAAHIPQGTDLAQVAPILCAGITVYKALKSANLM AGHWVAISGAAGGLGSLAVQYAKAMGYRVLGIDGGEGKEELFRSIGGEVFIDFTKE KDIVGAVLKATDGGAHGVINVSVSEAAIEASTRYVRANGTTVLVGMPAGAKCCSDV FNQVVKSISIVGSYVGNRADTREALDFFARGLVKSPIKVVGLSTLPEIYEKMEKGQIV GRYVVDTSK

Supplementary Figure 24 Alcohol dehydrogenase (a) cartoon exported from PDB entry 4W6Z[14] and (b) amino acid sequence exported from the UniProt database. The highlighted colours correspond to assigned segments of the amino acid sequence, presented in Supplementary Tables 68-82.

Supplementary Table 68 Peak list exported from SurfaceLab spectrum of alcohol dehydrogenase, consisting of ions detected in the spectrum and assigned as N-terminal sequence of the C chain ANTPDRLQQA. The *m/z* values represent the experimentally observed center mass of each peak. The deviation (dev.) represents the parts per million (ppm) accuracy of the assignment. The colour corresponds to the presence of the observed sequence in alcohol dehydrogenase presented in Supplementary Figure 24.

	a			b			c			a-NH3		
**Description**	***m/z***	**Assignment**	**Dev. (ppm)**	***m/z***	**Assignment**	**Dev. (ppm)**	***m/z***	**Assignment**	**Dev. (ppm)**	***m/z***	**Assignment**	**Dev. (ppm)**
SI				223.1055	$C_9H_{16}N_2O_3Na^+$	0.9373						
SIP	292.1632	$C_{13}H_{23}N_3O_3Na^+$	0.2685	320.1582	$C_{14}H_{23}N_3O_4Na^+$	0.4776						
SIPE	421.2060	$C_{18}H_{30}N_4O_6Na^+$	0.5182									
SIPET	522.2537	$C_{22}H_{37}N_5O_8Na^+$	0.4849									

Supplementary Table 69 Peak list exported from SurfaceLab spectrum of alcohol dehydrogenase, consisting of ions detected in the spectrum and assigned as C-terminus of the sequence RYVVDTSK. The *m/z* values represent the experimentally observed center mass of each peak. The deviation (dev.) represents the parts per million (ppm) accuracy of the assignment. The colour corresponds to the presence of the observed sequence in alcohol dehydrogenase presented in Supplementary Figure 24.

	y			z			z+1			z+2		
**Description**	***m/z***	**Assignment**	**Dev. (ppm)**	***m/z***	**Assignment**	**Dev. (ppm)**	***m/z***	**Assignment**	**Dev. (ppm)**	***m/z***	**Assignment**	**Dev. (ppm)**
TSK				319.1742	$C_{13}H_{25}N_3O_6^+$	1.2187				321.1899	$C_{13}H_{27}N_3O_6^+$	1.3031
DTSK				434.2012	$C_{17}H_{30}N_4O_9^+$	1.0099				436.2169	$C_{17}H_{32}N_4O_9^+$	1.0947
VDTSK				533.2698	$C_{22}H_{39}N_5O_{10}^+$	1.1498				535.2854	$C_{22}H_{41}N_5O_{10}^+$	1.1103
VVDTSK				632.3384	$C_{27}H_{48}N_6O_{11}^+$	1.3238				634.354	$C_{27}H_{50}N_6O_{11}^+$	1.1706
YVVDTSK												
RYVVDTSK										953.5191	$C_{42}H_{71}N_{11}O_{14}^+$	1.5431

Supplementary Table 70 Peak list exported from SurfaceLab spectrum of alcohol dehydrogenase, consisting of ions detected in the spectrum and assigned as internal fragments of the sequence RYVVDTS. The *m/z* values represent the experimentally observed center mass of each peak. The deviation (dev.) represents the parts per million (ppm) accuracy of the assignment. The colour corresponds to the presence of the observed sequence in alcohol dehydrogenase presented in Supplementary Figure 24.

	a			b			c			a-NH3		
**Description**	***m/z***	**Assignment**	**Dev. (ppm)**	***m/z***	**Assignment**	**Dev. (ppm)**	***m/z***	**Assignment**	**Dev. (ppm)**	***m/z***	**Assignment**	**Dev. (ppm)**
RYVV				542.3064	$C_{25}H_{41}N_7O_5Na^+$	0.4488				497.2853	$C_{24}H_{38}N_6O_4Na^+$	1.1632
RYVVD	629.3385	$C_{28}H_{46}N_8O_7Na^+$	0.5756	657.3337	$C_{29}H_{46}N_8O_8Na^+$	0.8807				612.3120	$C_{28}H_{43}N_7O_7Na^+$	0.5559
RYVVDT	730.3861	$C_{32}H_{53}N_9O_9Na^+$	0.3819							713.3596	$C_{32}H_{50}N_8O_9Na^+$	0.4172
RYVVDTS	817.4187	$C_{35}H_{58}N_{10}O_{11}Na^+$	1.0151				862.4415	$C_{36}H_{61}N_{11}O_{12}Na^+$	2.4951			

Supplementary Table 71 Peak list exported from SurfaceLab spectrum of alcohol dehydrogenase, consisting of ions detected in the spectrum and assigned as internal fragments of the sequence AQVAPIL. The *m/z* values represent the experimentally observed center mass of each peak. The deviation (dev.) represents the parts per million (ppm) accuracy of the assignment. The colour corresponds to the presence of the observed sequence in alcohol dehydrogenase presented in Supplementary Figure 24.

	a			b			c			a-NH3		
**Description**	***m/z***	**Assignment**	**Dev. (ppm)**	***m/z***	**Assignment**	**Dev. (ppm)**	***m/z***	**Assignment**	**Dev. (ppm)**	***m/z***	**Assignment**	**Dev. (ppm)**
AQ	196.1056	$C_7H_{15}N_3O_2Na^+$	-0.3407	224.1007	$C_8H_{15}N_3O_3Na^+$	0.5769				179.0789	$C_7H_{12}N_2O_2Na^+$	-0.8368
AQV	295.1742	$C_{12}H_{24}N_4O_3Na^+$	0.3305	323.1690	$C_{13}H_{24}N_4O_4Na^+$	0.1319						
AQVA	366.2112	$C_{15}H_{29}N_5O_4Na^+$	0.1582	394.2061	$C_{16}H_{29}N_5O_5Na^+$	0.0354						
AQVAP	463.2642	$C_{20}H_{36}N_6O_5Na^+$	0.5281	491.2590	$C_{21}H_{36}N_6O_6Na^+$	0.3572						
AQVAPI	576.3483	$C_{26}H_{47}N_7O_6Na^+$	0.5324	604.3435	$C_{27}H_{47}N_7O_7Na^+$	0.8842						
AQVAPIL	689.4324	$C_{32}H_{58}N_8O_7Na^+$	0.5153	717.4266	$C_{33}H_{58}N_8O_8Na^+$	-0.5119						

Supplementary Table 72 Peak list exported from SurfaceLab spectrum of alcohol dehydrogenase, consisting of ions detected in the spectrum and assigned as internal fragments of the sequence FRSIGGEV. The *m/z* values represent the experimentally observed center mass of each peak. The deviation (dev.) represents the parts per million (ppm) accuracy of the assignment. The colour corresponds to the presence of the observed sequence in alcohol dehydrogenase presented in Supplementary Figure 24.

	a			b			c			a-NH3		
**Description**	***m/z***	**Assignment**	**Dev. (ppm)**	***m/z***	**Assignment**	**Dev. (ppm)**	***m/z***	**Assignment**	**Dev. (ppm)**	***m/z***	**Assignment**	**Dev. (ppm)**
FRS				415.2065	$C_{18}H_{28}N_6O_4Na^+$	0.1939						
FRSI	500.2954	$C_{23}H_{39}N_7O_4Na^+$	-0.2797	528.2912	$C_{24}H_{39}N_7O_5Na^+$	1.3557	545.3179	$C_{24}H_{42}N_8O_5Na^+$	1.5401	483.2693	$C_{23}H_{36}N_6O_4Na^+$	0.6202
FRSIG	557.3172	$C_{25}H_{42}N_8O_5Na^+$	0.3272	585.3122	$C_{26}H_{42}N_8O_6Na^+$	0.4615				540.2910	$C_{25}H_{39}N_7O_5Na^+$	1.0308
FRSIGG	614.3388	$C_{27}H_{45}N_9O_6Na^+$	0.4851	642.3334	$C_{28}H_{45}N_9O_7Na^+$	-0.0315				597.3123	$C_{27}H_{42}N_8O_6Na^+$	0.5636
FRSIGGE	743.3837	$C_{32}H_{52}N_{10}O_9Na^+$	3.4548							788.4051	$C_{33}H_{55}N_{11}O_{10}Na^+$	3.2209
FRSIGGEV							887.4737	$C_{38}H_{64}N_{12}O_{11}Na^+$	3.0175	825.4231	$C_{37}H_{58}N_{10}O_{10}Na^+$	0.1656

Supplementary Table 73 Peak list exported from SurfaceLab spectrum of alcohol dehydrogenase, consisting of ions detected in the spectrum and assigned as internal fragments of the sequence VQYAKA. The *m/z* values represent the experimentally observed center mass of each peak. The deviation (dev.) represents the parts per million (ppm) accuracy of the assignment. The colour corresponds to the presence of the observed sequence in alcohol dehydrogenase presented in Supplementary Figure 24.

	a			b			c			a-NH3		
**Description**	***m/z***	**Assignment**	**Dev. (ppm)**	***m/z***	**Assignment**	**Dev. (ppm)**	***m/z***	**Assignment**	**Dev. (ppm)**	***m/z***	**Assignment**	**Dev. (ppm)**
VQ	224.1371	$C_9H_{19}N_3O_2Na^+$	0.5746	252.1319	$C_{10}H_{19}N_3O_3Na^+$	0.2076				207.1104	$C_9H_{16}N_2O_2Na^+$	-0.0131
VQY	387.2004	$C_{18}H_{28}N_4O_4Na^+$	0.3461	415.1953	$C_{19}H_{28}N_4O_5Na^+$	0.3277	432.2219	$C_{19}H_{31}N_5O_5Na^+$	0.4228	370.1739	$C_{18}H_{25}N_3O_4Na^+$	0.4678
VQYA	458.2375	$C_{21}H_{33}N_5O_5Na^+$	0.2677	486.2324	$C_{22}H_{33}N_5O_6Na^+$	0.2879	503.2590	$C_{22}H_{36}N_6O_6Na^+$	0.3839	441.2112	$C_{21}H_{30}N_4O_5Na^+$	0.7186
VQYAK	586.3327	$C_{27}H_{45}N_7O_6Na^+$	0.6089	614.3275	$C_{28}H_{45}N_7O_7Na^+$	0.4232	631.3542	$C_{28}H_{48}N_8O_7Na^+$	0.6197	569.3062	$C_{27}H_{42}N_6O_6Na^+$	0.6232
VQYAKA	657.3695	$C_{30}H_{50}N_8O_7Na^+$	0.0295	685.3645	$C_{31}H_{50}N_8O_8Na^+$	0.2204	702.3931	$C_{31}H_{53}N_9O_8Na^+$	3.0655	640.3429	$C_{30}H_{47}N_7O_7Na^+$	0.0159

Supplementary Table 74 Peak list exported from SurfaceLab spectrum of alcohol dehydrogenase, consisting of ions detected in the spectrum and assigned as internal fragments of the sequence GAVLKAT. The *m/z* values represent the experimentally observed center mass of each peak. The deviation (dev.) represents the parts per million (ppm) accuracy of the assignment. The colour corresponds to the presence of the observed sequence in alcohol dehydrogenase presented in Supplementary Figure 24.

	a			b			c			a-NH3		
**Description**	***m/z***	**Assignment**	**Dev. (ppm)**	***m/z***	**Assignment**	**Dev. (ppm)**	***m/z***	**Assignment**	**Dev. (ppm)**	***m/z***	**Assignment**	**Dev. (ppm)**
GAV	224.1371	$C_9H_{19}N_3O_2Na^+$	0.5746	252.1319	$C_{10}H_{19}N_3O_3Na^+$	0.2076				207.1104	$C_9H_{16}N_2O_2Na^+$	-0.0131
GAVL	337.2207	$C_{15}H_{30}N_4O_3Na^+$	-0.8032	365.2160	$C_{16}H_{30}N_4O_4Na^+$	0.1985				320.1946	$C_{15}H_{27}N_3O_3Na^+$	0.4133
GAVLK				493.3112	$C_{22}H_{42}N_6O_5Na^+$	0.6404				448.2895	$C_{21}H_{39}N_5O_4Na^+$	0.1837
GAVLKA	536.3538	$C_{24}H_{47}N_7O_5Na^+$	1.4037	564.3479	$C_{25}H_{47}N_7O_6Na^+$	-0.2451				519.3267	$C_{24}H_{44}N_6O_5Na^+$	0.3769
GAVLKAT				665.3967	$C_{29}H_{54}N_8O_8Na^+$	1.5304				620.3746	$C_{28}H_{51}N_7O_7Na^+$	0.5910

Supplementary Table 75 Peak list exported from SurfaceLab spectrum of alcohol dehydrogenase, consisting of ions detected in the spectrum and assigned as internal fragments of the sequence HADLSG. The *m/z* values represent the experimentally observed center mass of each peak. The deviation (dev.) represents the parts per million (ppm) accuracy of the assignment. The colour corresponds to the presence of the observed sequence in alcohol dehydrogenase presented in Supplementary Figure 24.

	a			b			c			a-NH3		
**Description**	***m/z***	**Assignment**	**Dev. (ppm)**	***m/z***	**Assignment**	**Dev. (ppm)**	***m/z***	**Assignment**	**Dev. (ppm)**	***m/z***	**Assignment**	**Dev. (ppm)**
HA	205.1060	$C_8H_{14}N_4ONa^+$	-0.0891	233.1010	$C_9H_{14}N_4O_2Na^+$	0.4705				188.0793	$C_8H_{11}N_3ONa^+$	-0.7133
HAD	320.1330	$C_{12}H_{19}N_5O_4Na^+$	0.3345	348.1280	$C_{13}H_{19}N_5O_5Na^+$	0.4537	365.155	$C_{13}H_{22}N_6O_5Na^+$	1.6112	303.1065	$C_{12}H_{16}N_4O_4Na^+$	0.5423
HADL	433.2171	$C_{18}H_{30}N_6O_5Na^+$	0.1849	461.2121	$C_{19}H_{30}N_6O_6Na^+$	0.4558	478.2388	$C_{19}H_{33}N_7O_6Na^+$	0.6317	416.1906	$C_{18}H_{27}N_5O_5Na^+$	0.4659
HADLS	520.2498	$C_{21}H_{35}N_7O_7Na^+$	1.4605	548.2447	$C_{22}H_{35}N_7O_8Na^+$	1.4702				503.2229	$C_{21}H_{32}N_6O_7Na^+$	0.7770
HADLSG	577.2710	$C_{23}H_{38}N_8O_8Na^+$	0.8693	605.2671	$C_{24}H_{38}N_8O_9Na^+$	2.8966				560.2440	$C_{23}H_{35}N_7O_8Na^+$	0.0970

Supplementary Table 76 Peak list exported from SurfaceLab spectrum of alcohol dehydrogenase, consisting of ions detected in the spectrum and assigned as internal fragments of the sequence AHIPQGT. The *m/z* values represent the experimentally observed center mass of each peak. The deviation (dev.) represents the parts per million (ppm) accuracy of the assignment. The colour corresponds to the presence of the observed sequence in alcohol dehydrogenase presented in Supplementary Figure 24.

	a			b			c			a-NH3		
**Description**	***m/z***	**Assignment**	**Dev. (ppm)**	***m/z***	**Assignment**	**Dev. (ppm)**	***m/z***	**Assignment**	**Dev. (ppm)**	***m/z***	**Assignment**	**Dev. (ppm)**
AH	205.1060	$C_8H_{14}N_4ONa^+$	-0.0891	233.101	$C_9H_{14}N_4O_2Na^+$	0.4705				188.0793	$C_8H_{11}N_3ONa^+$	-0.7133
AHI	318.1900	$C_{14}H_{25}N_5O_2Na^+$	-0.0767	346.1852	$C_{15}H_{25}N_5O_3Na^+$	0.5718				301.1637	$C_{14}H_{22}N_4O_2Na^+$	0.5145
AHIP	415.2430	$C_{19}H_{32}N_6O_3Na^+$	0.4147	443.2378	$C_{20}H_{32}N_6O_4Na^+$	0.1053				398.2164	$C_{19}H_{29}N_5O_3Na^+$	0.4391
AHIPQ	543.3013	$C_{24}H_{40}N_8O_5Na^+$	-0.1281	571.2966	$C_{25}H_{40}N_8O_6Na^+$	0.4647				526.2752	$C_{24}H_{37}N_7O_5Na^+$	0.7440
AHIPQG	600.3232	$C_{26}H_{43}N_9O_6Na^+$	0.6414	628.3181	$C_{27}H_{43}N_9O_7Na^+$	0.5415				583.2964	$C_{26}H_{40}N_8O_6Na^+$	0.2324
AHIPQGT	701.3733	$C_{30}H_{50}N_{10}O_8Na^+$	3.9968	729.3647	$C_{31}H_{50}N_{10}O_9Na^+$	-1.0366				684.3442	$C_{30}H_{47}N_9O_8Na^+$	0.3420

Supplementary Table 77 Peak list exported from SurfaceLab spectrum of alcohol dehydrogenase, consisting of ions detected in the spectrum and assigned as internal fragments of the sequence HIPQGTD. The *m/z* values represent the experimentally observed center mass of each peak. The deviation (dev.) represents the parts per million (ppm) accuracy of the assignment. The colour corresponds to the presence of the observed sequence in alcohol dehydrogenase presented in Supplementary Figure 24.

	a			b			C			a-NH3		
**Description**	***m/z***	**Assignment**	**Dev. (ppm)**	***m/z***	**Assignment**	**Dev. (ppm)**	***m/z***	**Assignment**	**Dev. (ppm)**	***m/z***	**Assignment**	**Dev. (ppm)**
HI	247.1529	$C_{11}H_{20}N_4ONa^+$	-0.2643	275.1479	$C_{12}H_{20}N_4O_2Na^+$	0.3644	292.1744	$C_{12}H_{23}N_5O_2Na^+$	0.1827	230.1264	$C_{11}H_{17}N_3ONa^+$	0.0546
HIP				372.2007	$C_{17}H_{27}N_5O_3Na^+$	0.2149				327.1790	$C_{16}H_{24}N_4O_2Na^+$	-0.5017
HIPQ	472.2643	$C_{21}H_{35}N_7O_4Na^+$	0.0926	500.2593	$C_{22}H_{35}N_7O_5Na^+$	0.1930	517.2859	$C_{22}H_{38}N_8O_5Na^+$	0.2402	455.2377	$C_{21}H_{32}N_6O_4Na^+$	-0.1164
HIPQG	529.2860	$C_{23}H_{38}N_8O_5Na^+$	0.4767	557.2808	$C_{24}H_{38}N_8O_6Na^+$	0.2667	574.3077	$C_{24}H_{41}N_9O_6Na^+$	0.9011	512.2593	$C_{23}H_{35}N_7O_5Na^+$	0.2829
HIPQGT	630.3335	$C_{27}H_{45}N_9O_7Na^+$	0.1849	658.3286	$C_{28}H_{45}N_9O_8Na^+$	0.4004				613.3072	$C_{27}H_{42}N_8O_7Na^+$	0.5399
HIPQGTD	772.4091	$C_{33}H_{55}N_{11}O_9Na^+$	1.9139							755.3817	$C_{33}H_{52}N_{10}O_9Na^+$	0.8454

Supplementary Table 78 Peak list exported from SurfaceLab spectrum of alcohol dehydrogenase, consisting of ions detected in the spectrum and assigned as internal fragments of the sequence QAAHIPQG. The *m/z* values represent the experimentally observed center mass of each peak. The deviation (dev.) represents the parts per million (ppm) accuracy of the assignment. The colour corresponds to the presence of the observed sequence in alcohol dehydrogenase presented in Supplementary Figure 24.

	a			b			c			a-NH3		
**Description**	***m/z***	**Assignment**	**Dev. (ppm)**	***m/z***	**Assignment**	**Dev. (ppm)**	***m/z***	**Assignment**	**Dev. (ppm)**	***m/z***	**Assignment**	**Dev. (ppm)**
QA	196.1056	$C_7H_{15}N_3O_2Na^+$	-0.3407	224.1007	$C_8H_{15}N_3O_3Na^+$	0.5769				179.0789	$C_7H_{12}N_2O_2Na^+$	-0.8368
QAA	267.1429	$C_{10}H_{20}N_4O_3Na^+$	0.5263	295.1378	$C_{11}H_{20}N_4O_4Na^+$	0.4391				250.1163	$C_{10}H_{17}N_3O_3Na^+$	0.3823
QAAH				432.1969	$C_{17}H_{27}N_7O_5Na^+$	0.6061				387.1753	$C_{16}H_{24}N_6O_4Na^+$	0.4363
QAAHI				545.2815	$C_{23}H_{38}N_8O_6Na^+$	1.5122						
QAAHIP												
QAAHIPQ	742.3969	$C_{32}H_{53}N_{11}O_8Na^+$	-0.2083	770.3926	$C_{33}H_{53}N_{11}O_9Na^+$	0.8102				725.3706	$C_{32}H_{50}N_{10}O_8Na^+$	0.1453
QAAHIPQG	799.4186	$C_{34}H_{56}N_{12}O_9Na^+$	0.0181	827.4132	$C_{35}H_{56}N_{12}O_{10}Na^+$	-0.2894				782.3921	$C_{34}H_{53}N_{11}O_9Na^+$	0.0739

Supplementary Table 79 Peak list exported from SurfaceLab spectrum of alcohol dehydrogenase, consisting of ions detected in the spectrum and assigned as internal fragments of the sequence LPLVGGHEG. The *m/z* values represent the experimentally observed center mass of each peak. The deviation (dev.) represents the parts per million (ppm) accuracy of the assignment. The colour corresponds to the presence of the observed sequence in alcohol dehydrogenase presented in Supplementary Figure 24.

	**a**			**b**			**c**			**a-NH3**		
**Description**	***m/z***	**Assignment**	**Dev. (ppm)**	***m/z***	**Assignment**	**Dev. (ppm)**	***m/z***	**Assignment**	**Dev. (ppm)**	***m/z***	**Assignment**	**Dev. (ppm)**
LPLVG	476.3211	$C_{23}H_{43}N_5O_4Na^+$	0.7412	504.3157	$C_{24}H_{43}N_5O_5Na^+$	0.1582						
LPLVGG	533.3425	$C_{25}H_{46}N_6O_5Na^+$	0.5145	561.3375	$C_{26}H_{46}N_6O_6Na^+$	0.6919	578.3644	$C_{26}H_{49}N_7O_6Na^+$	1.3319	516.3157	$C_{25}H_{43}N_5O_5Na^+$	0.0839
LPLVGGH				698.3956	$C_{32}H_{53}N_9O_7Na^+$	-0.5746				653.3749	$C_{31}H_{50}N_8O_6Na^+$	0.6036
LPLVGGHE				827.4389	$C_{37}H_{60}N_{10}O_{10}Na^+$	0.4104				782.4179	$C_{36}H_{57}N_9O_9Na^+$	0.9322
LPLVGGHEG	856.4681	$C_{38}H_{63}N_{11}O_{10}Na^+$	3.4062	884.4600	$C_{39}H_{63}N_{11}O_{11}Na^+$	-0.1029				839.4396	$C_{38}H_{60}N_{10}O_{10}Na^+$	1.2342

Supplementary Table 80 Peak list exported from SurfaceLab spectrum of alcohol dehydrogenase, consisting of ions detected in the spectrum and assigned as internal fragments of the sequence YVGNRA. The *m/z* values represent the experimentally observed center mass of each peak. The deviation (dev.) represents the parts per million (ppm) accuracy of the assignment. The colour corresponds to the presence of the observed sequence in alcohol dehydrogenase presented in Supplementary Figure 24.

	**a**			**b**			**c**			**a-NH3**		
**Description**	***m/z***	**Assignment**	**Dev. (ppm)**	***m/z***	**Assignment**	**Dev. (ppm)**	***m/z***	**Assignment**	**Dev. (ppm)**	***m/z***	**Assignment**	**Dev. (ppm)**
YV	259.1418	$C_{13}H_{20}N_2O_2Na^+$	0.5421	287.1367	$C_{14}H_{20}N_2O_3Na^+$	0.2579						
YVG	316.1633	$C_{15}H_{23}N_3O_3Na^+$	0.3147	344.1583	$C_{16}H_{23}N_3O_4Na^+$	0.5305						
YVGN	430.2063	$C_{19}H_{29}N_5O_5Na^+$	0.3975	458.2012	$C_{20}H_{29}N_5O_6Na^+$	0.3989	475.2278	$C_{20}H_{32}N_6O_6Na^+$	0.5347	413.1797	$C_{19}H_{26}N_4O_5Na^+$	0.3239
YVGNR	586.3070	$C_{25}H_{41}N_9O_6Na^+$	-0.2682	614.3022	$C_{26}H_{41}N_9O_7Na^+$	0.1609	631.3290	$C_{26}H_{44}N_{10}O_7Na^+$	0.4748	569.2807	$C_{25}H_{38}N_8O_6Na^+$	0.1178
YVGNRA	657.3449	$C_{28}H_{46}N_{10}O_7Na^+$	0.8505	685.3398	$C_{29}H_{46}N_{10}O_8Na^+$	0.8835						

Supplementary Table 81 Peak list exported from SurfaceLab spectrum of alcohol dehydrogenase, consisting of ions detected in the spectrum and assigned as internal fragments of the sequence YAGIKWL. The *m/z* values represent the experimentally observed center mass of each peak. The deviation (dev.) represents the parts per million (ppm) accuracy of the assignment. The colour corresponds to the presence of the observed sequence in alcohol dehydrogenase presented in Supplementary Figure 24.

	a			b			c			a-NH3		
**Description**	***m/z***	**Assignment**	**Dev. (ppm)**	***m/z***	**Assignment**	**Dev. (ppm)**	***m/z***	**Assignment**	**Dev. (ppm)**	***m/z***	**Assignment**	**Dev. (ppm)**
YA	231.1105	$C_{11}H_{16}N_2O_2Na^+$	0.5224	259.1055	$C_{12}H_{16}N_2O_3Na^+$	0.5538	276.132	$C_{12}H_{19}N_3O_3Na^+$	0.5252			
YAG	288.1320	$C_{13}H_{19}N_3O_3Na^+$	0.3891	316.127	$C_{14}H_{19}N_3O_4Na^+$	0.8105	333.1534	$C_{14}H_{22}N_4O_4Na^+$	0.3237	271.1054	$C_{13}H_{16}N_2O_3Na^+$	0.4042
YAGI	401.2161	$C_{19}H_{30}N_4O_4Na^+$	0.3709	429.2110	$C_{20}H_{30}N_4O_5Na^+$	0.3764	446.2376	$C_{20}H_{33}N_5O_5Na^+$	0.3601	384.1894	$C_{19}H_{27}N_3O_4Na^+$	0.1643
YAGIK	529.3111	$C_{25}H_{42}N_6O_5Na^+$	0.4775	557.3058	$C_{26}H_{42}N_6O_6Na^+$	-0.0475	574.3328	$C_{26}H_{45}N_7O_6Na^+$	0.7448	512.2842	$C_{25}H_{39}N_5O_5Na^+$	-0.2535
YAGIKW	715.3881	$C_{36}H_{52}N_8O_6Na^+$	-2.9229	743.3837	$C_{37}H_{52}N_8O_7Na^+$	-1.9566	760.4137	$C_{37}H_{55}N_9O_7Na^+$	2.6207			
YAGIKWL				856.4681	$C_{43}H_{63}N_9O_8Na^+$	-1.2907						

Supplementary Table 82 Peak list exported from SurfaceLab spectrum of alcohol dehydrogenase, consisting of ions detected in the spectrum and assigned as internal fragments of the sequence WVAISGAA. The *m/z* values represent the experimentally observed center mass of each peak. The deviation (dev.) represents the parts per million (ppm) accuracy of the assignment. The colour corresponds to the presence of the observed sequence in alcohol dehydrogenase presented in Supplementary Figure 24.

	a			b			c			a-NH3		
**Description**	***m/z***	**Assignment**	**Dev. (ppm)**	***m/z***	**Assignment**	**Dev. (ppm)**	***m/z***	**Assignment**	**Dev. (ppm)**	***m/z***	**Assignment**	**Dev. (ppm)**
WVAIS	553.3104	$C_{27}H_{42}N_6O_5Na^+$	-0.9048	581.3057	$C_{28}H_{42}N_6O_6Na^+$	-0.1180						
WVAISG	610.3335	$C_{29}H_{45}N_7O_6Na^+$	1.8569	638.3277	$C_{30}H_{45}N_7O_7Na^+$	0.6913	655.3541	$C_{30}H_{48}N_8O_7Na^+$	0.4516	593.3058	$C_{29}H_{42}N_6O_6Na^+$	-0.0395
WVAISGA	681.3693	$C_{32}H_{50}N_8O_7Na^+$	-0.1956	709.3650	$C_{33}H_{50}N_8O_8Na^+$	0.8179	726.392	$C_{33}H_{53}N_9O_8Na^+$	1.4972	664.3412	$C_{32}H_{47}N_7O_7Na^+$	-2.5319
WVAISGAA	752.4067	$C_{35}H_{55}N_9O_8Na^+$	0.0981	780.4022	$C_{36}H_{55}N_9O_9Na^+$	0.8723				735.3812	$C_{35}H_{52}N_8O_8Na^+$	1.5546

Concanavalin A

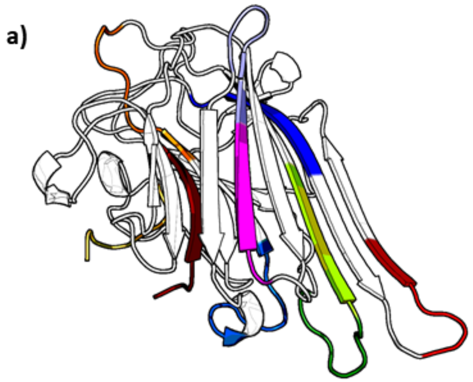

b) **Uniprot database code** **Amino acid sequence**

CONA_CANEN1n STHETNALHFMFNQFSKDQKDLILQGDATTGTDGNLELTRVSSNGSPQGSSVGRALFYAPVHIWESSAVVASFEATFTFLIKSPDSHPADGIAFFISNIDSSIPSGSTGRLLGLFPDAN

CONA_CANEN2n, ADTIVAVELDTYPNTDIGDPSYPHIGIDIKSVRSKKTAKWNMQNGKVGTAHIIYNSVDKRLSAVVSYPNADSATVSYDVDLDNVLPEWVRVGLSASTGLYKETNTILSWSFTSKLKSN

Supplementary Figure 25 Concanavalin A (a) cartoon exported from PDB entry 1JBC[15] and (b) amino acid sequence exported from the UniProt database. The highlighted colours correspond to assigned segments of the amino acid sequence, presented in Supplementary Tables 83-96.

Supplementary Table 83 Peak list exported from SurfaceLab spectrum of concanavalin A, consisting of ions detected in the spectrum and assigned as N-terminal sequence of the C chain STHETNA. The *m/z* values represent the experimentally observed center mass of each peak. The deviation (dev.) represents the parts per million (ppm) accuracy of the assignment. The colour corresponds to the presence of the observed sequence in concanavalin A presented in Supplementary Figure 25.

	a			b			c			a-NH3		
**Description**	***m/z***	**Assignment**	**Dev. (ppm)**	***m/z***	**Assignment**	**Dev. (ppm)**	***m/z***	**Assignment**	**Dev. (ppm)**	***m/z***	**Assignment**	**Dev. (ppm)**
STH	320.1330	$C_{12}H_{19}N_5O_4Na^+$	0.1034	348.1279	$C_{13}H_{19}N_5O_5Na^+$	0.0694	365.1548	$C_{13}H_{22}N_6O_5Na^+$	1.0212	303.1064	$C_{12}H_{16}N_4O_4Na^+$	0.1826
STHE	451.1912	$C_{17}H_{28}N_6O_7Na^+$	0.1487	479.1860	$C_{18}H_{28}N_6O_8Na^+$	-0.0886				434.1648	$C_{17}H_{25}N_5O_7Na^+$	0.3893
STHET	552.2390	$C_{21}H_{35}N_7O_9Na^+$	0.3334	580.2344	$C_{22}H_{35}N_7O_{10}Na^+$	1.1632	597.2644	$C_{22}H_{38}N_8O_{10}Na^+$	6.8240			
STHETN				694.2768	$C_{26}H_{41}N_9O_{12}Na^+$	0.1528						
STHETNA										737.3187	$C_{28}H_{46}N_{10}O_{12}Na^+$	-0.1991

Supplementary Table 84 Peak list exported from SurfaceLab spectrum of concanavalin A, consisting of ions detected in the spectrum and assigned as N-terminal sequence of the C chain ADTIVAVELD. The *m/z* values represent the experimentally observed center mass of each peak. The deviation (dev.) represents the parts per million (ppm) accuracy of the assignment. The colour corresponds to the presence of the observed sequence in concanavalin A presented in Supplementary Figure 25.

	a			b			c			a-NH3		
**Description**	***m/z***	**Assignment**	**Dev. (ppm)**	***m/z***	**Assignment**	**Dev. (ppm)**	***m/z***	**Assignment**	**Dev. (ppm)**	***m/z***	**Assignment**	**Dev. (ppm)**
ADT	282.1060	$C_{10}H_{17}N_3O_5Na^+$	-0.1010	310.1008	$C_{11}H_{17}N_3O_6Na^+$	-0.5641						
ADTI	395.1899	$C_{16}H_{28}N_4O_6Na^+$	-0.6391							440.2115	$C_{17}H_{31}N_5O_7Na^+$	-0.2715
ADTIV	494.2585	$C_{21}H_{37}N_5O_7Na^+$	-0.1322							539.2794	$C_{22}H_{40}N_6O_8Na^+$	-1.0424
ADTIVA	565.2957	$C_{24}H_{42}N_6O_8Na^+$	0.1725	593.2903	$C_{25}H_{42}N_6O_9Na^+$	-0.4997				610.3164	$C_{25}H_{45}N_7O_9Na^+$	-1.1316
ADTIVAV	664.3641	$C_{29}H_{51}N_7O_9Na^+$	0.1353							709.3858	$C_{30}H_{54}N_8O_{10}Na^+$	0.4062
ADTIVAVE	793.4070	$C_{34}H_{58}N_8O_{12}Na^+$	0.4147									
ADTIVAVEL	906.4878	$C_{40}H_{69}N_9O_{13}Na^+$	-3.2474							951.5113	$C_{41}H_{72}N_{10}O_{14}Na^+$	-0.8848
ADTIVAVELD				1049.5115	$C_{45}H_{74}N_{10}O_{17}Na^+$	-1.0182						

Supplementary Table 85 Peak list exported from SurfaceLab spectrum of concanavalin A, consisting of ions detected in the spectrum and assigned as internal fragments of the sequence IWESSAV. The *m/z* values represent the experimentally observed center mass of each peak. The deviation (dev.) represents the parts per million (ppm) accuracy of the assignment. The colour corresponds to the presence of the observed sequence in concanavalin A presented in Supplementary Figure 25.

	**a**			**b**			**c**			**a-NH3**		
**Description**	***m/z***	**Assignment**	**Dev. (ppm)**	***m/z***	**Assignment**	**Dev. (ppm)**	***m/z***	**Assignment**	**Dev. (ppm)**	***m/z***	**Assignment**	**Dev. (ppm)**
IWE	425.2159	$C_{21}H_{30}N_4O_4Na^+$	-0.0121	453.2110	$C_{22}H_{30}N_4O_5Na^+$	0.2444	470.2373	$C_{22}H_{33}N_5O_5Na^+$	-0.1180	408.1893	$C_{21}H_{27}N_3O_4Na^+$	-0.1149
IWES	512.2478	$C_{24}H_{35}N_5O_6Na^+$	-0.3826	540.2429	$C_{25}H_{35}N_5O_7Na^+$	-0.0340	557.2696	$C_{25}H_{38}N_6O_7Na^+$	0.3759			
IWESS	599.2802	$C_{27}H_{40}N_6O_8Na^+$	0.3398	627.2742	$C_{28}H_{40}N_6O_9Na^+$	-1.0498	644.3014	$C_{28}H_{43}N_7O_9Na^+$	-0.0987			
IWESSA	670.3152	$C_{30}H_{45}N_7O_9Na^+$	-2.7603	698.3111	$C_{31}H_{45}N_7O_{10}Na^+$	-1.2908	715.3377	$C_{31}H_{48}N_8O_{10}Na^+$	-1.2628			
IWESSAV	769.3847	$C_{35}H_{54}N_8O_{10}Na^+$	-1.0450	797.3806	$C_{36}H_{54}N_8O_{11}Na^+$	0.2218						

Supplementary Table 86 Peak list exported from SurfaceLab spectrum of concanavalin A, consisting of ions detected in the spectrum and assigned as internal fragments of the sequence VHIWESS. The *m/z* values represent the experimentally observed center mass of each peak. The deviation (dev.) represents the parts per million (ppm) accuracy of the assignment. The colour corresponds to the presence of the observed sequence in concanavalin A presented in Supplementary Figure 25.

	**a**			**b**			**c**			**a-NH3**		
**Description**	***m/z***	**Assignment**	**Dev. (ppm)**	***m/z***	**Assignment**	**Dev. (ppm)**	***m/z***	**Assignment**	**Dev. (ppm)**	***m/z***	**Assignment**	**Dev. (ppm)**
VHIWE	661.3430	$C_{32}H_{46}N_8O_6Na^+$	-0.3860	689.3376	$C_{33}H_{46}N_8O_7Na^+$	-0.7618	692.3575	$C_{33}H_{49}N_8O_7Na^+$	-6.0154	644.3155	$C_{32}H_{43}N_7O_6Na^+$	-1.8877
VHIWES	748.3748	$C_{35}H_{51}N_9O_8Na^+$	-0.6577	776.3692	$C_{36}H_{51}N_9O_9Na^+$	-1.2392	793.3967	$C_{36}H_{54}N_{10}O_9Na^+$	-0.0521			
VHIWESS	835.4075	$C_{38}H_{56}N_{10}O_{10}Na^+$	0.2717									

Supplementary Table 87 Peak list exported from SurfaceLab spectrum of concanavalin A, consisting of ions detected in the spectrum and assigned as internal fragments of the sequence AVVASFEA. The *m/z* values represent the experimentally observed center mass of each peak. The deviation (dev.) represents the parts per million (ppm) accuracy of the assignment. The colour corresponds to the presence of the observed sequence in concanavalin A presented in Supplementary Figure 25.

	a			b			c			a-NH3		
**Description**	***m/z***	**Assignment**	**Dev. (ppm)**	***m/z***	**Assignment**	**Dev. (ppm)**	***m/z***	**Assignment**	**Dev. (ppm)**	***m/z***	**Assignment**	**Dev. (ppm)**
AVVA	337.2212	$C_{15}H_{30}N_4O_3Na^+$	0.6235	365.2161	$C_{16}H_{30}N_4O_4Na^+$	0.4136				320.1945	$C_{15}H_{27}N_3O_3Na^+$	0.2497
AVVAS	424.2530	$C_{18}H_{35}N_5O_5Na^+$	-0.1753	452.2477	$C_{19}H_{35}N_5O_6Na^+$	-0.4649				407.2265	$C_{18}H_{32}N_4O_5Na^+$	-0.0789
AVVASF	571.3215	$C_{27}H_{44}N_6O_6Na^+$	0.0572	599.3166	$C_{28}H_{44}N_6O_7Na^+$	0.3972				554.2948	$C_{27}H_{41}N_5O_6Na^+$	-0.2003
AVVASFE				728.3590	$C_{33}H_{51}N_7O_{10}Na^+$	0.0381						
AVVASFEA	771.4007	$C_{35}H_{56}N_8O_{10}Na^+$	-0.6392									

Supplementary Table 88 Peak list exported from SurfaceLab spectrum of concanavalin A, consisting of ions detected in the spectrum and assigned as internal fragments of the sequence DSHPADGI. The *m/z* values represent the experimentally observed center mass of each peak. The deviation (dev.) represents the parts per million (ppm) accuracy of the assignment. The colour corresponds to the presence of the observed sequence in concanavalin A presented in Supplementary Figure 25.

	a			b			c			a-NH3		
**Description**	***m/z***	**Assignment**	**Dev. (ppm)**	***m/z***	**Assignment**	**Dev. (ppm)**	***m/z***	**Assignment**	**Dev. (ppm)**	***m/z***	**Assignment**	**Dev. (ppm)**
DSH	314.1449	$C_{12}H_{20}N_5O_5^+$	-3.0694	342.1424	$C_{13}H_{20}N_5O_6^+$	4.6400	359.1690	$C_{13}H_{23}N_6O_6^+$	4.4878			
DSHP	411.2003	$C_{17}H_{27}N_6O_6^+$	3.9642	439.1953	$C_{18}H_{27}N_6O_7^+$	3.8318	456.2215	$C_{18}H_{30}N_7O_7^+$	2.9802	394.1735	$C_{17}H_{24}N_5O_6^+$	3.6368
DSHPA	482.2372	$C_{20}H_{32}N_7O_7^+$	2.9147	510.2322	$C_{21}H_{32}N_7O_8^+$	3.0327	527.2580	$C_{21}H_{35}N_8O_8^+$	1.4667	465.2069	$C_{20}H_{29}N_6O_7^+$	-4.9318
DSHPAD	597.2644	$C_{24}H_{37}N_8O_{10}^+$	2.7968	625.2582	$C_{25}H_{37}N_8O_{11}^+$	0.8764	642.2853	$C_{25}H_{40}N_9O_{11}^+$	1.6809	580.2344	$C_{24}H_{34}N_7O_{10}^+$	-2.9821
DSHPADG	654.2857	$C_{26}H_{40}N_9O_{11}^+$	2.2988	682.2786	$C_{27}H_{40}N_9O_{12}^+$	-0.7010						
DSHPADGI	767.3684	$C_{32}H_{51}N_{10}O_{12}^+$	0.1681	795.3624	$C_{33}H_{51}N_{10}O_{13}^+$	-0.9835						

Supplementary Table 89 Peak list exported from SurfaceLab spectrum of concanavalin A, consisting of ions detected in the spectrum and assigned as internal fragments of the sequence GLFPDAN. The *m/z* values represent the experimentally observed center mass of each peak. The deviation (dev.) represents the parts per million (ppm) accuracy of the assignment. The colour corresponds to the presence of the observed sequence in concanavalin A presented in Supplementary Figure 25.

	a			b			c			a-NH3		
**Description**	***m/z***	**Assignment**	**Dev. (ppm)**	***m/z***	**Assignment**	**Dev. (ppm)**	***m/z***	**Assignment**	**Dev. (ppm)**	***m/z***	**Assignment**	**Dev. (ppm)**
GLF	314.1837	$C_{16}H_{25}N_3O_2Na^+$	-0.6427	342.1789	$C_{17}H_{25}N_3O_3Na^+$	0.3825	359.2053	$C_{17}H_{28}N_4O_3Na^+$	-0.2312			
GLFP	411.2358	$C_{21}H_{32}N_4O_3Na^+$	-2.1993	439.2313	$C_{22}H_{32}N_4O_4Na^+$	-0.6381						
GLFPD	526.2634	$C_{25}H_{37}N_5O_6Na^+$	-0.4274	554.2585	$C_{26}H_{37}N_5O_7Na^+$	-0.1183						
GLFPDA	597.3009	$C_{28}H_{42}N_6O_7Na^+$	0.2390	625.2954	$C_{29}H_{42}N_6O_8Na^+$	-0.4098	642.3223	$C_{29}H_{45}N_7O_8Na^+$	0.2108			
GLFPDAN	711.3480	$C_{32}H_{48}N_8O_9Na^+$	6.0708	739.3368	$C_{33}H_{48}N_8O_{10}Na^+$	-2.3831						

Supplementary Table 90 Peak list exported from SurfaceLab spectrum of concanavalin A, consisting of ions detected in the spectrum and assigned as internal fragments of the sequence KRLSAV. The *m/z* values represent the experimentally observed center mass of each peak. The deviation (dev.) represents the parts per million (ppm) accuracy of the assignment. The colour corresponds to the presence of the observed sequence in concanavalin A presented in Supplementary Figure 25.

	a			b			c			a-NH3		
**Description**	***m/z***	**Assignment**	**Dev. (ppm)**	***m/z***	**Assignment**	**Dev. (ppm)**	***m/z***	**Assignment**	**Dev. (ppm)**	***m/z***	**Assignment**	**Dev. (ppm)**
KR				285.2034	$C_{12}H_{25}N_6O_2^+$	0.0386	302.2295	$C_{12}H_{28}N_7O_2^+$	-1.2714			
KRL							415.3137	$C_{18}H_{39}N_8O_3^+$	-0.7484			
KRLS	457.3249	$C_{20}H_{41}N_8O_4^+$	0.8354	485.3189	$C_{21}H_{41}N_8O_5^+$	-1.1802	502.3460	$C_{21}H_{44}N_9O_5^+$	-0.0772	440.2979	$C_{20}H_{38}N_7O_4^+$	-0.2009
KRLSA	528.3616	$C_{23}H_{46}N_9O_5^+$	-0.1121	556.3569	$C_{24}H_{46}N_9O_6^+$	0.5953				511.3352	$C_{23}H_{43}N_8O_5^+$	0.1463
KRLSAV	627.4299	$C_{28}H_{55}N_{10}O_6^+$	-0.2339									

Supplementary Table 91 Peak list exported from SurfaceLab spectrum of concanavalin A, consisting of ions detected in the spectrum and assigned as internal fragments of the sequence NFTSKL. The *m/z* values represent the experimentally observed center mass of each peak. The deviation (dev.) represents the parts per million (ppm) accuracy of the assignment. The colour corresponds to the presence of the observed sequence in concanavalin A presented in Supplementary Figure 25.

	**a**			**b**			**c**			**a-NH3**		
**Description**	***m/z***	**Assignment**	**Dev. (ppm)**	***m/z***	**Assignment**	**Dev. (ppm)**	***m/z***	**Assignment**	**Dev. (ppm)**	***m/z***	**Assignment**	**Dev. (ppm)**
NF	258.1214	$C_{12}H_{17}N_3O_2Na^+$	0.2574	286.1163	$C_{13}H_{17}N_3O_3Na^+$	0.4753	303.1428	$C_{13}H_{20}N_4O_3Na^+$	0.1299	241.0947	$C_{12}H_{14}N_2O_2Na^+$	-0.0561
NFT	359.1690	$C_{16}H_{24}N_4O_4Na^+$	-0.0156	387.1638	$C_{17}H_{24}N_4O_5Na^+$	-0.1096	404.1905	$C_{17}H_{27}N_5O_5Na^+$	0.0922	342.1424	$C_{16}H_{21}N_3O_4Na^+$	-0.0665
NFTS	446.2010	$C_{19}H_{29}N_5O_6Na^+$	-0.0586	474.1957	$C_{20}H_{29}N_5O_7Na^+$	-0.4141	491.2224	$C_{20}H_{32}N_6O_7Na^+$	-0.1527	429.1745	$C_{19}H_{26}N_4O_6Na^+$	0.0920
NFTSK	574.2964	$C_{25}H_{41}N_7O_7Na^+$	0.6763	602.2913	$C_{26}H_{41}N_7O_8Na^+$	0.6129				557.2696	$C_{25}H_{38}N_6O_7Na^+$	0.3759
NFTSKL	687.3799	$C_{31}H_{52}N_8O_8Na^+$	-0.1199	715.3762	$C_{32}H_{52}N_8O_9Na^+$	1.7149				670.3538	$C_{31}H_{49}N_7O_8Na^+$	0.5320

Supplementary Table 92 Peak list exported from SurfaceLab spectrum of concanavalin A, consisting of ions detected in the spectrum and assigned as internal fragments of the sequence NTILSWS. The *m/z* values represent the experimentally observed center mass of each peak. The deviation (dev.) represents the parts per million (ppm) accuracy of the assignment. The colour corresponds to the presence of the observed sequence in concanavalin A presented in Supplementary Figure 25.

	**a**			**b**			**c**			**a-NH3**		
**Description**	***m/z***	**Assignment**	**Dev. (ppm)**	***m/z***	**Assignment**	**Dev. (ppm)**	***m/z***	**Assignment**	**Dev. (ppm)**	***m/z***	**Assignment**	**Dev. (ppm)**
NT	212.1005	$C_7H_{15}N_3O_3Na^+$	-0.3532	240.0954	$C_8H_{15}N_3O_4Na^+$	-0.1197				195.0740	$C_7H_{12}N_2O_3Na^+$	-0.2581
NTI	325.1845	$C_{13}H_{26}N_4O_4Na^+$	-0.2843	353.1794	$C_{14}H_{26}N_4O_5Na^+$	-0.4784				308.1581	$C_{13}H_{23}N_3O_4Na^+$	0.0736
NTIL				466.2637	$C_{20}H_{37}N_5O_6Na^+$	0.2610				421.2417	$C_{19}H_{34}N_4O_5Na^+$	-0.9707
NTILS										508.2737	$C_{22}H_{39}N_5O_7Na^+$	-0.8549
NTILSW	711.3790	$C_{33}H_{52}N_8O_8Na^+$	-1.4326	739.3742	$C_{34}H_{52}N_8O_9Na^+$	-1.0047	756.4041	$C_{34}H_{55}N_9O_9Na^+$	3.3827	694.3537	$C_{33}H_{49}N_7O_8Na^+$	0.3740
NTILSWS				826.4065	$C_{37}H_{57}N_9O_{11}Na^+$	-0.5289	843.4363	$C_{37}H_{60}N_{10}O_{11}Na^+$	3.2588	781.3857	$C_{36}H_{54}N_8O_{10}Na^+$	0.2178

Supplementary Table 93 Peak list exported from SurfaceLab spectrum of concanavalin A, consisting of ions detected in the spectrum and assigned as internal fragments of the sequence NVLPEWVR. The *m/z* values represent the experimentally observed center mass of each peak. The deviation (dev.) represents the parts per million (ppm) accuracy of the assignment. The colour corresponds to the presence of the observed sequence in concanavalin A presented in Supplementary Figure 25.

	a			b			c			a-NH3		
**Description**	***m/z***	**Assignment**	**Dev. (ppm)**	***m/z***	**Assignment**	**Dev. (ppm)**	***m/z***	**Assignment**	**Dev. (ppm)**	***m/z***	**Assignment**	**Dev. (ppm)**
NV	210.1213	$C_8H_{17}N_3O_2Na^+$	-0.2018	238.1162	$C_9H_{17}N_3O_3Na^+$	0.1336				193.0947	$C_8H_{14}N_2O_2Na^+$	-0.4004
NVL	323.2054	$C_{14}H_{28}N_4O_3Na^+$	0.0675	351.2003	$C_{15}H_{28}N_4O_4Na^+$	-0.0160				306.1789	$C_{14}H_{25}N_3O_3Na^+$	0.1958
NVLP	420.2581	$C_{19}H_{35}N_5O_4Na^+$	-0.1423	448.2530	$C_{20}H_{35}N_5O_5Na^+$	0.0006				403.2316	$C_{19}H_{32}N_4O_4Na^+$	0.1019
NVLPE	549.3003	$C_{24}H_{42}N_6O_7Na^+$	-0.7551	577.2956	$C_{25}H_{42}N_6O_8Na^+$	-0.0859				532.2742	$C_{24}H_{39}N_5O_7Na^+$	-0.0017
NVLPEW	735.3801	$C_{35}H_{52}N_8O_8Na^+$	0.1333	763.3750	$C_{36}H_{52}N_8O_9Na^+$	0.0671	780.4016	$C_{36}H_{55}N_9O_9Na^+$	0.1695			
NVLPEWV	834.4478	$C_{40}H_{61}N_9O_9Na^+$	-0.8263	862.4410	$C_{41}H_{61}N_9O_{10}Na^+$	-2.6831	879.4714	$C_{41}H_{64}N_{10}O_{10}Na^+$	1.7266	817.4206	$C_{40}H_{58}N_8O_9Na^+$	-1.6083
NVLPEWVR				1018.5432	$C_{47}H_{73}N_{13}O_{11}Na^+$	-1.2851				973.5216	$C_{46}H_{70}N_{12}O_{10}Na^+$	-1.4025

Supplementary Table 94 Peak list exported from SurfaceLab spectrum of concanavalin A, consisting of ions detected in the spectrum and assigned as internal fragments of the sequence SHPADGIA. The *m/z* values represent the experimentally observed center mass of each peak. The deviation (dev.) represents the parts per million (ppm) accuracy of the assignment. The colour corresponds to the presence of the observed sequence in concanavalin A presented in Supplementary Figure 25.

	a			b			c			a-NH3		
**Description**	***m/z***	**Assignment**	**Dev. (ppm)**	***m/z***	**Assignment**	**Dev. (ppm)**	***m/z***	**Assignment**	**Dev. (ppm)**	***m/z***	**Assignment**	**Dev. (ppm)**
SH	220.0932	$C_8H_{13}N_4O_2Na^+$	0.3541	248.0883	$C_9H_{13}N_4O_3Na^+$	1.4210						
SHP	318.1538	$C_{13}H_{21}N_5O_3Na^+$	0.5389	346.1486	$C_{14}H_{21}N_5O_4Na^+$	0.0917	363.1751	$C_{14}H_{24}N_6O_4Na^+$	0.0270	301.1272	$C_{13}H_{18}N_4O_3Na^+$	0.2767
SHPA	389.1907	$C_{16}H_{26}N_6O_4Na^+$	-0.1028	417.1858	$C_{17}H_{26}N_6O_5Na^+$	0.1664				372.1644	$C_{16}H_{23}N_5O_4Na^+$	0.3492
SHPAD	504.2179	$C_{20}H_{31}N_7O_7Na^+$	0.2862	532.2130	$C_{21}H_{31}N_7O_8Na^+$	0.6166	549.2400	$C_{21}H_{34}N_8O_8Na^+$	1.5038	487.1914	$C_{20}H_{28}N_6O_7Na^+$	0.5515
SHPADG				589.2347	$C_{23}H_{34}N_8O_9Na^+$	1.0145				544.2144	$C_{22}H_{31}N_7O_8Na^+$	3.2771
SHPADGI	674.3267	$C_{28}H_{45}N_9O_9Na^+$	5.0710	702.3208	$C_{29}H_{45}N_9O_{10}Na^+$	3.7727	719.3486	$C_{29}H_{48}N_{10}O_{10}Na^+$	5.4423			
SHPADGIA							790.3846	$C_{32}H_{53}N_{11}O_{11}Na^+$	3.5132	728.3355	$C_{31}H_{47}N_9O_{10}Na^+$	2.3045

Supplementary Table 95 Peak list exported from SurfaceLab spectrum of concanavalin A, consisting of ions detected in the spectrum and assigned as internal fragments of the sequence VASFEA. The *m/z* values represent the experimentally observed center mass of each peak. The deviation (dev.) represents the parts per million (ppm) accuracy of the assignment. The colour corresponds to the presence of the observed sequence in concanavalin A presented in Supplementary Figure 25.

	a			b			c			a-NH3		
**Description**	***m/z***	**Assignment**	**Dev. (ppm)**	***m/z***	**Assignment**	**Dev. (ppm)**	***m/z***	**Assignment**	**Dev. (ppm)**	***m/z***	**Assignment**	**Dev. (ppm)**
VAS	254.1475	$C_{10}H_{21}N_3O_3Na^+$	0.0875							237.1211	$C_{10}H_{18}N_2O_3Na^+$	0.3901
VASF	401.2158	$C_{19}H_{30}N_4O_4Na^+$	-0.3246	429.2108	$C_{20}H_{30}N_4O_5Na^+$	-0.1236	446.2374	$C_{20}H_{33}N_5O_5Na^+$	0.0194	384.1893	$C_{19}H_{27}N_3O_4Na^+$	-0.0821
VASFE	530.2589	$C_{24}H_{37}N_5O_7Na^+$	0.6272				575.2801	$C_{25}H_{40}N_6O_8Na^+$	0.2414			
VASFEA	601.2955	$C_{27}H_{42}N_6O_8Na^+$	-0.2088	629.2898	$C_{28}H_{42}N_6O_9Na^+$	-1.1448	646.3165	$C_{28}H_{45}N_7O_9Na^+$	-0.9078			

Supplementary Table 96 Peak list exported from SurfaceLab spectrum of concanavalin A, consisting of ions detected in the spectrum and assigned as internal fragments of the sequence VVSYPNAD. The *m/z* values represent the experimentally observed center mass of each peak. The deviation (dev.) represents the parts per million (ppm) accuracy of the assignment. The colour corresponds to the presence of the observed sequence in concanavalin A presented in Supplementary Figure 25.

	a			b			c			a-NH3		
**Description**	***m/z***	**Assignment**	**Dev. (ppm)**	***m/z***	**Assignment**	**Dev. (ppm)**	***m/z***	**Assignment**	**Dev. (ppm)**	***m/z***	**Assignment**	**Dev. (ppm)**
VVSYP				570.2905	$C_{27}H_{41}N_5O_7Na^+$	1.1186						
VVSYPN	656.3374	$C_{30}H_{47}N_7O_8Na^+$	-0.5844	684.3326	$C_{31}H_{47}N_7O_9Na^+$	-0.2355	701.3597	$C_{31}H_{50}N_8O_9Na^+$	0.5346	639.3105	$C_{30}H_{44}N_6O_8Na^+$	-1.2272
VVSYPNA	727.3748	$C_{33}H_{52}N_8O_9Na^+$	-0.1510	755.3686	$C_{34}H_{52}N_8O_{10}Na^+$	-1.6544	772.3992	$C_{34}H_{55}N_9O_{10}Na^+$	3.6490	710.3479	$C_{33}H_{49}N_7O_9Na^+$	-0.6636
VVSYPNAD	842.4014	$C_{37}H_{57}N_9O_{12}Na^+$	-0.5450	870.3973	$C_{38}H_{57}N_9O_{13}Na^+$	0.5960						

Bovine serum albumin and human serum albumin

a)

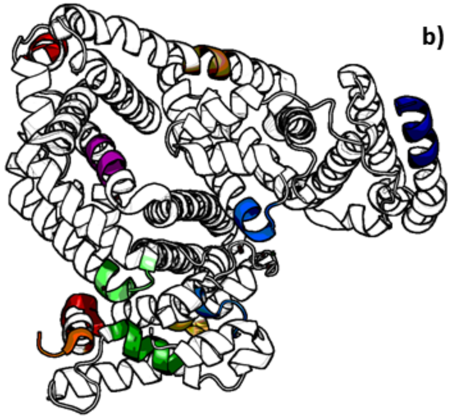

b)

Uniprot database code

ALBU_BOVIN1n

Amino acid sequence

DTHKSEIAHRFKDLGEEHFKGLVLIAFSQYLQQCPFDEHVKLVNELTEFAKTCVADESHA
GCEKSLHTLFGDELCKVASLRETYGDMADCCEKQEPERNECFLSHKDDSPDLPKLKPD
PNTLCDEFKADEKKFWGKYLYEIARRHPYFYAPELLYYANKYNGVFQECCQAEDKGACL
LPKIETMREKVLTSSARQRLRCASIQKFGERALKAWSVARLSQKFPKAEFVEVTKLVTDL
TKVHKECCHGDLLECADDRADLAKYICDNQDTISSKLKECCDKPLLEKSHCIAEVEKDAI
PENLPPLTADFAEDKDVCKNYQEAKDAFLGSFLYEYSRRHPEYAVSVLLRLAKEYEATL
EECCAKDDPHACYSTVFDKLKHLVDEPQNLIKQNCDQFEKLGEYGFQNALIVRYTRKVP
QVSTPTLVEVSRSLGKVGTRCCTKPESERMPCTEDYLSLILNRLCVLHEKTPVSEKVTKC
CTESLVNRRPCFSALTPDETYVPKAFDEKLFTFHADICTLPDTEKQIKKQTALVELLKHKP
KATEEQLKTVMENFVAFVDKCCAADDKEACFAVEGPKLVVSTQTALA

c)

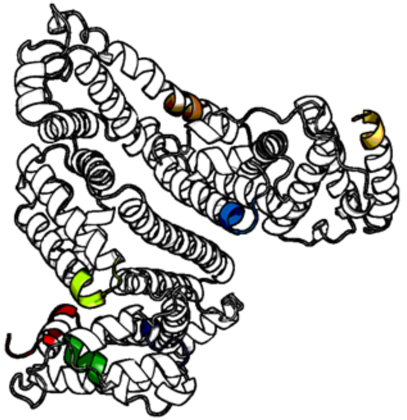

d)

Uniprot database code

ALBU_HUMAN1n

Amino acid sequence

DAHKSEVAHRFKDLGEENFKALVLIAFAQYLQQCPFEDHVKLVNEVTEFAKTCVADESA
ENCDKSLHTLFGDKLCTVATLRETYGEMADCCAKQEPERNECFLQHKDDNPNLPRLV
RPEVDVMCTAFHDNEETFLKKYLYEIARRHPYFYAPELLFFAKRYKAAFTECCQAADKA
ACLLPKLDELRDEGKASSAKQRLKCASLQKFGERAFKAWAVARLSQRFPKAEFAEVSK
LVTDLTKVHTECCHGDLLECADDRADLAKYICENQDSISSKLKECCEKPLLEKSHCIAEV
ENDEMPADLPSLAADFVESKDVCKNYAEAKDVFLGMFLYEYARRHPDYSVVLLLRLAK
TYETTLEKCCAAADPHECYAKVFDEFKPLVEEPQNLIKQNCELFEQLGEYKFQNALLVR
YTKKVPQVSTPTLVEVSRNLGKVGSKCCKHPEAKRMPCAEDYLSVVLNQLCVLHEKTP
VSDRVTKCCTESLVNRRPCFSALEVDETYVPKEFNAETFTFHADICTLSEKERQIKKQTA
LVELVKHKPKATKEQLKAVMDDFAAFVEKCCKADDKETCFAEEGKKLVAASQAALGL

Supplementary Figure 26 (a) Bovine serum albumin exported from PDB entry 4F5S[16] and (c) human serum albumin exported from PDB entry 1AO6[17] and (b) bovine serum albumin amino acid sequence and (d) human serum albumin amino acid sequence exported from the UniProt database. The highlighted colours correspond to assigned segments of the amino acid sequence, presented in Supplementary Tables 97-111.

Sequences specific to bovine serum albumin (BSA):

Supplementary Table 97 Peak list exported from SurfaceLab spectrum of bovine serum albumin, consisting of ions detected in the spectrum and assigned as sodium adducts of N-terminal sequence DTHK. The sequence is observed as a, b and a-NH_3 ions. The *m/z* values represent the experimentally observed center mass of each peak. The deviation (dev.) represents the parts per million (ppm) accuracy of the assignment. The colour corresponds to the presence of the observed sequence in bovine serum albumin presented in Supplementary Figure 26.

	a			b			c			a-NH_3		
**Description**	***m/z***	**Assignment**	**Dev. (ppm)**	***m/z***	**Assignment**	**Dev. (ppm)**	***m/z***	**Assignment**	**Dev. (ppm)**	***m/z***	**Assignment**	**Dev. (ppm)**
DTH	348.1278	$C_{13}H_{19}N_5O_5Na^+$	-0.1652	x	x	x	x	x	x	331.1013	$C_{13}H_{16}N_4O_5Na^+$	-0.07559
DTHK	476.2228	$C_{19}H_{31}N_7O_6Na^+$	0.0452	504.218	$C_{20}H_{31}N_7O_7Na^+$	0.4606	x	x	x	459.1962	$C_{19}H_{28}N_6O_6Na^+$	-0.07392

Supplementary Table 98 Peak list exported from SurfaceLab spectrum of bovine serum albumin, consisting of ions detected in the spectrum and assigned fragments of N-terminal sequence DTHKSEI. The *m/z* values represent the experimentally observed center mass of each peak. The deviation (dev.) represents the parts per million (ppm) accuracy of the assignment. The colour corresponds to the presence of the observed sequence in bovine serum albumin presented in Supplementary Figure 26.

	a			b			c			a-NH_3		
**Description**	***m/z***	**Assignment**	**Dev. (ppm)**	***m/z***	**Assignment**	**Dev. (ppm)**	***m/z***	**Assignment**	**Dev. (ppm)**	***m/z***	**Assignment**	**Dev. (ppm)**
DTHK	x	x	x	x	x	x	x	x	x	437.2155	$C_{19}H_{29}N_6O_6^+$	2.6766
DTHKS	x	x	x	569.2689	$C_{23}H_{37}N_8O_9^+$	1.9591	x	x	x	524.2477	$C_{22}H_{34}N_7O_8^+$	2.5712
DTHKSE	x	x	x	x	x	x	x	x	x	653.2888	$C_{27}H_{41}N_8O_{11}^+$	-0.2240
DTHKSEI	783.3999	$C_{33}H_{55}N_{10}O_{12}^+$	0.4793	x	x	x	x	x	x	x	x	x

Supplementary Table 99 Peak list exported from SurfaceLab spectrum of bovine serum albumin, consisting of ions detected in the spectrum and assigned as sodium adducts of internal fragments of the sequence, ADEKKF. The *m/z* values represent the experimentally observed center mass of each peak. The deviation (dev.) represents the parts per million (ppm) accuracy of the assignment. The colour corresponds to the presence of the observed sequence in bovine serum albumin presented in Supplementary Figure 26.

	ya			yb			yc			ya-NH_3		
**Description**	***m/z***	**Assignment**	**Dev. (ppm)**	***m/z***	**Assignment**	**Dev. (ppm)**	***m/z***	**Assignment**	**Dev. (ppm)**	***m/z***	**Assignment**	**Dev. (ppm)**
KKF	x	x	x	428.2626	$C_{21}H_{35}N_5O_3Na^+$	-1.5439	x	x	x	x	x	x
EKKF	529.3107	$C_{25}H_{42}N_6O_5Na^+$	-0.3328	557.3057	$C_{26}H_{42}N_6O_6Na^+$	-0.2425	574.3321	$C_{26}H_{45}N_7O_6Na^+$	-0.3625	512.2841	$C_{25}H_{39}N_5O_5Na^+$	-0.40883
DEKKF	644.3376	$C_{29}H_{47}N_7O_8Na^+$	-0.3680	672.3323	$C_{30}H_{47}N_7O_9Na^+$	-0.6363	689.3592	$C_{30}H_{50}N_8O_9Na^+$	-0.1909	x	x	x
ADEKKF	715.3753	$C_{32}H_{52}N_8O_9Na^+$	0.4660	743.369	$C_{33}H_{52}N_8O_{10}Na^+$	-1.1288	760.3979	$C_{33}H_{55}N_9O_{10}Na^+$	1.9754	x	x	x

Supplementary Table 100 Peak list exported from SurfaceLab spectrum of bovine serum albumin, consisting of ions detected in the spectrum and assigned as sodium adducts of internal fragments of the sequence, KAWSVA. The *m/z* values represent the experimentally observed center mass of each peak. The deviation (dev.) represents the parts per million (ppm) accuracy of the assignment. The colour corresponds to the presence of the observed sequence in bovine serum albumin presented in Supplementary Figure 26.

	ya			yb			yc			ya-NH_3		
**Description**	***m/z***	**Assignment**	**Dev. (ppm)**	***m/z***	**Assignment**	**Dev. (ppm)**	***m/z***	**Assignment**	**Dev. (ppm)**	***m/z***	**Assignment**	**Dev. (ppm)**
WS	270.1213	$C_{13}H_{17}N_3O_2Na^+$	0.0498	298.1162	$C_{14}H_{17}N_3O_3Na^+$	0.0262	315.1427	$C_{14}H_{20}N_4O_3Na^+$	-0.1394	253.0947	$C_{13}H_{14}N_2O_2Na^+$	-0.1205
WSV	369.1895	$C_{18}H_{26}N_4O_3Na^+$	-0.4634	397.1844	$C_{19}H_{26}N_4O_4Na^+$	-0.5907	414.2110	$C_{19}H_{29}N_5O_4Na^+$	-0.4966	352.1631	$C_{18}H_{23}N_3O_3Na^+$	-0.2993
WSVA	440.2265	$C_{21}H_{31}N_5O_4Na^+$	-0.7209	468.2215	$C_{22}H_{31}N_5O_5Na^+$	-0.5507	485.2480	$C_{22}H_{34}N_6O_5Na^+$	-0.5124	423.2001	$C_{21}H_{28}N_4O_4Na^+$	-0.3764
AWSVA	511.2636	$C_{24}H_{36}N_6O_5Na^+$	-0.7363	539.2586	$C_{25}H_{36}N_6O_6Na^+$	-0.4934	556.2852	$C_{25}H_{39}N_7O_6Na^+$	-0.3611	494.2371	$C_{24}H_{33}N_5O_5Na^+$	-0.6711
KAWSVA	639.3583	$C_{30}H_{48}N_8O_6Na^+$	-0.8944	667.3523	$C_{31}H_{48}N_8O_7Na^+$	-2.2938	684.3805	$C_{31}H_{51}N_9O_7Na^+$	0.2052	622.3320	$C_{30}H_{45}N_7O_6Na^+$	-0.5815

Supplementary Table 101 Peak list exported from SurfaceLab spectrum of bovine serum albumin, consisting of ions detected in the spectrum and assigned as sodium adducts of internal fragments of the sequence, NLPPLTA. The sequence is observed as ya, yb, yc and ya-NH_3 ions. The *m/z* values represent the experimentally observed center mass of each peak. The colour corresponds to the presence of the observed sequence in bovine serum albumin presented in Supplementary Figure 26.

	ya			yb			yc			ya-NH_3		
**Description**	***m/z***	**Assignment**	**Dev. (ppm)**	***m/z***	**Assignment**	**Dev. (ppm)**	***m/z***	**Assignment**	**Dev. (ppm)**	***m/z***	**Assignment**	**Dev. (ppm)**
NL	224.1370	$C_9H_{19}N_3O_2Na^+$	0.1761	252.1319	$C_{10}H_{19}N_3O_3Na^+$	-0.0484	x	x	x	207.1104	$C_9H_{16}N_2O_2Na^+$	-0.1653
NLP	321.1897	$C_{14}H_{26}N_4O_3Na^+$	-0.1324	349.1845	$C_{15}H_{26}N_4O_4Na^+$	-0.2465	366.2112	$C_{15}H_{29}N_5O_4Na^+$	0.0049	304.1632	$C_{14}H_{23}N_3O_3Na^+$	-0.0205
NLPP	418.2423	$C_{19}H_{33}N_5O_4Na^+$	-0.3145	446.2373	$C_{20}H_{33}N_5O_5Na^+$	-0.2253	463.2640	$C_{20}H_{36}N_6O_5Na^+$	0.1472	401.2158	$C_{19}H_{30}N_4O_4Na^+$	-0.3495
NLPPL	531.3264	$C_{25}H_{44}N_6O_5Na^+$	-0.1727	559.3213	$C_{26}H_{44}N_6O_6Na^+$	-0.2342	576.3480	$C_{26}H_{47}N_7O_6Na^+$	-0.0113	514.2997	$C_{25}H_{41}N_5O_5Na^+$	-0.6495
NLPPLT	632.3741	$C_{29}H_{51}N_7O_7Na^+$	-0.1954	660.3688	$C_{30}H_{51}N_7O_8Na^+$	-0.4899	677.3954	$C_{30}H_{54}N_8O_8Na^+$	-0.4867	615.3473	$C_{29}H_{48}N_6O_7Na^+$	-0.5796
NLPPLTA	703.4108	$C_{32}H_{56}N_8O_8Na^+$	-0.8049	731.4063	$C_{33}H_{56}N_8O_9Na^+$	0.0712	x	x	x	686.3844	$C_{32}H_{53}N_7O_8Na^+$	-0.5687

Supplementary Table 102 Peak list exported from SurfaceLab spectrum of bovine serum albumin, consisting of ions detected in the spectrum and assigned as C-terminal sequence VVSTQTALA. The colour corresponds to the presence of the observed sequence in bovine serum albumin presented in Supplementary Figure 26.

	y			z			z+2		
**Description**	***m/z***	**Assignment**	**Dev. (ppm)**	***m/z***	**Assignment**	**Dev. (ppm)**	***m/z***	**Assignment**	**Dev. (ppm)**
TALA	376.2318	$C_{16}H_{32}N_4O_6^+$	0.3756	359.2052	$C_{16}H_{29}N_3O_6^+$	0.3829	361.2209	$C_{16}H_{31}N_3O_6^+$	0.4676
QTALA	506.3067	$C_{21}H_{42}N_6O_8^+$	1.7283	489.2795	$C_{21}H_{39}N_5O_8^+$	0.3830	491.2950	$C_{21}H_{41}N_5O_8^+$	-0.0045
TQTALA	x	x	x	590.3272	$C_{25}H_{46}N_6O_{10}^+$	0.3040	592.3435	$C_{25}H_{48}N_6O_{10}^+$	1.3877
STQTALA	x	x	x	x	x	x	x	x	x
VSTQTALA	x	x	x	x	x	x	677.3591	$C_{28}H_{51}N_7O_{12}^+$	0.0806

Supplementary Table 103 Peak list exported from SurfaceLab spectrum of bovine serum albumin, consisting of ions detected in the spectrum and assigned as fragments of N-terminal sequence GPKLVVSTQTALA. The colour corresponds to the presence of the observed sequence in bovine serum albumin presented in Supplementary Figure 26.

	y			z+1		
**Description**	***m/z***	**Assignment**	**Dev. (ppm)**	***m/z***	**Assignment**	**Dev. (ppm)**
ALA	296.1580	$C_{12}H_{23}N_3O_4Na^+$	-0.3286			
TALA						
QTALA	525.2642	$C_{21}H_{38}N_6O_8Na^+$	-0.3240	510.2525	$C_{21}H_{37}N_5O_8Na^+$	-1.8945
TQTALA	626.3119	$C_{25}H_{45}N_7O_{10}Na^+$	-0.1256	611.3011	$C_{25}H_{44}N_6O_{10}Na^+$	0.0360
STQTALA	713.3435	$C_{28}H_{50}N_8O_{12}Na^+$	-0.7932	698.3334	$C_{28}H_{49}N_7O_{12}Na^+$	0.3319
VSTQTALA	812.4114	$C_{33}H_{59}N_9O_{13}Na^+$	-1.2803	797.4005	$C_{33}H_{58}N_8O_{13}Na^+$	-1.3659
VVSTQTALA	911.4806	$C_{38}H_{68}N_{10}O_{14}Na^+$	-0.2717	896.4690	$C_{38}H_{67}N_9O_{14}Na^+$	-1.0667
LVVSTQTALA	1024.5647	$C_{44}H_{79}N_{11}O_{15}Na^+$	-0.2360	1009.5543	$C_{44}H_{78}N_{10}O_{15}Na^+$	0.2717
KLVVSTQTALA	1152.6591	$C_{50}H_{91}N_{13}O_{16}Na^+$	-0.6906			
PKLVVSTQTALA	1249.7122	$C_{55}H_{98}N_{14}O_{17}Na^+$	-0.3296			
GPKLVVSTQTALA	1306.7336	$C_{57}H_{101}N_{15}O_{18}Na^+$	-0.4355			

Sequences present in both BSA and HSA:

Supplementary Table 104 Peak list exported from SurfaceLab spectrum of bovine serum albumin, consisting of ions detected in the spectrum and assigned fragments of N-terminal sequence DTHKSEI. The *m/z* values represent the experimentally observed center mass of each peak. The deviation (dev.) represents the parts per million (ppm) accuracy of the assignment. The colour corresponds to the presence of the observed sequence in human serum albumin presented in Supplementary Figure 26.

	**a**			**b**			**b-NH3**			**a-NH3**		
**Description**	***m/z***	**Assignment**	**Dev. (ppm)**	***m/z***	**Assignment**	**Dev. (ppm)**	***m/z***	**Assignment**	**Dev. (ppm)**	***m/z***	**Assignment**	**Dev. (ppm)**
KD							251.1002	$C_{10}H_{16}N_2O_4Na^+$	-0.2736	223.1053	$C_9H_{16}N_2O_3Na^+$	0.1182
KDL							364.1843	$C_{16}H_{27}N_3O_5Na^+$	-0.0202	336.1892	$C_{15}H_{27}N_3O_4Na^+$	-0.3862
KDLG	410.2370	$C_{17}H_{33}N_5O_5Na^+$	-0.9112				421.2058	$C_{18}H_{30}N_4O_6Na^+$	0.0501	393.2106	$C_{17}H_{30}N_4O_5Na^+$	-0.5139
KDLGE										522.2533	$C_{22}H_{37}N_5O_8Na^+$	-0.3155
KDLGEE	668.3226	$C_{27}H_{47}N_7O_{11}Na^+$	0.1008	696.3164	$C_{28}H_{47}N_7O_{12}Na^+$	-1.5101						

Supplementary Table 105 Peak list exported from SurfaceLab spectrum of bovine serum albumin, consisting of ions detected in the spectrum and assigned fragments of N-terminal sequence DTHKSEI. The sequence is observed as a-NH_3 ions. The *m/z* values represent the experimentally observed center mass of each peak. The deviation (dev.) represents the parts per million (ppm) accuracy of the assignment. The colour corresponds to the presence of the observed sequence in human serum albumin presented in Supplementary Figure 26.

	a			b			b-NH3			a-NH3		
**Description**	***m/z***	**Assignment**	**Dev. (ppm)**	***m/z***	**Assignment**	**Dev. (ppm)**	***m/z***	**Assignment**	**Dev. (ppm)**	***m/z***	**Assignment**	**Dev. (ppm)**
HG				219.0852	$C_8H_{12}N_4O_2Na^+$	-0.0789						
HGD	306.1173	$C_{11}H_{17}N_5O_4Na^+$	0.1852	334.1120	$C_{12}H_{17}N_5O_5Na^+$	-0.4325				289.0907	$C_{11}H_{14}N_4O_4Na^+$	-0.1545
HGDL	419.2014	$C_{17}H_{28}N_6O_5Na^+$	0.0310	447.1965	$C_{18}H_{28}N_6O_6Na^+$	0.4396	430.1698	$C_{18}H_{25}N_5O_6Na^+$	0.1979	402.1747	$C_{17}H_{25}N_5O_5Na^+$	-0.1044
HGDLL	532.2855	$C_{23}H_{39}N_7O_6Na^+$	0.2477	560.2801	$C_{24}H_{39}N_7O_7Na^+$	-0.3291	543.2535	$C_{24}H_{36}N_6O_7Na^+$	-0.5599	515.2587	$C_{23}H_{36}N_6O_6Na^+$	-0.3785
HGDLLE	661.3284	$C_{28}H_{46}N_8O_9Na^+$	0.6056	689.3245	$C_{29}H_{46}N_8O_{10}Na^+$	2.2503				644.3012	$C_{28}H_{43}N_7O_9Na^+$	-0.3589
HGDLLEC-SH_2				758.3444	$C_{32}H_{49}N_9O_{11}Na^+$	0.0490						

Supplementary Table 106 Peak list exported from SurfaceLab spectrum of bovine serum albumin, consisting of ions detected in the spectrum and assigned fragments of N-terminal sequence DTHKSEI. The sequence is observed as a-NH_3 ions. The *m/z* values represent the experimentally observed center mass of each peak. The deviation (dev.) represents the parts per million (ppm) accuracy of the assignment. The colour corresponds to the presence of the observed sequence in human serum albumin presented in Supplementary Figure 26.

	a			b			a-NH3		
**Description**	***m/z***	**Assignment**	**Dev. (ppm)**	***m/z***	**Assignment**	**Dev. (ppm)**	***m/z***	**Assignment**	**Dev. (ppm)**
LEC-SH2	308.1580	$C_{13}H_{23}N_3O_4Na^+$	-0.1061	336.1530	$C_{14}H_{23}N_3O_5Na^+$	-0.0650	291.1315	$C_{13}H_{20}N_2O_4Na^+$	-0.0534
LECA-SH2	379.1950	$C_{16}H_{28}N_4O_5Na^+$	-0.4092	407.1900	$C_{17}H_{28}N_4O_6Na^+$	-0.2853	362.1685	$C_{16}H_{25}N_3O_5Na^+$	-0.3521
LECAD-SH2	494.2220	$C_{20}H_{33}N_5O_8Na^+$	-0.3024				477.1954	$C_{20}H_{30}N_4O_8Na^+$	-0.4867

Supplementary Table 107 Peak list exported from SurfaceLab spectrum of bovine serum albumin, consisting of ions detected in the spectrum and assigned fragments of N-terminal sequence DTHKSEI. The sequence is observed as a-NH_3 ions. The *m/z* values represent the experimentally observed center mass of each peak. The deviation (dev.) represents the parts per million (ppm) accuracy of the assignment. The colour corresponds to the presence of the observed sequence in human serum albumin presented in Supplementary Figure 26.

	a			b			b-NH3			a-NH3		
**Description**	***m/z***	**Assignment**	**Dev. (ppm)**	***m/z***	**Assignment**	**Dev. (ppm)**	***m/z***	**Assignment**	**Dev. (ppm)**	***m/z***	**Assignment**	**Dev. (ppm)**
LH	247.1530	$C_{11}H_{20}N_4ONa^+$	0.2142	275.1479	$C_{12}H_{20}N_4O_2Na^+$	0.0613	258.1213	$C_{12}H_{17}N_3O_2Na^+$	0.0555	230.1265	$C_{11}H_{17}N_3ONa^+$	0.5891
LHT	348.2006	$C_{15}H_{27}N_5O_3Na^+$	-0.1535	376.1954	$C_{16}H_{27}N_5O_4Na^+$	-0.3682	359.1689	$C_{16}H_{24}N_4O_4Na^+$	-0.3265	331.1740	$C_{15}H_{24}N_4O_3Na^+$	-0.1167
LHTL	459.2686	$C_{21}H_{36}N_6O_4Na^+$	-0.8610	487.2637	$C_{22}H_{36}N_6O_5Na^+$	-0.4079	470.2373	$C_{22}H_{33}N_5O_5Na^+$	-0.2759	442.2421	$C_{21}H_{33}N_5O_4Na^+$	-0.8419
LHTLF	608.3525	$C_{30}H_{47}N_7O_5Na^+$	-0.9579	636.3474	$C_{31}H_{47}N_7O_6Na^+$	-0.9513						
LHTLFG	665.3745	$C_{32}H_{50}N_8O_6Na^+$	-0.0196	693.3681	$C_{33}H_{50}N_8O_7Na^+$	-2.0264	676.3429	$C_{33}H_{47}N_7O_7Na^+$	0.0380	648.3465	$C_{32}H_{47}N_7O_6Na^+$	-2.3424
LHTLFGD	780.4009	$C_{36}H_{55}N_9O_9Na^+$	-0.7423									
LHTLFGDE	909.4421	$C_{41}H_{62}N_{10}O_{12}Na^+$	-2.1397									
LHTLFGDEL							1,033.50	$C_{48}H_{70}N_{10}O_{14}Na^+$	0.9073			

Supplementary Table 108 Peak list exported from SurfaceLab spectrum of human serum albumin, consisting of ions detected in the spectrum and assigned as sodium adducts of internal fragments of the sequence PQNLIK. The *m/z* values represent the experimentally observed center mass of each peak. The deviation (dev.) represents the parts per million (ppm) accuracy of the assignment. The colour corresponds to the presence of the observed sequence in human serum albumin presented in Supplementary Figure 26.

	a			b			b-NH3			a-NH3		
**Description**	***m/z***	**Assignment**	**Dev. (ppm)**	***m/z***	**Assignment**	**Dev. (ppm)**	***m/z***	**Assignment**	**Dev. (ppm)**	***m/z***	**Assignment**	**Dev. (ppm)**
PQ	222.1215	$C_9H_{17}N_3O_2Na^+$	0.8445	250.1163	$C_{10}H_{17}N_3O_3Na^+$	0.4615	233.0899	$C_{10}H_{14}N_2O_3Na^+$	0.9107	205.0948	$C_9H_{14}N_2O_2Na^+$	0.2325
PQN	336.1644	$C_{13}H_{23}N_5O_4Na^+$	0.4871	364.1594	$C_{14}H_{23}N_5O_5Na^+$	0.6608	347.1328	$C_{14}H_{20}N_4O_5Na^+$	0.5448	319.1379	$C_{13}H_{20}N_4O_4Na^+$	0.6770
PQNL	449.2490	$C_{19}H_{34}N_6O_5Na^+$	1.5244	477.2438	$C_{20}H_{34}N_6O_6Na^+$	1.1650	460.2167	$C_{20}H_{31}N_5O_6Na^+$	0.1943	432.2220	$C_{19}H_{31}N_5O_5Na^+$	0.5452
PQNLI	562.3329	$C_{25}H_{45}N_7O_6Na^+$	1.0015	590.3280	$C_{26}H_{45}N_7O_7Na^+$	1.2917	573.2999	$C_{26}H_{42}N_6O_7Na^+$	-1.4596	545.3063	$C_{25}H_{42}N_6O_6Na^+$	0.8796
PQNLIK	0.0000						701.3960	$C_{32}H_{54}N_8O_8Na^+$	0.3849			

Supplementary Table 109 Peak list exported from SurfaceLab spectrum of human serum albumin, consisting of ions detected in the spectrum and assigned as sodium adducts of internal fragments of the sequence QYLQQC. The *m/z* values represent the experimentally observed center mass of each peak. The deviation (dev.) represents the parts per million (ppm) accuracy of the assignment. The colour corresponds to the presence of the observed sequence in human serum albumin presented in Supplementary Figure 26.

	a			b			c			a-NH3		
**Description**	***m/z***	**Assignment**	**Dev. (ppm)**	***m/z***	**Assignment**	**Dev. (ppm)**	***m/z***	**Assignment**	**Dev. (ppm)**	***m/z***	**Assignment**	**Dev. (ppm)**
QY	288.1320	$C_{13}H_{19}N_3O_3Na^+$	0.5724	316.1270	$C_{14}H_{19}N_3O_4Na^+$	0.8270	333.1536	$C_{14}H_{22}N_4O_4Na^+$	0.6931			
QYL	401.2163	$C_{19}H_{30}N_4O_4Na^+$	0.8183	429.2110	$C_{20}H_{30}N_4O_5Na^+$	0.3802				384.1896	$C_{19}H_{27}N_3O_4Na^+$	0.6274
QYLQ	529.2749	$C_{24}H_{38}N_6O_6Na^+$	0.7683	557.2688	$C_{25}H_{38}N_6O_7Na^+$	-1.0541	574.2963	$C_{25}H_{41}N_7O_7Na^+$	0.6584	512.2480	$C_{24}H_{35}N_5O_6Na^+$	0.1444
QYLQQ	657.3334	$C_{29}H_{46}N_8O_8Na^+$	0.4235	685.3287	$C_{30}H_{46}N_8O_9Na^+$	1.0414						
QYLQQC -S	0.0000			756.3643	$C_{33}H_{51}N_9O_{10}Na^+$	-1.0192						

Sequences specific to human serum albumin (HSA):

Supplementary Table 110 Peak list exported from SurfaceLab spectrum of human serum albumin, consisting of ions detected in the spectrum and assigned as sodium adducts of N-terminal sequence DAHKS. The *m/z* values represent the experimentally observed center mass of each peak. The deviation (dev.) represents the parts per million (ppm) accuracy of the assignment. The colour corresponds to the presence of the observed sequence in human serum albumin presented in Supplementary Figure 26.

	a			b			c			a-NH3		
**Description**	***m/z***	**Assignment**	**Dev. (ppm)**	***m/z***	**Assignment**	**Dev. (ppm)**	***m/z***	**Assignment**	**Dev. (ppm)**	***m/z***	**Assignment**	**Dev. (ppm)**
DA				209.0533	$C_7H_{10}N_2O_4Na^+$	0.1037						
DAH	318.1175	$C_{12}H_{17}N_5O_4Na^+$	0.5547	346.1126	$C_{13}H_{17}N_5O_5Na^+$	1.1748	363.1390	$C_{13}H_{20}N_6O_5Na^+$	0.7188			
DAHK	446.2125	$C_{18}H_{29}N_7O_5Na^+$	0.4938	474.2077	$C_{19}H_{29}N_7O_6Na^+$	1.2106				429.1859	$C_{18}H_{26}N_6O_5Na^+$	0.3876
DAHKS	533.2452	$C_{21}H_{34}N_8O_7Na^+$	1.8120									

Supplementary Table 111 Peak list exported from SurfaceLab spectrum of human serum albumin, consisting of ions detected in the spectrum and assigned as sodium adducts of internal fragments of the sequence LHEKTP. The *m/z* values represent the experimentally observed center mass of each peak. The deviation (dev.) represents the parts per million (ppm) accuracy of the assignment. The colour corresponds to the presence of the observed sequence in human serum albumin presented in Supplementary Figure 26.

	a			b			b-NH3			a-NH3		
**Description**	***m/z***	**Assignment**	**Dev. (ppm)**	***m/z***	**Assignment**	**Dev. (ppm)**	***m/z***	**Assignment**	**Dev. (ppm)**	***m/z***	**Assignment**	**Dev. (ppm)**
LH				275.1480	$C_{12}H_{20}N_4O_2Na^+$	0.5898	258.1215	$C_{12}H_{17}N_3O_2Na^+$	0.6062	230.1265	$C_{11}H_{17}N_3ONa^+$	0.4545
LHE	376.1956	$C_{16}H_{27}N_5O_4Na^+$	0.2255	404.1906	$C_{17}H_{27}N_5O_5Na^+$	0.5061	387.1641	$C_{17}H_{24}N_4O_5Na^+$	0.4296	359.1691	$C_{16}H_{24}N_4O_4Na^+$	0.4819
LHEK	504.2920	$C_{22}H_{39}N_7O_5Na^+$	3.0937	532.2862	$C_{23}H_{39}N_7O_6Na^+$	1.5463	515.2593	$C_{23}H_{36}N_6O_6Na^+$	0.7867	487.2641	$C_{22}H_{36}N_6O_5Na^+$	0.3681
LHEKT	605.3387	$C_{26}H_{46}N_8O_7Na^+$	0.8187	633.3346	$C_{27}H_{46}N_8O_8Na^+$	2.3442	616.3064	$C_{27}H_{43}N_7O_8Na^+$	-0.2820	588.3123	$C_{26}H_{43}N_7O_7Na^+$	1.2365
LHEKTP										685.3650	$C_{31}H_{50}N_8O_8Na^+$	0.8906

L-lactate dehydrogenase

a)

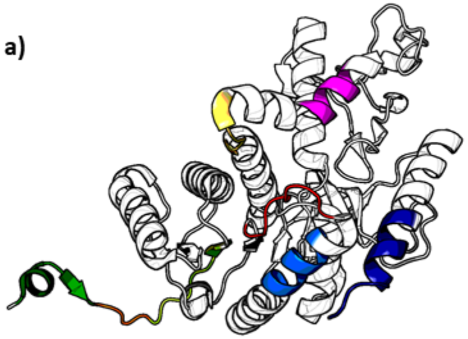

b)

Uniprot database code	Amino acid sequence
LDHA_RABIT1n	AALKDQLIHNLLKEEHVPQNKITVVGVGAVGMACAISILMKDLADELALVDVM<br>EDKLKGEMMDLQHGSLFLRTPKIVSGKDYSVTANSKLVIITAGARQQEGESR<br>LNLVQRNVNIFKFIIPNVVKYSPHCKLLVVSNPVDILTYVAWKISGFPKNRVIGS<br>GCNLDSARFRYLMGERLGVHALSCHGWILGEHGDSSVPVWSGMNVAGVSL<br>KTLHPELGTDADKEQWKQVHKQVVDSAYEVIKLKGYTTWAIGLSVADLAESI<br>MKNLRRVHPISTMLKGLYGIKEDVFLSVPCVLGQNGISDVVKVTLTSEEEAHL<br>KKSADTLWGIQKELQF

Supplementary Figure 27 L-lactate dehydrogenase (a) cartoon exported from PDB entry 5NQB[18] and (b) amino acid sequence exported from the UniProt database. The highlighted colours correspond to assigned segments of the amino acid sequence, presented in Supplementary Tables 112-123.

Supplementary Table 112 Peak list exported from SurfaceLab spectrum of L-lactate dehydrogenase, consisting of ions detected in the spectrum and assigned internal fragments of the sequence AGARQQEGE. The *m/z* values represent the experimentally observed center mass of each peak. The deviation (dev.) represents the parts per million (ppm) accuracy of the assignment. The colour corresponds to the presence of the observed sequence in L-lactate dehydrogenase presented in Supplementary Figure 27.

	a			b			b-NH3			a-NH3		
**Description**	***m/z***	**Assignment**	**Dev. (ppm)**	***m/z***	**Assignment**	**Dev. (ppm)**	***m/z***	**Assignment**	**Dev. (ppm)**	***m/z***	**Assignment**	**Dev. (ppm)**
AGA	172.1078	$C_7H_{14}N_3O_2^+$	-1.6293	200.1027	$C_8H_{14}N_3O_3^+$	-1.1218	183.0763	$C_8H_{11}N_2O_3^+$	-0.9014	155.0813	$C_7H_{11}N_2O_2^+$	-1.4005
AGAR	328.2091	$C_{13}H_{26}N_7O_3^+$	-0.0823	356.2040	$C_{14}H_{26}N_7O_4^+$	-0.1577						
AGARQ	456.2677	$C_{18}H_{34}N_9O_5^+$	-0.0348									
AGARQQ	584.3263	$C_{23}H_{42}N_{11}O_7^+$	0.0480	612.3215	$C_{24}H_{42}N_{11}O_8^+$	0.4832						
AGARQQE	713.3693	$C_{28}H_{49}N_{12}O_{10}^+$	0.5677									
AGARQQEG	770.3923	$C_{30}H_{52}N_{13}O_{11}^+$	2.5450	798.3828	$C_{31}H_{52}N_{13}O_{12}^+$	-3.0875						
AGARQQEGE	899.4328	$C_{35}H_{59}N_{14}O_{14}^+$	-0.2051									

Supplementary Table 113 Peak list exported from SurfaceLab spectrum of L-lactate dehydrogenase, consisting of ions detected in the spectrum and assigned internal fragments of the sequence ALKDQLIH. The *m/z* values represent the experimentally observed center mass of each peak. The deviation (dev.) represents the parts per million (ppm) accuracy of the assignment. The colour corresponds to the presence of the observed sequence in L-lactate dehydrogenase presented in Supplementary Figure 27.

	a			b			b-NH3			a-NH3		
**Description**	***m/z***	**Assignment**	**Dev. (ppm)**	***m/z***	**Assignment**	**Dev. (ppm)**	***m/z***	**Assignment**	**Dev. (ppm)**	***m/z***	**Assignment**	**Dev. (ppm)**
AL	157.1334	$C_8H_{17}N_2O^+$	-1.1563	185.1283	$C_9H_{17}N_2O_2^+$	-1.0649	168.1017	$C_9H_{14}NO_2^+$	-1.4951			
ALK				313.2234	$C_{15}H_{29}N_4O_3^+$	-0.0941	296.1969	$C_{15}H_{26}N_3O_3^+$	0.0395	268.2020	$C_{14}H_{26}N_3O_2^+$	0.1740
ALKD				428.2504	$C_{19}H_{34}N_5O_6^+$	0.0555	411.2233	$C_{19}H_{31}N_4O_6^+$	-1.2977	383.2287	$C_{18}H_{31}N_4O_5^+$	-0.6014
ALKDQ				556.3090	$C_{24}H_{42}N_7O_8^+$	0.1673	539.2819	$C_{24}H_{39}N_6O_8^+$	-0.9170			
ALKDQL				669.3932	$C_{30}H_{53}N_8O_9^+$	0.3306						
ALKDQLI				782.4770	$C_{36}H_{64}N_9O_{10}^+$	-0.1427						
ALKDQLIH				919.5359	$C_{42}H_{71}N_{12}O_{11}^+$	-0.0768						

Supplementary Table 114 Peak list exported from SurfaceLab spectrum of L-lactate dehydrogenase, consisting of ions detected in the spectrum and assigned internal fragments of the sequence DQLIHNLL. The *m/z* values represent the experimentally observed center mass of each peak. The deviation (dev.) represents the parts per million (ppm) accuracy of the assignment. The colour corresponds to the presence of the observed sequence in L-lactate dehydrogenase presented in Supplementary Figure 27.

	a			b			c			a-NH3		
**Description**	***m/z***	**Assignment**	**Dev. (ppm)**	***m/z***	**Assignment**	**Dev. (ppm)**	***m/z***	**Assignment**	**Dev. (ppm)**	***m/z***	**Assignment**	**Dev. (ppm)**
DQ				244.0929	$C_9H_{14}N_3O_5^+$	0.3573				199.0712	$C_8H_{11}N_2O_4^+$	-0.8408
DQL	329.1818	$C_{14}H_{25}N_4O_5^+$	-0.3690	357.1768	$C_{15}H_{25}N_4O_6^+$	-0.2513				312.1553	$C_{14}H_{22}N_3O_5^+$	-0.2590
DQLI				470.2615	$C_{21}H_{36}N_5O_7^+$	1.3173				425.2394	$C_{20}H_{33}N_4O_6^+$	-0.1288
DQLIH										562.2976	$C_{26}H_{40}N_7O_7^+$	-1.4119
DQLIHN	693.3681	$C_{30}H_{49}N_{10}O_9^+$	0.3696									
DQLIHNL	806.4512	$C_{36}H_{60}N_{11}O_{10}^+$	-0.8327									
DQLIHNLL	919.5359	$C_{42}H_{71}N_{12}O_{11}^+$	-0.0768									

Supplementary Table 115 Peak list exported from SurfaceLab spectrum of L-lactate dehydrogenase, consisting of ions detected in the spectrum and assigned internal fragments of the sequence EHVPQNKITV. The *m/z* values represent the experimentally observed center mass of each peak. The deviation (dev.) represents the parts per million (ppm) accuracy of the assignment. The colour corresponds to the presence of the observed sequence in L-lactate dehydrogenase presented in Supplementary Figure 27.

	a			b			c			a-NH3		
**Description**	***m/z***	**Assignment**	**Dev. (ppm)**	***m/z***	**Assignment**	**Dev. (ppm)**	***m/z***	**Assignment**	**Dev. (ppm)**	***m/z***	**Assignment**	**Dev. (ppm)**
EH	241.1295	$C_{10}H_{17}N_4O_3^+$	-0.2191	269.1244	$C_{11}H_{17}N_4O_4^+$	-0.1571	286.1509	$C_{11}H_{20}N_5O_4^+$	-0.2720	224.1030	$C_{10}H_{14}N_3O_3^+$	0.0469
EHV	340.1979	$C_{15}H_{26}N_5O_4^+$	-0.0090	368.1928	$C_{16}H_{26}N_5O_5^+$	-0.0053	385.2196	$C_{16}H_{29}N_6O_5^+$	0.4473	323.1714	$C_{15}H_{23}N_4O_4^+$	0.1051
EHVP	437.2510	$C_{20}H_{33}N_6O_5^+$	0.6823	465.2455	$C_{21}H_{33}N_6O_6^+$	-0.2483	482.2724	$C_{21}H_{36}N_7O_6^+$	0.5072	420.2243	$C_{20}H_{30}N_5O_5^+$	0.2966
EHVPQ	565.3091	$C_{25}H_{41}N_8O_7^+$	-0.3212							548.2818	$C_{25}H_{38}N_7O_7^+$	-1.7455
EHVPQN	679.3544	$C_{29}H_{47}N_{10}O_9^+$	3.2531							662.3258	$C_{29}H_{44}N_9O_9^+$	0.2994
EHVPQNK	807.4472	$C_{35}H_{59}N_{12}O_{10}^+$	0.0265							790.4223	$C_{35}H_{56}N_{11}O_{10}^+$	2.1895
EHVPQNKI										903.5048	$C_{41}H_{67}N_{12}O_{11}^+$	0.1027
EHVPQNKIT												
EHVPQNKITV	1118.6309	$C_{50}H_{84}N_{15}O_{14}^+$	-0.6456	1146.6262	$C_{51}H_{84}N_{15}O_{15}^+$	-0.3369	1163.6512	$C_{51}H_{87}N_{16}O_{15}^+$	-1.6787	1101.6041	$C_{50}H_{81}N_{14}O_{14}^+$	-0.8987

Supplementary Table 116 Peak list exported from SurfaceLab spectrum of L-lactate dehydrogenase, consisting of ions detected in the spectrum and assigned internal fragments of the sequence KGYTT. The *m/z* values represent the experimentally observed center mass of each peak. The deviation (dev.) represents the parts per million (ppm) accuracy of the assignment. The colour corresponds to the presence of the observed sequence in L-lactate dehydrogenase presented in Supplementary Figure 27.

	a			b			c			a-NH3		
**Description**	***m/z***	**Assignment**	**Dev. (ppm)**	***m/z***	**Assignment**	**Dev. (ppm)**	***m/z***	**Assignment**	**Dev. (ppm)**	***m/z***	**Assignment**	**Dev. (ppm)**
KG	158.1286	$C_7H_{16}N_3O^+$	-0.8996	186.1235	$C_8H_{16}N_3O_2^+$	-1.0053	203.1504	$C_8H_{19}N_4O_2^+$	0.5374			
KGY	321.1922	$C_{16}H_{25}N_4O_3^+$	0.1657	349.1870	$C_{17}H_{25}N_4O_4^+$	-0.0421	366.2137	$C_{17}H_{28}N_5O_4^+$	0.2032	304.1656	$C_{16}H_{22}N_3O_3^+$	0.0252
KGYT	422.2396	$C_{20}H_{32}N_5O_5^+$	-0.3594	450.2347	$C_{21}H_{32}N_5O_6^+$	-0.0308	467.2614	$C_{21}H_{35}N_6O_6^+$	0.2812	405.2131	$C_{20}H_{29}N_4O_5^+$	-0.3825
KGYTT	523.2875	$C_{24}H_{39}N_6O_7^+$	0.1377				568.3083	$C_{25}H_{42}N_7O_8^+$	-1.0997			

Supplementary Table 117 Peak list exported from SurfaceLab spectrum of L-lactate dehydrogenase, consisting of ions detected in the spectrum and assigned internal fragments of the sequence HNLLKEE. The *m/z* values represent the experimentally observed center mass of each peak. The deviation (dev.) represents the parts per million (ppm) accuracy of the assignment. The colour corresponds to the presence of the observed sequence in L-lactate dehydrogenase presented in Supplementary Figure 27.

	a			b			c			a-NH3		
**Description**	***m/z***	**Assignment**	**Dev. (ppm)**	***m/z***	**Assignment**	**Dev. (ppm)**	***m/z***	**Assignment**	**Dev. (ppm)**	***m/z***	**Assignment**	**Dev. (ppm)**
HN	224.1142	$C_9H_{14}N_5O_2^+$	-0.2055	252.1091	$C_{10}H_{14}N_5O_3^+$	-0.1605	269.1357	$C_{10}H_{17}N_6O_3^+$	-0.0220	207.0875	$C_9H_{11}N_4O_2^+$	-0.9548
HNL	337.1982	$C_{15}H_{25}N_6O_3^+$	-0.1221	365.1932	$C_{16}H_{25}N_6O_4^+$	-0.0094	382.2197	$C_{16}H_{28}N_7O_4^+$	-0.0422	320.1717	$C_{15}H_{22}N_5O_3^+$	0.0421
HNLL	450.2824	$C_{21}H_{36}N_7O_4^+$	0.1460	478.2772	$C_{22}H_{36}N_7O_5^+$	-0.0664	495.3036	$C_{22}H_{39}N_8O_5^+$	-0.3952	433.2557	$C_{21}H_{33}N_6O_4^+$	-0.2795
HNLLK				606.3717	$C_{28}H_{48}N_9O_6^+$	-0.7989				561.3503	$C_{27}H_{45}N_8O_5^+$	-0.8236
HNLLKE				735.4151	$C_{33}H_{55}N_{10}O_9^+$	0.4495				690.3937	$C_{32}H_{52}N_9O_8^+$	0.5519
HNLLKEE				864.4564	$C_{38}H_{62}N_{11}O_{12}^+$	-1.1462				819.4365	$C_{37}H_{59}N_{10}O_{11}^+$	0.7218

Supplementary Table 118 Peak list exported from SurfaceLab spectrum of L-lactate dehydrogenase, consisting of ions detected in the spectrum and assigned internal fragments of the sequence LKEEHVP. The *m/z* values represent the experimentally observed center mass of each peak. The deviation (dev.) represents the parts per million (ppm) accuracy of the assignment. The colour corresponds to the presence of the observed sequence in L-lactate dehydrogenase presented in Supplementary Figure 27.

	a			b			c			a-NH3		
**Description**	***m/z***	**Assignment**	**Dev. (ppm)**	***m/z***	**Assignment**	**Dev. (ppm)**	***m/z***	**Assignment**	**Dev. (ppm)**	***m/z***	**Assignment**	**Dev. (ppm)**
LK	214.1912	$C_{11}H_{24}N_3O^+$	-0.7010	242.1863	$C_{12}H_{24}N_3O_2^+$	-0.1306	259.2130	$C_{12}H_{27}N_4O_2^+$	0.3940	197.1647	$C_{11}H_{21}N_2O^+$	-0.9105
LKE				371.2289	$C_{17}H_{31}N_4O_5^+$	0.0162				326.2074	$C_{16}H_{28}N_3O_4^+$	0.0277
LKEE										455.2511	$C_{21}H_{35}N_4O_7^+$	2.4104
LKEEH	609.3353	$C_{27}H_{45}N_8O_8^+$	-0.3760	637.3301	$C_{28}H_{45}N_8O_9^+$	-0.5222				592.3090	$C_{27}H_{42}N_7O_8^+$	0.0617
LKEEHV	708.4034	$C_{32}H_{54}N_9O_9^+$	-0.6444	736.3983	$C_{33}H_{54}N_9O_{10}^+$	-0.6815				691.3772	$C_{32}H_{51}N_8O_9^+$	-0.2219
LKEEHVP										788.4280	$C_{37}H_{58}N_9O_{10}^+$	-2.7127

Supplementary Table 119 Peak list exported from SurfaceLab spectrum of L-lactate dehydrogenase, consisting of ions detected in the spectrum and assigned internal fragments of the sequence VHKQVVD. The *m/z* values represent the experimentally observed center mass of each peak. The deviation (dev.) represents the parts per million (ppm) accuracy of the assignment. The colour corresponds to the presence of the observed sequence in L-lactate dehydrogenase presented in Supplementary Figure 27.

	a			b			c			a-NH3		
**Description**	***m/z***	**Assignment**	**Dev. (ppm)**	***m/z***	**Assignment**	**Dev. (ppm)**	***m/z***	**Assignment**	**Dev. (ppm)**	***m/z***	**Assignment**	**Dev. (ppm)**
VH	209.1396	$C_{10}H_{17}N_4O^+$	-0.3548	237.1346	$C_{11}H_{17}N_4O_2^+$	0.0195	254.1611	$C_{11}H_{20}N_5O_2^+$	-0.1985	192.1129	$C_{10}H_{14}N_3O^+$	-1.2326
VHK				365.2296	$C_{17}H_{29}N_6O_3^+$	0.1447				320.2082	$C_{16}H_{26}N_5O_2^+$	0.1578
VHKQ	465.2933	$C_{21}H_{37}N_8O_4^+$	0.2278	493.2882	$C_{22}H_{37}N_8O_5^+$	0.1406	510.3144	$C_{22}H_{40}N_9O_5^+$	-0.6615	448.2669	$C_{21}H_{34}N_7O_4^+$	0.3982
VHKQV	564.3618	$C_{26}H_{46}N_9O_5^+$	0.3560	592.3561	$C_{27}H_{46}N_9O_6^+$	-0.6910						
VHKQVV	663.4297	$C_{31}H_{55}N_{10}O_6^+$	-0.4816	691.4249	$C_{32}H_{55}N_{10}O_7^+$	-0.1183	708.4514	$C_{32}H_{58}N_{11}O_7^+$	-0.1985			
VHKQVVD				806.4512	$C_{36}H_{60}N_{11}O_{10}^+$	-0.8327				761.4290	$C_{35}H_{57}N_{10}O_9^+$	-1.8577

Supplementary Table 120 Peak list exported from SurfaceLab spectrum of L-lactate dehydrogenase, consisting of ions detected in the spectrum and assigned internal fragments of the sequence LVQRNVN. The *m/z* values represent the experimentally observed center mass of each peak. The deviation (dev.) represents the parts per million (ppm) accuracy of the assignment. The colour corresponds to the presence of the observed sequence in L-lactate dehydrogenase presented in Supplementary Figure 27.

	a			b			c			a-NH3		
**Description**	***m/z***	**Assignment**	**Dev. (ppm)**	***m/z***	**Assignment**	**Dev. (ppm)**	***m/z***	**Assignment**	**Dev. (ppm)**	***m/z***	**Assignment**	**Dev. (ppm)**
LV	185.1646	$C_{10}H_{21}N_2O^+$	-1.0619	213.1597	$C_{11}H_{21}N_2O_2^+$	-0.3687				168.1381	$C_{10}H_{18}NO^+$	-1.4221
LVQ	313.2234	$C_{15}H_{29}N_4O_3^+$	-0.0941	341.2183	$C_{16}H_{29}N_4O_4^+$	-0.1580	358.2449	$C_{16}H_{32}N_5O_4^+$	0.1728	296.1969	$C_{15}H_{26}N_3O_3^+$	0.0395
LVQR	469.3245	$C_{21}H_{41}N_8O_4^+$	-0.0668	497.3193	$C_{22}H_{41}N_8O_5^+$	-0.3695	514.3457	$C_{22}H_{44}N_9O_5^+$	-0.5832	452.2978	$C_{21}H_{38}N_7O_4^+$	-0.3155
LVQRN	583.3671	$C_{25}H_{47}N_{10}O_6^+$	-0.5270	611.3625	$C_{26}H_{47}N_{10}O_7^+$	0.1553				566.3410	$C_{25}H_{44}N_9O_6^+$	0.0872
LVQRNV				710.4310	$C_{31}H_{56}N_{11}O_8^+$	0.3026						
LVQRNVN	796.4784	$C_{34}H_{62}N_{13}O_9^+$	-0.4722	824.4743	$C_{35}H_{62}N_{13}O_{10}^+$	0.7728				779.4524	$C_{34}H_{59}N_{12}O_9^+$	0.1414

Supplementary Table 121 Peak list exported from SurfaceLab spectrum of L-lactate dehydrogenase, consisting of ions detected in the spectrum and assigned internal fragments of the sequence NLLKEEHVP. The *m/z* values represent the experimentally observed center mass of each peak. The deviation (dev.) represents the parts per million (ppm) accuracy of the assignment. The colour corresponds to the presence of the observed sequence in L-lactate dehydrogenase presented in Supplementary Figure 27.

	a			b			c			a-NH3		
**Description**	***m/z***	**Assignment**	**Dev. (ppm)**	***m/z***	**Assignment**	**Dev. (ppm)**	***m/z***	**Assignment**	**Dev. (ppm)**	***m/z***	**Assignment**	**Dev. (ppm)**
NL	200.1391	$C_9H_{18}N_3O_2^+$	-1.0332	228.1343	$C_{10}H_{18}N_3O_3^+$	0.1797	245.1609	$C_{10}H_{21}N_4O_3^+$	0.3624	183.1126	$C_9H_{15}N_2O_2^+$	-0.9500
NLL	313.2234	$C_{15}H_{29}N_4O_3^+$	-0.0941	341.2183	$C_{16}H_{29}N_4O_4^+$	-0.1580	358.2449	$C_{16}H_{32}N_5O_4^+$	0.1728	296.1969	$C_{15}H_{26}N_3O_3^+$	0.0395
NLLK												
NLLKE				598.3560	$C_{27}H_{48}N_7O_8^+$	0.1367				553.3342	$C_{26}H_{45}N_6O_7^+$	-0.4070
NLLKEE												
NLLKEEH				864.4564	$C_{38}H_{62}N_{11}O_{12}^+$	-1.1462				819.4365	$C_{37}H_{59}N_{10}O_{11}^+$	0.7218
NLLKEEHV	935.5342	$C_{42}H_{71}N_{12}O_{12}^+$	3.5638	963.5262	$C_{43}H_{71}N_{12}O_{13}^+$	0.3959						
NLLKEEHVP	1004.5888	$C_{46}H_{78}N_{13}O_{12}^+$	0.0123	1032.5833	$C_{47}H_{78}N_{13}O_{13}^+$	-0.3743	1049.6093	$C_{47}H_{81}N_{14}O_{13}^+$	-0.8520			

Supplementary Table 122 Peak list exported from SurfaceLab spectrum of L-lactate dehydrogenase, consisting of ions detected in the spectrum and assigned internal fragments of the sequence QRNVNIF. The *m/z* values represent the experimentally observed center mass of each peak. The deviation (dev.) represents the parts per million (ppm) accuracy of the assignment. The colour corresponds to the presence of the observed sequence in L-lactate dehydrogenase presented in Supplementary Figure 27.

	a			b			c			a-NH3		
**Description**	***m/z***	**Assignment**	**Dev. (ppm)**	***m/z***	**Assignment**	**Dev. (ppm)**	***m/z***	**Assignment**	**Dev. (ppm)**	***m/z***	**Assignment**	**Dev. (ppm)**
QR	257.1720	$C_{10}H_{21}N_6O_2^+$	-0.2625	285.1670	$C_{11}H_{21}N_6O_3^+$	-0.0369	302.1938	$C_{11}H_{24}N_7O_3^+$	0.8632	240.1455	$C_{10}H_{18}N_5O_2^+$	-0.0898
QRN	371.2150	$C_{14}H_{27}N_8O_4^+$	0.1702	399.2103	$C_{15}H_{27}N_8O_5^+$	1.0884				354.1884	$C_{14}H_{24}N_7O_4^+$	-0.0805
QRNV	470.2836	$C_{19}H_{36}N_9O_5^+$	0.4376	498.2783	$C_{20}H_{36}N_9O_6^+$	-0.0086	515.3047	$C_{20}H_{39}N_{10}O_6^+$	-0.2236	453.2568	$C_{19}H_{33}N_8O_5^+$	-0.0905
QRNVN	584.3263	$C_{23}H_{42}N_{11}O_7^+$	0.0480	612.3215	$C_{24}H_{42}N_{11}O_8^+$	0.4832				567.2998	$C_{23}H_{39}N_{10}O_7^+$	0.0986
QRNVNI	697.4116	$C_{29}H_{53}N_{12}O_8^+$	1.7624							680.3836	$C_{29}H_{50}N_{11}O_8^+$	-0.3386
QRNVNIF	0.0000			872.4746	$C_{39}H_{62}N_{13}O_{10}^+$	1.0363						

Supplementary Table 123 Peak list exported from SurfaceLab spectrum of L-lactate dehydrogenase, consisting of ions detected in the spectrum and assigned C-terminus sequence TLWGIQKELQF. The *m/z* values represent the experimentally observed center mass of each peak. The deviation (dev.) represents the parts per million (ppm) accuracy of the assignment. The colour corresponds to the presence of the observed sequence in L-lactate dehydrogenase presented in Supplementary Figure 27.

	**y**			**y+Na**			**z**			**z-1**		
**Description**	***m/z***	**Assignment**	**Dev. (ppm)**	***m/z***	**Assignment**	**Dev. (ppm)**	***m/z***	**Assignment**	**Dev. (ppm)**	***m/z***	**Assignment**	**Dev. (ppm)**
F	166.0860	$C_9H_{12}NO_2^+$	-1.3064									
QF	294.1448	$C_{14}H_{20}N_3O_4^+$	0.0225							277.1183	$C_{14}H_{17}N_2O_4^+$	0.0162
LQF	407.2290	$C_{20}H_{31}N_4O_5^+$	0.1543							390.2025	$C_{20}H_{28}N_3O_5^+$	0.5160
ELQF												
KELQF	664.3662	$C_{31}H_{50}N_7O_9^+$	-0.3473									
QKELQF	792.4247	$C_{36}H_{58}N_9O_{11}^+$	-0.4260	814.4063	$C_{36}H_{57}N_9O_{11}Na^+$	-0.7850						
IQKELQF	905.5069	$C_{42}H_{69}N_{10}O_{12}^+$	-2.4356									
GIQKELQF	962.5304	$C_{44}H_{72}N_{11}O_{13}^+$	-0.1666	984.5126	$C_{44}H_{71}N_{11}O_{13}Na^+$	0.1078						
WGIQKELQF												
LWGIQKELQF				1283.6752	$C_{61}H_{92}N_{14}O_{15}Na^+$	-0.4991						
TLWGIQKELQF				1384.7225	$C_{65}H_{99}N_{15}O_{17}Na^+$	-0.7999						

Catalase

a)

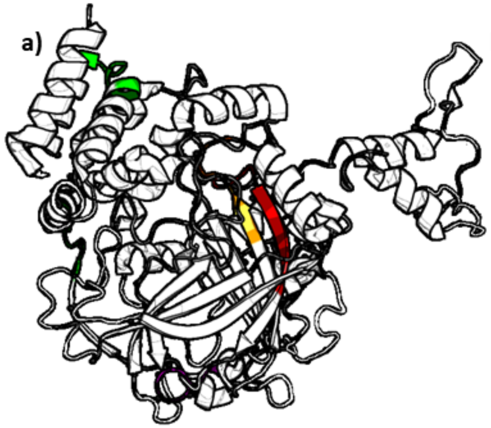

b)

Uniprot database code	Amino acid sequence
CATA_BOVIN1n	ADNRDPASDQMKHWKEQRAAQKPDVLTTGGGNPVGDKLNSLTVGPRGPLL<br>VQDVVFTDEMAHFDRERIPERVVHAKGAGAFGYFEVTHDITRYSKAKVFEHI<br>GKRTPIAVRFSTVAGESGSADTVRDPRGFAVKFYTEDGNWDLVGNNTPIFFI<br>RDALLFPSFIHSQKRNPQTHLKDPDMVWDFWSLRPESLHQVSFLFSDRGIPD<br>GHRHMNGYGSHTFKLVNANGEAVYCKFHYKTDQGIKNLSVEDAARLAHEDP<br>DYGLRDLFNAIATGNYPSWTLYIQVMTFSEAEIFPFNPFDLTKVWPHGDYPLI<br>PVGKLVLNRNPVNYFAEVEQLAFDPSNMPPGIEPSPDKMLQGRLFAYPDTH<br>RHRLGPNYLQIPVNCPYRARVANYQRDGPMCMMDNQGGAPNYYPNSFSAP<br>EHQPSALEHRTHFSGDVQRFNSANDDNVTQVRTFYLKVLNEEQRKRLCENI<br>AGHLKDAQLFIQKKAVKNFSDVHPEYGSRIQALLDKYNEEKPKNAVHTYVQH<br>GSHLSAREKANL

Supplementary Figure 28 Bovine catalase (a) cartoon exported from PDB entry 3RE8[19] and (b) amino acid sequence exported from the UniProt database. The highlighted colours correspond to assigned segments of the amino acid sequence, presented in Supplementary Tables 124-131.

Supplementary Table 124 Peak list exported from SurfaceLab spectrum of bovine catalase, consisting of ions detected in the spectrum and assigned as internal fragments of the sequence GAGAFG. The *m/z* values represent the experimentally observed center mass of each peak. The deviation (dev.) represents the parts per million (ppm) accuracy of the assignment. The colour corresponds to the presence of the observed sequence in bovine catalase presented in Supplementary Figure 28.

	a			b			b-NH3			a-NH3		
**Description**	***m/z***	**Assignment**	**Dev. (ppm)**	***m/z***	**Assignment**	**Dev. (ppm)**	***m/z***	**Assignment**	**Dev. (ppm)**	***m/z***	**Assignment**	**Dev. (ppm)**
GAG	160.1079	$C_6H_{14}N_3O_2^+$	-0.8260	188.1028	$C_7H_{14}N_3O_3^+$	-0.9424	171.0763	$C_7H_{11}N_2O_3^+$	-0.8274			
GAGA							242.1137	$C_{10}H_{16}N_3O_4^+$	0.5581	214.1186	$C_9H_{16}N_3O_3^+$	-0.0263
GAGAF	378.2137	$C_{18}H_{28}N_5O_4^+$	0.3744	406.2088	$C_{19}H_{28}N_5O_5^+$	0.6989	389.1824	$C_{19}H_{25}N_4O_5^+$	1.1273	361.1873	$C_{18}H_{25}N_4O_4^+$	0.6997
GAGAFG	435.2351	$C_{20}H_{31}N_6O_5^+$	0.1228	463.2303	$C_{21}H_{31}N_6O_6^+$	0.8061						

Supplementary Table 125 Peak list exported from SurfaceLab spectrum of bovine catalase, consisting of ions detected in the spectrum and assigned as internal fragments of the sequence GHLKDAQL. The *m/z* values represent the experimentally observed center mass of each peak. The deviation (dev.) represents the parts per million (ppm) accuracy of the assignment. The colour corresponds to the presence of the observed sequence in bovine catalase presented in Supplementary Figure 28.

	a			b			b-NH3			a-NH3		
**Description**	***m/z***	**Assignment**	**Dev. (ppm)**	***m/z***	**Assignment**	**Dev. (ppm)**	***m/z***	**Assignment**	**Dev. (ppm)**	***m/z***	**Assignment**	**Dev. (ppm)**
GH	169.1082	$C_7H_{13}N_4O^+$	-0.9471	197.1032	$C_8H_{13}N_4O_2^+$	-0.3411	180.0766	$C_8H_{10}N_3O_2^+$	-0.7330	152.0817	$C_7H_{10}N_3O^+$	-0.6977
GHL	282.1926	$C_{13}H_{24}N_5O_2^+$	0.5304	310.1875	$C_{14}H_{24}N_5O_3^+$	0.3874	293.1609	$C_{14}H_{21}N_4O_3^+$	0.2892	265.1660	$C_{13}H_{21}N_4O_2^+$	0.5363
GHLK	410.2880	$C_{19}H_{36}N_7O_3^+$	1.3051	438.2825	$C_{20}H_{36}N_7O_4^+$	0.3574	421.2559	$C_{20}H_{33}N_6O_4^+$	0.3772	393.2609	$C_{19}H_{33}N_6O_3^+$	0.1308
GHLKD	525.3145	$C_{23}H_{41}N_8O_6^+$	0.2952	553.3091	$C_{24}H_{41}N_8O_7^+$	-0.2236	536.2833	$C_{24}H_{38}N_7O_7^+$	1.0831	508.2881	$C_{23}H_{38}N_7O_6^+$	0.5235
GHLKDA	596.3519	$C_{26}H_{46}N_9O_7^+$	0.7117	624.3468	$C_{27}H_{46}N_9O_8^+$	0.7255	607.3200	$C_{27}H_{43}N_8O_8^+$	0.2794	579.3248	$C_{26}H_{43}N_8O_7^+$	-0.1541
GHLKDAQ	724.4102	$C_{31}H_{54}N_{11}O_9^+$	0.2143	752.4047	$C_{32}H_{54}N_{11}O_{10}^+$	-0.3265						
GHLKDAQL										820.4664	$C_{37}H_{62}N_{11}O_{10}^+$	-1.3819

Supplementary Table 126 Peak list exported from SurfaceLab spectrum of bovine catalase, consisting of ions detected in the spectrum and assigned as internal fragments of the sequence KNAVHTY. The *m/z* values represent the experimentally observed center mass of each peak. The deviation (dev.) represents the parts per million (ppm) accuracy of the assignment. The colour corresponds to the presence of the observed sequence in bovine catalase presented in Supplementary Figure 28.

	a			b			b-NH3			a-NH3		
**Description**	***m/z***	**Assignment**	**Dev. (ppm)**	***m/z***	**Assignment**	**Dev. (ppm)**	***m/z***	**Assignment**	**Dev. (ppm)**	***m/z***	**Assignment**	**Dev. (ppm)**
KN	217.1660	$C_9H_{21}N_4O_2^+$	0.6368				228.1345	$C_{10}H_{18}N_3O_3^+$	0.8050	200.1393	$C_9H_{18}N_3O_2^+$	-0.4637
KNA	288.2031	$C_{12}H_{26}N_5O_3^+$	0.2926				299.1715	$C_{13}H_{23}N_4O_4^+$	0.3873	271.1765	$C_{12}H_{23}N_4O_3^+$	0.2253
KNAV	370.2455	$C_{17}H_{32}N_5O_4^+$	1.5776	398.2397	$C_{18}H_{32}N_5O_5^+$	-0.2773	381.2134	$C_{18}H_{29}N_4O_5^+$	0.3233	353.2186	$C_{17}H_{29}N_4O_4^+$	0.6692
KNAVH	524.3304	$C_{23}H_{42}N_9O_5^+$	0.0655	552.3253	$C_{24}H_{42}N_9O_6^+$	0.0499	535.2990	$C_{24}H_{39}N_8O_6^+$	0.5012	507.3042	$C_{23}H_{39}N_8O_5^+$	0.8513
KNAVHT	625.3781	$C_{27}H_{49}N_{10}O_7^+$	0.1197	653.3732	$C_{28}H_{49}N_{10}O_8^+$	0.4276				608.3518	$C_{27}H_{46}N_9O_7^+$	0.5216
KNAVHTY	788.4425	$C_{36}H_{58}N_{11}O_9^+$	1.4729									

Supplementary Table 127 Peak list exported from SurfaceLab spectrum of bovine catalase, consisting of ions detected in the spectrum and assigned as internal fragments of the sequence NIAGHL. The *m/z* values represent the experimentally observed center mass of each peak. The deviation (dev.) represents the parts per million (ppm) accuracy of the assignment. The colour corresponds to the presence of the observed sequence in bovine catalase presented in Supplementary Figure 28.

	a			b			b-NH3			a-NH3		
**Description**	***m/z***	**Assignment**	**Dev. (ppm)**	***m/z***	**Assignment**	**Dev. (ppm)**	***m/z***	**Assignment**	**Dev. (ppm)**	***m/z***	**Assignment**	**Dev. (ppm)**
NI	0.0000			230.1500	$C_{10}H_{20}N_3O_3^+$	0.3264	213.1234	$C_{10}H_{17}N_2O_3^+$	0.1723	185.1284	$C_9H_{17}N_2O_2^+$	-0.4462
NIA	0.0000						284.1607	$C_{13}H_{22}N_3O_4^+$	0.6255	256.1657	$C_{12}H_{22}N_3O_3^+$	0.3834
NIAG	0.0000						341.1821	$C_{15}H_{25}N_4O_5^+$	0.5419	313.1871	$C_{14}H_{25}N_4O_4^+$	0.2431
NIAGH	467.2726	$C_{20}H_{35}N_8O_5^+$	0.2737	495.2676	$C_{21}H_{35}N_8O_6^+$	0.4527	478.2412	$C_{21}H_{32}N_7O_6^+$	0.7224	450.2461	$C_{20}H_{32}N_7O_5^+$	0.4153
NIAGHL	580.3566	$C_{26}H_{46}N_9O_6^+$	0.0880	608.3518	$C_{27}H_{46}N_9O_7^+$	0.5216				563.3303	$C_{26}H_{43}N_8O_6^+$	0.4632

Supplementary Table 128 Peak list exported from SurfaceLab spectrum of bovine catalase, consisting of ions detected in the spectrum and assigned as internal fragments of the sequence RFSTVAG. The *m/z* values represent the experimentally observed center mass of each peak. The deviation (dev.) represents the parts per million (ppm) accuracy of the assignment. The colour corresponds to the presence of the observed sequence in bovine catalase presented in Supplementary Figure 28.

	a			b			b-NH3			a-NH3		
**Description**	***m/z***	**Assignment**	**Dev. (ppm)**	***m/z***	**Assignment**	**Dev. (ppm)**	***m/z***	**Assignment**	**Dev. (ppm)**	***m/z***	**Assignment**	**Dev. (ppm)**
RF	278.1977	$C_{14}H_{24}N_5O^+$	0.5394	306.1926	$C_{15}H_{24}N_5O_2^+$	0.3348	289.1660	$C_{15}H_{21}N_4O_2^+$	0.4808	261.1712	$C_{14}H_{21}N_4O^+$	0.6555
RFS	365.2297	$C_{17}H_{29}N_6O_3^+$	0.4640	393.2246	$C_{18}H_{29}N_6O_4^+$	0.3385	376.1981	$C_{18}H_{26}N_5O_4^+$	0.4794	348.2032	$C_{17}H_{26}N_5O_3^+$	0.5539
RFST	466.2775	$C_{21}H_{36}N_7O_5^+$	0.6096	494.2724	$C_{22}H_{36}N_7O_6^+$	0.5375	477.2459	$C_{22}H_{33}N_6O_6^+$	0.5479	449.2509	$C_{21}H_{33}N_6O_5^+$	0.4668
RFSTV	565.3462	$C_{26}H_{45}N_8O_6^+$	0.9690	593.3407	$C_{27}H_{45}N_8O_7^+$	0.2940				548.3195	$C_{26}H_{42}N_7O_6^+$	0.6371
RFSTVA	636.3832	$C_{29}H_{50}N_9O_7^+$	0.7463	664.3775	$C_{30}H_{50}N_9O_8^+$	-0.3203				619.3570	$C_{29}H_{47}N_8O_7^+$	1.2636
RFSTVAG	693.4046	$C_{31}H_{53}N_{10}O_8^+$	0.5913				704.3731	$C_{32}H_{50}N_9O_9^+$	0.7318	676.3782	$C_{31}H_{50}N_9O_8^+$	0.7336

Supplementary Table 129 Peak list exported from SurfaceLab spectrum of bovine catalase, consisting of ions detected in the spectrum and assigned as internal fragments of the sequence TRYSKA. The *m/z* values represent the experimentally observed center mass of each peak. The deviation (dev.) represents the parts per million (ppm) accuracy of the assignment. The colour corresponds to the presence of the observed sequence in bovine catalase presented in Supplementary Figure 28.

	a			b			b-NH3			a-NH3		
**Description**	***m/z***	**Assignment**	**Dev. (ppm)**	***m/z***	**Assignment**	**Dev. (ppm)**	***m/z***	**Assignment**	**Dev. (ppm)**	***m/z***	**Assignment**	**Dev. (ppm)**
RY	294.1927	$C_{14}H_{24}N_5O_2^+$	0.7023	322.1876	$C_{15}H_{24}N_5O_3^+$	0.6393	305.1610	$C_{15}H_{21}N_4O_3^+$	0.6398	277.1661	$C_{14}H_{21}N_4O_2^+$	0.6160
RYS	381.2246	$C_{17}H_{29}N_6O_4^+$	0.2697	409.2197	$C_{18}H_{29}N_6O_5^+$	0.6239	392.1930	$C_{18}H_{26}N_5O_5^+$	0.4116	364.1981	$C_{17}H_{26}N_5O_4^+$	0.5366
RYSK	509.3196	$C_{23}H_{41}N_8O_5^+$	0.3388	537.3147	$C_{24}H_{41}N_8O_6^+$	0.5509	520.2880	$C_{24}H_{38}N_7O_6^+$	0.3765	492.2932	$C_{23}H_{38}N_7O_5^+$	0.5672
TRYSKA	681.4048	$C_{30}H_{53}N_{10}O_8^+$	0.8355	709.3990	$C_{31}H_{53}N_{10}O_9^+$	-0.2617	692.3736	$C_{31}H_{50}N_9O_9^+$	1.3748	664.3775	$C_{30}H_{50}N_9O_8^+$	-0.3203

Supplementary Table 130 Peak list exported from SurfaceLab spectrum of bovine catalase, consisting of ions detected in the spectrum and assigned as internal fragments of the sequence VHAKGAGA. The *m/z* values represent the experimentally observed center mass of each peak. The deviation (dev.) represents the parts per million (ppm) accuracy of the assignment. The colour corresponds to the presence of the observed sequence in bovine catalase presented in Supplementary Figure 28.

	a			b			b-NH3			a-NH3		
**Description**	***m/z***	**Assignment**	**Dev. (ppm)**	***m/z***	**Assignment**	**Dev. (ppm)**	***m/z***	**Assignment**	**Dev. (ppm)**	***m/z***	**Assignment**	**Dev. (ppm)**
VH	211.1554	$C_{10}H_{19}N_4O^+$	0.1988	239.1504	$C_{11}H_{19}N_4O_2^+$	0.4574	222.1239	$C_{11}H_{16}N_3O_2^+$	0.6709	194.1287	$C_{10}H_{16}N_3O^+$	-0.2096
VHA	282.1926	$C_{13}H_{24}N_5O_2^+$	0.5304	310.1875	$C_{14}H_{24}N_5O_3^+$	0.3874	293.1609	$C_{14}H_{21}N_4O_3^+$	0.2892	265.1660	$C_{13}H_{21}N_4O_2^+$	0.5363
VHAK	410.2880	$C_{19}H_{36}N_7O_3^+$	1.3051	438.2825	$C_{20}H_{36}N_7O_4^+$	0.3574	421.2559	$C_{20}H_{33}N_6O_4^+$	0.3772	393.2609	$C_{19}H_{33}N_6O_3^+$	0.1308
VHAKG	467.3092	$C_{21}H_{39}N_8O_4^+$	0.6131	495.3040	$C_{22}H_{39}N_8O_5^+$	0.3295	478.2773	$C_{22}H_{36}N_7O_5^+$	0.0659	450.2824	$C_{21}H_{36}N_7O_4^+$	0.2611
VHAKGA	538.3467	$C_{24}H_{44}N_9O_5^+$	1.2352	566.3411	$C_{25}H_{44}N_9O_6^+$	0.4192	549.3147	$C_{25}H_{41}N_8O_6^+$	0.5317	521.3198	$C_{24}H_{41}N_8O_5^+$	0.6678
VHAKGAG	595.3670	$C_{26}H_{47}N_{10}O_6^+$	-0.7999	623.3626	$C_{27}H_{47}N_{10}O_7^+$	0.3772	606.3363	$C_{27}H_{44}N_9O_7^+$	0.7522	578.3410	$C_{26}H_{44}N_9O_6^+$	0.1457
VHAKGAGA	666.4047	$C_{29}H_{52}N_{11}O_7^+$	0.2202	694.3997	$C_{30}H_{52}N_{11}O_8^+$	0.2865	677.3728	$C_{30}H_{49}N_{10}O_8^+$	-0.1871	649.3783	$C_{29}H_{49}N_{10}O_7^+$	0.4417

Supplementary Table 131 Peak list exported from SurfaceLab spectrum of bovine catalase, consisting of ions detected in the spectrum and assigned as internal fragments of the sequence VQRFNSA. The *m/z* values represent the experimentally observed center mass of each peak. The deviation (dev.) represents the parts per million (ppm) accuracy of the assignment. The colour corresponds to the presence of the observed sequence in bovine catalase presented in Supplementary Figure 28.

	a			b			b-NH3			a-NH3		
**Description**	***m/z***	**Assignment**	**Dev. (ppm)**	***m/z***	**Assignment**	**Dev. (ppm)**	***m/z***	**Assignment**	**Dev. (ppm)**	***m/z***	**Assignment**	**Dev. (ppm)**
VQ	0.0000			230.1500	$C_{10}H_{20}N_3O_3^+$	0.3264	213.1234	$C_{10}H_{17}N_2O_3^+$	0.1723	185.1284	$C_9H_{17}N_2O_2^+$	-0.4462
VQR	358.2563	$C_{15}H_{32}N_7O_3^+$	0.5550	386.2511	$C_{16}H_{32}N_7O_4^+$	0.2139	369.2246	$C_{16}H_{29}N_6O_4^+$	0.2729	341.2297	$C_{15}H_{29}N_6O_3^+$	0.5163
VQRF	0.0000			533.3199	$C_{25}H_{41}N_8O_5^+$	0.8178	516.2933	$C_{25}H_{38}N_7O_5^+$	0.8063	488.2981	$C_{24}H_{38}N_7O_4^+$	0.2903
VQRFN	0.0000			647.3628	$C_{29}H_{47}N_{10}O_7^+$	0.6925	630.3361	$C_{29}H_{44}N_9O_7^+$	0.4993	602.3416	$C_{28}H_{44}N_9O_6^+$	1.1205
VQRFNS	0.0000			734.3936	$C_{32}H_{52}N_{11}O_9^+$	-1.1274	717.3676	$C_{32}H_{49}N_{10}O_9^+$	-0.3269	689.3734	$C_{31}H_{49}N_{10}O_8^+$	0.7183
VQRFNSA	777.4371	$C_{34}H_{57}N_{12}O_9^+$	0.6614									

Fibronectin

Bovine fibronectin (UniProt ID FINC_BOVIN1n) amino acid sequence exported from the UniProt database. The highlighted colours correspond to assigned segments of the amino acid sequence, presented in Supplementary Tables 132-147.

QA**QQIVQPQSPL**TVSQSKPGCYDNGKHYQINQQWERTYLGSALVCTCYGGSRGFNCESKPEPEETCFDKYTGNTYRVGDTYE**RPKDS**MIWDCTCI
GAGRGRISCTIANRCHEGGQSYKIGDTWR**RPHETG**GYMLECVCLGNGKGEWTCKPIAEKCFDQAAGTSYVVG**ETWEKPY**QGWMMVDCTCLGE
GSGRITCTSRNRCNDQDTRTSYR**IGDT**WS**KKDNRGNLL**QCICTGNGRGEWKCERHTSLQTTSAGSGS**FTDVRTAIYQ**PQPHPQPPPYGHCVTDSG
VVYSVGMQWLKTQGNKQMLCTCLGNGVSCQETAVTQTYGGNSNGEPCVLPFTYNGKTFYSCTTEGRQDGHLWCSTTSNYEQDQKYSFCTDHTV
LVQTRGGNSNGALCHFPFLYNNHNYTDCTSEGRRDNMKWCGTTQNYDADQKFGFCPMAAHEEICTTNEGVMYRIGDQWDKQHDMGHMMRCT
CVGNGRGEWTCVAYSQLRDQCIVDGITYNVNDTFHKRHEEGHMLNCTCFGQGRGRWKCDPVDQCQDSETRTFYQIGDSWEKYLQGVRYQCY**CY**
GRGIGEWACQPLQTYPDTSGPVQVIITETPSQPNSHPIQWSAPESSHISKYILRWKPKNSPDRWKEATIPGHLNSYTIKGLRPGVVYEGQLISVQHYG
QREVTR**FDFTT**TSTSPAVTSNTVTGETTPLSPVVATSESVTEITASSFVVSWVSASDTVSGFRVEYELSEEGDEPQYLDLPSTATSVNIPDLLPGRKY**T**
VNVYEISEEGEQNLILSTSQTTAPDAPPDPTVDQVDDTSIVVRWSRPRAPITGYRIVYSPSVEGSSTELNLPETANSVTLSDLQPGVQYNITIYAVEEN
QESTPVFIQQETTGVPRSDKVPP**PRDLQFV**EVTDVKITIMWTPPESPVTGYRVDVIPVNLPGEHGQRLPVSRNTFAEVTGLSPGVTYHFKVFAVNQG
RESKPLTAQQATKLDAPTNLQFINETDTTVIVTWTPPRARIVGYRLTVGLTRGGQPKQYNVGPAASQYPLRNLQPGSEYAVSLVAVKGNQQSPRVT
GVFTTLQPLGSIPHYNTEVTETTIVITWTPAPRIGFKLGVRPSQGGEAPREVTSESGSIVVSGLTPGVEYVYTISVLRDGQERDAPIVKKVVTPLSPPTN
LHLEANPDTGVLTVSWERSTTPDITGYRITTTPTNGQQGYSLEEVVHADQSSCTFENLSPGLEYNVSVYTVKDDKESVPISDTIIPEVPQLTDLSFVDIT
DSSIGLRWTPLNSSTIIGYRITVVAAGEGIPIFEDFVDSSVGYYTVTGLEPGIDYDISVITLINGGESAPTTLTQQTA**VPPPTD**LRFTNVGPDTMRVTWA
PPSSIELTNLLVRYSPVKNEEDVAELSISPSDNAVVLTNLLPGTEYLVSVSSVYEQHESIPLRGRQKTALDSPSGIDFSDITANSFTVHWIAPRATITGYR
IRHHPENMGGRPREDRVPPSRNSITLTNLNPGTEYVVSIVALNSKEESLPLVGQQSTVSDVPRDLEVIAATPTSLLISWDAPAVTVRYYRITYGETGGS
SPVQEFTVPGSKSTATISGLKPGVDYTITVYAVT**GRGD**SPASSKPVSINYRTEIDKPSQMQVTDVQDNSISVRWLPSSSPVTGYRVTTAPKNGPGPSK
TKTVGPDQTEMTIEGLQPTVEYVVSVYAQNQNGESQPLVQTAVTNIDRPKGLAFTDVDVDSIKIAWESPQGQVSRYRVTYSSPEDGIHELFPAPDGE
EETAELQGLRPGSEYTVSVVALHDDMESQPLIGTQSTTIPAPTNLKFTQVTPTSLTAQWTAPNVQLTGYRVRVTPKEKTGPMKEINLAPDSSSVVVS
GLMVATKYEVSVYALKDTLTSRPAQGVVTTLENVSPPRRARVTDATETTITISWRTKTETITGFQVDAIPANGQTPIQRTIRPDVRSYTITGLQPGTDY
KIHLYTLNDNARSSPVVIDASTAIDAPSNLRFLATTPNSLLVSWQPPRARITGYIIKYEKPGSPPREVVPRPRPGVTEATITGLEPGTEYTIQVIALKNNQ
KSEPLIGRKKTDELPQLVTLPHPNLHGPEILDVPSTVQKTPFITNPGYDTGNGIQLPGTSGQQPSLGQQMIFEEHGFRRTTPPTTATPVRHRPRPYPPNV
NEEIQIGHVP**RGDVDHH**LYPHVVGLNPNASTGQEALSQTTISWTPFQESSEYIISCHPVGIDEEPLQFRVPGTSASATLTGLTRGATYNIIVEAVKDQQ
RQKVREEVVTVGNSVDQGLSQPTDDSCFDPYTVSHYAIGEEWERLSDSGFKLSCQCLGFGSGHFRCDSSKWCHDNGVNYKIGEKWDRQGENGQM
MSCTCLGNGKGEFKCDPHEATCYDDGKT**YHVGEQ**WQKEYLGAICSCTCFGGQRGWRCDNCRRPGAEPGNEGSTAHSYNQYSQRYHQRTNTNV
NCPIECFMPLDVQADREDSRE

FINC_BOVIN2n, 75, 9913

PIQWSAPESSHISKYILRWKPKNSPDRWKEATIPGHLNSYTIKGLRPGVVYEGQLISVQHYGQREVTR**FDFTT**TS

Supplementary Table 132 Peak list exported from SurfaceLab spectrum of bovine fibronectin, consisting of ions detected in the spectrum and assigned as fragments of N-terminal sequence QAQQIVQPQ. The *m/z* values represent the experimentally observed center mass of each peak. The deviation (dev.) represents the parts per million (ppm) accuracy of the assignment.

	a			b			c			a-NH3		
**Description**	***m/z***	**Assignment**	**Dev. (ppm)**	***m/z***	**Assignment**	**Dev. (ppm)**	***m/z***	**Assignment**	**Dev. (ppm)**	***m/z***	**Assignment**	**Dev. (ppm)**
QA	172.1079	$C_7H_{14}N_3O_2^+$	-1.0623	200.1029	$C_8H_{14}N_3O_3^+$	-0.5018				155.0814	$C_7H_{11}N_2O_2^+$	-0.8489
QAQ	300.1668	$C_{12}H_{22}N_5O_4^+$	0.5516	328.1617	$C_{13}H_{22}N_5O_5^+$	0.5767				283.1402	$C_{12}H_{19}N_4O_4^+$	0.4901
QAQQ	428.2258	$C_{17}H_{30}N_7O_6^+$	1.4904	456.2210	$C_{18}H_{30}N_7O_7^+$	1.9349				411.1988	$C_{17}H_{27}N_6O_6^+$	0.4248
QAQQI	541.3098	$C_{23}H_{41}N_8O_7^+$	1.0184	569.3047	$C_{24}H_{41}N_8O_8^+$	0.9268				524.2830	$C_{23}H_{38}N_7O_7^+$	0.5065
QAQQIV	640.3789	$C_{28}H_{50}N_9O_8^+$	1.9152	668.3730	$C_{29}H_{50}N_9O_9^+$	0.5346				623.3514	$C_{28}H_{47}N_8O_8^+$	0.3789
QAQQIVQ										751.4098	$C_{33}H_{55}N_{10}O_{10}^+$	0.0678
QAQQIVQP										848.4625	$C_{38}H_{62}N_{11}O_{11}^+$	0.0165
QAQQIVQPQ										976.5208	$C_{43}H_{70}N_{13}O_{13}^+$	-0.2809

Supplementary Table 133 Peak list exported from SurfaceLab spectrum of bovine fibronectin, consisting of ions detected in the spectrum and assigned as fragments of N-terminal sequence QAQQIVQPQSPL. The *m/z* values represent the experimentally observed center mass of each peak. The deviation (dev.) represents the parts per million (ppm) accuracy of the assignment.

	**a**			**b**			**c**			**a-NH3**		
**Description**	***m/z***	**Assignment**	**Dev. (ppm)**	***m/z***	**Assignment**	**Dev. (ppm)**	***m/z***	**Assignment**	**Dev. (ppm)**	***m/z***	**Assignment**	**Dev. (ppm)**
QA	194.0900	$C_7H_{13}N_3O_2Na^+$	-0.0932	222.0851	$C_8H_{13}N_3O_3Na^+$	0.6566	239.1117	$C_8H_{16}N_4O_3Na^+$	0.9614	177.0634	$C_7H_{10}N_2O_2Na^+$	-0.3176
QAQ	322.1487	$C_{12}H_{21}N_5O_4Na^+$	0.4185	350.1436	$C_{13}H_{21}N_5O_5Na^+$	0.3996	367.1702	$C_{13}H_{24}N_6O_5Na^+$	0.3398	305.1221	$C_{12}H_{18}N_4O_4Na^+$	0.4028
QAQQ	450.2078	$C_{17}H_{29}N_7O_6Na^+$	1.3695	478.2026	$C_{18}H_{29}N_7O_7Na^+$	1.1076	495.2293	$C_{18}H_{32}N_8O_7Na^+$	1.3135	433.1809	$C_{17}H_{26}N_6O_6Na^+$	0.6021
QAQQI	563.2930	$C_{23}H_{40}N_8O_7Na^+$	3.1208	591.2875	$C_{24}H_{40}N_8O_8Na^+$	2.3322	619.2828	$C_{25}H_{40}N_8O_9Na^+$	2.7650	546.2650	$C_{23}H_{37}N_7O_7Na^+$	0.6563
QAQQIV	662.3612	$C_{28}H_{49}N_9O_8Na^+$	2.3896	690.3555	$C_{29}H_{49}N_9O_9Na^+$	1.4288	707.3838	$C_{29}H_{52}N_{10}O_9Na^+$	3.8833	645.3339	$C_{28}H_{46}N_8O_8Na^+$	1.2023
QAQQIVQ	790.4206	$C_{33}H_{57}N_{11}O_{10}Na^+$	3.0762	818.4159	$C_{34}H_{57}N_{11}O_{11}Na^+$	3.3673	835.4421	$C_{34}H_{60}N_{12}O_{11}Na^+$	2.9375	773.3920	$C_{33}H_{54}N_{10}O_{10}Na^+$	0.3992
QAQQIVQP	887.4729	$C_{38}H_{64}N_{12}O_{11}Na^+$	2.1432	915.4667	$C_{39}H_{64}N_{12}O_{12}Na^+$	0.9422				870.4450	$C_{38}H_{61}N_{11}O_{11}Na^+$	0.6373
QAQQIVQPQ				1043.5244	$C_{44}H_{72}N_{14}O_{14}Na^+$	-0.0826				998.5021	$C_{43}H_{69}N_{13}O_{13}Na^+$	-0.9070
QAQQIVQPQS										1085.5377	$C_{46}H_{74}N_{14}O_{15}Na^+$	2.4377
QAQQIVQPQSP				1227.6079	$C_{52}H_{84}N_{16}O_{17}Na^+$	-1.1027						
QAQQIVQPQSPL				1340.6953	$C_{58}H_{95}N_{17}O_{18}Na^+$	1.4674						

Supplementary Table 134 Peak list exported from SurfaceLab spectrum of bovine fibronectin, consisting of ions detected in the spectrum and assigned as C-terminal fragments of the sequence FDFTTTS. The *m/z* values represent the experimentally observed center mass of each peak. The deviation (dev.) represents the parts per million (ppm) accuracy of the assignment.

	y			z			z-1			z+1		
**Description**	***m/z***	**Assignment**	**Dev. (ppm)**	***m/z***	**Assignment**	**Dev. (ppm)**	***m/z***	**Assignment**	**Dev. (ppm)**	***m/z***	**Assignment**	**Dev. (ppm)**
TS	219.1341	$C_9H_{19}N_2O_4^+$	0.5686									
TTS												
TTTS	488.3195	$C_{21}H_{42}N_7O_6^+$	0.7539									
FTTTS	651.3818	$C_{30}H_{51}N_8O_8^+$	-1.0140	635.3627	$C_{30}H_{49}N_7O_8^+$	-1.6622	634.3545	$C_{30}H_{48}N_7O_8^+$	-2.2230	636.3710	$C_{30}H_{50}N_7O_8^+$	-0.8364
DFTTTS	708.4049	$C_{32}H_{54}N_9O_9^+$	1.3447	692.3833	$C_{32}H_{52}N_8O_9^+$	-2.6730	691.3760	$C_{32}H_{51}N_8O_{9+}$	-1.9233	693.3918	$C_{32}H_{53}N_8O_9^+$	-1.6799
FDFTTTS	807.4733	$C_{37}H_{63}N_{10}O_{10}^+$	1.2288	791.4527	$C_{37}H_{61}N_9O_{10}^+$	-1.1244	790.4442	$C_{37}H_{60}N_9O_{10}^+$	-1.9629			

Supplementary Table 135 Peak list exported from SurfaceLab spectrum of bovine fibronectin, consisting of ions detected in the spectrum and assigned as internal fragments of the sequence GSFTDVRTAIYQ. The *m/z* values represent the experimentally observed center mass of each peak. The deviation (dev.) represents the parts per million (ppm) accuracy of the assignment.

	a			b			c			a-NH3		
**Description**	***m/z***	**Assignment**	**Dev. (ppm)**	***m/z***	**Assignment**	**Dev. (ppm)**	***m/z***	**Assignment**	**Dev. (ppm)**	***m/z***	**Assignment**	**Dev. (ppm)**
GS				169.0582	$C_5H_{10}N_2O_3Na^+$	-0.7074						
GSF	288.1320	$C_{13}H_{19}N_3O_3Na^+$	0.5759	316.1269	$C_{14}H_{19}N_3O_4Na^+$	0.2509	333.1534	$C_{14}H_{22}N_4O_4Na^+$	0.2319			
GSFT	389.1792	$C_{17}H_{26}N_4O_5Na^+$	-0.7506	417.1759	$C_{18}H_{26}N_4O_6Na^+$	3.4635	434.2015	$C_{18}H_{29}N_5O_6Na^+$	1.2474	372.1524	$C_{17}H_{23}N_3O_5Na^+$	-1.5239
GSFTD	504.2078	$C_{21}H_{31}N_5O_8Na^+$	2.5568	532.2017	$C_{22}H_{31}N_5O_9Na^+$	0.5947	549.2290	$C_{22}H_{34}N_6O_9Na^+$	1.9229			
GSFTDV	619.2705	$C_{26}H_{40}N_6O_{10}Na^+$	1.1603	647.2654	$C_{27}H_{40}N_6O_{11}Na^+$	1.0988	664.2920	$C_{27}H_{43}N_7O_{11}Na^+$	1.1053	602.2425	$C_{26}H_{37}N_5O_{10}Na^+$	-1.2131
GSFTDVR	759.3781	$C_{32}H_{52}N_{10}O_{10}Na^+$	2.7203	787.3717	$C_{33}H_{52}N_{10}O_{11}Na^+$	0.9374	804.4004	$C_{33}H_{55}N_{11}O_{11}Na^+$	3.6314	742.3509	$C_{32}H_{49}N_9O_{10}Na^+$	1.9195
GSFTDVRT	860.4264	$C_{36}H_{59}N_{11}O_{12}Na^+$	3.2080	888.4209	$C_{37}H_{59}N_{11}O_{13}Na^+$	2.5443	905.4497	$C_{37}H_{62}N_{12}O_{13}Na^+$	5.0573	843.3983	$C_{36}H_{56}N_{10}O_{12}Na^+$	1.4061
GSFTDVRTA	931.4646	$C_{39}H_{64}N_{12}O_{13}Na^+$	4.0627	959.4592	$C_{40}H_{64}N_{12}O_{14}Na^+$	3.6252				914.4350	$C_{39}H_{61}N_{11}O_{13}Na^+$	0.8698
GSFTDVRTAI	1044.5467	$C_{45}H_{75}N_{13}O_{14}Na^+$	1.7128							1027.5182	$C_{45}H_{72}N_{12}O_{14}Na^+$	-0.0944
GSFTDVRTAIY	1207.6162	$C_{54}H_{84}N_{14}O_{16}Na^+$	6.6210									
GSFTDVRTAIYQ	1335.6719	$C_{59}H_{92}N_{16}O_{18}Na^+$	3.8144	1363.6613	$C_{60}H_{92}N_{16}O_{19}Na^+$	-0.3196						

Supplementary Table 136 Peak list exported from SurfaceLab spectrum of bovine fibronectin, consisting of ions detected in the spectrum and assigned as internal fragments of the sequence KTYHVGEQ. The *m/z* values represent the experimentally observed center mass of each peak. The deviation (dev.) represents the parts per million (ppm) accuracy of the assignment.

	a			b			c		
**Description**	***m/z***	**Assignment**	**Dev. (ppm)**	***m/z***	**Assignment**	**Dev. (ppm)**	***m/z***	**Assignment**	**Dev. (ppm)**
KT				232.1657	$C_{10}H_{22}N_3O_3^+$	0.6359			
KTY	367.2343	$C_{18}H_{31}N_4O_4^+$	0.7808	395.2288	$C_{19}H_{31}N_4O_5^+$	-0.1852	412.2556	$C_{19}H_{34}N_5O_5^+$	0.2809
KTYH	504.2927	$C_{24}H_{38}N_7O_5^+$	-0.4300	532.2869	$C_{25}H_{38}N_7O_6^+$	-1.7070	549.3146	$C_{25}H_{41}N_8O_6^+$	0.3977
KTYHV	603.3605	$C_{29}H_{47}N_8O_6^+$	-1.4121	631.3550	$C_{30}H_{47}N_8O_7^+$	-2.0031	648.3826	$C_{30}H_{50}N_9O_7^+$	-0.2804
KTYHVG	660.3830	$C_{31}H_{50}N_9O_7^+$	0.3816	688.3773	$C_{32}H_{50}N_9O_8^+$	-0.5289	705.4044	$C_{32}H_{53}N_{10}O_8^+$	0.1890
KTYHVGE	789.4250	$C_{36}H_{57}N_{10}O_{10}^+$	-0.4963	817.4197	$C_{37}H_{57}N_{10}O_{11}^+$	-0.6747	834.4471	$C_{37}H_{60}N_{11}O_{11}^+$	0.3092
KTYHVGEQ	917.4828	$C_{41}H_{65}N_{12}O_{12}^+$	-1.2159	945.4809	$C_{42}H_{65}N_{12}O_{13}^+$	2.1199	962.5061	$C_{42}H_{68}N_{13}O_{13}^+$	0.7545

Supplementary Table 137 Peak list exported from SurfaceLab spectrum of bovine fibronectin, consisting of ions detected in the spectrum and assigned as internal fragments of the sequence KYTVNVYE. The *m/z* values represent the experimentally observed center mass of each peak. The deviation (dev.) represents the parts per million (ppm) accuracy of the assignment.

	a			b			c			a-NH3		
**Description**	***m/z***	**Assignment**	**Dev. (ppm)**	***m/z***	**Assignment**	**Dev. (ppm)**	***m/z***	**Assignment**	**Dev. (ppm)**	***m/z***	**Assignment**	**Dev. (ppm)**
KY	288.1667	$C_{14}H_{23}N_3O_2Na^+$	-5.2155	316.1619	$C_{15}H_{23}N_3O_3Na^+$	-4.0174	333.1895	$C_{15}H_{26}N_4O_3Na^+$	-0.5242			
KYT	367.2343	$C_{18}H_{31}N_4O_4^+$	0.7808	395.2288	$C_{19}H_{31}N_4O_5^+$	-0.1852	412.2556	$C_{19}H_{34}N_5O_5^+$	0.2809			
KYTV	488.2833	$C_{23}H_{39}N_5O_5Na^+$	-2.2092	516.2789	$C_{24}H_{39}N_5O_6Na^+$	-0.6679	533.3062	$C_{24}H_{42}N_6O_6Na^+$	0.8173			
KYTVN	602.3271	$C_{27}H_{45}N_7O_7Na^+$	-0.3131	630.3226	$C_{28}H_{45}N_7O_8Na^+$	0.6006	647.3502	$C_{28}H_{48}N_8O_8Na^+$	2.2794			
KYTVNV	701.3946	$C_{32}H_{54}N_8O_8Na^+$	-1.5448	729.3915	$C_{33}H_{54}N_8O_9Na^+$	1.1837	746.4183	$C_{33}H_{57}N_9O_9Na^+$	1.5948			
KYTVNVY	864.4579	$C_{41}H_{63}N_9O_{10}Na^+$	-1.2946	892.4530	$C_{42}H_{63}N_9O_{11}Na^+$	-1.0323	909.4803	$C_{42}H_{66}N_{10}O_{11}Na^+$	-0.1485	847.4302	$C_{41}H_{60}N_8O_{10}Na^+$	-2.6568
KYTVNVYE	993.4987	$C_{46}H_{70}N_{10}O_{13}Na^+$	-2.9491	1021.4935	$C_{47}H_{70}N_{10}O_{14}Na^+$	-2.9101						

Supplementary Table 138 Peak list exported from SurfaceLab spectrum of bovine fibronectin, consisting of ions detected in the spectrum and assigned as internal fragments of the sequence VTGRGD. The *m/z* values represent the experimentally observed center mass of each peak. The deviation (dev.) represents the parts per million (ppm) accuracy of the assignment.

	a			b			b-NH3			a-NH3		
**Description**	***m/z***	**Assignment**	**Dev. (ppm)**	***m/z***	**Assignment**	**Dev. (ppm)**	***m/z***	**Assignment**	**Dev. (ppm)**	***m/z***	**Assignment**	**Dev. (ppm)**
VT	175.1441	$C_8H_{19}N_2O_2^+$	-0.1526	203.1390	$C_9H_{19}N_2O_3^+$	0.08804	186.1124	$C_9H_{16}NO_3^+$	-0.4751	158.1175	$C_8H_{16}NO_2^+$	-0.6008
VTG	232.1657	$C_{10}H_{22}N_3O_3^+$	0.6359	260.1606	$C_{11}H_{22}N_3O_4^+$	0.4910	243.1341	$C_{11}H_{19}N_2O_4^+$	0.7233	215.1392	$C_{10}H_{19}N_2O_3^+$	0.6761
VTGR	388.2669	$C_{16}H_{34}N_7O_4^+$	0.6788	416.2618	$C_{17}H_{34}N_7O_5^+$	0.5290	399.2354	$C_{17}H_{31}N_6O_5^+$	0.8475	371.2403	$C_{16}H_{31}N_6O_4^+$	0.3910
VTGRG	445.2885	$C_{18}H_{37}N_8O_5^+$	0.8932	473.2834	$C_{19}H_{37}N_8O_6^+$	0.7790	456.2568	$C_{19}H_{34}N_7O_6^+$	0.5296	428.2619	$C_{18}H_{34}N_7O_5^+$	0.7606
VTGRGD	560.3171	$C_{22}H_{42}N_9O_8^+$	3.5404	588.3118	$C_{23}H_{42}N_9O_9^+$	3.0299	571.2850	$C_{23}H_{39}N_8O_9^+$	2.6536	543.2897	$C_{22}H_{39}N_8O_8^+$	2.1954

Supplementary Table 139 Peak list exported from SurfaceLab spectrum of bovine fibronectin, consisting of ions detected in the spectrum and assigned as internal fragments of the sequence VPPPTDLR. The *m/z* values represent the experimentally observed center mass of each peak. The deviation (dev.) represents the parts per million (ppm) accuracy of the assignment.

	a			b			c			a-NH3		
**Description**	***m/z***	**Assignment**	**Dev. (ppm)**	***m/z***	**Assignment**	**Dev. (ppm)**	***m/z***	**Assignment**	**Dev. (ppm)**	***m/z***	**Assignment**	**Dev. (ppm)**
LR	244.2134	$C_{11}H_{26}N_5O^+$	0.7368	272.2082	$C_{12}H_{26}N_5O_2^+$	0.4682				227.1867	$C_{11}H_{23}N_4O_+$	0.3615
DLR	359.2403	$C_{15}H_{31}N_6O_4^+$	0.4328	387.2353	$C_{16}H_{31}N_6O_5^+$	0.6710				342.2137	$C_{15}H_{28}N_5O_4^+$	0.3798
TDLR	460.2883	$C_{19}H_{38}N_7O_6^+$	1.1301	488.2833	$C_{20}H_{38}N_7O_7^+$	1.1034						
PTDLR				585.3361	$C_{25}H_{45}N_8O_8^+$	1.0269						
PPTDLR	654.3937	$C_{29}H_{52}N_9O_8^+$	0.5825	682.3886	$C_{30}H_{52}N_9O_9^+$	0.4587						
PPPTDLR				779.4412	$C_{35}H_{59}N_{10}O_{10}^+$	0.2898						
VPPPTDLR				878.5095	$C_{40}H_{68}N_{11}O_{11}^+$	0.0626						

Supplementary Table 140 Peak list exported from SurfaceLab spectrum of bovine fibronectin, consisting of ions detected in the spectrum and assigned as internal fragments of the sequence VVGETWEKPY. The *m/z* values represent the experimentally observed center mass of each peak. The deviation (dev.) represents the parts per million (ppm) accuracy of the assignment.

	**a**			**b**			**c**			**a-NH3**		
**Description**	***m/z***	**Assignment**	**Dev. (ppm)**	***m/z***	**Assignment**	**Dev. (ppm)**	***m/z***	**Assignment**	**Dev. (ppm)**	***m/z***	**Assignment**	**Dev. (ppm)**
VVG	252.1684	$C_{11}H_{23}N_3O_2Na^+$	0.7406	280.1633	$C_{12}H_{23}N_3O_3Na^+$	0.4327				235.1418	$C_{11}H_{20}N_2O_2Na^+$	0.6362
VVGE				409.2068	$C_{17}H_{30}N_4O_6Na^+$	2.5921						
VVGET	482.2604	$C_{20}H_{37}N_5O_7Na^+$	3.9113	510.2546	$C_{21}H_{37}N_5O_8Na^+$	2.3157						
VVGETW	668.3372	$C_{31}H_{47}N_7O_8Na^+$	-0.9348	696.3317	$C_{32}H_{47}N_7O_9Na^+$	-1.5125	713.3593	$C_{32}H_{50}N_8O_9Na^+$	0.0201			
VVGETWE	797.3800	$C_{36}H_{54}N_8O_{11}Na^+$	-0.4764	825.3729	$C_{37}H_{54}N_8O_{12}Na^+$	-2.9273				780.3521	$C_{36}H_{51}N_7O_{11}Na^+$	-2.3121
VVGETWEK	925.4759	$C_{42}H_{66}N_{10}O_{12}Na^+$	0.6025	953.4703	$C_{43}H_{66}N_{10}O_{13}Na^+$	0.0043				908.4472	$C_{42}H_{63}N_9O_{12}Na^+$	-1.7527
VVGETWEKP	1022.5292	$C_{47}H_{73}N_{11}O_{13}Na^+$	0.9975	1050.5249	$C_{48}H_{73}N_{11}O_{14}Na^+$	1.7257				1005.5009	$C_{47}H_{70}N_{10}O_{13}Na^+$	-0.7409
VVGETWEKPY				1213.5896	$C_{57}H_{82}N_{12}O_{16}Na^+$	2.6269						

Supplementary Table 141 Peak list exported from SurfaceLab spectrum of bovine fibronectin, consisting of ions detected in the spectrum and assigned as internal fragments of the sequence WRRPHETG. The *m/z* values represent the experimentally observed center mass of each peak. The deviation (dev.) represents the parts per million (ppm) accuracy of the assignment.

	a			b			c			a-NH3		
**Description**	***m/z***	**Assignment**	**Dev. (ppm)**	***m/z***	**Assignment**	**Dev. (ppm)**	***m/z***	**Assignment**	**Dev. (ppm)**	***m/z***	**Assignment**	**Dev. (ppm)**
WR				345.2034	$C_{17}H_{25}N_6O_2^+$	0.1805						
WRR	495.2921	$C_{22}H_{36}N_{10}O_2Na^+$	1.2619	523.2868	$C_{23}H_{36}N_{10}O_3Na^+$	0.8010						
WRRP	592.3448	$C_{27}H_{43}N_{11}O_3Na^+$	0.9250	620.3387	$C_{28}H_{43}N_{11}O_4Na^+$	-0.7931						
WRRPH	729.4034	$C_{33}H_{50}N_{14}O_4Na^+$	0.3577	757.3976	$C_{34}H_{50}N_{14}O_5Na^+$	-0.6691						
WRRPHE	858.4455	$C_{38}H_{57}N_{15}O_7Na^+$	-0.3065	886.4407	$C_{39}H_{57}N_{15}O_8Na^+$	0.0240						
WRRPHET				987.4893	$C_{43}H_{64}N_{16}O_{10}Na^+$	0.9593						
WRRPHETG				1044.5088	$C_{45}H_{67}N_{17}O_{11}Na^+$	-0.9468						

Supplementary Table 142 Peak list exported from SurfaceLab spectrum of bovine fibronectin, consisting of ions detected in the spectrum and assigned as internal fragments of the sequence GKTYHVGEQ. The *m/z* values represent the experimentally observed center mass of each peak. The deviation (dev.) represents the parts per million (ppm) accuracy of the assignment.

	a			b			c			a-NH3		
**Description**	***m/z***	**Assignment**	**Dev. (ppm)**	***m/z***	**Assignment**	**Dev. (ppm)**	***m/z***	**Assignment**	**Dev. (ppm)**	***m/z***	**Assignment**	**Dev. (ppm)**
GKT				289.1871	$C_{12}H_{25}N_4O_4^+$	0.2837						
GKTF	408.2605	$C_{20}H_{34}N_5O_4^+$	-0.0733				453.2821	$C_{21}H_{37}N_6O_5^+$	0.3030			
GKTY	424.2556	$C_{20}H_{34}N_5O_5^+$	0.4209	452.2496	$C_{21}H_{34}N_5O_6^+$	-1.5723	469.2770	$C_{21}H_{37}N_6O_6^+$	0.1633	407.2292	$C_{20}H_{31}N_4O_5^+$	0.6404
GKTYH	561.3142	$C_{26}H_{41}N_8O_6^+$	-0.2915	589.3083	$C_{27}H_{41}N_8O_7^+$	-1.6304	606.3359	$C_{27}H_{44}N_9O_7^+$	0.1176			
GKTYHV	660.3830	$C_{31}H_{50}N_9O_7^+$	0.3816	688.3773	$C_{32}H_{50}N_9O_8^+$	-0.5289	705.4044	$C_{32}H_{53}N_{10}O_8^+$	0.1890			
GKTYHVG	717.4042	$C_{33}H_{53}N_{10}O_8^+$	-0.0330	745.3989	$C_{34}H_{53}N_{10}O_9^+$	-0.3607	762.4254	$C_{34}H_{56}N_{11}O_9^+$	-0.4023			
GKTYHVGE	846.4464	$C_{38}H_{60}N_{11}O_{11}^+$	-0.5551	874.4417	$C_{39}H_{60}N_{11}O_{12}^+$	-0.0853	891.4690	$C_{39}H_{63}N_{12}O_{12}^+$	0.8412	829.4192	$C_{38}H_{57}N_{10}O_{11}^+$	-1.2791
GKTYHVGEQ	974.5066	$C_{43}H_{68}N_{13}O_{13}^+$	1.2247	1002.4989	$C_{44}H_{68}N_{13}O_{14}^+$	-1.4256						

Supplementary Table 143 Peak list exported from SurfaceLab spectrum of bovine fibronectin, consisting of ions detected in the spectrum and assigned as internal fragments of the sequence YRIGDT. The *m/z* values represent the experimentally observed center mass of each peak. The deviation (dev.) represents the parts per million (ppm) accuracy of the assignment.

	ya			yb			yc			ya-NH3		
**Description**	***m/z***	**Assignment**	**Dev. (ppm)**	***m/z***	**Assignment**	**Dev. (ppm)**	***m/z***	**Assignment**	**Dev. (ppm)**	***m/z***	**Assignment**	**Dev. (ppm)**
YR	294.1926	$C_{14}H_{24}N_5O_2^+$	0.6677	322.1876	$C_{15}H_{24}N_5O_3^+$	0.6753	339.2140	$C_{15}H_{27}N_6O_3^+$	0.2591	277.1661	$C_{14}H_{21}N_4O_2^+$	0.5619
YRI	407.2765	$C_{20}H_{35}N_6O_3^+$	-0.1251	435.2716	$C_{21}H_{35}N_6O_4^+$	0.4055	452.2981	$C_{21}H_{38}N_7O_4^+$	0.3102	390.2503	$C_{20}H_{32}N_5O_3^+$	0.7841
YRIG	464.2987	$C_{22}H_{38}N_7O_4^+$	1.6007	492.2930	$C_{23}H_{38}N_7O_5^+$	0.1228	509.3199	$C_{23}H_{41}N_8O_5^+$	0.8569	447.2718	$C_{22}H_{35}N_6O_4^+$	0.7992
YRIGD	579.3253	$C_{26}H_{43}N_8O_7^+$	0.6210	607.3193	$C_{27}H_{43}N_8O_8^+$	-0.8445	624.3467	$C_{27}H_{46}N_9O_8^+$	0.5053	562.2972	$C_{26}H_{40}N_7O_7^+$	-2.0622
YRIGDT	680.3726	$C_{30}H_{50}N_9O_9^+$	0.0501	708.3676	$C_{31}H_{50}N_9O_{10}^+$	0.0775	725.3942	$C_{31}H_{53}N_{10}O_{10}^+$	0.1870	663.3453	$C_{30}H_{47}N_8O_9^+$	-1.0894

Supplementary Table 144 Peak list exported from SurfaceLab spectrum of bovine fibronectin, consisting of ions detected in the spectrum and assigned as internal fragments of the sequence YERPKDS. The *m/z* values represent the experimentally observed center mass of each peak. The deviation (dev.) represents the parts per million (ppm) accuracy of the assignment.

	ya			yb			yc			ya-NH3		
**Description**	***m/z***	**Assignment**	**Dev. (ppm)**	***m/z***	**Assignment**	**Dev. (ppm)**	***m/z***	**Assignment**	**Dev. (ppm)**	***m/z***	**Assignment**	**Dev. (ppm)**
YE	289.1159	$C_{13}H_{18}N_2O_4Na^+$	0.0374	317.1110	$C_{14}H_{18}N_2O_5Na^+$	0.7542	334.1374	$C_{14}H_{21}N_3O_5Na^+$	0.2102			
YER	445.2169	$C_{19}H_{30}N_6O_5Na^+$	-0.2710	473.2123	$C_{20}H_{30}N_6O_6Na^+$	0.7923	490.2395	$C_{20}H_{33}N_7O_6Na^+$	2.2145	428.1904	$C_{19}H_{27}N_5O_5Na^+$	0.0176
YERP	542.2695	$C_{24}H_{37}N_7O_6Na^+$	-0.4902	570.2644	$C_{25}H_{37}N_7O_7Na^+$	-0.4514	587.2917	$C_{25}H_{40}N_8O_7Na^+$	0.8455	525.2437	$C_{24}H_{34}N_6O_6Na^+$	0.9477
YERPK	670.3647	$C_{30}H_{49}N_9O_7Na^+$	0.0463	698.3599	$C_{31}H_{49}N_9O_8Na^+$	0.3603	715.3871	$C_{31}H_{52}N_{10}O_8Na^+$	1.2870	653.3373	$C_{30}H_{46}N_8O_7Na^+$	-1.3380
YERPKD	785.3925	$C_{34}H_{54}N_{10}O_{10}Na^+$	1.0730	813.3878	$C_{35}H_{54}N_{10}O_{11}Na^+$	1.4984	830.4144	$C_{35}H_{57}N_{11}O_{11}Na^+$	1.5091	768.3660	$C_{34}H_{51}N_9O_{10}Na^+$	1.1430
YERPKDS	872.4259	$C_{37}H_{59}N_{11}O_{12}Na^+$	2.5710				917.4483	$C_{38}H_{62}N_{12}O_{13}Na^+$	3.3794			

Supplementary Table 145 Peak list exported from SurfaceLab spectrum of bovine fibronectin, consisting of ions detected in the spectrum and assigned as internal fragments of the sequence WSKKDNRGNLL. The *m/z* values represent the experimentally observed center mass of each peak. The deviation (dev.) represents the parts per million (ppm) accuracy of the assignment.

	ya			yb			yc			ya-NH3		
**Description**	***m/z***	**Assignment**	**Dev. (ppm)**	***m/z***	**Assignment**	**Dev. (ppm)**	***m/z***	**Assignment**	**Dev. (ppm)**	***m/z***	**Assignment**	**Dev. (ppm)**
WS										315.1429	$C_{14}H_{20}N_4O_3Na^+$	0.2866
WSK	412.2192	$C_{19}H_{29}N_6O_3Na^+$	-0.3139	440.2134	$C_{20}H_{29}N_6O_4Na^+$	-2.0386	457.2408	$C_{20}H_{32}N_7O_4Na^+$	0.0423	395.1930	$C_{19}H_{26}N_5O_3Na^+$	0.5732
WSKK	526.3101	$C_{25}H_{41}N_7O_4Na^+$	-2.1385	554.3051	$C_{26}H_{41}N_7O_5Na^+$	-1.7888	571.3328	$C_{26}H_{44}N_8O_5Na^+$	0.2509	509.2830	$C_{25}H_{38}N_6O_4Na^+$	-3.3184
WSKKD	641.3377	$C_{29}H_{46}N_8O_7Na^+$	-0.6784	669.3341	$C_{30}H_{46}N_8O_8Na^+$	1.5040	686.3604	$C_{30}H_{49}N_9O_8Na^+$	1.1747	624.3105	$C_{29}H_{43}N_7O_7Na^+$	-1.7559
WSKKDN	756.3888	$C_{33}H_{53}N_{10}O_9Na^+$	-0.1246	784.3850	$C_{34}H_{53}N_{10}O_{10}Na^+$	1.4960	801.4113	$C_{34}H_{56}N_{11}O_{10}Na^+$	1.1682	739.3612	$C_{33}H_{50}N_9O_9Na^+$	-1.6438
WSKKDNR	911.4819	$C_{39}H_{64}N_{14}O_{10}Na^+$	-0.3742	939.4768	$C_{40}H_{64}N_{14}O_{11}Na^+$	-0.2985						
WSKKDNRG	968.5045	$C_{41}H_{67}N_{15}O_{11}Na^+$	0.8131	996.4967	$C_{42}H_{67}N_{15}O_{12}Na^+$	-1.9162				951.4775	$C_{41}H_{64}N_{14}O_{11}Na^+$	0.3541
WSKKDNRGN	1082.5470	$C_{45}H_{73}N_{17}O_{13}Na^+$	0.3464									
WSKKDNRGNL	1195.6287	$C_{51}H_{84}N_{18}O_{14}Na^+$	-1.6124	1223.6152	$C_{52}H_{84}N_{18}O_{15}Na^+$	-8.5171						
WSKKDNRGNLL				1336.7013	$C_{58}H_{95}N_{19}O_{16}Na^+$	-6.2546						

Supplementary Table 146 Peak list exported from SurfaceLab spectrum of bovine fibronectin, assigned as internal fragments of the sequence CYGRGIGE. The *m/z* values represent the experimentally observed center mass of each peak. The deviation (dev.) represents the parts per million (ppm) accuracy of the assignment.

	ya			yb			yc			ya-NH3		
**Description**	***m/z***	**Assignment**	**Dev. (ppm)**	***m/z***	**Assignment**	**Dev. (ppm)**	***m/z***	**Assignment**	**Dev. (ppm)**	***m/z***	**Assignment**	**Dev. (ppm)**
GE	161.0919	$C_6H_{13}N_2O_3^+$	-0.8162	189.0869	$C_7H_{13}N_2O_4^+$	-0.5933						
IGE	274.1762	$C_{12}H_{24}N_3O_4^+$	0.3238	302.1712	$C_{13}H_{24}N_3O_5^+$	0.6123				257.1496	$C_{12}H_{21}N_2O_4^+$	0.2225
GIGE	331.1976	$C_{14}H_{27}N_4O_5^+$	0.0274	359.1925	$C_{15}H_{27}N_4O_6^+$	-0.1682				314.1712	$C_{14}H_{24}N_3O_5^+$	0.3848
RGIGE	487.2990	$C_{20}H_{39}N_8O_6^+$	0.6022	515.2941	$C_{21}H_{39}N_8O_7^+$	0.8941				470.2725	$C_{20}H_{36}N_7O_6^+$	0.8282
GRGIGE	544.3209	$C_{22}H_{42}N_9O_7^+$	1.4218	572.3160	$C_{23}H_{42}N_9O_8^+$	1.6142				527.2942	$C_{22}H_{39}N_8O_7^+$	1.0630
YGRGIGE	707.3838	$C_{31}H_{51}N_{10}O_9^+$	0.4830	735.3783	$C_{32}H_{51}N_{10}O_{10}^+$	-0.1706				690.3555	$C_{31}H_{48}N_9O_9^+$	-2.0554
CYGRGIGE -S	778.4209	$C_{34}H_{56}N_{11}O_{10}^+$	0.3146	806.4160	$C_{35}H_{56}N_{11}O_{11}^+$	0.5405						
CYGRGIGE -SH	777.4121	$C_{34}H_{55}N_{11}O_{10}^+$	-0.9284	805.4070	$C_{35}H_{55}N_{11}O_{11}^+$	-0.8160						
CYGRGIGE -SH2	776.4043	$C_{34}H_{54}N_{11}O_{10}^+$	-0.8656	804.4004	$C_{35}H_{54}N_{11}O_{11}^+$	0.6412						

Supplementary Table 147 Peak list exported from SurfaceLab spectrum of bovine fibronectin, assigned as internal fragments of the sequence PPPRDLQFV. The *m/z* values represent the experimentally observed center mass of each peak. The deviation (dev.) represents the parts per million (ppm) accuracy of the assignment.

	ya			yb			yc			ya-NH3		
**Description**	***m/z***	**Assignment**	**Dev. (ppm)**	***m/z***	**Assignment**	**Dev. (ppm)**	***m/z***	**Assignment**	**Dev. (ppm)**	***m/z***	**Assignment**	**Dev. (ppm)**
PP	169.1334	$C_9H_{17}N_2O^+$	-0.9378	197.1284	$C_{10}H_{17}N_2O_2^+$	-0.4606	214.1550	$C_{10}H_{20}N_3O_2^+$	0.0804	152.1069	$C_9H_{14}NO^+$	-0.5506
PPP	266.1864	$C_{14}H_{24}N_3O_2^+$	0.4696	294.1814	$C_{15}H_{24}N_3O_3^+$	0.6388	311.2079	$C_{15}H_{27}N_4O_3^+$	0.4350	249.1599	$C_{14}H_{21}N_2O_2^+$	0.5496
PPPR				450.2825	$C_{21}H_{36}N_7O_4^+$	0.2725	467.3093	$C_{21}H_{39}N_8O_4^+$	0.8420	405.2610	$C_{20}H_{33}N_6O_3^+$	0.3262
PPPRD	537.3146	$C_{24}H_{41}N_8O_6^+$	0.4587	565.3092	$C_{25}H_{41}N_8O_7^+$	-0.1238	582.3361	$C_{25}H_{44}N_9O_7^+$	0.3961	520.2874	$C_{24}H_{38}N_7O_6^+$	-0.8586
PPPRDL	650.3987	$C_{30}H_{52}N_9O_7^+$	0.3761	678.3934	$C_{31}H_{52}N_9O_8^+$	0.0830	695.4203	$C_{31}H_{55}N_{10}O_8^+$	0.6665	633.3715	$C_{30}H_{49}N_8O_7^+$	-0.6627
PPPRDLQ	778.4575	$C_{35}H_{60}N_{11}O_9^+$	0.6598	806.4518	$C_{36}H_{60}N_{11}O_{10}^+$	-0.1404				761.4302	$C_{35}H_{57}N_{10}O_9^+$	-0.2763
PPPRDLQF	925.5254	$C_{44}H_{69}N_{12}O_{10}^+$	0.0127									
PPPRDLQFV				1052.5836	$C_{50}H_{78}N_{13}O_{12}^+$	-4.8629	1069.6046	$C_{50}H_{81}N_{14}O_{12}^+$	-10.0336			

Negative mode

α-chymotrypsin

Supplementary Table 148 Peak list exported from SurfaceLab negative mode spectrum of bovine α-chymotrypsin, consisting of ions detected in the spectrum and assigned fragments of C-terminal sequence GVPAIQPVLSGL. The *m/z* values represent the experimentally observed center mass of each peak. The deviation (dev.) represents the parts per million (ppm) accuracy of the assignment. The colour corresponds to the presence of the observed sequence in human insulin presented in Supplementary Figure 12.

	**y**			**z-H**			**x**		
**Description**	***m/z***	**Assignment**	**Dev. (ppm)**	***m/z***	**Assignment**	**Dev. (ppm)**	***m/z***	**Assignment**	**Dev. (ppm)**
GL	187.1090	$C_8H_{15}N_2O_3^-$	0.8439						
SGL	274.1414	$C_{11}H_{20}N_3O_5^-$	1.8715						
LSGL	387.2251	$C_{17}H_{31}N_4O_6^-$	0.5689						
VLSGL	486.2937	$C_{22}H_{40}N_5O_7^-$	0.7638	471.2828	$C_{22}H_{39}N_4O_7^-$	0.8396	514.2884	$C_{23}H_{40}N_5O_8^-$	0.2331
PVLSGL	583.3467	$C_{27}H_{47}N_6O_8^-$	1.1188				611.3416	$C_{28}H_{47}N_6O_9^-$	0.9268
QPVLSGL	711.4057	$C_{32}H_{55}N_8O_{10}^-$	1.5194	696.3948	$C_{32}H_{54}N_7O_{10}^-$	1.4593	739.4006	$C_{33}H_{55}N_8O_{11}^-$	1.4451
IQPVLSGL	824.4900	$C_{38}H_{66}N_9O_{11}^-$	1.5137	809.4790	$C_{38}H_{65}N_8O_{11}^-$	1.4908	852.4847	$C_{39}H_{66}N_9O_{12}^-$	1.2615
AIQPVLSGL	895.5269	$C_{41}H_{71}N_{10}O_{12}^-$	1.2357	880.5159	$C_{41}H_{70}N_9O_{12}^-$	1.0428	923.5219	$C_{42}H_{71}N_{10}O_{13}^-$	1.2279
PAIQPVLSGL	992.5797	$C_{46}H_{78}N_{11}O_{13}^-$	1.1400				1020.5744	$C_{47}H_{78}N_{11}O_{14}^-$	0.8362
VPAIQPVLSGL	1091.6485	$C_{51}H_{87}N_{12}O_{14}^-$	1.3571	1076.6372	$C_{51}H_{86}N_{11}O_{14}^-$	1.0460	1119.6432	$C_{52}H_{87}N_{12}O_{15}^-$	1.1113
GVPAIQPVLSGL	1148.6696	$C_{53}H_{90}N_{13}O_{15}^-$	0.9667	1133.6590	$C_{53}H_{89}N_{12}O_{15}^-$	1.2792	1176.6644	$C_{54}H_{90}N_{13}O_{16}^-$	0.8594
CGVPAIQPVLSGL	1251.6794	$C_{56}H_{95}N_{14}O_{16}S^-$	1.3940						
CGVPAIQPVLSGL -S	1219.7036	$C_{56}H_{95}N_{14}O_{16}^-$	-1.6487						
CGVPAIQPVLSGL -SH_2	1217.6910	$C_{56}H_{93}N_{14}O_{16}^-$	0.9050						

Supplementary Table 149 Peak list exported from SurfaceLab negative mode spectrum of bovine α-chymotrypsin, consisting of ions detected in the spectrum and assigned fragments of N-terminal sequence IVNGEEAVPGSW. The *m/z* values represent the experimentally observed center mass of each peak. The deviation (dev.) represents the parts per million (ppm) accuracy of the assignment. The colour corresponds to the presence of the observed sequence in human insulin presented in Supplementary Figure 12.

	a			b			c			a-NH3		
**Description**	***m/z***	**Assignment**	**Dev. (ppm)**	***m/z***	**Assignment**	**Dev. (ppm)**	***m/z***	**Assignment**	**Dev. (ppm)**	***m/z***	**Assignment**	**Dev. (ppm)**
IV	183.1504	$C_{10}H_{19}N_2O^-$	0.5586	211.1455	$C_{11}H_{19}N_2O_2^-$	1.1798	228.1720	$C_{11}H_{22}N_3O_2^-$	1.0457			
IVN	297.1934	$C_{14}H_{25}N_4O_3^-$	0.6659	325.1884	$C_{15}H_{25}N_4O_4^-$	0.7366	342.2148	$C_{15}H_{28}N_5O_4^-$	0.3728	280.1670	$C_{14}H_{22}N_3O_3^-$	1.0874
IVNG	354.2150	$C_{16}H_{28}N_5O_4^-$	0.7767	382.2098	$C_{17}H_{28}N_5O_5^-$	0.6061	399.2365	$C_{17}H_{31}N_6O_5^-$	0.8879	337.1884	$C_{16}H_{25}N_4O_4^-$	0.7564
IVNGE	483.2576	$C_{21}H_{35}N_6O_7^-$	0.6732	511.2525	$C_{22}H_{35}N_6O_8^-$	0.6275	528.2792	$C_{22}H_{38}N_7O_8^-$	0.8620	466.2305	$C_{21}H_{32}N_5O_7^-$	-0.5378
IVNGEE	612.3005	$C_{26}H_{42}N_7O_{10}^-$	1.0063	640.2960	$C_{27}H_{42}N_7O_{11}^-$	1.9194	657.3222	$C_{27}H_{45}N_8O_{11}^-$	1.3301			
IVNGEEA	683.3379	$C_{29}H_{47}N_8O_{11}^-$	1.3201	711.3331	$C_{30}H_{47}N_8O_{12}^-$	1.6711	728.3595	$C_{30}H_{50}N_9O_{12}^-$	1.4595			
IVNGEEAV	782.4065	$C_{34}H_{56}N_9O_{12}^-$	1.3930	810.4015	$C_{35}H_{56}N_9O_{13}^-$	1.4936	827.4279	$C_{35}H_{59}N_{10}O_{13}^-$	1.2157			
IVNGEEAVP	879.4592	$C_{39}H_{63}N_{10}O_{13}^-$	1.2019				924.4804	$C_{40}H_{66}N_{11}O_{14}^-$	0.8872			
IVNGEEAVPG	936.4801	$C_{41}H_{66}N_{11}O_{14}^-$	0.4924	964.4753	$C_{42}H_{66}N_{11}O_{15}^-$	0.8220	981.5019	$C_{42}H_{69}N_{12}O_{15}^-$	0.8214			
IVNGEEAVPGS	1023.5125	$C_{44}H_{71}N_{12}O_{16}^-$	0.8077	1051.5074	$C_{45}H_{71}N_{12}O_{17}^-$	0.7635	1068.5346	$C_{45}H_{74}N_{13}O_{17}^-$	1.3460			
IVNGEEAVPGSW	1209.5924	$C_{55}H_{81}N_{14}O_{17}^-$	1.1872									

Supplementary Table 150 Peak list exported from SurfaceLab negative mode spectrum of bovine α-chymotrypsin, consisting of ions detected in the spectrum and assigned fragments of C-terminal sequence TGWGLTRY. The *m/z* values represent the experimentally observed center mass of each peak. The deviation (dev.) represents the parts per million (ppm) accuracy of the assignment. The colour corresponds to the presence of the observed sequence in human insulin presented in Supplementary Figure 12.

	y			z-H			x		
**Description**	***m/z***	**Assignment**	**Dev. (ppm)**	***m/z***	**Assignment**	**Dev. (ppm)**	***m/z***	**Assignment**	**Dev. (ppm)**
TRY	437.2158	$C_{19}H_{29}N_6O_6^-$	0.8593						
LTRY	x								
GLTRY	607.3218	$C_{27}H_{43}N_8O_8^-$	1.3981				635.3164	$C_{28}H_{43}N_8O_9^-$	0.9016
WGLTRY	793.4015	$C_{38}H_{53}N_{10}O_9^-$	1.6274						
GWGLTRY	850.4228	$C_{40}H_{56}N_{11}O_{10}^-$	1.2966				878.4181	$C_{41}H_{56}N_{11}O_{11}^-$	1.6993
TGWGLTRY	951.4705	$C_{44}H_{63}N_{12}O_{12}^-$	1.2069				979.4663	$C_{45}H_{63}N_{12}O_{13}^-$	2.0047

Supplementary Table 151 Peak list exported from SurfaceLab negative mode spectrum of bovine α-chymotrypsin, consisting of ions detected in the spectrum and assigned fragments of N-terminal sequence ANTPDRL. The *m/z* values represent the experimentally observed center mass of each peak. The deviation (dev.) represents the parts per million (ppm) accuracy of the assignment. The colour corresponds to the presence of the observed sequence in human insulin presented in Supplementary Figure 12.

	**a**			**b**			**c**		
**Description**	***m/z***	**Assignment**	**Dev. (ppm)**	***m/z***	**Assignment**	**Dev. (ppm)**	***m/z***	**Assignment**	**Dev. (ppm)**
AN	156.0780	$C_6H_{10}N_3O_2^-$	0.7434	184.0729	$C_7H_{10}N_3O_3^-$	0.6210	201.0996	$C_7H_{13}N_4O_3^-$	1.1741
ANT	257.1258	$C_{10}H_{17}N_4O_4^-$	1.1870	285.1208	$C_{11}H_{17}N_4O_5^-$	1.2729	302.1472	$C_{11}H_{20}N_5O_5^-$	0.5454
ANTP	354.1786	$C_{15}H_{24}N_5O_5^-$	0.9245	382.1736	$C_{16}H_{24}N_5O_6^-$	0.9252	399.2000	$C_{16}H_{27}N_6O_6^-$	0.6363
ANTPD	469.2055	$C_{19}H_{29}N_6O_8^-$	0.4802	497.2007	$C_{20}H_{29}N_6O_9^-$	1.1999			
ANTPDR	x								
ANTPDRL	738.3913	$C_{31}H_{52}N_{11}O_{10}^-$	1.2427				783.4106	$C_{32}H_{55}N_{12}O_{11}^-$	-1.6717

Supplementary Table 152 Peak list exported from SurfaceLab negative mode spectrum of bovine α-chymotrypsin, consisting of ions detected in the spectrum and assigned fragments of C-terminal sequence TALVNWVQQTLAAN. The *m/z* values represent the experimentally observed center mass of each peak. The deviation (dev.) represents the parts per million (ppm) accuracy of the assignment. The colour corresponds to the presence of the observed sequence in human insulin presented in Supplementary Figure 12.

	y			z-H			x		
**Description**	***m/z***	**Assignment**	**Dev. (ppm)**	***m/z***	**Assignment**	**Dev. (ppm)**	***m/z***	**Assignment**	**Dev. (ppm)**
AN	x			187.0726	$C_{7}H_{11}N_{2}O_{4}^{-}$	0.7264			
AAN	x			258.1098	$C_{10}H_{16}N_{3}O_{5}^{-}$	1.0986			
LAAN	x			371.1935	$C_{16}H_{27}N_{4}O_{6}^{-}$	-0.2595			
TLAAN	x			472.2414	$C_{20}H_{34}N_{5}O_{8}^{-}$	0.2003			
QTLAAN	x			600.3005	$C_{25}H_{42}N_{7}O_{10}^{-}$	1.1395			
QQTLAAN	743.3710	$C_{30}H_{51}N_{10}O_{12}^{-}$	2.2270	728.3595	$C_{30}H_{50}N_{9}O_{12}^{-}$	1.4595	771.3654	$C_{31}H_{51}N_{10}O_{13}^{-}$	1.5380
VQQTLAAN	842.4389	$C_{35}H_{60}N_{11}O_{13}^{-}$	1.3952	827.4279	$C_{35}H_{59}N_{10}O_{13}^{-}$	1.2157	870.4337	$C_{36}H_{60}N_{11}O_{14}^{-}$	1.1739
WVQQTLAAN	1028.5179	$C_{46}H_{70}N_{13}O_{14}^{-}$	0.7758	1013.5070	$C_{46}H_{69}N_{12}O_{14}^{-}$	0.7728	1056.5129	$C_{47}H_{70}N_{13}O_{15}^{-}$	0.8869
NWVQQTLAAN	x			1127.5506	$C_{50}H_{75}N_{14}O_{16}^{-}$	1.3082	1170.5565	$C_{51}H_{76}N_{15}O_{17}^{-}$	1.3377
VNWVQQTLAAN	1241.6290	$C_{55}H_{85}N_{16}O_{17}^{-}$	0.4496	1226.6187	$C_{55}H_{84}N_{15}O_{17}^{-}$	0.9778	1269.6252	$C_{56}H_{85}N_{16}O_{18}^{-}$	1.4429
LVNWVQQTLAAN	1354.7145	$C_{61}H_{96}N_{17}O_{18}^{-}$	1.5284	1339.7028	$C_{61}H_{95}N_{16}O_{18}^{-}$	0.8937	1382.7075	$C_{62}H_{96}N_{17}O_{19}^{-}$	0.0865
ALVNWVQQTLAAN	1425.7515	$C_{64}H_{101}N_{18}O_{19}^{-}$	1.3644	1410.7400	$C_{64}H_{100}N_{17}O_{19}^{-}$	0.9025	1453.7453	$C_{65}H_{101}N_{18}O_{20}^{-}$	0.5211
TALVNWVQQTLAAN	1526.7994	$C_{68}H_{108}N_{19}O_{21}^{-}$	1.3900	1511.7875	$C_{68}H_{107}N_{18}O_{21}^{-}$	0.7344			

Supplementary References

1. Wagner, M. S. & Castner, D. G. Characterization of Adsorbed Protein Films by Time-of-Flight Secondary Ion Mass Spectrometry with Principal Component Analysis. *Langmuir* **17**, 4649–4660 (2001).

2. Zubarev, R. A. & Makarov, A. Orbitrap Mass Spectrometry. *Anal. Chem.* **85**, 5288–5296 (2013).

3. Shard, A. G. A straightforward method for interpreting XPS data from core-shell nanoparticles. *J. Phys. Chem. C* **116**, 16806–16813 (2012).

4. Ray, S. *et al.* Neutralized chimeric avidin binding at a reference biosensor surface. *Langmuir* **31**, 1921–1930 (2015).

5. Starr, N. J. *et al.* Enhanced vitamin C skin permeation from supramolecular hydrogels, illustrated using in situ ToF-SIMS 3D chemical profiling. *Int. J. Pharm.* **563**, 21–29 (2019).

6. Tran, N. H., Zhang, X., Xin, L., Shan, B. & Li, M. De novo peptide sequencing by deep learning. *Proc. Natl. Acad. Sci. U. S. A.* **114**, 8247–8252 (2017).

7. Timofeev, V. I. *et al.* X-ray investigation of gene-engineered human insulin crystallized from a solution containing polysialic acid. *Acta Crystallogr. Sect. F Struct. Biol. Cryst. Commun.* **66**, 259–263 (2010).

8. Qi, P. X., Beckman, R. A. & Joshua Wand, A. Solution structure of horse heart ferricytochrome c and detection of redox-related structural changes by high-resolution 1H NMR. *Biochemistry* **35**, 12275–12286 (1996).

9. Lim, K., Nadarajah, A., Forsytiie, E. L. & Pusey, M. L. Locations of bromide ions in tetragonal lysozyme crystals. *Acta Crystallogr. Sect. D Biol. Crystallogr.* **54**, 899–904 (1998).

10. Maurus, R. *et al.* A myoglobin variant with a polar substitution in a conserved hydrophobic cluster in the heme binding pocket. *Biochim. Biophys. Acta - Protein Struct. Mol. Enzymol.* **1341**, 1–13 (1997).

11. Huang, Q., Wang, Z., Li, Y., Liu, S. & Tang, Y. Refined 1.8Å resolution crystal structure of the porcine ϵ-trypsin. *Biochim. Biophys. Acta - Protein Struct. Mol. Enzymol.* **1209**, 77–82 (1994).

12. Sielecki, A. R., Fedorov, A. A., Boodhoo, A., Andreeva, N. S. & James, M. N. G. Molecular and crystal structures of monoclinic porcine pepsin refined at 1.8A˚resolution. *J. Mol. Biol.* **214**, 143–170 (1990).

13. Kashima, A. *et al.* X-ray crystal structure of a dipeptide-chymotrypsin complex in an inhibitory interaction. *Eur. J. Biochem.* **255**, 12–23 (1998).

14. Raj, S. B., Ramaswamy, S. & Plapp, B. V. Yeast Alcohol Dehydrogenase Structure and Catalysis. *Biochemistry* **53**, 5791–5803 (2014).

15. Parkin, S., Rupp, B. & Hope, H. Atomic Resolution Structure of Concanavalin A at 120 K. *Acta Crystallogr. Sect. D Biol. Crystallogr.* **52**, 1161–1168 (1996).

16. Bujacz, A. Structures of bovine, equine and leporine serum albumin. *Acta Crystallogr. Sect. D Biol. Crystallogr.* **68**, 1278–1289 (2012).

17. Sugio, S., Kashima, A., Mochizuki, S., Noda, M. & Kobayashi, K. Crystal structure of human serum albumin at 2.5 Å resolution. *Protein Eng. Des. Sel.* **12**, 439–446 (1999).

18. Alam, M. T. *et al.* The self-inhibitory nature of metabolic networks and its alleviation through

compartmentalization. *Nat. Commun.* **8**, 16018 (2017).

19. Purwar, N., McGarry, J. M., Kostera, J., Pacheco, A. A. & Schmidt, M. Interaction of Nitric Oxide with Catalase: Structural and Kinetic Analysis. *Biochemistry* **50**, 4491–4503 (2011).
