# Supplementary material for: Protein identification by 3D OrbiSIMS to facilitate in situ imaging and depth profiling
Source: Nat Commun. 2020 Nov 17;11:5832. doi: 10.1038/s41467-020-19445-x (PMC7672064; doi:10.1038/s41467-020-19445-x)
Supplement: Supplementary file 2 — Reporting Summary [file 41467_2020_19445_MOESM2_ESM.pdf]

nature research

Corresponding author(s): David Scurr

Last updated by author(s): Oct 8, 2020

Reporting Summary

Nature Research wishes to improve the reproducibility of the work that we publish. This form provides structure for consistency and transparency in reporting. For further information on Nature Research policies, see our Editorial Policies and the Editorial Policy Checklist.

Statistics

For all statistical analyses, confirm that the following items are present in the figure legend, table legend, main text, or Methods section.

n/a	Confirmed	
x		The exact sample size (*n*) for each experimental group/condition, given as a discrete number and unit of measurement
x		A statement on whether measurements were taken from distinct samples or whether the same sample was measured repeatedly
x		The statistical test(s) used AND whether they are one- or two-sided<br>*Only common tests should be described solely by name; describe more complex techniques in the Methods section.*
x		A description of all covariates tested
x		A description of any assumptions or corrections, such as tests of normality and adjustment for multiple comparisons
	x	A full description of the statistical parameters including central tendency (e.g. means) or other basic estimates (e.g. regression coefficient) AND variation (e.g. standard deviation) or associated estimates of uncertainty (e.g. confidence intervals)
x		For null hypothesis testing, the test statistic (e.g. *F*, *t*, *r*) with confidence intervals, effect sizes, degrees of freedom and *P* value noted<br>*Give P values as exact values whenever suitable.*
x		For Bayesian analysis, information on the choice of priors and Markov chain Monte Carlo settings
x		For hierarchical and complex designs, identification of the appropriate level for tests and full reporting of outcomes
x		Estimates of effect sizes (e.g. Cohen's *d*, Pearson's *r*), indicating how they were calculated

Our web collection on statistics for biologists contains articles on many of the points above.

Software and code

Policy information about availability of computer code

Data collection	3D OrbiSIMS spectra were acquired using IonToF SurfaceLab 7.1.116182.
Data analysis	Thermo Xcalibur 3.1.66.10 and IonToF SurfaceLab 7.1.116182 were used to process the results and assign the peaks. Xcalibur was used to identify amino acid neutral losses between peaks and SurfaceLab was used to create the peak lists. SurfaceLab was used to measure lateral resolution of ToF-SIMS and 3D OrbiSIMS images. Ellipsometry results were analysed using CompleteEASE 4.06. XPS results were analysed using CasaXPS 2.3.19PR1.0. Protein illustrations with highlighted sequences were generated using PyMol (TM) 2.3.2. Proposed chemical structures of observed protein fragments were produced using ChemDraw Professional 16.0. A custom code was written in MATLAB for automated sequence assignment and protein identification. The code is available on Github link: https://github.com/guferraz/simsdenovo/

For manuscripts utilizing custom algorithms or software that are central to the research but not yet described in published literature, software must be made available to editors and reviewers. We strongly encourage code deposition in a community repository (e.g. GitHub). See the Nature Research guidelines for submitting code & software for further information.

Data

Policy information about availability of data

All manuscripts must include a data availability statement. This statement should provide the following information, where applicable:

- Accession codes, unique identifiers, or web links for publicly available datasets
- A list of figures that have associated raw data
- A description of any restrictions on data availability

All data will has been uploaded to Nottingham Data Repository and is available under: DOI: 10.17639/nott.7070 or https://rdmc.nottingham.ac.uk/handle/internal/8604. Protein sequences were obtained from UniProt database (28th July 2019) https://www.uniprot.org/. Protein 3D structures were obtained from The

Protein Data Bank http://www.rcsb.org/ and source publications are cited with each structure in the Supplementary Information. Protein location in the human skin was based on the Human Protein Atlas available from http://www.proteinatlas.org.

Field-specific reporting

Please select the one below that is the best fit for your research. If you are not sure, read the appropriate sections before making your selection.

[x] Life sciences [ ] Behavioural & social sciences [ ] Ecological, evolutionary & environmental sciences

For a reference copy of the document with all sections, see nature.com/documents/nr-reporting-summary-flat.pdf

Life sciences study design

All studies must disclose on these points even when the disclosure is negative.

Sample size	Two different areas were analyzed on each of sixteen investigated protein films. Two different areas were analyzed on the protein biochip samples and each of biochip control samples. Two different areas were analyzed on the skin sample. The aim of this study was to develop a method of assigning protein fragment peaks in the reference spectra and demonstrate the method in example biological applications. This study did not include statistical differences between different protein spectra. Only peak presence, not the intensity was relevant in this study and in all cases, reference samples and applications, the detected peaks were present consistently in two measurements.
Data exclusions	No data was excluded from the analyses.
Replication	All attempts at replication of all of the experiments were successful. The experiments were performed by one operator (A.K. or D.S.) on one instrument, on different days.
Randomization	Samples were not randomised. Randomisation is not relevant to this study. The aim of this work was to demonstrate protein fragment assignment, not any biological differences between the samples.
Blinding	There was no group allocation involved in this study.

Reporting for specific materials, systems and methods

We require information from authors about some types of materials, experimental systems and methods used in many studies. Here, indicate whether each material, system or method listed is relevant to your study. If you are not sure if a list item applies to your research, read the appropriate section before selecting a response.

Materials & experimental systems

n/a	Involved in the study	
[x]	[ ]	Antibodies
[x]	[ ]	Eukaryotic cell lines
[x]	[ ]	Palaeontology and archaeology
[x]	[ ]	Animals and other organisms
[x]	[ ]	Human research participants
[x]	[ ]	Clinical data
[x]	[ ]	Dual use research of concern

Methods

n/a	Involved in the study	
[x]	[ ]	ChIP-seq
[x]	[ ]	Flow cytometry
[x]	[ ]	MRI-based neuroimaging
